# Tree mortality submodels drive simulated long‐term forest dynamics: assessing 15 models from the stand to global scale

**DOI:** 10.1002/ecs2.2616

**Published:** 2019-02-20

**Authors:** Harald Bugmann, Rupert Seidl, Florian Hartig, Friedrich Bohn, Josef Brůna, Maxime Cailleret, Louis François, Jens Heinke, Alexandra‐Jane Henrot, Thomas Hickler, Lisa Hülsmann, Andreas Huth, Ingrid Jacquemin, Chris Kollas, Petra Lasch‐Born, Manfred J. Lexer, Ján Merganič, Katarína Merganičová, Tobias Mette, Brian R. Miranda, Daniel Nadal‐Sala, Werner Rammer, Anja Rammig, Björn Reineking, Edna Roedig, Santi Sabaté, Jörg Steinkamp, Felicitas Suckow, Giorgio Vacchiano, Jan Wild, Chonggang Xu, Christopher P. O. Reyer

**Affiliations:** ^1^ Forest Ecology ETH Zürich Universitätstrasse 22 8092 Zürich Switzerland; ^2^ University of Natural Resources and Life Sciences (BOKU) Vienna Peter Jordan Straße 82 1190 Wien Austria; ^3^ Theoretical Ecology University of Regensburg Universitätsstraße 31 93053 Regensburg Germany; ^4^ Helmholtz Centre for Environmental Research – UFZ Leipzig Germany; ^5^ Institute of Meteorology and Climate Research, Atmospheric Environmental Research (IMK‐IFU) – Karlsruhe Institute of Technology Garmisch‐Partenkirchen Germany; ^6^ Institute of Botany The Czech Academy of Sciences Průhonice Czech Republic; ^7^ Research Unit Forest Dynamics Swiss Federal Institute for Forest, Snow and Landscape Research WSL Zürcherstrasse 111 8903 Birmensdorf Switzerland; ^8^ Unit for Modelling of Climate and Biogeochemical Cycles UR SPHERES University of Liège Liège Belgium; ^9^ Member of the Leibniz Association Potsdam Institute for Climate Impact Research (PIK) Potsdam Germany; ^10^ Senckenberg Biodiversity and Climate Research Centre BiK‐F Frankfurt/Main Germany; ^11^ Department of Physical Geography Goethe University Frankfurt/Main Germany; ^12^ Institute for Environmental Systems Research University of Osnabrück Osnabrück Germany; ^13^ German Centre for Integrative Biodiversity Research (iDiv) Halle‐Jena‐Leipzig Leipzig Germany; ^14^ Faculty of Forestry Technical University in Zvolen T.G. Masaryka 24 Zvolen 96053 Slovakia; ^15^ Soil and Climate Department Bavarian State Institute of Forestry (LWF) 85354 Freising Germany; ^16^ USDA Forest Service, Northern Research Station Rhinelander Wisconsin USA; ^17^ Department de Biologia Evolutiva, Ecologia i Ciències Ambientals Universitat de Barcelona Av. Diagonal 643 08028 Barcelona Spain; ^18^ TUM School of Life Sciences Weihenstephan Technical University of Munich Freising Germany; ^19^ Irstea, LESSEM Univ. Grenoble Alpes 38000 Grenoble France; ^20^ CREAF Campus de Bellaterra Edifici C 08193 Cerdanyola del Vallès Spain; ^21^ Università degli Studi di Milano, DISAA 20123 Milano Italy; ^22^ Los Alamos National Laboratory Los Alamos New Mexico 87544 USA

**Keywords:** climate change impacts, forest dynamics, model comparison, mortality modeling, succession

## Abstract

Models are pivotal for assessing future forest dynamics under the impacts of changing climate and management practices, incorporating representations of tree growth, mortality, and regeneration. Quantitative studies on the importance of mortality submodels are scarce. We evaluated 15 dynamic vegetation models (DVMs) regarding their sensitivity to different formulations of tree mortality under different degrees of climate change. The set of models comprised eight DVMs at the stand scale, three at the landscape scale, and four typically applied at the continental to global scale. Some incorporate empirically derived mortality models, and others are based on experimental data, whereas still others are based on theoretical reasoning. Each DVM was run with at least two alternative mortality submodels. Model behavior was evaluated against empirical time series data, and then, the models were subjected to different scenarios of climate change. Most DVMs matched empirical data quite well, irrespective of the mortality submodel that was used. However, mortality submodels that performed in a very similar manner against past data often led to sharply different trajectories of forest dynamics under future climate change. Most DVMs featured high sensitivity to the mortality submodel, with deviations of basal area and stem numbers on the order of 10–40% per century under current climate and 20–170% under climate change. The sensitivity of a given DVM to scenarios of climate change, however, was typically lower by a factor of two to three. We conclude that (1) mortality is one of the most uncertain processes when it comes to assessing forest response to climate change, and (2) more data and a better process understanding of tree mortality are needed to improve the robustness of simulated future forest dynamics. Our study highlights that comparing several alternative mortality formulations in DVMs provides valuable insights into the effects of process uncertainties on simulated future forest dynamics.

## Introduction

Forests have a pivotal role in providing ecosystem functions and services at multiple scales, from the global (carbon and water cycling, energy balance, biodiversity conservation) across the regional (e.g., timber production, recreation) to the local scale (e.g., protection from natural hazards such as flooding or rockfall). The ability of forest ecosystems to provide these multiple services may be jeopardized by global climate change (Settele et al. 2014). Thus, it is important to assess the future trajectories of forest ecosystems for adapting ecosystem management to climate change; quantitative, dynamic models have a key role in this regard.

A wide range of models of the dynamics of forest structure and composition at decadal to centennial time scales were developed over the past decades (cf. reviews by Shugart 1984, Liu and Ashton 1995, Bugmann 2001, Keane et al. 2015). Many of these dynamic vegetation models (DVMs) have been used to project the impacts of climate change on future forest trajectories (Solomon 1986, Elkin et al. 2013). From these studies, it is becoming increasingly evident that the impacts of climate, CO_2_, and other driving forces on growth alone would lead to fairly smooth future changes in stand structure, species composition, and other forest properties (Lloret et al. 2012, Rasche et al. 2013).

There is increasing concern, however, that mortality could lead to fast and strong changes in forest properties in the context of changing climatic conditions (Allen et al. 2010, Reyer et al. 2015). For example, carbon storage in the global forest is not just a function of growth, but is strongly determined by turnover rates, which critically depend on tree mortality (Bugmann and Bigler 2011, Friend et al. 2014). Also, other ecosystem functions and dynamics such as water fluxes and biodiversity are crucially affected by tree mortality (Anderegg et al. 2016). Changes in mortality rates are induced on the one hand by changing disturbance regimes (e.g., wind, fire, insects; Temperli et al. 2013a, Seidl et al. 2014, 2017) and, on the other hand, by stress‐related processes (e.g., drought‐induced mortality; Bigler et al. 2006, Adams et al. 2009, Allen et al. 2015).

In the past, little attention was paid to mortality in DVMs, and stress‐related mortality was most often captured using simple formulations (but cf. Bircher et al. 2015). These mortality formulations can be split into three broad categories, as briefly reviewed below.

First, some DVMs are based on theoretical mortality formulations that are not derived from data but from theoretical reasoning, the original mortality formulations of forest gap models fall under this category (Botkin et al. 1972), the self‐thinning rule (Yoda et al. 1963) as well as more process‐based approaches that assume, for example, that trees die when their carbon balance turns negative (McDowell et al. 2011, Hickler et al. 2012).

Second, other DVMs are based on empirically derived formulations that often rely on forest inventory data and use predictor variables such as tree diameter, relative growth rate, or the basal area of all trees that are larger than the target tree to feed statistical models of mortality probability (Pretzsch et al. 2002, Hlásny et al. 2014).

Third, in a few DVMs, highly mechanistic and detailed formulations are used to capture ecophysiological processes such as hydraulic failure and/or carbon starvation, which then are related to mortality risk (McDowell et al. 2013, 2016).

In general terms, the sources of mortality considered in DVMs typically include competition for light as a fundamental driver of forest dynamics under mesic conditions, and some models also emphasize the role of drought‐induced mortality under arid conditions. The limitations on growth induced by low light availability, drought, and other drivers (such as low nutrient availability) are typically used to drive a stress‐related mortality component, thus leading to an enhanced mortality probability compared to the background mortality.

It may be intuitive to assume that the sensitivity of DVMs to changes in the mortality formulation follows this categorization, that is, that replacing a mechanistic mortality function by an empirical one (e.g., as done in ForClim) is a larger difference than replacing a theoretical one with another theoretical one (e.g., as done in 4C). However, in the absence of simulation evidence, no quantitative statements are possible.

The forest structure and dynamics simulated with theoretical mortality formulations have been shown to be highly sensitivity to the exact assumptions that are being made (cf. Bugmann 2001: Fig. 14). The fact that it is impossible to determine a priori which theoretical formulation may be right has fueled the search for empirically based mortality formulations, based on either tree‐ring or forest inventory data. However, some studies (Bircher et al. 2015) suggest that also different empirically based formulations can lead to widely different forest trajectories in long‐term simulations.

The observation that model assumptions are subject to uncertainty is neither new, nor unique to the description of mortality. The topic of uncertainty and its quantification have attracted increasing attention in recent years (Cramer et al. 2001), both due to the realization that quantitative uncertainty estimates are key for sound management recommendations, and due to the increasing computing power, which makes the systematic exploration of uncertainty possible for larger models. However, while many studies have recently started to quantify parametric uncertainty (Hartig et al. 2012), and some results exist on the contributions of different model sectors to overall parametric uncertainty (Augustynczik et al. 2017), the quantification of structural uncertainty is less advanced. Most studies on this topic are model comparisons and multi‐model projections (Warszawski et al. 2014), which are helpful to explore differences between models regarding the effective variability in outputs due to structural differences. However, the fact that most models differ in most processes makes it excessively hard to track the model sectors that contribute most to output uncertainty.

To date, the investigation of the sensitivity of dynamic forest models to the formulation of mortality is restricted to a fairly small number of case studies (Bircher et al. 2015). Thus, a comprehensive understanding is lacking how the wide variety of mortality formulations in DVMs influence projections of future forest dynamics. Particularly, it is unclear how large the uncertainty induced by different mortality functions is relative to the magnitude of simulated forest changes that are induced by climate change. In the present paper, we seek to evaluate model sensitivity to different mortality formulations on a broad basis, jointly analyzing 15 models of forest dynamics that operate at the stand, landscape, or global scale.

Specifically, the objectives of this study were (1) to quantify the uncertainty in climate change projections that are due to differences in mortality formulations, using a wide range of forest models and an even wider range of mortality formulations; (2) to elucidate the reasons for differences in sensitivity (model type, model structure); and (3) to compare forest model sensitivity to mortality formulations with model sensitivity to climate change (i.e., the magnitude of climate change; IPCC 2013).

## Materials and Methods

### Dynamic vegetation models from the stand to the global scale

Fifteen models participated in the comparison (Tables 1 and 2).

**Table 1:** Overview of the 15 dynamic vegetation models employed in this study

Stand‐scale		Landscape‐scale		Global‐scale	
Model	References	Model	References	Model	References
4C	Reyer et al. (2014), Lasch‐Born et al. (2015)	iLand	Seidl et al. (2012)	CARAIB	Warnant et al. (1994), Dury et al. (2011)
ForClim	Bugmann (1996), Bircher et al. (2015)	LandClim	Schumacher et al. (2006), Temperli et al. (2013b)	ED(X)	Moorcroft et al. (2001)
FORMIND	Bohn et al. (2014), Fischer et al. (2016)	LANDIS‐II	Scheller and Mladenoff (2004)	LPJ‐GUESS	Smith et al. (2014), parameterized for species by Hickler et al. (2012)
FVS	Wykoff et al. (1982)			LPJmL	Bondeau et al. (2007), Schaphoff et al. (2013)
GOTILWA+	Nadal‐Sala et al. (2017)				
PICUS	Lexer and Hönninger (2001), Irauschek et al. (2017)				
SIBYLA	Fabrika (2005)				
xComp	Mette (2014)				

Notes

Each model, the simulation studies, and results are described in detail in Appendix S1.

**Table 2:** Overview of the 15 dynamic vegetation models (DVMs) participating in the comparison exercise regarding model type and mortality formulations

Name	DVM type^a^	Standard mortality (1a,b)	Alternative mortality (11, 12,…)
4C	P	1a) Intrinsic—Weibull (increase with age)	11a) Intrinsic—Weibull (increase with age)
		1b) Stress—foliage growth	11b) Stress—NPP
ForClim	S	1a) Background—const (based on max. age)	*11) NFI‐derived, randNr*
		1b) Stress—min. abs/min. rel. dInc	*12) as 11) + bckgrnd*
			*13) Tree‐ring derived, randNr*
			*14) as 13) + bckgrnd*
FORMIND	P	1a) Base mortality of 2% per yr	11a) Base mortality of 2% per yr
		1b) NPP < 0 = > immediate death	11b) NPP < 0 = > higher probability
FVS	E	*1) SDI‐based (Reineke)*	11) JABOWA min abs inc
GOTILWA+	P	1a) Carbon starvation	*11) Yoda's law (empirical)*
		1b) Reduction of sapwood functionality	
PICUS	P	1a) Background—max. age	*11) PROGNAUS empirical = ƒ(dbh, crown ratio, BAL)*
		1b) Stress—min. abs/min. rel. dInc	
SIBYLA	E	*1a) Empirical = ƒ(dbh, i*_*g*_ *, h, SI)*	*11) PROGNAUS empirical = ƒ(dbh, crown ratio, BAL)*
		*1b) Max. stand density (G*_*max*_ *)*	
xComp	E	*1) Empirical = ƒ(dbh, SDI, SI) based on Reineke's rule (using optimization)*	11a) Empirical as 1), plus
			11b) ‘Height‐antagonistic function’ time‐variable SI: if top height > max. height (SI) => death
iLand	P	1a) Background—max. age	11a) Background—max. age
		1b) Stress—neg. C balance (no delay for enhanced probability)	11b) Stress—minimum abs. dInc (5 yr delay)
LandClim	I	1a) Background—max. age	*11a) Background—max. age alone (NFI‐fitted)*
		1b) Growth‐dependent—dInc	*11b) Growth‐dependent—instantaneous (NFI‐fitted)*
LANDIS‐II	E	1) Age‐related mortality (sigmoidal increase w/age)	*11) Height‐related mortality*
CARAIB	P	1a) Constant mortality rate	11a) Growth efficiency (à la LPJ)
		1b) Stress‐induced (low T, low soil moisture)	11b) Stress‐induced (low T, low soil moisture)
LPJ‐GUESS	P	1a) Various drivers	11a) Various drivers
		1b) Stress—growth efficiency	11b) Stress—growth efficiency
LPJmL	P	1a) Background—max. age	11a) Background—max. age
		1b) stress—growth efficiency	*11b) Empirical, Pretzsch* et al.*, fitted to standard mortality*
ED(X)	P	1) Growth efficiency (as in LPJ)	11) Carbon starvation
			12) Hydraulic failure
			13) Phloem failure

Notes

Alternative formulations are numbered starting with 11, to distinguish them from standard formulations. Numbers with the denomination “a” and “b” refer to mortality formulations that are combined within a given DVM. Cells with italic font indicate empirically based mortality formulations that in some models are part of the standard setup, in others they are part of the alternative setup only. For more details on the individual models, cf. Appendix S1. NPP, net primary productivity; dInc, diameter increment; SDI, Stand Density Index; SI, Site Index; BAL, Basal Area of Larger trees; dbh, diameter at breast height; *i*_g_, basal area increment; *h*, tree height; *G*_max_, maximum stand basal area; NFI, National Forest Inventory.

a E is empirical (based on relationships derived, e.g., from forest inventory data); S is standard (similar to JABOWA, Botkin et al. 1972); P is physiological (based on physiological considerations such as photosynthesis, respiration, mechanistic allocation of carbon to plant organs, etc.).

At the stand scale, this included three models whose formulations were derived using statistical analyses based on inventory data (so‐called “empirical” models: FVS, SIBYLA, xComp); one model (ForClim) that closely adheres to the original approach underlying forest gap models (ForClim; cf.Botkin et al. 1972); three models that, to an increasing extent, contain elements that reflect plant ecophysiology (FORMIND, PICUS, and 4C); and one model that is highly physiological throughout its formulations (GOTILWA+).

At the landscape scale, only three models participated, partly reflecting the fact that there are far fewer such models in the literature than stand‐scale forest models (cf. Bugmann 2001, He 2008). The three models again represent a gradient of increasing complexity and level of process‐based detail, starting with LANDIS‐II Biomass Succession (hereafter simply “LANDIS‐II”; cf. Appendix S1: Section 10), which features many simple, empirically based elements, across LandClim, which reflects a higher level of detail, for example, regarding the representation of crown architecture, to iLand, a multi‐scale model that scales individual tree processes to the landscape level. Here, the landscape models were run for individual sites, thus essentially ignoring any landscape‐scale processes (such as the occurrence and spread of disturbances, or seed dispersal). This was done to enable comparison with the sensitivity of the stand‐scale models; local‐scale regeneration processes were of course maintained in the simulations.

At the continental to global scale, virtually all DVMs that are currently in use are highly process‐based (cf. Piao et al. 2013), and this is reflected in our selection. These models can also be used at the stand scale, but their formulations are typically tailored toward large‐scale application. Thus, subsequently they will be referred to as “global” models. Here, two variants of the LPJ model (LPJ‐GUESS and LPJmL) were used, as well as the CARAIB model and the ED(X) variant of the global model ED. All global DVMs except for LPJmL were run for individual sites, so as to enable comparisons with the behavior of the other models. Applying continental to global DVMs at the site scale is common practice for model evaluation, and straightforward as global DVMs increasingly include site‐scale processes that are not fundamentally different from those used in stand‐scale models (Hickler et al. 2012). However, a direct comparison of the CARAIB and ED(X) results with those of the other models was not possible because of fundamentally different output variables (e.g., biomass and carbon content).

A more detailed description of all the models and the individual simulations is provided as Appendix S1. Below, an overview of the technicalities of the simulations across all models is provided (cf. Tables 1, 2 and 3).

**Table 3:** Overview of the 15 dynamic vegetation models participating in the comparison exercise regarding sites of application, baseline climate data, and climate change scenarios

Name	Biome and site(s)	Baseline vs. clim. change period	Climate scenarios (summer ΔT; summer fractP)
4C	Temperate, Brandenburg/Germany (Peitz)	1981–2010 2071–2100	REMO RCP2.6 (+0.6; 0.94) REMO RCP8.5 (+2.0; 1.06) RCA RCP2.6 (+0.9; 0.97) RCA RCP8.5 (3.7; 0.97)
ForClim	Temperate, Switzerland (Sigriswil [historical only] and Scatlè)	1981–2010 2071–2100	RCP3PD (+2.2; 0.85) A1B (+4.8; 0.7)
FORMIND	Temperate, Brandenburg/Germany (Peitz)	1981–2010 2070–2099	RCP2.6 (+1.9; 1.07) RCP8.5 (+5.3; 0.95)
FVS	Mediterranean, California (Modoc National Forest)	1960–1990 2090	RCP4.5 (+3.9; 0.89) RCP8.5 (+7.4; 0.78)
GOTILWA+	Temperate, Brandenburg/Germany (Peitz)	1971–2010	RCP2.6 (+1.62; 0.81) RCP8.5 (+5.0; 0.69)
PICUS	Temperate, Austria (three sites in northern front range of Alps)	1961–1990 2080–2100	A1B (low = 500 m a.s.l.: +3.9; 0.81) A1B (med = 900 m: +3.6; 0.93) A1B (high = 1400 m: +3.8; 0.93)
SIBYLA	Temperate, Slovakia (Predmier)	1961–1990 2100	IMAGE‐RCP3PD(2.6) (+2.6; 0.93) MESSAGE‐RCP8.5 (+7.4; 0.90)
xComp	Temperate, Bavaria/Germany (NFI sample plot with Norway spruce)	1971–2000 2080–2100	mg4.5 (+1.2; 1) no4.5 (+3; 0.93) gf4.5 (+4.3; 0.89) mg8.5 (+2; 1.07) no8.5 (+3.3; 0.91) gf8.5 (+5.9; 0.86)
iLand	Temperate, Austria (Eibiswald, Karlstift, Ottenstein)	1981–2010 2080–2099	Eibiswald (other sites very similar): CNRM‐RM4/ARPEGE (+4.9; 0.82) CNRM‐RM4/MPI‐REMO (+3.9; 0.74) ICTP‐RegCM3/ECHAM5 (+3.5; 1.16)
LandClim	Temperate, Rhône‐Alps region/France (112 NFI plots)	1981–2010 2100	GCM MPI‐ESM‐LR RCP 2.6 (+5.2; 0.83) GCM MPI‐ESM‐LR RCP 8.5 (+9.5; 0.52)
LANDIS‐II	Temperate, Czech Republic (Sumava)	N/A	20% growth increase (climate‐ and CO_2_‐induced)
CARAIB	Temperate and boreal, Europe (6 FLUXNET sites)	1981–2000 2081–2100	GCM GFDL‐ESM2M (averaged over six sites): RCP 2.6 (+1.1; 1.01) RCP 4.5 (+1.6; 0.97) RCP 6.0 (+2.5; 0.91) RCP 8.5 (+3.7; 0.88)
LPJ‐GUESS	Temperate and boreal, Europe (5 FLUXNET sites)	1981–2010 2070–2099	5 ISI‐MIP fast‐track GCMs: RCP 2.6 (averaged) (+2; 1.07) RCP 8.5 (averaged) (+5.5; 1.03)
LPJmL	Tropical, Amazonia (117 plots, cf. Brienen et al. 2015)	1981–2010 2081–2100	GCM MPI‐ESM‐LR: RCP 4.5 (2.3; 0.88) RCP 8.5 (5.3; 0.80)
ED(X)	Arid temperate, USA (Sevilleta rainshelter experiment)	2007–2011 2071–2100	RCP8.5 (+4; −34 mm)

Notes

fractP indicates the fractional change of precipitation (e.g., 0.94 implies a 6% decrease of precipitation by the end of the climate change period). For more details on the individual models and their setup in the simulations, cf. Appendix S1.

### Simulation protocol

Model comparison exercises have a long history (Bugmann et al. 1996, Cramer et al. 2001, Morales et al. 2005, Piao et al. 2013) and are potentially rewarding for disentangling the response of various models, for example, to common driving forces, using a common set of sites, climatic conditions, and other boundary conditions. However, they require substantial streamlining of data and protocols, such that many modeling teams do not participate because of the sheer investment required.

For the present study, a different approach was used to foster wide participation by a diversity of modeling teams, resulting in a large number of modeling teams participating and contributing models that were developed for different spatial scales. Each team had a specific DVM at its disposal and was running simulations with their standard model for at least one site for which the data required to run the model were readily available. This is subsequently termed the standard set of simulations, which included runs under both the historical climate as well as a set of climate change scenarios (for details, see below). Then, the standard formulation of mortality was replaced by an alternative formulation from the literature that is appropriate for the resolution of the model (e.g., stand‐level vs. individual‐level rates, predictor variables available for alternative formulations). The choice of this alternative mortality formulation was at the discretion of the modeling teams.

Each modeling team defined at least one site or a set of sites for which the model comparison was run, including all necessary driver information (climate, soils, etc.). Then, the standard model was run for the(se) site(s) under current climate, and for six models, the standard and alternative functions were tested against measured historical time series data to assess the importance of the choice of the mortality model when running relatively short‐term (i.e., several decades) simulations under current climate.

This was followed by the definition of a set of climate change scenarios for the respective site(s) that cover a wide range of climatic conditions, preferably including a moderate (2° target, i.e., RCP2.6) as well as a severe scenario (i.e., RCP8.5). For the analysis, climate change scenarios were classified as moderate if their annual average temperature change for the end of the 21st century was below +3°C; otherwise they were classified as severe (Table 3).

Subsequently, the standard models were run from the current state (as given by the latest inventory, or based on a spin‐up run) for 200 yr into the future under these climate change scenarios, assuming a constant climate after the year 2100 (due to the lack of climate information beyond that point, but to still be able to take into account that climate‐induced forest dynamics may take much longer than a century to unfold).

These standard simulations were then complemented by simulations using an alternative mortality formulation that was identified from the literature and implemented in the model. For some models, several alternatives (or combinations thereof) were considered and are described in Appendix S1, but for the comparison, only a subset of these alternative formulations (or a combination of alternatives) was used for the sake of simplicity (Table 2).

With the alternative model, the same simulations as described above were run under current climate as well as under the scenarios of climate change.

Our approach may be criticized because it lacks rigor compared to the standard model comparison exercises (Cramer et al. 2001, Morales et al. 2005, Piao et al. 2013). However, our approach has two distinct advantages: First, the models are run in their comfort zone, and hence, we rule out artifact responses that one may see in other model comparisons because models are run under conditions that they were never really parameterized for. Second, the way we did the study represents the reality of model applications, that is, a large number of different models are applied to different sites across the globe to estimate climate change impacts. We for the first time evaluate how sensitive such real‐world model applications are to different mortality formulations. Hence, our goal was not primarily a formal model comparison exercise, but rather an exercise highlighting how sensitive the DVMs currently applied in impact assessments are to mortality formulations.

### Model comparison

Simulation results were reported as total basal area, stem number, diameter distributions as well as species composition for those five models that simulated multi‐species forests (i.e., FVS, iLand, LandClim, LPJ‐GUESS, and PICUS), the composition in terms of Plant Functional Types (LPJmL), or species‐specific biomass proportions (CARAIB) over time. Stand‐level basal area and stem numbers were the major variables upon which we focused in the subsequent comparison.

A sensitivity index sens was developed to compare simulation results under different mortality formulations or different climate change scenarios within each model at each site, as follows:

(1) $$\text{sens}=\left|\frac{y_{\mathrm{alt}}\left(t_{\mathrm{end}}\right)}{y_{\mathrm{std}}\left(t_{\mathrm{end}}\right)}\right|\cdot \frac{100}{t_{\mathrm{end}}-t_{\mathrm{start}}}$$

where *y* is the target variable that is considered (basal area or stem number, both at the stand scale), std and alt are subscripts denoting the standard or alternative formulation (or the current and a future climate, respectively), and *t*_start_ and *t*_end_ denote the time of the beginning and end of the evaluation period (in yr). In the case where *y*_alt_ was smaller than *y*_std_, the two variables were swapped. Thus, in essence Eq. 1 reflects the difference between two simulation runs expressed as the *absolute fractional* change *per century*.

All calculations and graphics were made using the statistical software *R* v3.3.2 (R Core Team 2017).

### Behavioral patterns expected to arise from the model pool

The 15 models that were used in this exercise (Table 1) featured widely different pairs of standard and alternative mortality algorithms (Table 2), thus leading to strongly different expectations regarding model sensitivity to these variations, as explained below.

First, some models were set up to employ mortality formulations that were closely related to one another. For example, in the FORMIND model the standard formulation uses a 2% per yr background mortality combined with a stress‐related component that kills trees instantly whenever their annual net primary productivity (NPP) is below zero. In the alternative formulation, the background mortality formulation was maintained, and the stress‐related component was formulated such that when NPP < 0, tree survival probability was reduced, rather than being set to zero (for details, cf. Appendix S1: Section 5). In such cases, one would expect low model sensitivity to the mortality formulation irrespective of the setup of the simulation, as the formulations are only slight variations of each other. Similar reasoning applies to five models: 4C, CARAIB, FORMIND, iLand, and LPJ‐GUESS (Table 2).

Second, in some cases the alternative formulations were calibrated (statistically or by hand) to yield comparable results under current climatic conditions as the standard formulation. In these cases, one would expect very low sensitivity under current climatic conditions and also low sensitivity under future climatic conditions as long as the range of the calibration data is not exceeded. This applies to LandClim, LANDIS‐II, and LPJmL (Table 2).

Third, the majority of the DVMs were set up to feature conceptually and structurally different alternative mortality formulations. For example, in the standard GOTILWA+ model, the mechanisms of carbon starvation and hydraulic failure are incorporated as drivers of mortality. For the alternative formulation, Yoda's (1963) self‐thinning law was used, which in contrast to the standard formulation does not operate at the individual tree level and is not based on any physiological mechanisms. Thus, one may expect widely different simulation trajectories from the two approaches potentially already under the current climate, but certainly under a changing climate. Similar reasoning applies to eight models: ED(X), ForClim, FVS, GOTILWA+, PICUS, SIBYLA, and xComp (Table 2).

## Results

### Sensitivity to mortality formulations under current climate

**Figure 1:**
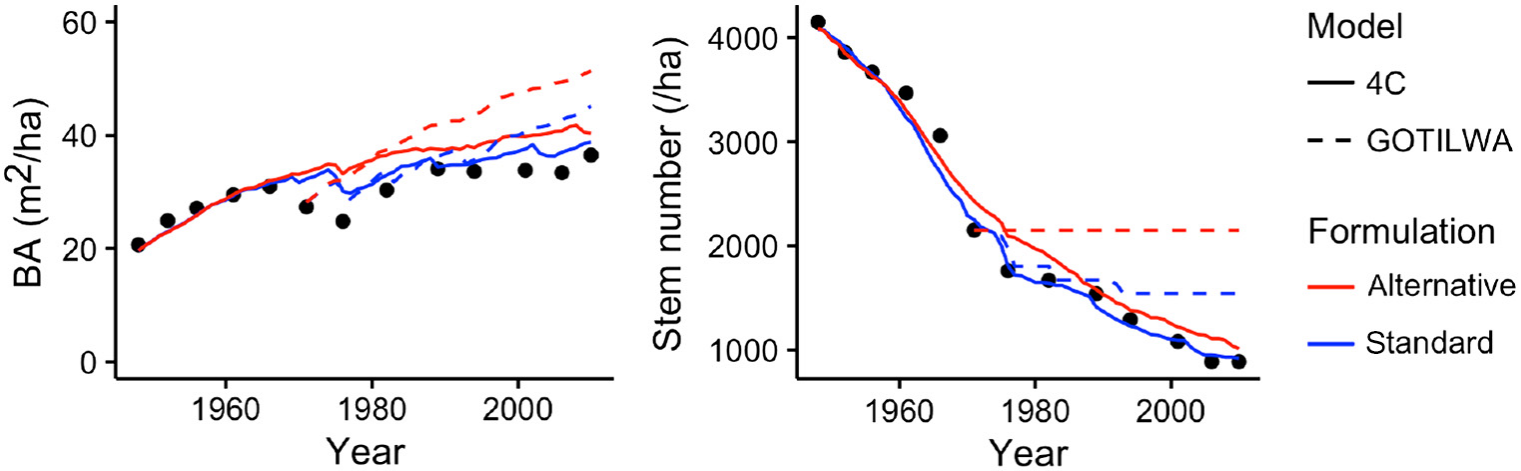
Examples of the comparison of simulated trajectories against empirical data (black dots), with the cases of the 4C and the GOTILWA+ model, both being run for the ISI‐MIP site Peitz in Brandenburg, Germany.

The simulation results from six models were compared against long‐term time series data from forest inventories (e.g., the site Peitz in Brandenburg, Germany, featuring stand development data over 60 yr), including 4C, ForClim, FORMIND, GOTILWA+, iLand, and LPJ‐GUESS. These tests typically showed that each of the model formulations is able to represent the observed mortality at decadal time scales under current climatic conditions (cf. Fig. 1). An important exception was GOTILWA+, which featured a strong decrease in tree numbers with the standard (physiological) mortality algorithm that matches the empirical data rather well, but virtually no mortality occurred during the second half of the test period when employing the alternative (self‐thinning) algorithm (Fig. 1).

CARAIB, ED(X), and SIBYLA featured a rather short‐term evaluation period only (~15 yr), thus making it unlikely to detect differences as a function of different mortality algorithms. Yet, SIBYLA showed very strong sensitivity: In the very short‐term perspective (7 yr), the alternative formulation (Monserud and Sterba 1999) led to unrealistic mortality rates. However, when taking the whole evaluation period (i.e., 14 yr) into account, the simulated mortality rates were approaching the measured data over this period.

Several models including FVS, LANDIS‐II, LPJmL, and xComp were checked for the realism of model behavior under current climatic conditions, albeit not as stringent long‐term tests, thus not providing conclusive insights on model sensitivity under current climatic conditions.

### Sensitivity to mortality formulations vs. sensitivity to climate change

#### Stand‐scale models

**Figure 2:**
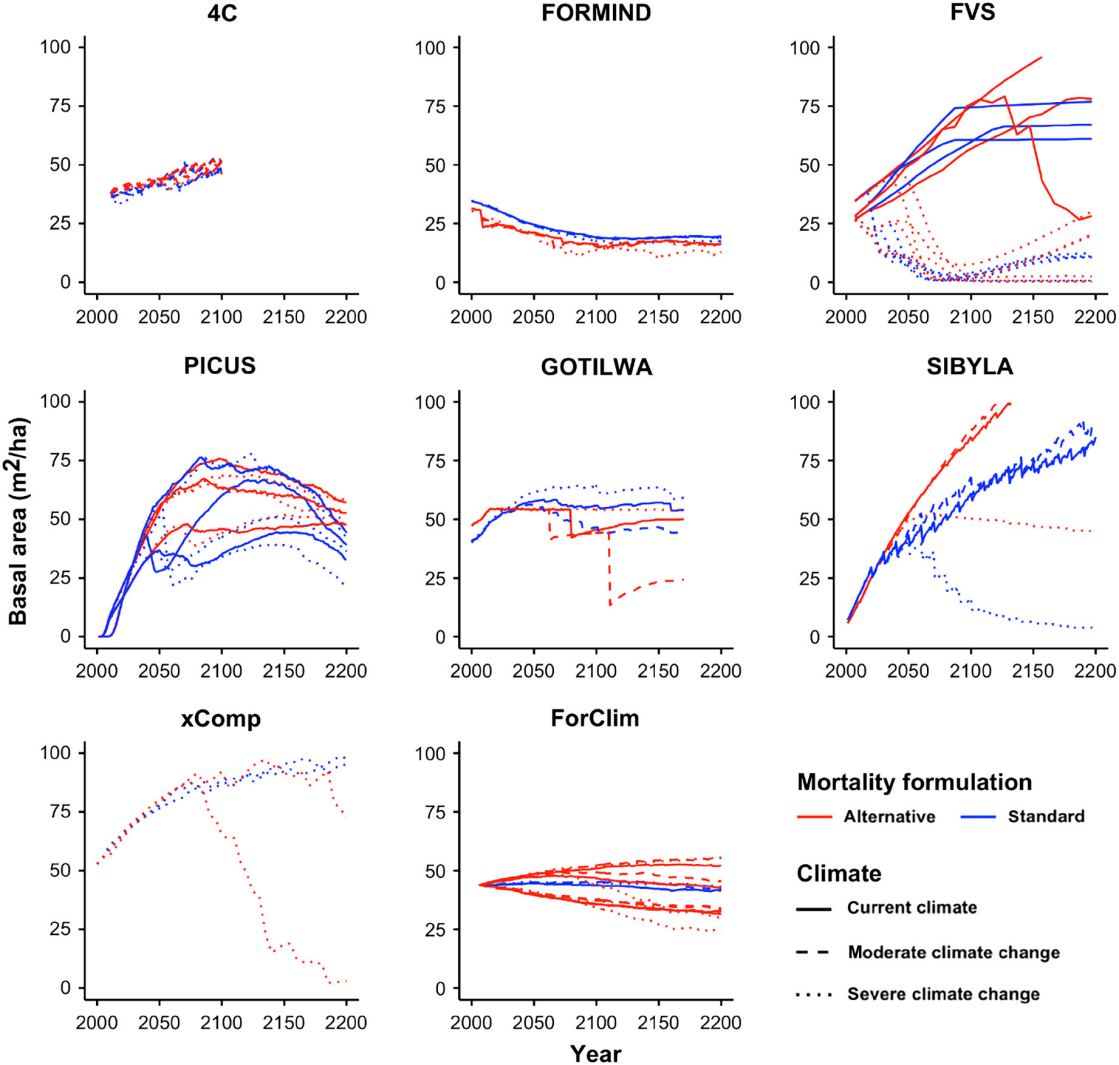
Simulation of basal area (m^2^/ha) by the eight stand‐scale models at the respective sites (cf. Table 3) for 200 yr into the future (with the exception of 4C, for which the simulation was ended in the year 2100 in all cases). Note that in the case of PICUS, three sites were simulated (cf. Table 3). Moderate climate change: change of annual mean temperature <3°C (cf. *Methods*).

**Figure 3:**
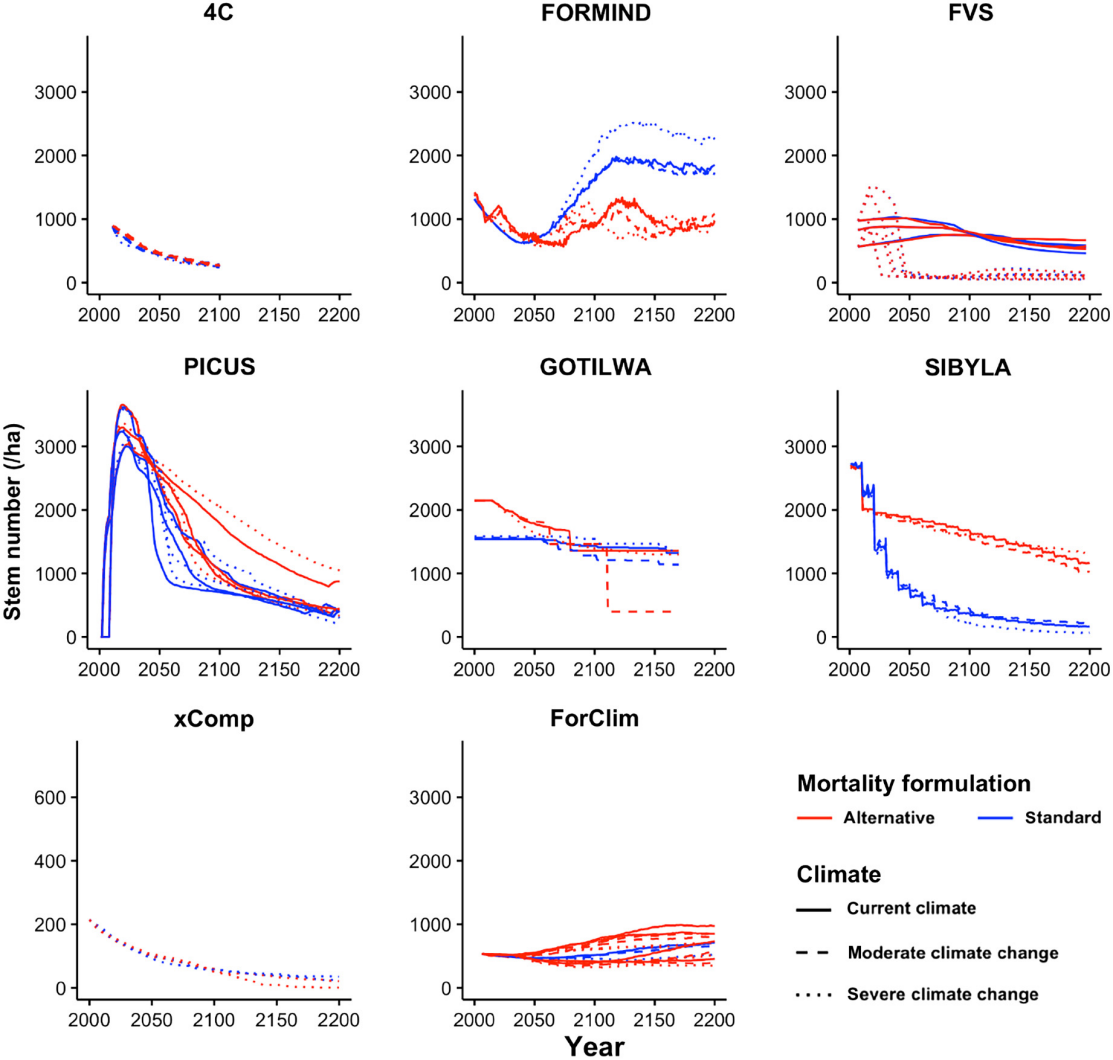
Same as Fig. 2, except that stem numbers (ha^−1^) are shown. Note the scaling of the *y*‐axis for xComp, which differs from that used for the other models.

Simulated trajectories of basal area (Fig. 2) and stem numbers (Fig. 3) suggested that different mortality formulations often led to low sensitivity of model outputs over the first ~100 yr of the simulation (e.g., 4C, FORMIND, GOTILWA+, xComp). However, most models featured strongly different trajectories of these two variables over longer time scales, with the differences developing either smoothly (e.g., ForClim), or in a rather abrupt fashion (e.g., FVS, GOTILWA+, SIBYLA).

It is noteworthy that for some models the trajectories of basal area (Fig. 2) were rather similar in response to different mortality formulations, whereas simulated tree numbers (Fig. 3) differed substantially in the long term (e.g., FORMIND), but the opposite pattern was also found in one case (i.e., xComp).

**Figure 4:**
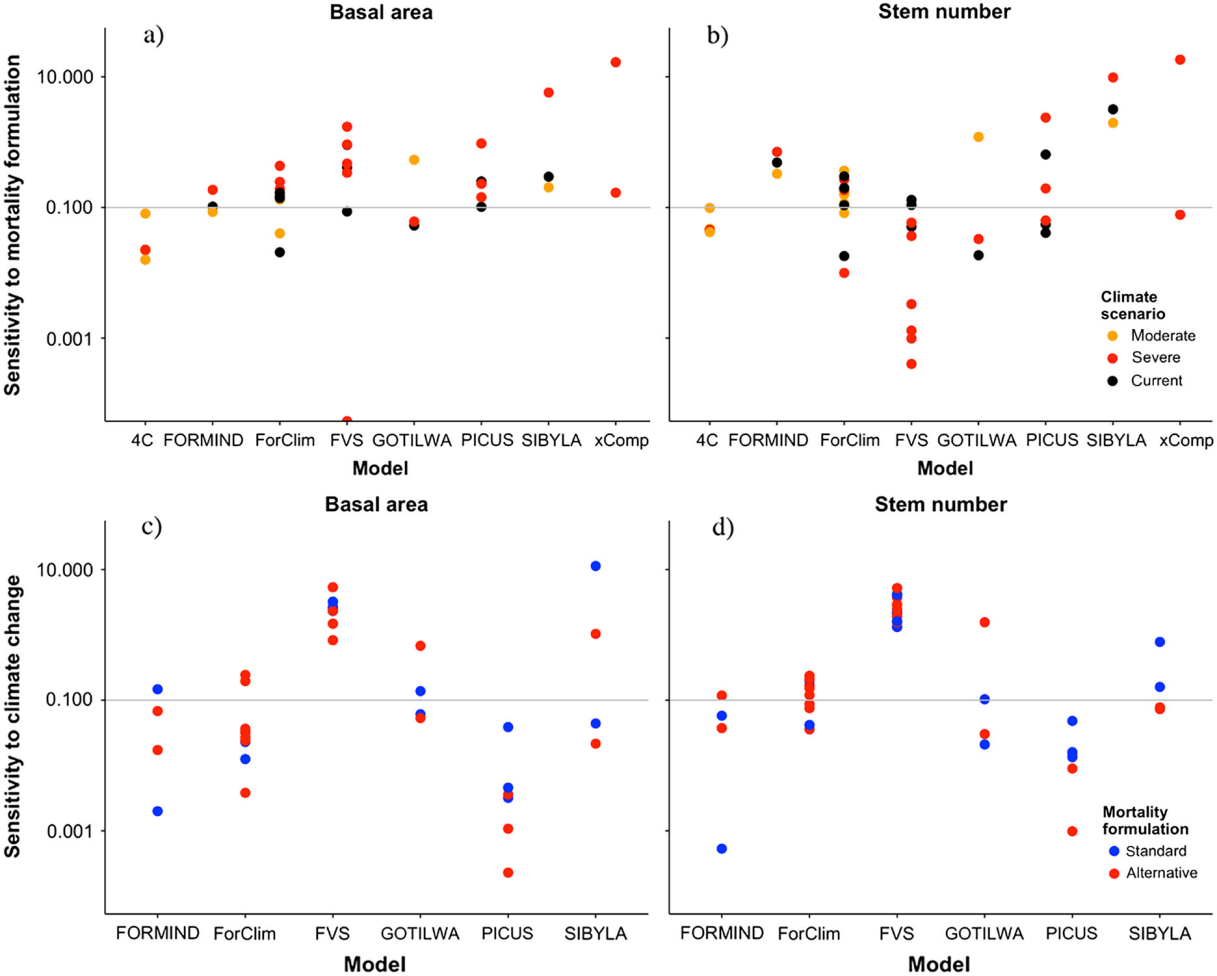
Sensitivity index for basal area (left column, panels a and c) and stem numbers (right column, panels b and d) for the eight stand‐scale models, extending over 200 simulation years (i.e., for most models the period until the year 2200, or 200 yr after the beginning of the climate change scenario). Note the logarithmic scale of the *y*‐axis. The horizontal gray line indicates a sensitivity index of 0.1. Top row (a,b): sensitivity to mortality formulations under different scenarios of climate change. Bottom row (c, d): sensitivity to climate change using different mortality formulations. Note that the xComp and 4C models could not be evaluated for their sensitivity to climate change, as no simulation under current climate into the future was performed. Also. 4C was run until 2100 only, that is, its sensitivity index refers to 100 yr, rather than 200 yr as for the other models (cf. Appendix S1).

The evaluation of the sensitivity index (Eq. 1) for the stand‐scale models (Fig. 4) yielded the following distinct patterns:

First, some combinations of forest models and mortality formulations tended to be more sensitive than others. PICUS, SIBYLA, and xComp uniformly featured sensitivity indices for basal area (Fig. 4a) that were higher than 0.1 (i.e., more than a 10% difference in basal area over a century depending on the choice of the mortality formulation), with maxima up to a 17‐fold difference (i.e., sensitivity index of ≈17), whereas the model 4C had values uniformly below 0.1. The other four models ranged in between. For stem numbers (Fig. 4b), which are known to be much more variable than basal area (cf. Pretzsch 2009), the pattern was less clear except that for FVS, often low sensitivity was found.

Second, the sensitivity indices of the mortality formulations for basal area under the current climate (black dots in Fig. 4a) tended to be similar as for moderate climate change (typically, RCP2.6; orange dots), whereas the sensitivity indices for severe climate change (RCP8.5; red dots) were typically much higher than under both current climate and moderate climate change (Table 4). Thus, the sensitivity to the formulation of mortality becomes stronger as the climate is shifting away from current conditions, largely independent of the forest model that is considered.

**Table 4:** Averages (μ) and median (med) values of the sensitivity indices for basal area (BA) and stem numbers (no.) for all stand‐scale simulations under current climate, moderate, and severe climate change, respectively (cf. Fig. 4a, b), showing a clear tendency for higher sensitivity to the mortality formulation with increasing degree of climate change

Climate	μ (BA)	med (BA)	μ (no.)	med (no.)
Current	0.23	0.14	0.41	0.11
Moderate change	0.14	0.09	0.27	0.11
Severe change	0.87	0.18	1.72	0.07

Note

A statistical analysis of the distributions is not possible because the data within each group are not independent.

Third, sensitivity of simulated basal area and stem numbers to the magnitude of climate change (Fig. 4c, d) tended to be lower than sensitivity to the mortality formulation (Fig. 4a, b), as the points were clustered between 0.01 and 0.1 regarding climate sensitivity, but between 0.1 and 1.0 regarding mortality sensitivity. However, there was no clear pattern for the standard vs. the alternative mortality formulation regarding climate sensitivity (Fig. 4c, d). Also, there were strong model‐specific exceptions (e.g., FVS, whose climate sensitivity is much larger than its sensitivity to alternative mortality formulations).

#### Landscape‐scale models

**Figure 5:**
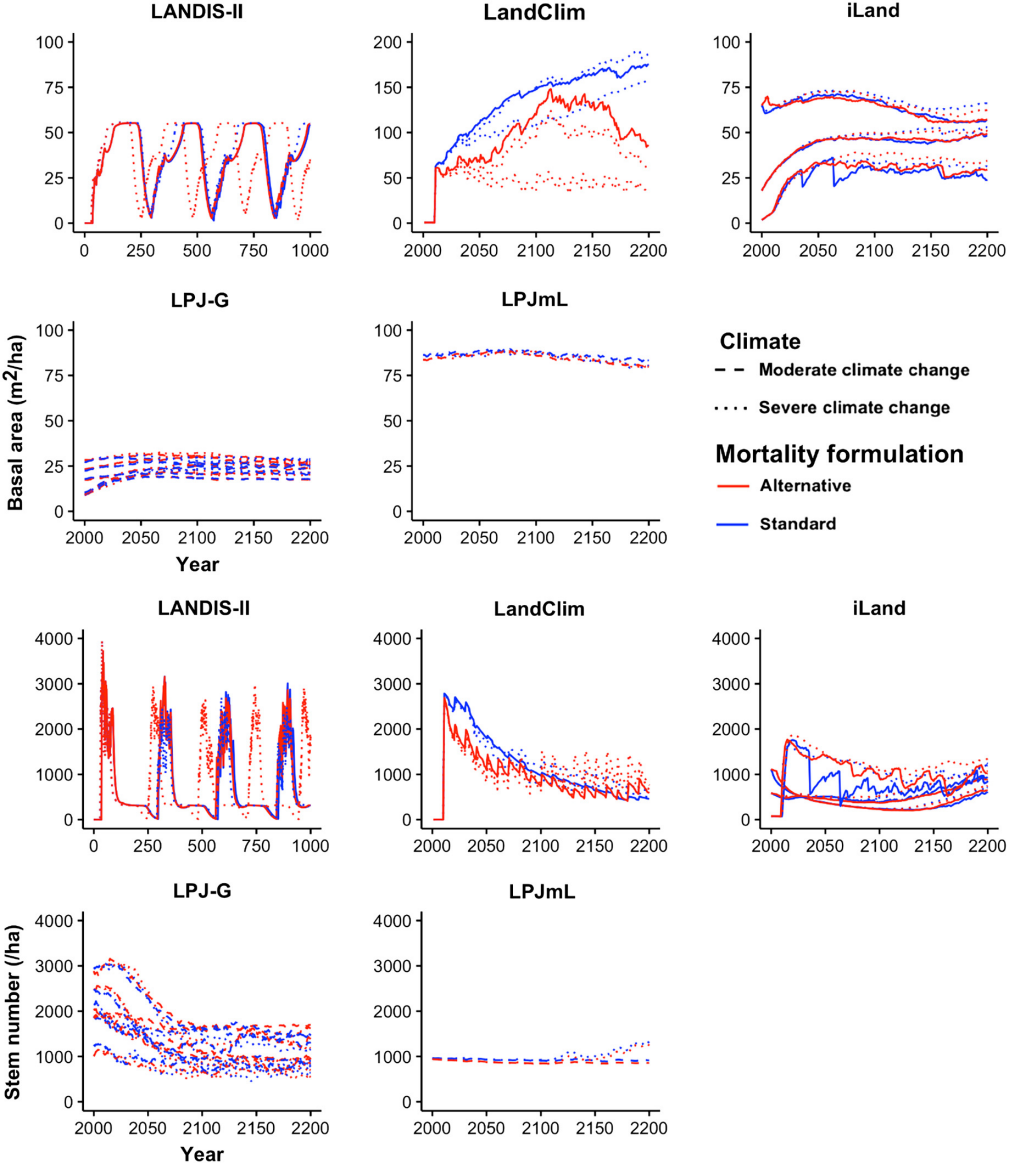
Basal area (top) and stem numbers (bottom) simulated by the three landscape‐scale models and the global models LPJ‐GUESS and LPJmL (which were run at the stand scale for this analysis) at the respective sites (cf. Table 3) for at least 200 yr into the future. Note the different time scale of the simulation shown for LANDIS‐II, as the results from that model over the first 200 yr were nearly identical irrespective of the mortality formulation; note also the different *y*‐axis scale in the basal area panel of LandClim (top row).

Among the three landscape‐scale models, LandClim provided widely different trajectories of basal area depending on the mortality formulation (Fig. 5, top), but much lower variation of stem numbers (Fig. 5, bottom). iLand (shown in Fig. 5 for three sites) showed little sensitivity with respect to either basal area or stem numbers. LANDIS‐II featured virtually identical simulation results under both mortality formulations during the first two centuries of the simulation (i.e., in the early succession phase), and later primarily a phase shift was evident from the simulation results, depending more on the climate scenario than on the mortality formulation (Fig. 5).

**Figure 6:**
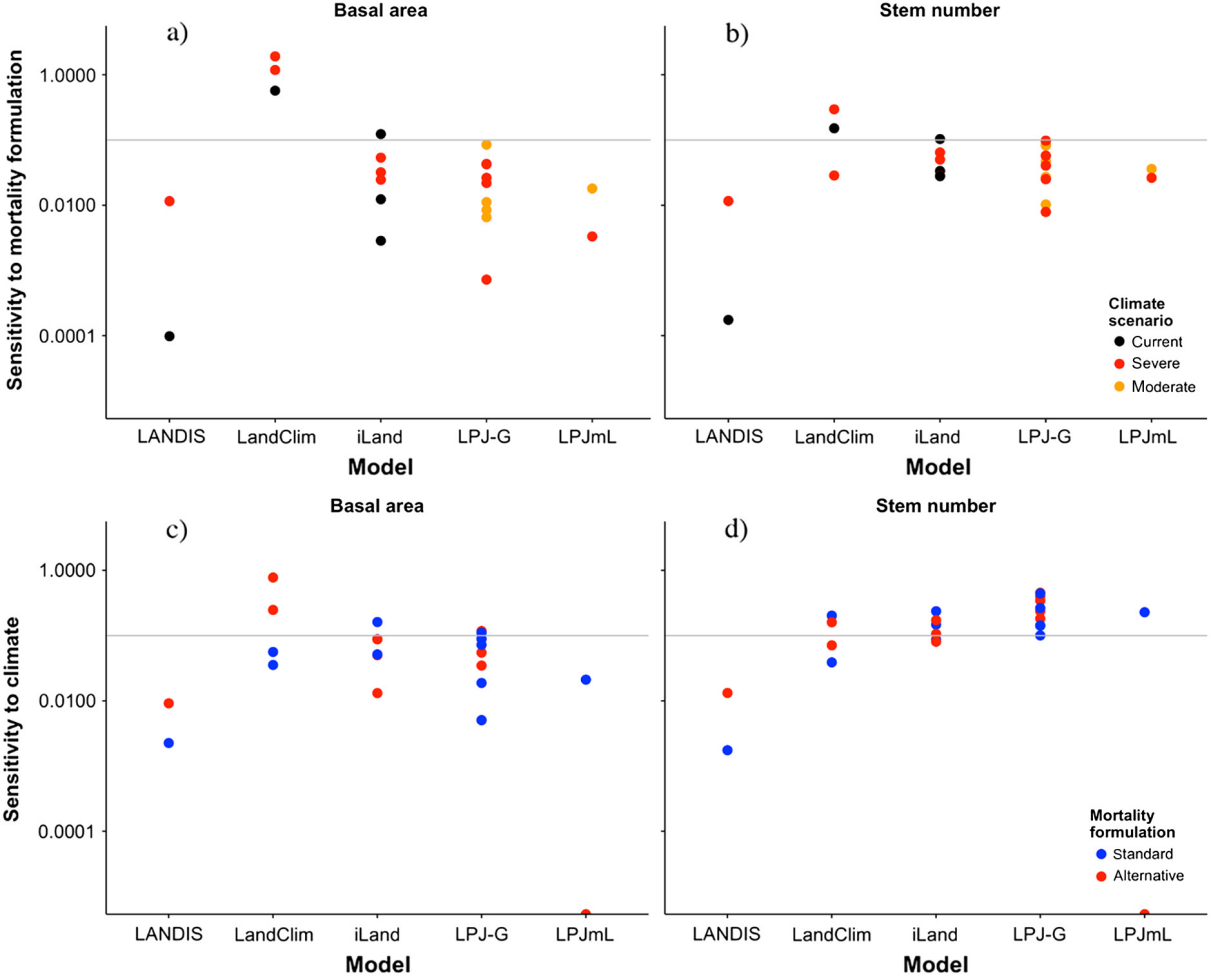
Sensitivity index for basal area (left) and stem numbers (right) for the three landscape‐scale models and the global models LPJ‐GUESS and LPJmL. The horizontal gray line indicates a sensitivity index of 0.1. For details, cf. caption of Fig. 4.

The three landscape‐scale models featured sensitivity indices regarding the choice of mortality formulation (Fig. 6a, b) that tended to be distinctly lower than those in the stand‐scale analysis (Fig. 4a, b), not often exceeding a value of 0.1. However, their sensitivity to climate change (Fig. 6c, d) tended to be higher than the sensitivity to the formulation of mortality, most pronounced for basal area, which again is a distinct difference compared to the stand‐scale results (Fig. 4c, d).

#### Global‐scale models

Three global DVMs were run for individual sites in our study, that is, CARAIB, ED(X), and LPJ‐GUESS. The latter essentially is a stand model based on the gap paradigm (Shugart 1984), but it is typically applied across larger areas on a grid. Therefore, its results are directly comparable to those of the models at the other scales, and they were thus analyzed accordingly (Figs. 5 and 6), showing relatively low variation of basal area and stem numbers depending on the choice of mortality formulation, and a larger sensitivity to the choice of the climate scenario than the mortality formulation, particularly regarding simulated stem numbers.

LPJmL was run for the entire Amazon region, but its results were aggregated to average values across the region (Figs. 5 and 6), showing very low sensitivity of basal area and stem numbers to the formulation of mortality. A closer inspection of the simulated spatial patterns (cf. Appendix S1: Section 12) suggests that this is mostly due to spatial averaging, that is, for subregions of the Amazon noticeable differences are evident, although they are not very large either.

CARAIB featured strongly different long‐term development of biomass carbon depending on the mortality formulation (results not shown here; cf. Appendix S1: Section 2), indicating a high sensitivity of this global model to the choice of mortality formulation.

Lastly, ED(X) was run for an experimental site in the southwestern United States, evaluating short‐term tree mortality simulations using the concepts of carbon starvation, hydraulic failure, phloem failure, or growth efficiency (McDowell et al. 2013). Also this model featured strong sensitivity to the assumptions about the drivers of mortality, that is, widely different mortality probabilities. In general, the carbon starvation mechanism predicted lowest mortality due to the CO_2_ fertilization effect; the hydraulic failure mechanism induced highest mortality under the RCP8.5 greenhouse emission scenario; and the phloem failure mechanism yielded a medium level of mortality (cf. Appendix S1: Section 3).

## Discussion

**Figure 7:**
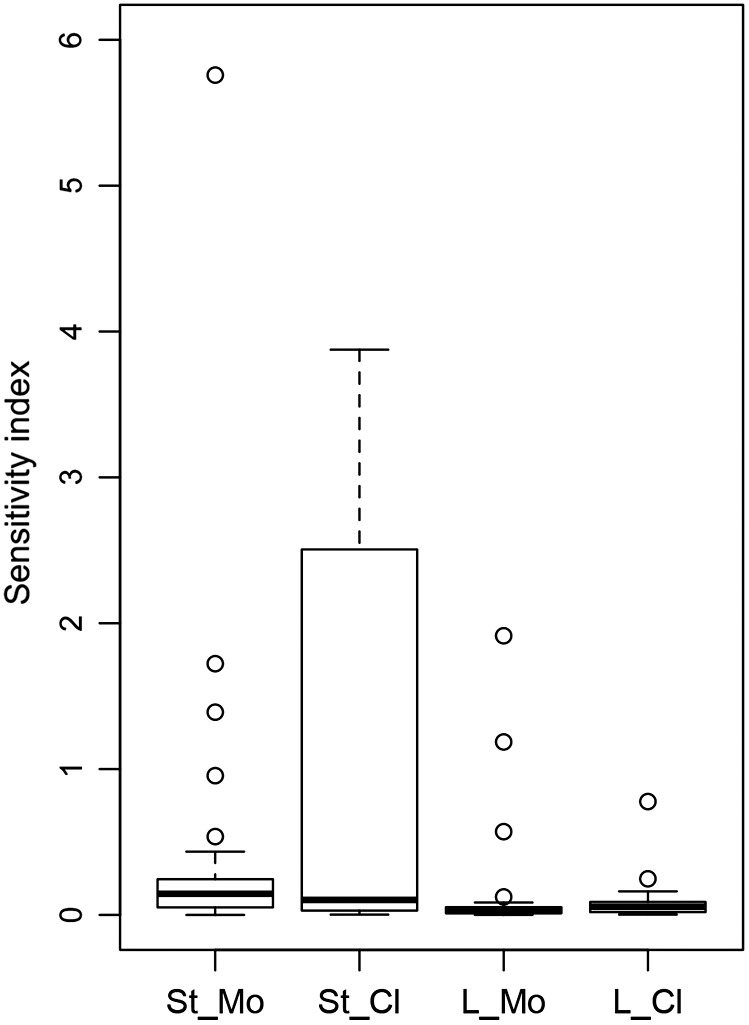
Box plots of the distribution of the sensitivity indices for basal area regarding model formulation (Mo) and climate (Cl) at the stand (St) and landscape (L) scales for 200‐yr simulations into the future. Four outliers in stand‐scale sensitivity with respect to mortality (St_Mo) and climate (St_Cl) are not shown (cf. Fig. 4a, c), as they represent situations where excessively low biomass values were obtained (two scenarios in FVS, and one each in xComp and SIBYLA; cf. Fig. 2).

The present study suggests that there is considerable sensitivity of stand‐scale DVMs to the formulation of mortality (Fig. 7), which tends to be larger than the sensitivity to different scenarios of climate change. For the three landscape‐scale DVMs examined here, sensitivity to both mortality modeling and climate change is lower, although the latter is relatively larger. Yet, this may not provide solid ground for generalizations because structurally rather similar mortality functions were used in the landscape‐scale models as standard and alternative approaches (unlike in many stand‐scale models; cf. Table 2). Furthermore, the low number of participating models (*n *=* *3) needs to be considered as well. Thus, further tests are required by including additional landscape‐scale models. For the global‐scale models, it is difficult to generalize beyond the few case studies that were examined here, and further studies are sorely needed.

### Behavioral patterns arising from the model pool

Model sensitivity to the formulation of mortality only partly followed the expectations that were based on DVM structure and driving variables. However, the deviations, as well as the patterns within the three groups of expected model behavior, yielded relevant insights.

Among the five DVMs that were expected to feature low sensitivity to the formulations of mortality due to strong structural similarity of the approaches that were applied, three actually showed low sensitivity: 4C, iLand, and LPJ‐GUESS. These models either employed a background mortality rate that is constant and combined it with somewhat different approaches for portraying stress‐induced mortality (e.g., NPP vs. investment into foliage growth in the case of 4C), or they used the same stress‐induced mortality and combined it with different formulations of the background mortality (e.g., age‐related vs. diameter‐related mortality in the case of LPJ‐GUESS). In this latter example, it is noteworthy that there are distinct advantages of using a diameter‐related mortality (cf. Manusch et al. 2012), for example, for reproducing the trade‐off between juvenile growth rate and lifespan (Bigler and Veblen 2009). Overall, in these three DVMs only (1) a part of the mortality formulations was altered, and (2) to a moderate extent, thus not surprisingly leading to low variations in the simulation results. Thus, these models resulted in robust simulation trajectories even when moderate changes in the mortality formulation were assumed.

The other two DVMs that were expected to feature low sensitivity based on the structural proximity of the standard and alternative mortality formulations, that is, FORMIND and CARAIB, featured surprisingly high sensitivity in spite of only slight mathematical differences in just one part of the formulation (Table 2). This was most pronounced for FORMIND, where the only difference was that in one approach trees are killed immediately (i.e., survival probability is zero) when their NPP falls below zero, whereas in the other approach their survival probability is reduced by a certain degree, proportional to the negative NPP level. Although technically this is just a difference in the value of a parameter, from an ecological point of view it constitutes an important difference: Certain death is qualitatively different from having a non‐zero survival probability.

Two DVMs were expected to feature low sensitivity to the mortality formulation because they were calibrated with the objective to yield similar results under current climate, that is, LandClim and LPJmL. This expectation was met fully for LPJmL. For LandClim, however, the highest sensitivity of all landscape‐scale models was found, even under current climate. This suggests that the calibration may have resulted in a model that matches well a rather small data set (i.e., a subset of the French National Forest Inventory), which represents just a short snapshot of forest dynamics in space and time, such that the model was used beyond its calibration range in our exercise. Overfitting of DVMs to short‐term data series was found to be a major problem in other studies as well (Mette et al. 2009, Bircher 2015, Hülsmann et al. 2018).

The other eight models were expected to feature higher sensitivity to the different mortality algorithms because they were based on widely different driving variables (such as a physiological approach vs. stand density, as in GOTILWA+) or had a distinctly different structure (theoretical vs. inventory‐based formulation, as in ForClim). This expectation was met with the exception of FVS in the case of stem numbers, which typically featured very low sensitivity. In FVS, climate change has a strong effect on the species’ growth, mortality, and regeneration rates at the study sites. Already 40 yr into the simulation, climate‐induced mortality calculated (empirically) by the model drove some species nearly to local extinction. A consequence of such (unrealistic) behavior was that the mortality estimates for the end of the simulations were quite similar to each other, under both RCP 4.5 and 8.5, regardless of the algorithm used, because they were calculated from a very low living biomass.

In at least some scenarios, many of the other seven models reached sensitivity values that reflected a difference in the target variables (Eq. 1) of at least 50% per century (for basal area, this was the case in 14% and for stem numbers in 13% of the simulation runs, respectively). This indicates vast differences in simulation results depending on the mortality formulation. Specifically, the collapse of basal area (and often also stem numbers) that occurred with just one of the mortality formulations in FVS, SIBYLA, xComp, and, to some extent, in GOTLIWA+ reflects the presence of non‐linear effects and thresholds in the mortality formulations. These thresholds may be exceeded in some, but not in all cases, thus yielding highly different simulation results with manifold differences in the target variables, that is, completely different trajectories of the DVM. Such divergence may arise when the original process descriptions are tailored to one another (e.g., applying physiological principles throughout), such that the modification of one single process description (e.g., replacement by a population‐based approach such as Yoda's self‐thinning law in GOTILWA+) may render the DVM inconsistent, leading to unwanted feedforward/feedback effects and, ultimately, incongruent projections of future dynamics.

Lastly, it is conspicuous that the highest sensitivity values regarding basal area and often also stem numbers were reached by models that feature an empirically based overall structure and at least one empirically based mortality formulation, that is, FVS, xComp, and SIBYLA. Although we cannot make firm statements about the appropriateness of mortality formulations under future no‐analog conditions (IPCC 2013), it is likely that some empirically based mortality formulations are exceeding their range of applicability in a future climate, whereas theoretically based approaches that try to reflect biological dependencies and ecological relationships may be less likely to yield erroneous results when applied under future climatic conditions.

### Sensitivity to mortality formulations under current climate

It is little surprising that DVMs that featured low sensitivity to the choice of mortality formulation under scenarios of climate change, such as 4C, were also little sensitive to mortality under the current climate (Fig. 1). However, the cases of FORMIND, ForClim, and LandClim demonstrate that the absence of evidence of sensitivity under current climate does not constitute evidence for the absence of such sensitivity under climate change. Thus, neither the validation of simulated DVM trajectories against long‐term inventory data (FORMIND, ForClim) nor the calibration of model behavior against (shorter) inventory data (LandClim) are a guarantee for reliable model behavior under future conditions.

This situation reflects a fundamental dilemma in the context of dynamic forest models. Relative to the time scale of forest development, even a 60‐yr time series of inventory data is short and does not allow us to fully test the behavior of a DVM. Since longer time series data (such as from palynology; e.g., Henne et al. 2011) typically have much lower temporal, spatial, and taxonomic resolution compared to more recent data such as forest inventories, paleoecological model tests are often not conclusive regarding species proportions and particularly their rates of change, either (cf. Lischke et al. 1999, Heiri et al. 2006). A promising way ahead may lie in the combined use of multiple data sources at different temporal and spatial scales to rigorously evaluate DVM simulation results; DVMs should be transferable in both space and time, constituting a necessary (but not sufficient) condition for their applicability under scenarios of climate change. Particularly regarding tree mortality, few such studies exist (Steinkamp and Hickler 2015).

### Sensitivity to mortality formulations vs. sensitivity to climate change

The simulation results of DVMs using different mortality formulations tended to be similar for up to a century into the future, irrespective of the scale for which the models were formulated. This is good news for model applications that are geared toward providing decision support for climate‐adaptive forest management, for example, as this is the time scale that is relevant for planning interventions toward the next tree generation (Seidl et al. 2011, Bircher 2015).

The longer‐term disagreement among simulation results using different mortality formulations is disconcerting, however, for example in the context of assessments of the role of forests in the global carbon cycle (cf. Friend et al. 2014). This clearly indicates that a better understanding of tree mortality is needed as well as an appropriate encapsulation of that understanding in tree mortality formulations for DVMs. Simply calling for empirically based models, which typically are based on decadal‐scale time series of inventory data, may not be the best solution (Keane et al. 2001) as such models may be overfitted to the peculiar environmental conditions under which the data were measured (Hülsmann et al. 2017). Thus, a more integrative approach relying on multiple data sources and possibly involving inverse Bayesian calibration is needed (Hartig et al. 2012), which can be viewed as a further development of “pattern‐oriented modeling” (Grimm et al. 2005). Such approaches are facilitated by the increasing availability of tree mortality data across large climatic gradients (Neumann et al. 2017) and by the use of remotely sensed information to improve the climate sensitivity of mortality models.

In some DVMs, sensitivity of simulated basal area and stem numbers to variations in the mortality formulation was similar, whereas in others these two variables reacted quite differently. This suggests that rather different feedbacks from tree mortality to regeneration are embodied in the various models, that is, in some models enhanced tree death gave rise to a surge of tree regeneration, whereas in others it did not. In this context, it has to be taken into account that some DVMs of our study are based on the assumption of unlimited seed availability (e.g., ForClim), whereas in others regeneration rates depend on the presence of seed trees (e.g., iLand), and in some DVMs, regeneration was turned off for the present simulations (e.g., 4C). This confirms that tree regeneration in addition to mortality is a highly sensitive process in at least some DVMs, which is in agreement with empirical findings (Clark et al. 1999, Martinez‐Vilalta and Lloret 2016). Subsequent research should address the interplay between these two crucial processes of population dynamics.

Trajectories of ecosystem development are often non‐linear (Scheffer et al. 2001). Therefore, the identification of tipping points (Scheffer 2010) is highly important in the context of forest dynamics in a changing climate (Williams et al. 2013, Reyer et al. 2015). Several of the DVMs investigated here featured tipping points, but they resulted from empirically based mortality models that may simply be outside of the range of their calibration data, thus possibly yielding erratic predictions. The more process‐based mortality models used in our study may not have featured tipping points because the design of the analysis was not to evaluate the impact of, for example, mega droughts (Dai 2011) or disturbances (Seidl and Rammer 2017), although inter‐annual variability of weather conditions was included in the climatic drivers of all models (cf. Appendix S1). A more in‐depth evaluation of the causes and features of mortality‐induced tipping points in DVMs by exploring a broader possibility of climate change scenarios through dedicated, quantitative sensitivity analyses would thus be highly valuable.

In many models, sensitivity to the formulation of mortality increased with the degree of climate change that was imposed on the DVMs. On the one hand, this indicates that most mortality formulations tend to share some common ground under current climatic conditions as well as under a moderate magnitude of climate change, which is encouraging, but on the other hand, it also indicates that model uncertainty is particularly large under high‐end scenarios of climate change (IPCC 2013), which, in the absence of dramatic emission reductions, are increasingly likely to materialize (Peters et al. 2013). This strongly complicates assessments of future feedback effects within the climate system that may be induced by the behavior of forest vegetation (cf. Sitch et al. 2008).

It also needs to be kept in mind that the exercise presented here differed not only in the structure of the DVMs that were employed, but also in the sites at which the simulations were conducted. It is possible that for certain combinations of site *x* climate conditions, sensitivity to the mortality formulation is low because mortality simply is not important, at least for a certain time frame (such as the 200 yr upon which our analyses were focused). For a more thorough assessment of the underlying causes of mortality in the models, more standardized and complex model comparisons would be needed. While we acknowledge that this is important, we consider our results as still valid because for some models that used the same site and climate (e.g., Peitz with 4C, FORMIND, and GOTILWA+; cf. Table 3), we still found strong differences that were in line with our overall results.

Lastly, our analysis clearly indicates that DVM sensitivity to different mortality formulations is at least of the same order of magnitude as sensitivity to climate change scenarios (i.e., RCP2.6 vs. RCP8.5). As long as we cannot supply robust models of tree mortality that can be used faithfully in DVMs under climate change conditions, the way toward robust projections could be to either use several, conceptually different model formulations, or formal sensitivity analysis in order to gauge the range and uncertainty of future forest trajectories.

## Conclusions

We provide the first study to comprehensively evaluate the sensitivity of DVMs to the formulation of mortality; we examined the reasons underlying the different sensitivity in the different DVMs in terms of the type and structure of the mortality formulation; and we compared the sensitivity of DVMs to mortality formulations with their sensitivity to climate change.

First, we conclude that a rich set of tree mortality algorithms is available today (cf. Hülsmann et al. 2017), and thus, structural sensitivity tests of DVMs with different mortality algorithms are perfectly feasible and should be emphasized in the future.

Second, we found widely different sensitivities of DVMs to different mortality formulations, with a large number of DVMs featuring high sensitivity. Our results are novel and helpful for guiding future model development as well as assisting the interpretation of model outcomes in terms of their uncertainty. Unfortunately, the identification of a suitable mortality formulation is not usually possible based on past data that typically cover several decades only. In spite of the attractiveness of empirically based algorithms (typically using forest inventory data), these tended to lead to most pronounced differences when subjected to scenarios of climate change. Due to the fact that under scenarios of climate change, these algorithms tend to be operating beyond the range of their calibration data, and their projections may often not be robust. Thus, the future of mortality modeling may lie more in synthetic approaches that embody various data sources rather than formulations that are based on a single set of temporally and spatially limited data.

Third, even though in the long run under future climate change vastly different model trajectories may be obtained, most mortality formulations led to good agreement of simulated trajectories for up to a century into the future. This is the time scale that is most relevant, for example, for decision making in ecosystem management, and hence, DVM results are useful in this context.

Fourth, for long‐term evaluations of, for example, the role of forests in the global carbon cycle, using only one mortality algorithm in DVM studies vastly underestimates the uncertainty of predictions. Hence, in future applications of DVMs to study the long‐term impacts of climate change on forests, let alone their feedback to the climate system, mortality processes should receive much more attention, so as to better embrace the uncertainty and range of possible future trajectories.

Lastly and most fundamentally, we conclude that the sensitivity of DVMs to the formulation of mortality is of at least the same order of magnitude and often larger than DVM sensitivity to climate change. Thus, mortality is one of the most uncertain ecosystem processes when it comes to assessing forest response to climate change. More data and a better process understanding of tree mortality are needed to improve the robustness of simulated future forest dynamics.
