# Supplementary material for: Tree mortality submodels drive simulated long‐term forest dynamics: assessing 15 models from the stand to global scale
Source: Ecosphere. 2019 Feb 20;10(2):e02616. doi: 10.1002/ecs2.2616 (PMC8609442; doi:10.1002/ecs2.2616)
Supplement: Supplementary file 1 [file ECS2-10-e02616-s001.pdf]

Appendix S1

for the manuscript by Bugmann, H. et al.,
Tree mortality submodels drive simulated long-term forest dynamics: an assessment across 15 models from the stand to the global scale

This document provides model descriptions, simulation settings and more detailed results of the individual simulation runs from all 15 models involved in this comparison.

TABLE OF CONTENTS

1. Simulation results from 4C ... 2
2. Simulation results from CARAIB ... 11
3. Simulation results from the Ecosystem Demography (ED) model ... 20
4. Simulation results from ForClim v3.0 ... 27
5. Simulation results from FORMIND ... 34
6. Simulation results from Climate-FVS ... 38
7. Simulation Results from GOTILWA+ ... 47
8. Simulation results from iLand ... 56
9. Simulation results from LandClim ... 75
10. Simulation results from LANDIS-II ... 80
11. Simulation results from LPJ-GUESS ... 87
12. Simulation results from LPJmL ... 99
13. Simulation results from PICUS ... 105
14. Simulation results from Sibyla ... 111
15. Simulation results from xComp ... 118

1. Simulation results from 4C

P. Lasch-Born, F. Suckow, C.P.O. Reyer, C. Kollas

Potsdam Institute for Climate Impact Research (PIK), Member of the Leibniz Association, Potsdam, Germany

1. Methods

1.1 Forest model

The model 4C ('FORESEE' - Forest Ecosystems in a Changing Environment) has been developed to describe long-term forest behavior under changing environmental conditions (Bugmann et al., 1997; Lasch et al., 2005; Reyer et al., 2010). It describes processes on the tree and stand level based on findings from eco-physiological experiments, long-term observations and physiological modeling. The model includes descriptions of tree species composition, forest structure, leaf area index as well as ecosystem carbon and water balances. Establishment, growth and mortality of tree cohorts are explicitly modeled on a patch on which horizontal homogeneity is assumed. The soil sub-model describes temperature, and water, carbon and nitrogen dynamics in different soil layers. 4C requires daily meteorological variables as well as a soil and a forest stand description.

1.2 Mortality formulations in 4C

Intrinsic (age-dependent) mortality

Based on a Weibull distribution depending on a_{max} (maximum age of species) and age of tree at a(t), the annual death rate p_{wint} of each species is defined:

$$p_{w\text{int}}(t) = \alpha_{w\text{int}} \cdot \lambda_{w\text{int}} \cdot a(t)^{\alpha_{w\text{int}}-1} \tag{1.1}$$

where α_{wint} (=0.7) and λ_{wint} are parameters of the Weibull distribution that have been empirically derived from long-term thinning experiments. The parameter λ_{wint} is calculated using the survival function S(t) of the Weibull distribution under the assumption that the survival probability at time t = a_{max} is equal 0.01.

$$\begin{aligned} S(a_{\max}) &= e^{-\lambda_{w\text{int}} \cdot a_{\max}^{\alpha_{w\text{int}}}} = 0.01 \\ \lambda_{w\text{int}} &= \frac{-\ln(0.01)}{a_{\max}^{\alpha_{w\text{int}}}} \end{aligned} \tag{1.2}$$

The parameter α_{wint} determines the increase or decrease of mortality rate with age.

Stress-induced mortality with foliage growth as criterion

This mortality formulation assumes that mortality depends on the tree's carbon balance. A measure of the carbon balance of a tree cohort is the yearly foliage growth dM_f/dt. If the foliage growth is less than zero the tree is not able to reproduce the same amount of foliage as last year and the tree experiences stress (Mäkelä, 1986). To take into account not only the carbon balance of the actual year t, a concept developed by Keane et al. (1996) is used. The model counts the following years of stress

of each tree cohort by the stress counter c_{stress} and years without stress by the counter c_{health} in the following way:

$$c_{stress}(t) = \begin{cases} c_{stress}(t-1)+1 & \frac{dM_f}{dt} < 0 \\ c_{stress}(t-1)-1 & c_{health}(t) > 0 \quad \wedge c_{stress}(t-1) \neq 0 \\ 0 & otherwise \end{cases} \qquad (1.3)$$

and

$$c_{health}(t) = \begin{cases} c_{health}(t-1)+1 & \frac{dM_f}{dt} \geq 0 \\ 0 & \frac{dM_f}{dt} < 0 \end{cases} \qquad (1.4)$$

The stress counter is used as a predictor to calculate an annual probability of mortality of each tree cohort due to stress using the hazard function of the Weibull distribution:

$$p_{stress}(c_{stress}(t)) = \alpha_{wstress} \cdot \lambda_{wstres} \cdot c_{stress}(t)^{\alpha_{wstress}-1} \qquad (1.5)$$

where $\alpha_{wstress}$ (=1.5) and $\lambda_{wstress}$ are scale and shape parameter of the Weibull distribution that have been empirically derived from long-term thinning experiments. The parameterization of the function is species-specific due to different tolerance of species with regard to stress. We define five tolerance classes j concerning stress and for each class a variable Y_s, which reflects the number of stress years leading to mortality with nearly 100%. The tolerance class is defined by the species specific parameter shade tolerance p_{st} (Ellenberg, 1996), according to Keane et al. (1996). The variable Y_s is defined by a table function (see

Table 1) for each species. The parameter $\lambda_{wstress}$ is calculated using the survival function S(t) of the Weibull distribution assuming that after Y_s years the survival probability of a tree cohort is 0.01:

$$S(Y_s) = e^{-\lambda_{wstress} \cdot Y_s^{\alpha_{wstress}}} = 0.01$$
$$\lambda_{wstress} = \frac{-\ln(0.01)}{Y_s^{\alpha_{wstress}}} \qquad (1.6)$$

Table 1: Parameters for stress mortality according to (Keane et al., 1996).

Shade tolerance class (p_{st})	Number of stress years (Y_s)
**1**	20
**2**	40
**3**	60
**4**	80
**5**	100

Stress-induced mortality by NPP-criterion

This formulation is similar to the "Stress- induced mortality with foliage growth as criterion" but instead of yearly foliage growth (dM_f/dt) overall NPP is used as a criterion to define if the tree experiences stress. Therefore, the following criterion is used to define stress in formula (1.3) and (1.4):

$$NPP(t) < S_f(t) + (1+\alpha_c)S_s(t) + S_r(t) \tag{1.7}$$

with the senescence rates of foliage (S_f), sapwood (S_s), fine roots (S_r), twigs, branches & coarse roots to sapwood ratio (α_c). This criterion is similar to the vitality index (relation between net primary production and carbon loss due to compartment senescence) in the model FORSANA (Grote and Erhard, 1999), where stress mortality is calculated:

$$p_{stress}(c_{stress}(t)) = \alpha_{wstress} \cdot \lambda_{wstres} \cdot \left(\frac{c_{stress}(t)}{3}\right)^{\alpha_{wstress}-1} \tag{1.8}$$

Parameters are the same as in the 4C approach (b).

Total mortality

Total mortality is calculated as follows:

$$p_{mort}(t) = p_{wint}(t) + (1 - p_{wint}(t)) \cdot p_{stress}(t) \tag{1.9}$$

2. SIMULATION SETTINGS

2.1 SITE DATA

- Site: Peitz, Germany, Species: Scots pine (dry site with sandy soil)
- Measured stand data available for 1948-2011
- Climate data available for 1948-2011 from German weather station "Lieberose" (distance 4 km)
- Soil data are derived from personal information (M. Noack Eberswalde, see also CA FP1304 PROFOUND database)
- Nitrogen deposition according to EMEP data base (Co-operative Programme for Monitoring and Evaluation of the Long-range Transmission of Air Pollutants in Europe, http://www.nilu.no/projects/ccc/emepdata.html)
- Climate scenarios used: RCP2.6, RCP8.5 from IMPACT2C (bias corrected):
  - MPI-M-ESM-LR > MPI-CSC-REMO2009
  - ICHEC-EC-EARTH > SMHI-RCA4
- Historical increase of atmospheric CO_2 following the Mauna Loa data

2.2 TEST DATA

We compare the dbh, height, stem biomass, tree number and basal area simulated by 4C with the corresponding data available for the Peitz stand. These data have not been used for parameterizing/calibrating the mortality model. The data are available via Reyer et al. (in prep.).

2.3 SIMULATION EXPERIMENTS

a) Simulation with observed climate (1948-2011)

  - Stand has been initialized for the year 1948
  - Silvicultural management has not been simulated
  - Historical CO_2 increase (Source: dataset from Mauna Loa)
  - N deposition, on average 15 kg N/ha per year

b) Simulation with climate scenarios (2011-2100)
  - Simulation starts with generated (tree cohort) initialization in 2010, which is the finale state of the simulation 1948-2010
  - Climate scenarios for 2011-2100 from MPI-CSC-REMO2009 and SMHI-RCA4
  - Constant CO_2 (400 ppm) for RCP2.6 and RCP8.5 ➔We assume constant CO_2 concentration to exclude (strong) fertilization effects.
  - N deposition, on average 9 kg N/ha per year

We did not perform an experiment with a long-term climate scenario (2100-2200), because in the current 4C version, regeneration and/or planting requires some further testing and therefore has been disabled for all simulations (also for a and b listed above). Extending the simulations beyond 2100 without regeneration would oversimplify existing forest dynamics. We think that the sensitivity of 4C with regard to different mortality formulations can be sufficiently tested/displayed using the experiments until 2100.

Climate scenarios

The description of absolute changes (temperature) and relative changes (precipitation, global radiation, relative humidity) are given for 4 climate scenarios (Tables 2,3) generated with the regional climate models MPI-CSC-REMO2009 and SMHI-RCA4 (source: EU project IMPACT2C).

Table 2 Temperature and precipitation changes 2071-2100 versus 1981-2010 and CO_2 concentration of simulation period

	**Temperature (delta values, K)**				**Precipitation (mult.)**				**CO_2 (ppm)**
**Scenario**	DJF	MAM	JJA	SON	DJF	MAM	JJA	SON	400
**REMO RCP2.6**	+1.50	+0.18	+0.55	+0.15	1.07	1.03	0.94	1.19	400
**REMO RCP8.5**	+4.15	+1.59	+1.98	+2.77	1.14	1.25	1.06	1.19	400
**RCA RCP2.6**	+0.88	+0.58	+0.90	+0.63	1.03	0.98	0.97	0.99	400
**RCA RCP8.5**	+4.25	+3.09	+3.65	+3.58	1.12	1.25	0.97	0.98	400

Table 3 Global radiation and relative humidity changes 2071-2100 versus 1981-2010

	**Global radiation (mult.)**				**Relative humidity (mult.)**			
**Scenario**	DJF	MAM	JJA	SON	DJF	MAM	JJA	SON
**REMO RCP2.6**	1.03	1.06	1.03	1.04	1.02	1.02	1.04	0.95
**REMO RCP8.5**	0.98	0.97	0.96	1.00	1.01	1.06	1.11	0.95
**RCA RCP2.6**	0.99	1.06	1.04	1.04	0.99	1.09	1.00	0.98
**RCA RCP8.5**	0.94	1.02	1.04	1.05	1.01	1.14	1.07	0.98

3. Simulation results

Simulation without management 1948-2010 and intrinsic mortality and stress via criterion of foliage growth

The simulated mortality (in terms of stem number) corresponds well to tree numbers observed at the site. This shows that the conservative thinning from below applied in the stand in reality was designed to match mortality (removal of the same amount of stems that would die due to self-thinning). The simulations for (mean) height and (mean) dbh fit the observed values well, although height tends to be underestimated towards the end of the simulation period. Stem biomass as shown on the plots is overestimated because the “observed biomass” is derived from volumes of merchantable wood using a tree form factor. This derived biomass is only considering stems larger than 7cm dbh leading to lower stem wood biomass than simulated with 4C. This overestimation decreases over time as the proportion of older and thicker stems increases.

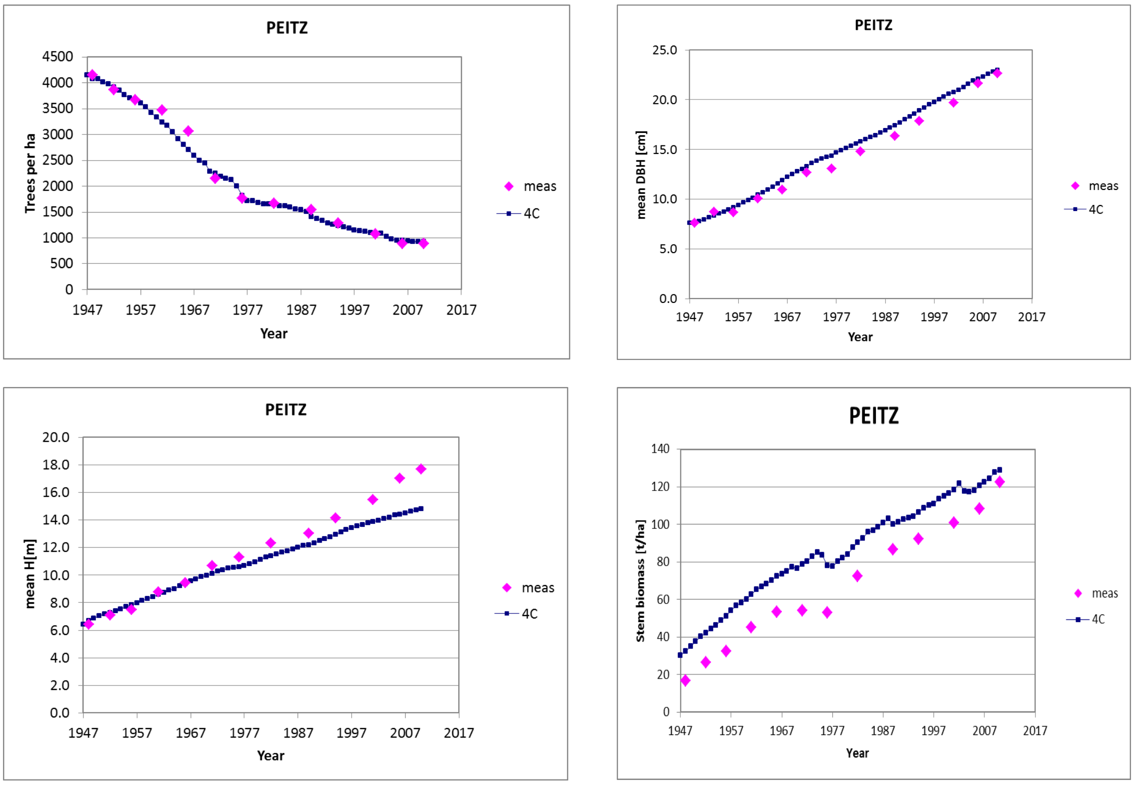

Fig. 1: Comparison of simulated values with measured data

Simulation without management, alternative mortality approach (intrinsic mortality and stress via NPP criterion)

The alternative mortality formulation based on NPP seems to fit the observed values slightly less well than the original approach (slightly stronger overestimation of biomass, basal area, underestimation of height). However, generally the differences between the two approaches are small.

Mortality due to the alternative approach for stress mortality (NPP criterion) is a little bit lower than with the standard approach in 4C (Table 4). The final diameter distributions differ for both approaches, with the alternative approach having more trees in the lower diameter classes (20, 22 cm).

Simulation with climate scenarios (2011-2100)

The effects of the climate scenarios (RCP2.6 versus RCP8.5) on the considered variables are greater than the effects of changing the stress mortality criterion from the standard approach to the alternative approach. With the alternative approach the average mortality rate is slightly lower than with the standard approach (see Tables 5,6) for three of the four simulations of climate scenarios.

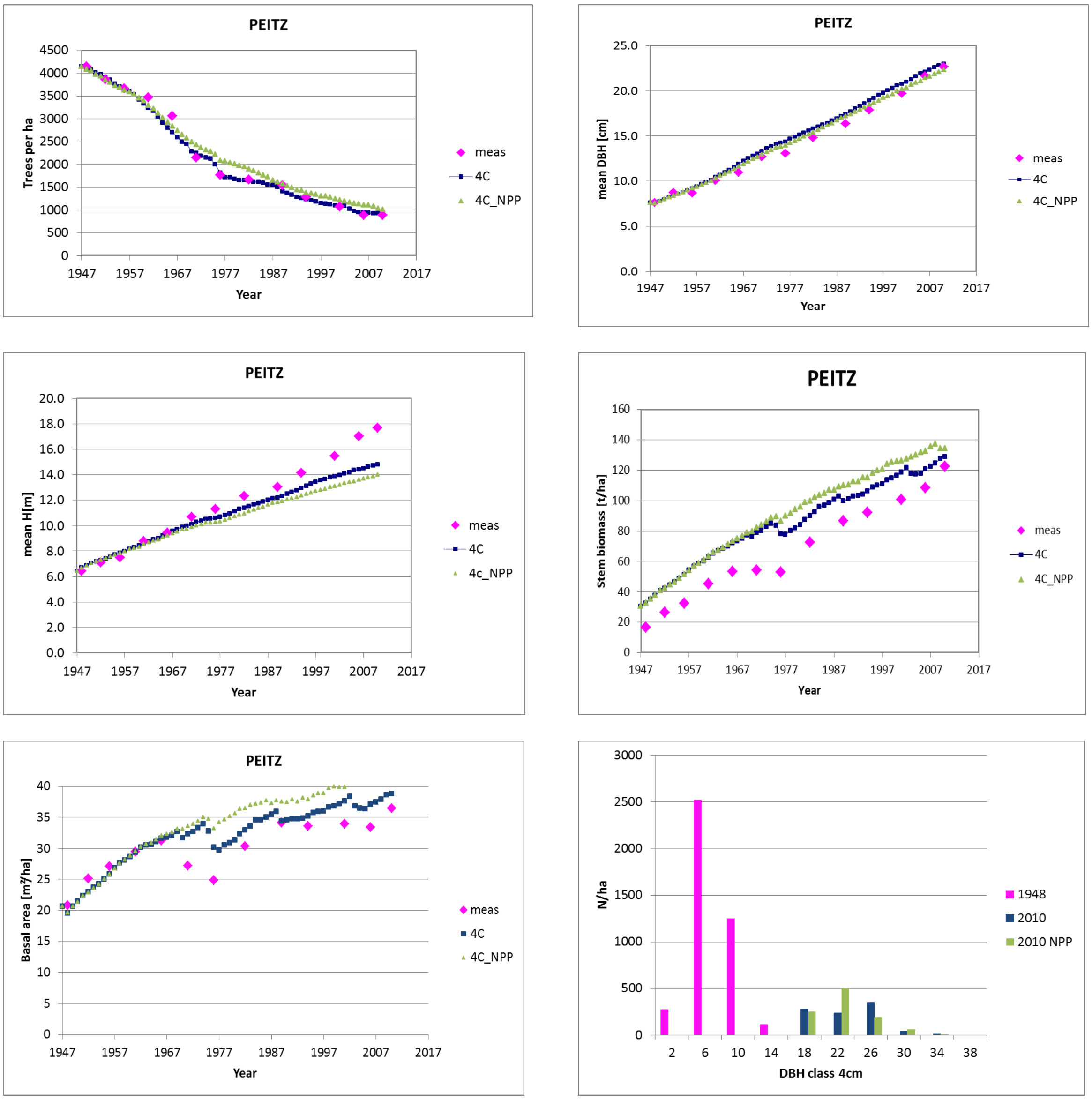

Figure 2 Comparison of simulated values with both mortality approaches (blue line: foliage increment criterion, green line: NPP criterion) and measurement data (pink diamonds); Diameter distribution according to 4cm classes

Table 4 Comparison of standard and alternative approach for stress mortality

	Standard approach	Alternative approach
**Mean annual mortality rate [t DW ha^{-1} a^{-1}]**	1.39	1.32
**Final basal area [m²]**	38.8	40.4
**Final stem number [ha^{-1}]**	920	1010
**Final stem volume [m³ ha^{-1}]**	319	332

Table 5 Stress mortality simulated with standard approach (leaf growth-based)

	REMO RCP2.6	REMO RCP8.5	RCA4 RCP2.6	RCA4 RCP8.5
**Mean annual mortality rate [t DW ha^{-1} a^{-1}]**	2.13	1.95	2.02	1.87
**Final basal area [m²]**	45.4	52.0	45.2	50.0
**Final stem number [ha^{-1}]**	240	290	250	260
**Final stem volume [m³ ha^{-1}]**	533	607	529	599

Table 6 Stress mortality simulated with alternative approach (NPP-based)

	REMO RCP2.6	REMO RCP8.5	RCA4 RCP2.6	RCA4 RCP8.5
**Mean annual mortality rate [t DW ha^{-1} a^{-1}]**	2.0	1.97	2.01	1.84
**Final basal area [m²]**	48.5	51.3	46.0	51.0
**Final stem number [ha^{-1}]**	260	280	260	270
**Final stem volume [m³ ha^{-1}]**	565	602	535	610

4. REFERENCES

Bugmann, H., Grote, R., Lasch, P., Lindner, M. and Suckow, F., 1997. A new forest gap model to study the effects of environmental change on forest structure and functioning. In: G.M.J. Mohren, K. Kramer and S. Sabaté (Editors), Impacts of Global Change on Tree Physiology and Forest Ecosystems. Forestry Sciences. Kluwer Academic Publishers, Dordrecht, pp. 255-261.

Ellenberg, H., 1996. Vegetation Mitteleuropas mit den Alpen in ökologischer Sicht, 5. Aufl. Ulmer Verlag, Stuttgart, 989 pp.

Grote, R. and Erhard, M., 1999. Simulation of tree and stand development under different environmental conditions with a physiologically based model. Forest Ecology & Management, 120(1-3): 59-76.

Keane, R.E., Morgan, P. and Running, S.W., 1996. FIRE-BGC - A mechanistic ecological process model for simulating fire succession on coniferous forest landscapes of the northern Rocky Mountains. INT-RP-484, United States Department of Agriculture, Forest Service, Intermountain Research Station, Ogden, UT.

Lasch, P., Badeck, F.W., Suckow, F., Lindner, M. and Mohr, P., 2005. Model-based analysis of management alternatives at stand and regional level in Brandenburg (Germany). Forest Ecology And Management, 207(1-2): 59-74.

Mäkelä, A., 1986. Implications of the pipe model theory on dry matter partitioning and height growth trees. J. Theor. Biol., 123: 103-120.

Reyer, C., Lasch, P., Mohren, G.M.J. and Sterck, F.J., 2010. Inter-specific competition in mixed forests of Douglas-fir (Pseudotsuga menziesii) and common beech (Fagus sylvatica) under climate change - a model-based analysis. Annals Of Forest Science, 67(8): 805.

Reyer CPO, R Silveyra Gonzalez, K Dolos, F Hartig, Y Hauf, M Noack, P Lasch-Born,T Rötzer, H Pretzsch, H Meesenburg, S Fleck, M Wagner, A Bolte, T Sanders, P Kolari, A Mäkelä, J Pumpanen, G Matteucci, A Collalti, E D'Andrea, L Krupkova, J Krejza, A Ibrom, K Pilegaard, D Loustau, J-M Bonnefond, P Berbigier ,D Picart, S Lafont, M Dietze, D Cameron, M Vieno, H Tian, A Palacios, V Cicuendez, M Büchner, S Lange, J Volkholz, J Horemans, S Martel, F Bohn, J Steinkamp. I. Vega del Valle, A Chikalanov, K. Frieler. in prep. The PROFOUND database for evaluating vegetation models and simulating climate impacts on forest stands. To be submitted to Earth Science Data.

2. SIMULATION RESULTS FROM CARAIB

Louis François, Alexandra-Jane Henrot, Ingrid Jacquemin

Unit for Modelling of Climate and Biogeochemical Cycles, UR SPHERES, University of Liège, Bât B5c, Quartier Agora, Allée du Six Août 19C, B-4000 Liège, Belgium

(Louis.Francois@ULG.AC.BE ; Alexandra.Henrot@ULG.AC.BE ; Ingrid.Jacquemin@ULG.AC.BE)

1. METHODS

1.1 THE CARAIB MODEL

The CARAIB model (CARbon Assimilation In the Biosphere) is a global dynamic vegetation model (Dury et al., 2011). It is made up of five modules respectively describing (1) the hydrological budget, (2) canopy photosynthesis and stomatal regulation, (3) carbon allocation and plant growth, (4) heterotrophic respiration and litter/soil carbon dynamics, and (5) plant competition and biogeography.

The model takes into account a set of herbaceous and tree species (for the current study, 1 herb and 40 trees are considered, see the list in Annex 1), which can coexist on the same grid cell and compete for resources (space, light and water). For each of these species, the model calculates the temporal evolution of two carbon reservoirs in the plant (metabolic and structural carbon) and three in the soil (metabolic and structural litter, soil organic carbon). The model time step is one day for updating all water and carbon reservoirs. For photosynthesis and plant respiration a shorter time step of two hours is used, allowing us taking into account non-linear effects associated with the variation of photosynthetic/respiration fluxes over the day. Vegetation cover is updated once a month for herbs and once a year for trees. Spatially, the CARAIB model is a grid-point model and it can be used with different spatial resolutions.

The input climatic fields that are necessary to run the model are: (1) the diurnal mean air temperature (Td=0.5(Tmax+Tmin)), (2) the diurnal amplitude of air temperature change between day and night (ΔT=Tmax-Tmin), (3) precipitation, (4) air relative humidity, (5) percentage of sunshine hours and (6) wind speed. Daily values of all these variables are required. The soil texture (average percentages of silt, sand and clay in the root zone) is also required to setup the model.

1.2 MORTALITY FORMULATION

In CARAIB, in the absence of age and size classes, the default calculation of mortality rate for trees is the inverse of a characteristic time for wood *gkfall*, fixed in the simulations presented here to 40 years for warm temperate/Mediterranean species and to 80 years for boreal/cold temperate species. To this background mortality rate, is added a stress-induced mortality rate during cold or dry periods, i.e., when the daily air temperature or the root zone soil water content falls below a species-dependent threshold. The characteristic times for stress-induced mortality are fixed to 6 days for temperature and 2 months for soil water stresses.

In the sensitivity test illustrated here, we have replaced the background mortality formulation with a dynamic mortality formulation related to growth efficiency, derived from the one used in LPJ (Sitch et al., 2003, Glob. Chang. Biol. 9, 161–185):

$$M_{BG} = (\frac{k_{BG}}{1 + 0{,}035V})/365$$

where M_{BG} is the dynamic background mortality rate per day for trees, k_{BG} is the maximum background mortality rate (set to 0.1 yr^{-1} for all trees) and V is vigor or growth efficiency, defined as the ratio of the net annual biomass increment to maximum LAI of the preceding year. V equals to 0 in case of net annual biomass loss. In this new formulation, the stress-induced mortality has been suppressed, since it is already included through the growth efficiency parameter.

2. SIMULATION SETTINGS

2.1 SITES

We defined a set of 6 forest sites for which the model comparison has been run. The list of sites and corresponding information are given in the table below.

Site name	Country	Lat	Long	Dominant Species
**Brasschaat**	Belgium	51.31	4.5205	Pinus sylvestris
**Colellongo**	Italy	41.85	13.5881	Fagus sylvatica
**Hyytiala**	Finland	61.85	24.295	Pinus sylvestris,<br>Picea abies
**Peitz**	Germany	51.95	14.36	Pinus sylvestris
**Soroe**	Denmark	55.48	11.64	Fagus sylvatica
**Vielsalm**	Belgium	50.30	6.00	Fagus sylvatica

2.2 TEST DATA

The sites used for the model experiments are forest sites from the FLUXNET network (http://fluxnet.ornl.gov). They are equipped with eddy covariance systems for the measurement of the exchange fluxes of heat, water and carbon dioxide between the ecosystem and the atmosphere. They also provide time series of the gross primary productivity (GPP) of the site based on these measurements. We will use these GPP estimates to test our model over the years when the eddy covariance experiments have been run. Note that the data available on the sites have not been used for calibration.

2.3 SIMULATION EXPERIMENTS

For each site, we extracted the climatic data and GCM outputs from the 0.5°x0.5° grid-cell in which the forest site is located. The climatic data have been made available via the ISI-MIP2 project (www.isi-mip.org). We have used the climatic data of the Princeton (PGFv2) dataset from 1901 to 2012 to force the runs. The model was initialized through a spin-up of 136 years before 1901, where the ISI-MIP2 detrended data for the period 1901-1930 were repeated as necessary. Future runs have been forced with the bias corrected climate of the GFDL-ESM2M GCM (Hempel et al., 2013, ESD). We selected GCM data for the time period from 1951 to 2099 and for all RCPs (RCP 2.6, 4.5, 6.0 and 8.5). The climatic forcings for the time period from 2100 to 2199 have been generated by randomly cycling through the last 20 years of the corresponding climate scenario run. All runs using GCM data as inputs were initialized with CARAIB outputs of the Princeton historical simulation at the end of year

1950. The temperature and precipitation changes reached in the GFDL-ESM2M climate simulation at the end of the 21st century (2081-2100) compared to the end of the 20th century (1981-2000) are reported in Annex 2 for all RCP scenarios and all sites.

Soil data for each site have been derived from the Harmonized World Soil Database, version 1.21 (http://webarchive.iiasa.ac.at/Research/LUC/External-World-soil-database/HTML/). The current simulations of the CARAIB model do not account for forest management. The species composition of the forest has not been fixed; the model is thus free to calculate the fraction of species present on the sites.

3. Simulation results

3.1 Comparison with site eddy covariance data

Figure 1 shows a comparison of the model GPP for the standard and the altered mortality formulation with on-site GPP estimates from eddy covariance (EC) measurements taken between 1997 and 2011 at the Vielsalm (BE) site. Both mortality formulations provide very similar results for GPP over this measurement period and this simulated GPP is relatively close to the one derived from the EC data, although the model shows much lower fluctuations from day to day or from year to year than the data. This is partly linked to the fact that we have not used on-site meteorological measurements to force the model, but average data from a 0.5°x0.5° grid cell in the ISI-MIP2 database. It is, however, also partly linked to the inability of the model to produce very high values of GPP, such as those exhibited by the EC data during 1998 or 2009.

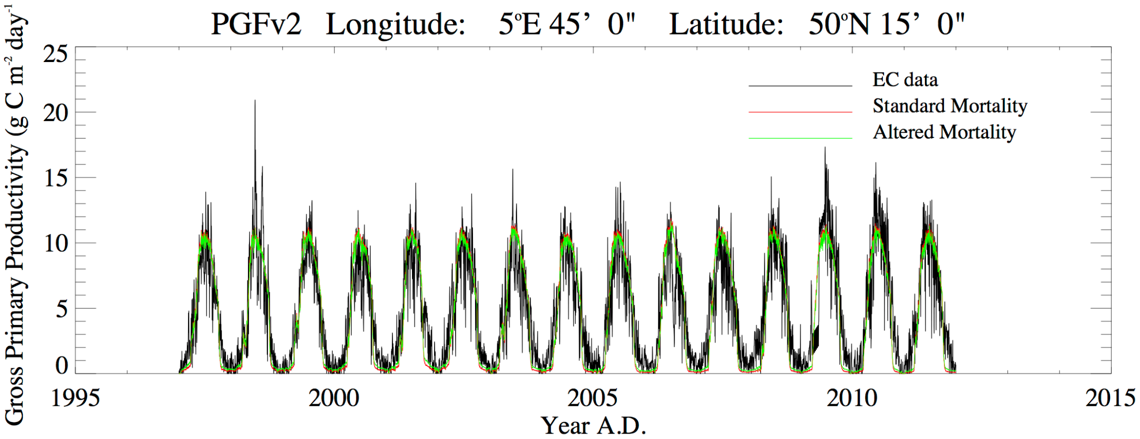

Fig. 1 Simulated GPP at Vielsalm (BE) site for standard and altered mortality formulations and comparison with GPP derived from eddy covariance data (EC data)

3.2 Future scenarios

3.2.1 Effect of mortality formulation on biomass

As illustrated in Figure 2 for the Brasschaat (BE) site, the impact of changing the formulation of background mortality is highest on simulated biomass. For the altered formulation with vigor-dependent mortality rates, CARAIB produces significantly lower biomass than for the standard formulation. This is true for several sites and some of the tree species, but it is not a general feature. For instance, at the Soroe (DK) site, the altered formulation tends to produce slightly higher biomass.

For the standard formulation, the evolution of biomass is punctuated by a series of sharp drops in biomass, associated with stress-induced mortality. Figure 3 indicates that these drops are linked to soil water stress. Such an event already occurs in the historical period (in the 1950's). It is likely over-

estimated, suggesting a very high sensitivity of the model to water stress. Similar events are regularly repeated in the future and are more frequent under the RCP8.5 scenario. The sensitivity to water stress looks to be reduced in the 22nd century, possibly as a response to higher levels of atmospheric CO_2, which reduce transpiration losses through stomatal closure. With the altered mortality formulation, drops of biomass are also associated with the soil water stress events, but they are much smaller in amplitude.

(a)

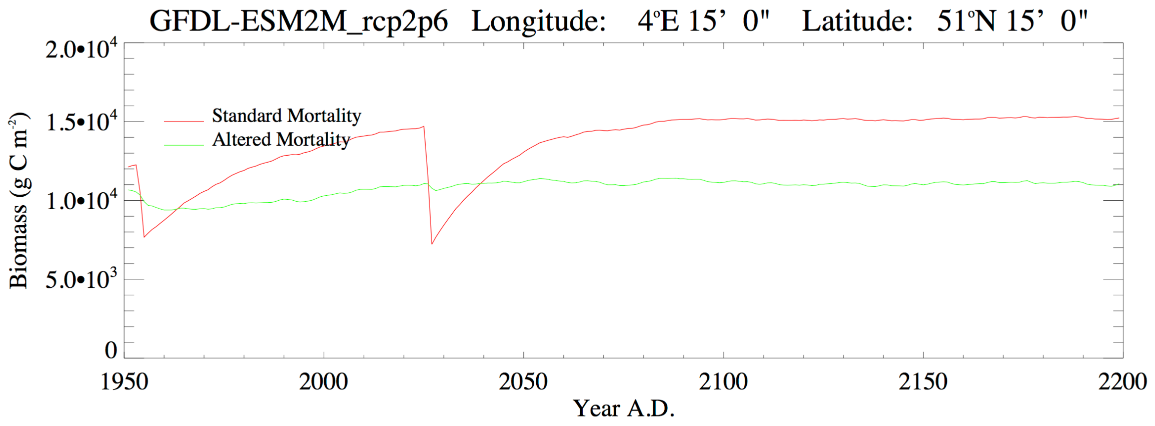

(b)

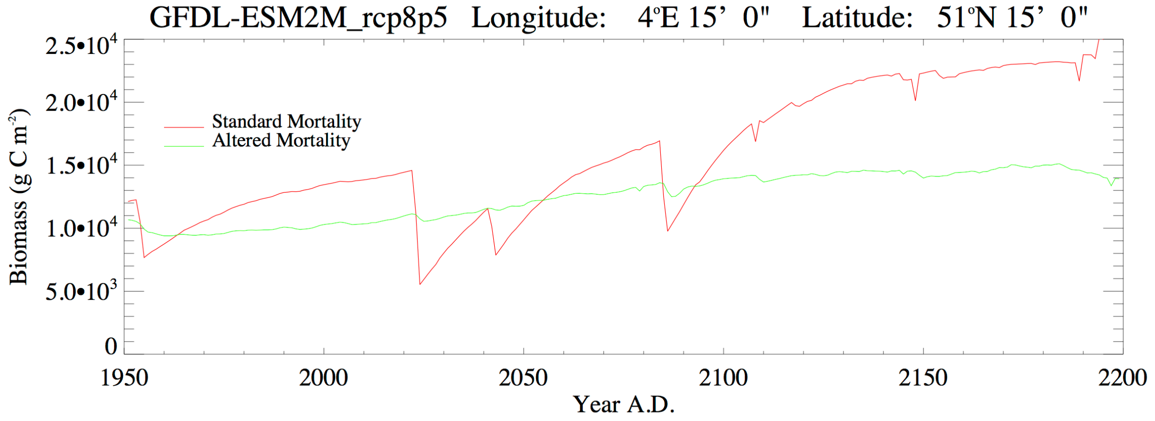

Figure 2: Evolution of total annual biomass at the Brasschaat (BE) site over the period 1950-2199 for scenario (a) RCP2.6 and (b) RCP 8.5

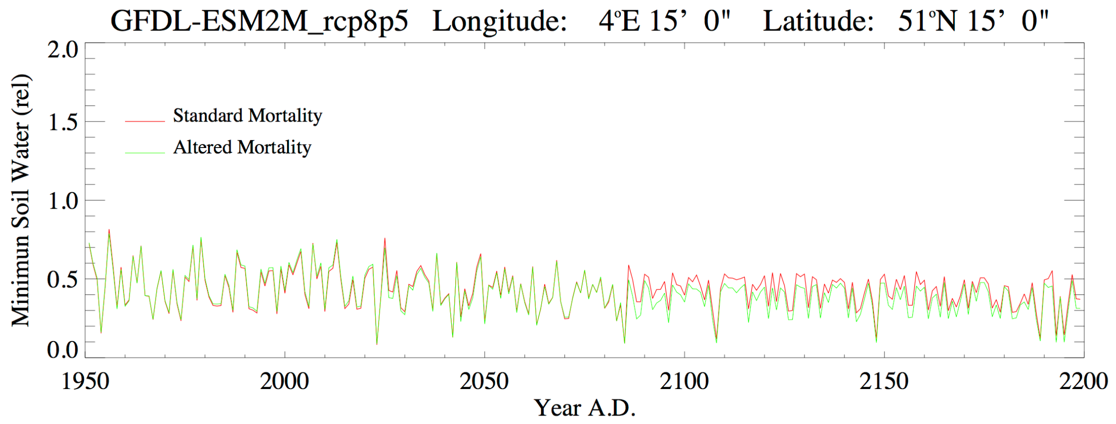

Figure 3: Minimum daily value of soil water content in the root zone reached at the Brasschaat (BE) site over each year between 1950 and 2200 for the RCP8.5 scenario. Soil water content is expressed in relative units, as (SW-WP)/(FC-WP), where SW is soil water content, WP is wilting point and FC is field capacity.

3.2.2 Effect of mortality formulation on species survival

As shown in Figure 4, the two mortality formulations provide significantly different results for the biomass of *Ulmus glabra* at the Brasschaat site. As observed for total biomass in section 3.2.1, the species biomass evolution is much smoother with the altered mortality. It seems to be the case for most of the tree species simulated on the selected sites. At Brasschaat, *Ulmus glabra* is not able to survive until 2200 when the standard formulation of mortality is used: it disappears (biomass falls to 0) near 2173 under the RCP2.6 scenario and near 2085 under RCP8.5. By contrast, with the altered mortality formulation, it is able to survive at the site until 2200 under both climatic scenarios.

(a)

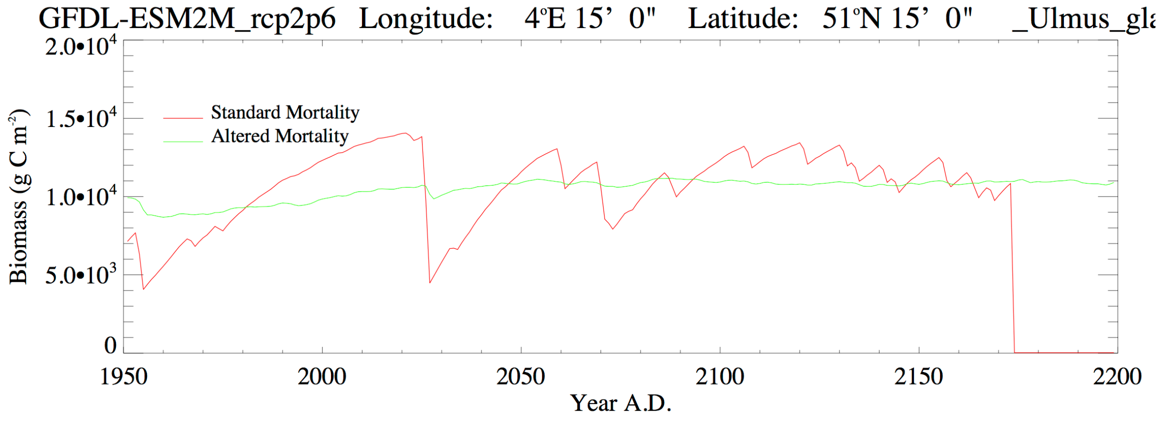

(b)

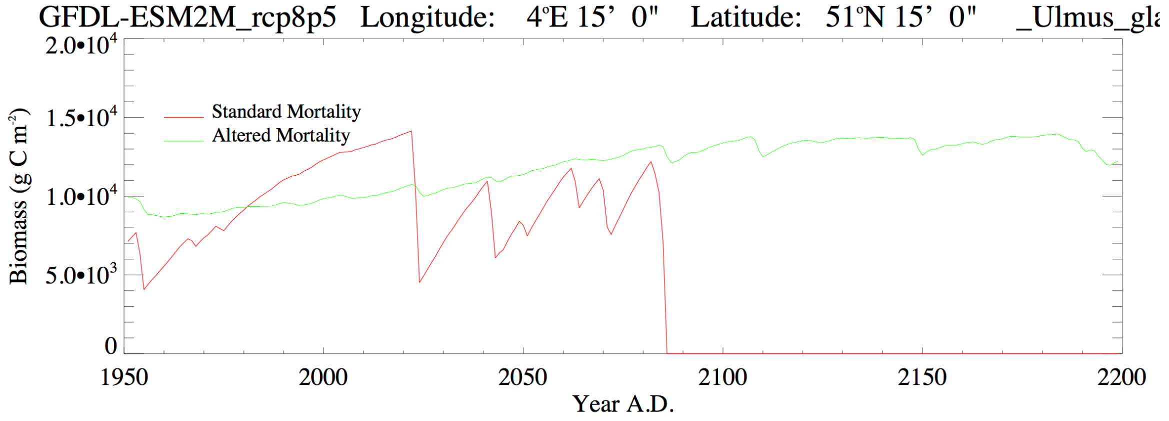

Figure 4: Evolution of *Ulmus glabra* biomass in Brasschaat (BE) over the period 1950-2199 for scenarios (a) RCP 2.6 and (b) RCP 8.5

3.2.3 Effect of mortality formulation on species net primary productivity

As illustrated in Figure 5 for *Fagus sylvatica* at the Vielsalm site under the RCP8.5 scenario, both mortality formulations provide very similar results for the NPP evolution of the simulated tree species in the future. The response of NPP to drought events is, however, slightly more pronounced with standard mortality.

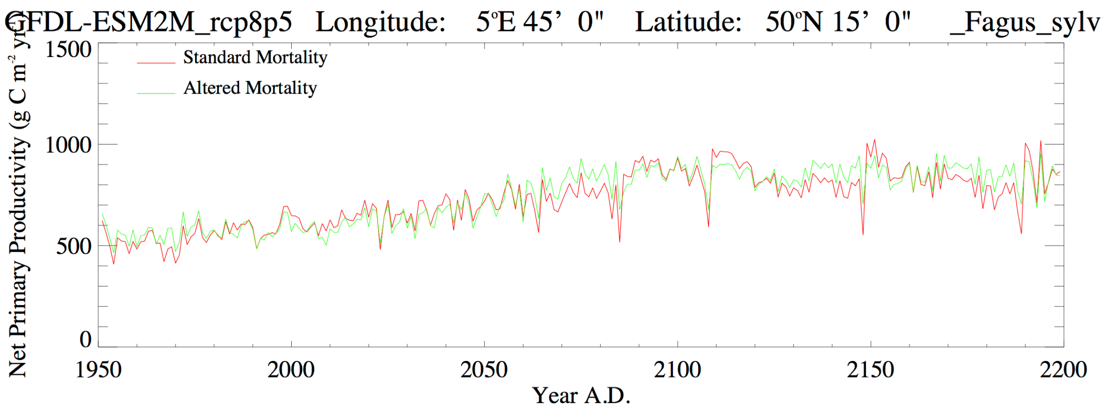

Figure 5: Evolution of *Fagus sylvatica* NPP in Vielsalm (BE) over the period 1950-2199 under the RCP 8.5 scenario.

4. References

Dury, M., Hambuckers, A., Warnant, P., Henrot, A.-J., Favre, E., Ouberdous, M., and François, L. : Response of the European forests to climate change: a modelling approach for the 21st century. iForest 4, 82-99, 2011.

Hempel, S., Frieler, K., Warszawski, L., Schewe, J., Piontek, F. A trend-preserving bias correction - the ISI-MIP approach. Earth Syst. Dynam., 4, 219-236, 2013.

Sitch, S., Smith, B., Prentice, I. C., Arneth, A., Bondeau, A., Cramer, W., Kaplan, J. O., Levis, S., Lucht, W., Sykes, M. T., Thonicke, K., and Venevsky, S.: Evaluation of ecosystem dynamics, plant geography and terrestrial carbon cycling in the LPJ dynamic global vegetation model. Glob. Change Biol., 9, 161-185, 2003.

Annex 1: Tree species simulated with CARAIB

Abies alba
Acer campestre
Alnus glutinosa
Alnus incana
Betula pendula
Betula pubescens
Carpinus betulus
Carpinus orientalis
Castanea sativa
Corylus avellana
Cupressus sempervirens
Fagus sylvatica
Fraxinus excelsior
Juniperus communis subsp. communis
Larix decidua
Olea europaea
Ostrya carpinifolia
Picea abies
Pinus halepensis
Pinus mugo
Pinus pinaster
Pinus pinea
Pinus sylvestris
Populus nigra
Quercus coccifera
Quercus ilex
Quercis petraea
Quercus pubescens
Quercus robur
Salix alba
Salix eleagnos
Salix hastata
Salix purpurea
Salix retusa
Taxus baccata
Tilia cordata
Tilia platyphyllos
Ulmus glabra
Ulmus laevis
Ulmus minor

ANNEX 2: CLIMATIC ANOMALIES IN THE RCP SCENARIOS

Table 1: Temperature anomalies (delta, °C), precipitation anomalies (multipliers, unitless) and CO2 concentration for the site of Brasschaat (BE). The anomalies are calculated from the seasonal means for the period 2081-2100 and the seasonal means for the reference period 1981-2000. The absolute values for the reference period are given in the first line of the table (temperature is in °C and precipitation in mm/month). The CO2 concentration is the mean value for years 1981-2000 or 2081-2100.

	Temperature (delta, °C)				Precipitation (mult., unitless)				CO_2(ppm)
**Scenario**	DJF	MAM	JJA	SON	DJF	MAM	JJA	SON	
**Hist**	4.052	12.531	16.648	7.269	62.337	61.239	71.551	79.213	353.805
**RCP2.6**	1.047	0.532	0.491	0.764	1.093	0.970	1.033	1.184	425.891
**RCP4.5**	1.268	0.650	0.939	1.213	1.061	1.102	0.938	1.137	534.288
**RCP6.0**	2.208	1.307	2.183	1.991	1.172	1.005	0.809	1.167	636.153
**RCP8.5**	2.443	1.994	2.996	2.319	1.126	1.044	0.807	1.211	849.615

Table 2: Temperature anomalies (delta, °C), precipitation anomalies (multipliers, unitless) and CO_2 concentration for the site of Collelongo (IT). The anomalies are calculated from the seasonal means for the period 2081-2100 and the seasonal means for the reference period 1981-2000. The absolute values for the reference period are given in the first line of the table (temperature is in °C and precipitation in mm/month). The CO_2 concentration is the mean value for years 1981-2000 or 2081-2100.

	Temperature (delta, °C)				Precipitation (mult., unitless)				CO_2(ppm)
**Scenar-io**	DJF	MAM	JJA	SON	DJF	MAM	JJA	SON	
**Hist**	8.329	16.982	22.509	11.954	91.684	65.651	50.302	111.328	353.805
**RCP2.6**	0.997	0.569	1.378	0.968	0.894	1.028	0.942	0.924	425.891
**RCP4.5**	1.025	1.262	2.277	0.967	0.908	0.815	0.954	0.953	534.288
**RCP6.0**	1.611	1.393	3.539	1.906	0.910	0.951	0.714	0.836	636.153
**RCP8.5**	2.017	2.879	5.193	2.757	0.820	0.648	0.682	0.899	849.615

Table 3: Temperature anomalies (delta, °C), precipitation anomalies (multipliers, unitless) and CO_2 concentration for the site of Hyytiala (FI). The anomalies are calculated from the seasonal means for the period 2081-2100 and the seasonal means for the reference period 1981-2000. The absolute values for the reference period are given in the first line of the table (temperature is in °C and precipitation in mm/month). The CO_2 concentration is the mean value for years 1981-2000 or 2081-2100.

	Temperature (delta, °C)				Precipitation (mult., unitless)				CO_2(ppm)
**Scenario**	DJF	MAM	JJA	SON	DJF	MAM	JJA	SON	
**Hist**	-7.253	8.525	12.308	-1.072	44.115	48.446	78.685	62.942	353.805
**RCP2.6**	2.680	1.696	1.785	1.857	1.139	0.936	1.022	1.089	425.891
**RCP4.5**	2.450	1.543	2.019	2.499	1.111	1.146	1.016	1.067	534.288
**RCP6.0**	4.043	2.841	2.577	3.756	1.173	1.007	1.156	1.144	636.153
**RCP8.5**	6.235	4.491	3.769	5.317	1.278	1.041	0.976	1.225	849.615

Tab. 4: Temperature anomalies (delta, °C), precipitation anomalies (multipliers, unitless) and CO_2 concentration for the site of Peitz (GE). The anomalies are calculated from the seasonal means for the period 2081-2100 and the seasonal means for the reference period 1981-2000. The absolute values for the reference period are given in the first line of the table (temperature is in °C and precipitation in mm/month). The CO_2 concentration is the mean value for years 1981-2000 or 2081-2100.

	Temperature (delta, °C)				Precipitation (mult., unitless)				CO_2(ppm)
**Scenario**	DJF	MAM	JJA	SON	DJF	MAM	JJA	SON	
**Hist**	1.492	13.364	17.064	5.321	37.975	58.185	57.050	49.165	353.805
**RCP2.6**	1.475	0.940	1.338	1.196	1.141	1.050	0.963	1.042	425.891
**RCP4.5**	1.386	0.883	1.735	1.567	1.102	1.194	0.916	1.125	534.288
**RCP6.0**	2.716	1.309	2.297	2.435	1.194	1.136	0.934	1.061	636.153
**RCP8.5**	3.308	2.485	3.764	3.216	1.185	1.029	0.953	1.050	849.615

Tab. 5: Temperature anomalies (delta, °C), precipitation anomalies (multipliers, unitless) and CO_2 concentration for the site of Soroe (DK). The anomalies are calculated from the seasonal means for the period 2081-2100 and the seasonal means for the reference period 1981-2000. The absolute values for the reference period are given in the first line of the table (temperature is in °C and precipitation in mm/month). The CO_2 concentration is the mean value for years 1981-2000 or 2081-2100.

	Temperature (delta, °C)				Precipitation (mult., unitless)				CO_2(ppm)
**Scenario**	DJF	MAM	JJA	SON	DJF	MAM	JJA	SON	
**Hist**	0.954	10.652	15.364	5.689	39.184	44.014	58.631	60.628	353.805
**RCP2.6**	1.430	1.043	1.015	1.220	1.067	1.049	1.058	1.089	425.891
**RCP4.5**	1.729	1.208	1.295	1.724	1.145	1.147	1.052	1.087	534.288
**RCP6.0**	2.858	1.668	1.897	2.553	1.222	1.101	1.016	1.139	636.153
**RCP8.5**	3.555	2.918	2.855	3.334	1.182	1.252	1.061	1.208	849.615

Tab. 6: Temperature anomalies (delta, °C), precipitation anomalies (multipliers, unitless) and CO_2 concentration for the site of Vielsalm (BE). The anomalies are calculated from the seasonal means for the period 2081-2100 and the seasonal means for the reference period 1981-2000. The absolute values for the reference period are given in the first line of the table (temperature is in °C and precipitation in mm/month). The CO_2 concentration is the mean value for years 1981-2000 or 2081-2100.

	Temperature (delta, °C)				Precipitation (mult., unitless)				CO_2(ppm)
**Scenario**	DJF	MAM	JJA	SON	DJF	MAM	JJA	SON	
**Hist**	2.290	11.555	15.467	5.537	88.314	78.288	82.867	100.266	353.805
**RCP2.6**	1.137	0.591	0.627	0.894	1.117	1.017	1.059	1.158	425.891
**RCP4.5**	1.238	0.649	1.155	1.320	1.067	1.057	0.920	1.156	534.288
**RCP6.0**	2.201	1.295	2.467	2.110	1.182	0.979	0.848	1.149	636.153
**RCP8.5**	2.486	2.050	3.577	2.498	1.166	1.033	0.815	1.156	849.615

3. Simulation results from the Ecosystem Demography (ED) model

Chonggang Xu

Los Alamos National Laboratory, Los Alamos, NM 87544, USA

1. Methods

1.1 Forest model

We used the Ecosystem Demography (ED) model (Moorcroft *et al*., 2001) with modifications described by Fisher *et al*. (2010), McDowell et al (2013) and Xu et al (2013) to explore the drought-induced mortality. The model tracks the cohort of trees defined as a group of trees with similar size and the same species or plant functional types (PFTs). It also tracks forest successional dynamics by tracking the successional stages (i.e., time since last disturbance). For this application, regeneration processes were turned off in our simulations because we focused on the mortality patterns. For carbon dynamics, the model simulates photosynthesis based on Farquhar model (Farquhar et al 1980), which is then fed into carbon storage pools for different tissues. For the water dynamics, the model simulates water storage pools of cohorts for individual leaf layers, the stem section with leaf attached, and the combined component of stem section with no leaf attached and roots (Fig. 1). The plants and calculates the xylem water potential changes based on the relative water content in xylem (Barnard et al 2011). For each short time step (~10 minutes, but it is dynamically adjusted depending on the water fluxes among pools), the model solves leaf water potential by equalizing the water demand as determined by the Ball-Berry stomatal conductance model (Ball et al 1987) to the water supply from the leaf water storage pool and the water storage pool for the upper section of the xylem with leaves. The water storage pool is recharged by the root water uptake as determined by the pressure gradient from soil to xylem and the xylem conductance. During the drought, the plant halts photosynthesis if the leaf water potential is lower than minimum leaf water potential with no water recharge from the soil and the water storage will decline due to the loss of water by cuticular transpiration and the root water loss to the soil. This ED version uses a single soil layer. The soil water potential (ψ_s) is simulated based on an empirical equation of soil water content and soil texture (Niu and Yang 2006).

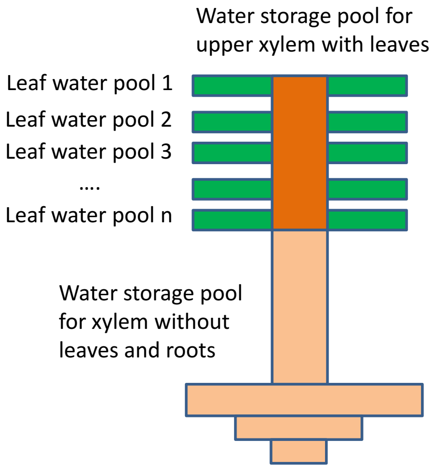

Figure 1 Illustration of water storage pools in the version of ED(X) used in this study

1.2 MORTALITY FORMULATIONS

We considered three process-based mortality algorithms: carbon starvation, hydraulic failure and phloem failure. For comparison, we also consider one traditional mortality algorithm based on growth efficiency, which are commonly used in forest model and earth system models. The carbon starvation algorithm assumes that, during drought, plant will use non-structural carbon pool to maintain their metabolic functions (McDowell et al 2011). When this storage pool becomes too low with prolonged drought, the plant will have a high risk of mortality because it is not able to maintain its metabolic functions or defend against pathogen and insect attacks. The carbon starvation is incorporated in ED(X) by Fisher et al (2010). In this version of ED(X), we improved the storage pool so that it will not become negative by incorporation of water stress impact on growth, resorption of carbon storage with tissue turnover and down-regulation of maintenance respiration depending on the size of storage pool. For comparison with data, it is allocated to different tissues by assuming equilibrium carbon storage concentration ratios among tissues. The mortality rate due to carbon starvation ($Mort_c$) is calculated as follows,

$$Mort_c = Mort_{c0} \frac{(LCSC_{crit} - LCSC \quad)}{LCSC_{crit}} \qquad (1)$$

where $LCSC$ is the concentration of non-structural carbon storage in the leaf (g storage C/g leaf C) and $LCSC_{crit}$ is the critical concentration that leads to risk of mortality. $Mort_{c0}$ is the baseline mortality probability associated with carbon deficit.

The hydraulic failure mortality algorithm assumes that the reduced water content in xylem during drought can lead to cavitation (forming of bubbles in xylem conduits), which impairs the xylem conductivity (Sperry 2000). If the amount of cavitation passes a critical threshold, xylem becomes dysfunctional and hydraulic failure ensues. The proportion loss of conductivity (PLC) due to cavitation is calculated based on the xylem water potential using the Weibull equation as follows (Neufeld et al., 1992):

$$PLC = 1.0 - e^{-(\psi_x / \Phi_{73})^c}, \qquad (2)$$

where Φ_{73} is the critical soil water potential that cause 73% loss of xylem conductivity and *c* is the shape parameter for conductivity loss. Different parts of stem could be subjective to hydraulic failure. In this study, we use the upper xylem part as an indicator of plant xylem cavitation, as our simulations does not result in large differences in the simulation results among the two parts of xylem. The mortality rate ($Mort_h$) due to hydraulic failure is calculated as follows:

$$Mort_h = Mort_{h0} \frac{(PLC \quad - PLC_{crit})}{1.0 - PLC_{crit}} \tag{3}$$

where PLC_{crit} is the critical proportion loss of conductivity that leads to risk of mortality. $Mort_{h0}$ is the baseline mortality probability associated with hydraulic failure.

The phloem failure mortality algorithm assumes that, during drought, xylem pressure become so negative that the plant is not able to maintain the turgor in phloem as a result of the water loss from phloem to xylem (Sevanto et al 2014). To simulate phloem failure induced mortality, the model first calculates the osmotic pressure (Ψ_p; Mpa) in the phloem based on the sugar content in leaves (Sevanto et al 2014). Then, the pressure deficit (Ψ_d; MPa) between upper section of xylem with leaf attachment and phloem are calculated as follows,

$$\Psi_d = \Psi_p - \Psi_{x1} \tag{4}$$

where Ψ_{x1} is the xylem water potential (MPa) in upper section of xylem with leaf attachment. The mortality rate ($Mort_h$) due to hydraulic failure is calculated as follows:

$$Mort_p = Mort_{p0} \frac{(\Psi_d - \Psi_{d,crit})}{\Psi_{d,max}} \tag{5}$$

where $\Psi_{d,crit}$ is the critical pressure deficit that leads to risk of mortality. $Mort_{p0}$ is the baseline mortality probability associated with phloem failure. $\Psi_{d,max}$ is the maximum pressure deficit (MPa) beyond $\Psi_{d,crit}$ that leads to the baseline mortality rate.

The growth efficiency algorithm assumes that trees have a high risk of mortality when it grows relatively slow (Sitch et al 2003). In this study, we use a similar mortality formulation to calculate growth efficiency based mortality rate ($Mort_g$) as other three process-based algorithms,

$$Mort_g = Mort_{g0} \frac{(NPP_{crit} - NPP)}{NPP_{crit}}, \tag{6}$$

where NPP is the annual net primary production per unit of leaf area (g C/m^2 leaf). NPP_{crit} is the critical NPP that leads to risk of mortality. $Mort_{g0}$ is the baseline mortality rate per year associated with growth efficiency.

2. SIMULATION SETTINGS

2.1 *SITE DATA*

We evaluated our model using a drought manipulation experiment site with a ~45% rain exclusions, located in the Los Pinos mountains within the Sevilleta Long-Term Ecological Research site and Sevilleta National Wildlife Refuge, Socorro County, New Mexico (N 34 º 23’11”, W 106 º31’46”). Site elevation is 1911 m, and piñon pine and juniper are the dominant woody species at the site. Study plots were located on 8-18% slopes on calcid aridisols. Climate records from a meteorological station located within 2.2 km indicate a mean annual temperature (20-yr) of 12.7 ˚C, with a mean July maximum of 31.0 ˚C, mean December minimum of -3.3 ˚C, and mean annual precipitation total of 362.7 mm yr^{-1}. Roughly half of the annual precipitation is driven by the North American Monsoon during July-September.

The data used to evaluate the ED(X) model included near monthly measurements of pre-dawn leaf water potential (φ_{pd}), daily measurements of soil water potential (φ_s) at 20 cm, half-hourly or daily

E, half-hourly meteorological data (solar radiation, air and soil temperature, and precipitation) as well as standard metrics of stand density, tree size, biomass, leaf area, and soil properties (Pangle et al. 2012, Plaut et al. 2012). Meteorological data was collected on site to drive the model simulations. Leaf samples were collected throughout the duration of this study and analyzed for *NSC.* Additional site, experimental infrastructure, and measurement details are reported in Pangle et al. (2012) and Plaut et al. (2012).

2.2 *Simulation experiments*

Parameter estimation

To simplify the mortality comparison, we only consider piñon pine in this study. We first tune key model parameters to fit the observations on the ambient site (Tab. 1). The model was able to reasonably capture the observed pattern of soil water potential, leaf predawn water potential, leaf non-structural carbohydrate and leaf transpirations (Fig. 1). The amount of variation that was not accounted for by the model could result from the natural variability across landscape for individual trees and missing mechanisms such as carbon storage transportation, water redistribution across multiple soil layers. Then, for the estimation of mortality parameters, we tune the threshold value (i.e., $LCSC_{crit}$ for carbon starvation algorithm, PLC_{crit} for hydraulic failure algorithm, $\Psi_{d,crit}$ for phloem failure algorithm, and NPP_{crit} for growth efficiency algorithm) so that the mortality for the ambient site is close to zero (Fig 2a). For the baseline mortality rate (i.e., $Mort_{c0}$ for carbon starvation, $Mort_{h0}$ for hydraulic failure, $Mort_{p0}$ for phloem failure and $Mort_{g0}$ for growth efficiency), it is tuned so that the simulated 2010 survival of piñon pines is close to observations (Fig 2b).

Table 1: Estimated mortality parameters

Parameters	Descriptions	Value in this study
$\boldsymbol{LCSC_{crit}}$	Critical leaf nonstructural carbon concentration (g NSC/g leaf c) that leads to risk of mortality	0.045
$\boldsymbol{PLC_{crit}}$	Critical proportion loss of conductivity that leads to risk of mortality	0.65
$\Psi_{d,crit}$	critical pressure deficit (MPa) that leads to risk of mortality	2.5
$\boldsymbol{NPP_{crit}}$	critical NPP (g C/m^2 leaf/year) that leads to risk of mortality	60
$\boldsymbol{Mort_{c0}}$	baseline mortality probability associated with carbon deficit per day	0.008
$\boldsymbol{Mort_{h0}}$	baseline mortality probability per day associated with hydraulic failure	0.5
$\boldsymbol{Mort_{p0}}$	the baseline mortality probability associated with phloem failure per day	0.05
$\boldsymbol{Mort_{g0}}$	baseline mortality rate per year associated with growth efficiency	0.55

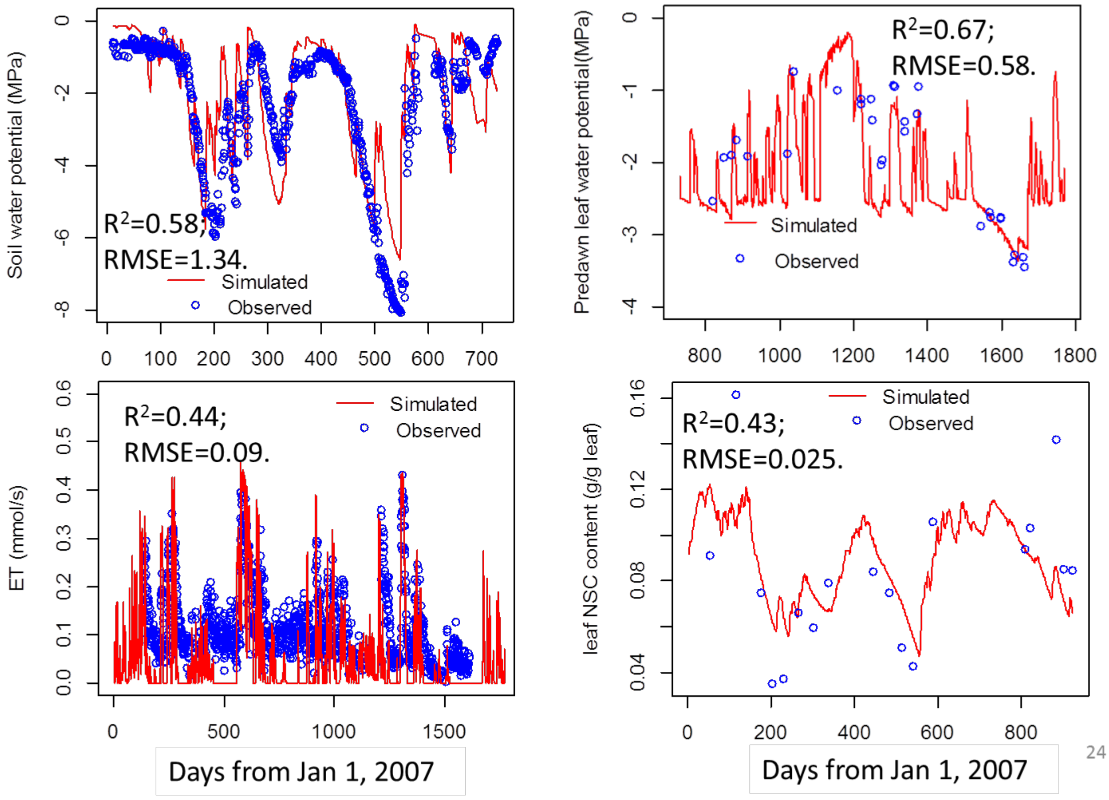

Figure 1 ED(X) Model simulations against data at ambient conditions at the Sevilleta site in New Mexico, USA.

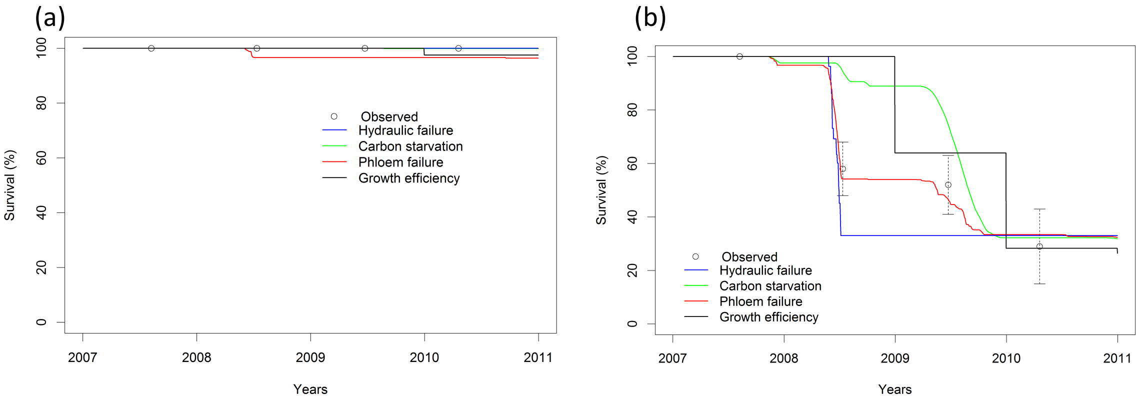

Figure 2 Simulated survival of pinion pines compared to observations for ambient plots (a) and precipitation exclusion plots (b).

Climate scenario

We consider the RCP 8.5 climate predictions by CESM in CMIP5 for this site from year 2010-2100. The range of the climate change from year 2071-2100 compared to the site level climate data from 2006-2010 are given in Tab. 2.

Table 2: Potential climate change for our study site

Scenario	Temperature(°C)				Precipitation (mm)				CO_2
	DJF	MAM	JJA	SON	DJF	MAM	JJA	SON	
**RCP 8.5**	+2.24	+2.27	+4.01	+2.67	-11.98	-9.57	-33.94	+9.27	+470ppm

3. Results

Our simulations suggest that the difference in the predicted survival of piñon pine among the four considered mortality algorithms increased through time (Fig. 3). At the beginning of the simulation before year 2055, hydraulic failure and carbon starvation predicts higher survival compared to growth efficiency and phloem failure. After that, we see a large increase in predicted mortality for hydraulic failure algorithm. There is almost no changes for the algorithms based on carbon starvation and growth efficiency, which may result from the beneficial impact of CO_2 fertilization on these two algorithms. Because the phloem failure algorithm depends on both carbon storage and hydraulic stress, it gives the intermediate estimation of survival.

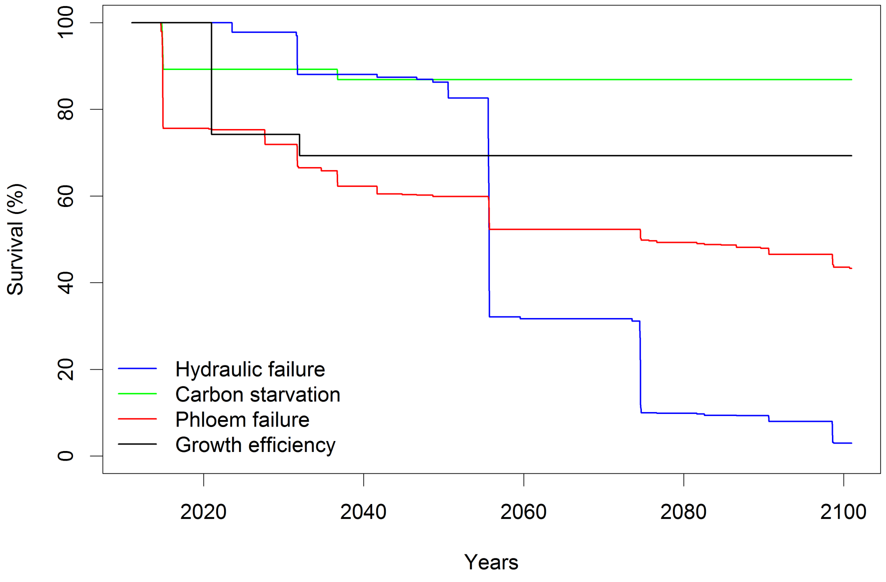

Figure 3: Estimated survival of pinion pines under RCP8.5 for different mortality algorithms

4. References

J. T. Ball, I. E. Woodrow, J. A. Berry, in *Progress in Photosynthesis Research,* J. Biggins, Ed. (Martinus Nijhoff Publishers, Netherlands), vol. 4, pp. 221–224. (1987)

D. M. Barnard *et al.*, Climate-related trends in sapwood biophysical properties in two conifers: avoidance of hydraulic dysfunction through coordinated adjustments in xylem efficiency, safety and capacitance. *Plant Cell Environ* **34**, 643 (2011).

G. D. Farquhar, S. von Caemmerer, J. A. Berry, A biochemical model of photosynthetic CO2 assimilation in leaves of C3 species. Planta 149, 78 (1980).

N. G. McDowell, Mechanisms of vegetation mortality. Plant Physiol 155, 1051 (2011).

H. S. Neufeld *et al.*, Genotypic variability in vulnerability of leaf xylem to cavitation in water-stressed and well-irrigated sugarcane. *Plant Physiol* **100**, 1020 (1992).

G.-Y. Niu, Z.-L. Yang, Effects of Frozen Soil on Snowmelt Runoff and Soil Water Storage at a Continental Scale. *J Hydrometeorol* **7**, 937 (2006).

J. S. Sperry, F. R. Adler, G. S. Campbell, J. P. Comstock, Limitation of plant water use by rhizosphere and xylem conductance: results from a model. *Plant Cell Environ* **21**, 347 (1998).

Sperry, J. S. "Hydraulic constraints on plant gas exchange."(2000), Agric For Meteorol 104, 13-23.

F. Tardieu, T. Simonneau, Variability among species of stomatal control under fluctuating soil water status and evaporative demand: modelling isohydric and anisohydric behaviours. *J Exp Bot* **49**, 419 (Mar, 1998).

S. Sevanto, N. G. McDowell, L. T. Dickman, R. Pangle, W. T. Pockman, How do trees die? A test of the hydraulic failure and carbon starvation hypotheses. Plant, Cell & Environment 37, 153 (2014).

S. Sitch *et al.*, Evaluation of ecosystem dynamics, plant geography and terrestrial carbon cycling in the LPJ dynamic global vegetation model. *Glob Change Biol* **9**, 161 (2003).

C. Xu, N. G. McDowell, S. Sevanto, R. A. Fisher, Our limited ability to predict vegetation dynamics under water stress. New Phytol 200, 298 (2013).

4. SIMULATION RESULTS FROM FORCLIM V3.0

Maxime Cailleret[1,2], Nicolas Bircher[1] & Harald Bugmann[1]

1) Forest Ecology, ETH Zürich, 8092 Zürich, Switzerland

2) Research Unit Forest Dynamics, Swiss Federal Institute for Forest, Snow and Landscape Research - WSL, Zürcherstrasse 111, 8903 Birmensdorf, Switzerland

1. METHODS

1.1 FOREST MODEL

ForClim is a cohort-based forest gap model that was developed to analyze successional pathways of various forest types in Central Europe (Bugmann 1996) and other parts of the temperate zone (Bugmann and Solomon 2000; Shao et al. 2001). The model simulates establishment, growth and mortality of tree cohorts with an annual time step based on species characteristics (e.g., shade and drought tolerance) and on environmental factors.. Saplings are established with a predefined diameter at breast height (dbh) of 1.27 cm, provided that a range of biotic and abiotic factors are within species-specific thresholds (Bugmann 1996). Radial tree growth is modeled based on the carbon budget by Moore (1989), with several modifications (Rasche et al. 2012). Species-specific optimal growth is reduced by crown characteristics of the cohort, and by several environmental factors, including light availability, warmth (degree-day sum) and drought (soil moisture) during the growing season, and nutrient availability (plant-available nitrogen). Note that ForClim does not include any carbon or nutrient storage pools and thus is lacking temporal autocorrelation in simulated tree growth.

1.2 MORTALITY FORMULATIONS

Standard formulation (essentially based on Solomon 1986)

The mortality probability for trees of cohort *c* and species *s* is modeled by two functions: A 'background' component that provides a constant, species-specific mortality rate that is derived from the putative maximum age of each species (gPAge$_c$):

$$gP\mathrm{Age}_c = \frac{k\mathrm{Death}P}{k\mathrm{AMax}_s}$$

where kDeathP is a mortality coefficient (4.605 by default) and kAMax$_s$ is the species-specific maximum age (e.g., 930 years for Norway spruce; Bugmann 1994). This corresponds to the assumption that 1% of a tree population will survive to kAMax$_s$. Additionally, a stress-induced mortality (gPStr$_c$) is included: if diameter increment falls below 10% of its maximum or below 3 mm (i.e., slow growth) for more than two consecutive years (kSGrT = 2), the annual mortality probability is augmented by 0.368 (kSlowGrP):

$$gP\mathrm{Str}_c = \begin{cases} k\mathrm{SlowGr}P & \mathrm{SGr}_c > k\mathrm{SGr}T \\ 0 & \mathrm{else} \end{cases}$$

where SGr_c denotes the number of consecutive years a cohort has experienced slow growth. This corresponds to the assumption that slow growth leads to a 99% die-off within 10 years for all affected cohorts. The overall mortality probability $gPMort_c$ is calculated for each cohort using Monte Carlo techniques:

$$gP\mathrm{Mort}_c = gP\mathrm{Age}_c + [1 - gP\mathrm{Age}_c]gP\mathrm{Str}_c$$

While establishment and growth are modeled at the cohort level, mortality is applied to each single tree of a cohort. For all the trees within the cohort, a random number generator is used to determine whether a tree dies (i.e., a tree dies if a uniform random number [0…1] is below $gPMort_c$).

Alternative formulations: empirical mortality models

A) Inventory-based mortality function (IM): The IM function was derived using single-tree data from plots of the Swiss National Forest Inventory (NFI) that had not experienced forest management for at least 50 years (Wunder and Abegg in prep.). The callipering threshold was 12 cm, and the interval between inventories was 11 years. IM was formulated as a logistic regression model where the survival probability depends on tree size, growth rate, shade tolerance and the degree-day sum:

$$\begin{aligned}\Pr(Y_{i,t} = 1\,|\,X_{i,t}) = 1/(1 + \exp[&\alpha_0 + \alpha_1 \times \mathrm{dbh}_{i,t} + \alpha_2 \times \mathrm{dbh}^2_{i,t} \\ &+ \mathrm{relbai}_{i,t,j} + \alpha_3 \times \mathrm{DD}_t \\ &+ \mathrm{shadeTol}_{ik}]^{-1}) \qquad (4)\end{aligned}$$

where $\Pr(Y_{i,t} = 1\ |X_{i,t})$ is the probability of tree *i* at year *t* to be still alive in 11 years; DD is the logarithm of the annual degree-day sum. The estimate of the *relbai* variable (relative basal area increment) changes according to the class *j* of growth rates (four classes), whereas estimates of the shadeTol variable change among three classes *k* of species-specific shade tolerance ('high', 'intermediate' and 'low'; cf. Bugmann 1994).

Since ForClim has an annual time step, $\Pr(Y_{i,t} = 1\ |X_{i,t})$ was scaled to an annual survival probability:

$$gP\mathrm{Surv}_{i,t} = 1 - (1 - \Pr(Y_{i,t} = 1\,|\,X_{i,t}))^{1/11}$$

B) Tree-ring-based mortality function (TRM): The TRM function was taken from Bigler and Bugmann (2004), who cored pairs of dead and living Norway spruce with a minimum dbh of 10 cm at three sites in the Swiss Alps. They used variable combinations of three different categories – absolute growth level, relative growth level, and growth trend – to fit logistic regression models of the annual probability of tree survival. For the present study, we used the model that showed the best goodness-of-fit in Bigler and Bugmann (2004):

$$\begin{aligned}gP\mathrm{Surv}_{i,t} = 1/(1 + \exp[&\beta_0 + \beta_1 \times \mathrm{locreg}_{5,i,t} + \beta_2 \\ &\times \log(\mathrm{BAI}_{3,i,t}) + \beta \\ &\times \log(\mathrm{relbai}_{i,t})]^{-1})\end{aligned}$$

where locreg denotes the slope of a local linear regression over 5 years of annual basal area increment, $\log(BAI_3)$ is the natural logarithm of the average basal area increment of the last 3 years, and log(relbai) is the natural logarithm of relative basal area increment of the last year.

Combining empirically-based mortality with 'background' mortality

Due to the suspicion that particularly the inventory-based mortality model was leading to mortality rates that were very low, particularly for medium-sized trees, we combined the empirically-based approaches with the 'background' mortality of the theoretical formulation by adding the 'background' mortality rate $gPAge_c$ = 0.00495 yr^{-1} to the intercept of the empirical formulations (cf. Eq. 1).

Using each empirical mortality function (TRM; IM) as such and combining it with a 'background' mortality (TRM_bg; IM_bg) resulted in four new ForClim versions in addition to the standard version (3.0). These mortality functions were applied to all trees in ForClim regardless of their size (i.e, down to newly established saplings with a dbh of 1.27 cm) even though the data for model calibration did not include trees with a dbh <10 cm (TRM) or <12 cm (IM).

2. SIMULATION SETTINGS

2.1 *SITE DATA*

Two sites were used to conduct simulation studies, both located in the Central Alps of Switzerland, i.e. the managed stand of Sigriswil and the unmanaged, primeval forest of Scatlè. Due to the restriction of the TRM function to Norway spruce, only mono-specific stands of that species could be selected for the present study.

Monthly mean temperature and precipitation sum for 1930–2010 were provided by the Land Use Dynamics Research Group at WSL. These data had been derived by a spatial interpolation of data from the MeteoSwiss network using DAYMET (Thornton et al. 1997) to a grid with cell size of 1 ha. For obtaining long-term temperature and precipitation means, we followed the approach of Rasche et al. (2011), i.e. we used the data series of the cell covering the center of the forest stand and its eight closest neighbors. Averages, standard deviations and cross-correlations of monthly temperature and precipitation were derived by aggregating the daily climate data from the different cells and averaging the resulting data series. Further site parameters include nitrogen availability [$kg \cdot ha^{-1} \cdot yr^{-1}$], maximum soil water holding capacity [cm] and slope/aspect; they were estimated from site descriptions.

2.2 *TEST DATA*

Sigriswil is a site of the Growth-And-Yield research network of the Swiss Federal Institute for Forest, Snow and Landscape Research (WSL). It consists of an uneven-aged selection forest where management was conducted on a regular basis (Wehrli et al. 2005). Since 1925, harvest data (i.e., year of intervention, tree removal, and targeted species) including a follow-up inventory of the stand have been recorded ten times until 1997. The callipering threshold for the inventories was 7.5 cm.

Scatlè is a strictly protected forest reserve and one of the few relicts of primeval coniferous forests in Central Europe. There are no records or on-site evidence of human disturbance (i.e., management) for the last several centuries (Brang et al. 2011). Data from four forest inventories were available between 1965 and 2006. Living and standing dead trees had been surveyed with a callipering threshold of 8 cm in the first inventory, and 4 cm thereafter.

Neither of the two sites had been used in any sort of model parameterization or calibration effort, i.e. these data are truly independent of the model.

2.2 *SIMULATION EXPERIMENTS*

Simulations were run for two time periods: "short-term simulations" under current climate for a comparison against the historical time series (inventory data), and "long-term simulations" into the

future including climate change scenarios. Only Norway spruce was allowed to establish, and no other tree species were initialized (cf. explanation above).

For the short-term simulations, the models were initialized with the single-tree data (species, dbh) of the first inventory conducted at the study sites (years 1925 and 1965 in Sigriswil and Scatlè, respectively). In contrast to Scatlè, where no management was applied, we implemented an uneven-aged ('plentering') regime at Sigriswil.

Simulations into the future were run for the natural forest reserve of Scatlè only. The five models were initialized with the single-tree data of the last inventory (2006) and run until the year 2400. Climate change was assumed to take place between 2010 and 2085, employing a linear trend between the current and future climate while the climate was assumed to be stable afterwards. We applied the delta change method on the current climate using delta values from the CH2011 report (CH2011 2011) for northeastern Switzerland (CHNE) (Table 1). Simulations were run for an RCP3PD scenario, which is based on the assumption of a *global* average temperature increase of about 2 °C ("2° scenario"), and for an A1B scenario. As a baseline, a simulation was also run for the period 2006-2400 using current climatic conditions.

Table 1: Climate change scenarios (RCP3PD and A1B) for northeastern Switzerland according to the CH2011 report. Upper estimates (i.e., the 97.5th percentile) for temperature and lower estimates (i.e., the 2.5th percentile) for precipitation were used. Compared to the reference climate, absolute changes (differences; between 2010 and 2085) are given for temperatures while changes in precipitation are relative (factors). Standard deviations and the cross-correlations between monthly temperature and precipitation values were assumed to stay constant during climate change

	RCP3PD		A1B	
**Season**	T (°C)	P (%)	T (°C)	P (%)
**Spring**	+1.67	*0.935	+3.69	*0.936
**Summer**	+2.20	*0.853	+4.84	*0.713
**Fall**	+1.89	*0.863	+4.29	*0.824
**Winter**	+2.15	*0.896	+4.22	*0.891

3. SIMULATION RESULTS

Current climate

In Sigriswil, simulated basal area and stem numbers of all model versions matched measured data quite well, although simulated BAI was underestimated compared to the empirical data, especially after 1950 (Fig. 1). This underestimation in spruce annual growth, especially after harvesting, was typical of ForClim 3.0 because of the overestimation of the effect of tree crown size on its growth (see Bircher et al. 2015 and Mina et al. 2017). Stem numbers were consistently underestimated as well up after this point, which is possibly related to the initialization data. In the empirical data, trees with a dbh <7.5 cm were not included due to the callipering threshold, and hence the trees that were initially present in the lower size classes in reality were not simulated in the model. As expected, the two versions with additional background mortality (TRM_bg and IM_bg) predicted lower BA than the versions without mortality (TRM, IM), and no strong differences could be observed between the ForClim versions with the theoretical mortality function (3.0) and with the empirical ones.

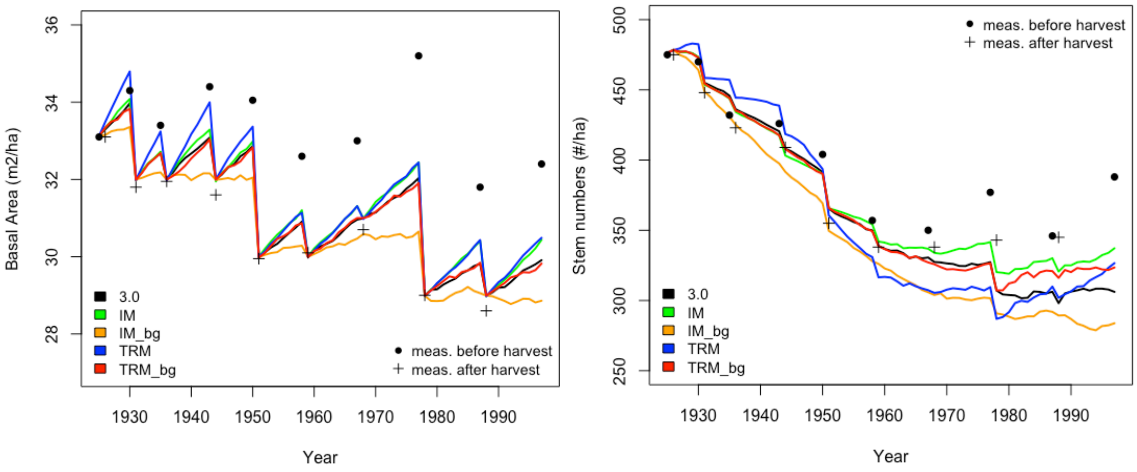

Figure 1: Observed and simulated mean basal area (left) and stem numbers (right) for Sigriswil. Years on the x-axis indicate time points when management and inventories were conducted. For better visualization, standard deviations of simulated basal area are not shown

In Scatlè, stem numbers was also underestimated by all model versions; especially the ones including background mortality (IM_bg and TRM_bg), while simulated BA seems to better match measured data (Fig. 2). For the period 1965-1977, all versions underestimated the increase in stem numbers and especially BAI, such as in Sigriswil. From 1977 to 1989, a sharp decline in basal area and stem numbers occurred in the empirical data, mainly due to avalanche-induced mortality in winter 1984 (Brang et al. 2011). The avalanches impacted mainly trees with a dbh between 18 and 38 cm. As natural disturbances are not considered in ForClim, these effects could not be reproduced in the simulation. Instead, a steady decrease in stem numbers was simulated in all versions while basal area increased slightly or was constant. For the period 1989 to 2006, forest dynamics in Scatlè were characterized by an enhanced phase of regeneration, visible from the strong increase in stem numbers of trees with a dbh ≤16 cm in the empirical data, which most likely was an indirect consequence of the avalanches, and thus could not be predicted by the model.

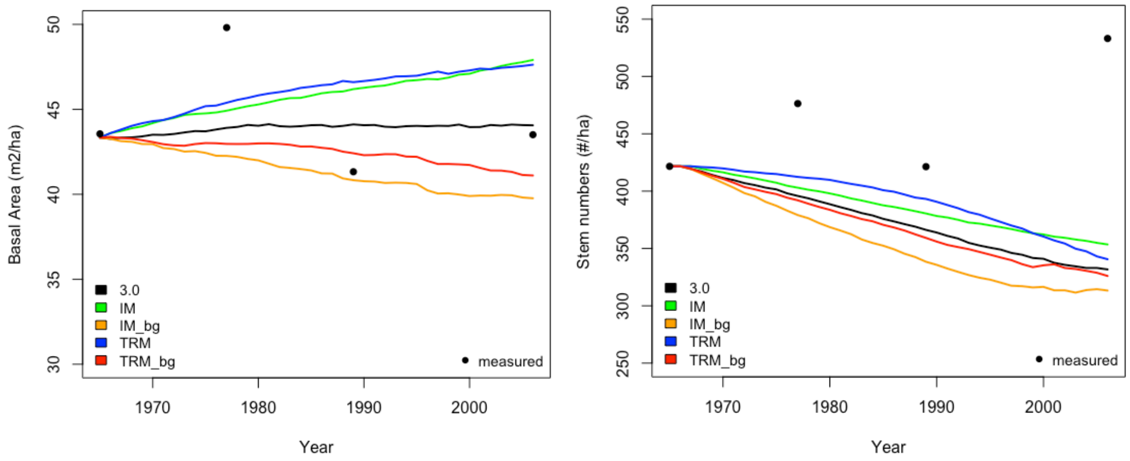

Figure 2: Observed and simulated mean basal area (left) and stem numbers (right) for Scatlè. For better visualization, standard deviations of simulated basal area are not shown

Extrapolations into the future

In contrast to the short-term simulations, simulations over 400 years under current climatic conditions revealed distinct differences between the five model versions (Fig. 3). ForClim v3.0 showed a

weak increase of basal area up to a maximum of 44.6 $m^2 \cdot ha^{-1}$ in 2046, followed by a slow but steady decrease until the end of the simulation period. Similarly, the model versions without additional background mortality also predicted an initial increase in basal area, but reaching different maximum values (52.6 and 47.8$m^2 \cdot ha^{-1}$) at different points in time (years 2167 and 2066) for the IM and TRM model versions, respectively. The versions with additional background mortality showed a gradual and similar decrease of BA over time.

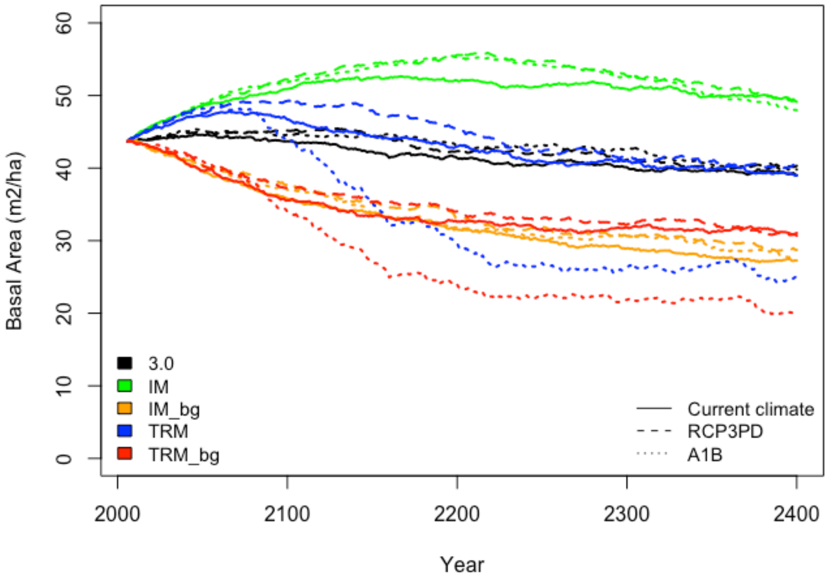

Fig. 3: Simulated basal area for the period of 2006-2400 for Scatlè under current climate (solid lines) and two climate change scenarios (RCP3PD scenario [“moderate”]: dashed lines; A1B scenario [“strong”]: dotted lines) for the five model variants (colors)

In the simulations under a climate change scenario (Fig. 3), two growth-limiting factors were directly but differently modified: the degree-day and the soil moisture growth factors, with strong impacts on the simulated forest dynamics:

Under moderate climate change (scenario RCP3PD), the degree-day growth factor increased by 40%, whereas the soil moisture growth factor was marginally reduced (<1%), essentially leading to better growth of Norway spruce compared to the growth simulated under current climate. However, changes in basal area were hardly significant for ForClim v3.0 (paired t test; $p>0.05$). For the other model versions, the differences were partly significant, but amounted to a few square meters per hectare only.

Under stronger climate change (A1B), simulated growing conditions for Norway spruce were improved as well. Compared to current climate, the degree-day growth factor increased by 70% while the growth reduction due to drier conditions (i.e., reduced soil moisture) amounted to 5%. Still, compared to the RCP3PD scenario, ForClim v3.0, IM and IM_bg did not show notable differences in basal area. The patterns of basal area predicted by TRM and TRM_bg were in stark contrast to those of the other model versions and dropped from 49 $m^2 \cdot ha^{-1}$ and 53 $m^2 \cdot ha^{-1}$ to around 24 $m^2 \cdot ha^{-1}$ and 20 $m^2 \cdot ha^{-1}$ (in 2388), respectively.

A more detailed description of most of these simulations (except the ones with additional background mortality) can be found in Bircher et al. (2015).

4. References

Bigler, C. & Bugmann, H., 2004. Predicting the time of tree death using dendrochronological data. *Ecol. Appl.* **14**: 902-914.

Bircher, N., Cailleret, M. & Bugmann, H., 2015. The agony of choice: different empirical mortality models lead to sharply different future forest dynamics. *Ecol. Appl.* **25**: 1303-1318.

Brang, P., Bugmann, H. & Heiri, C., 2011. *Waldreservate. 50 Jahre natürliche Waldentwicklung in der Schweiz.* Birmensdorf, Eidg. Forschungsanstalt WSL; Zürich, ETH Zürich. Bern, Stuttgart, Wien, Haupt.

Bugmann, H., 1994. On the ecology of mountainous forests in a changing climate: A simulation study. Ph.D. Thesis no. 10638, ETH Zurich, Switzerland, 258 pp.

Bugmann, H., 1996. A simplified forest model to study species composition along climate gradients. *Ecology* **77**: 2055-2074.

Bugmann, H.K.M. & Solomon, A.M., 2000. Explaining forest biomass and species composition across multiple biogeographical regions. *Ecol. Appl.* **10**: 95-114.

CH2011, 2011. *Swiss Climate Change Scenarios CH2011*. C2SM, MeteoSwiss, ETH, NCCR Climate and OcCC.

Moore, A.D., 1989. On the maximum growth equation used in forest gap simulation models. *Ecol. Modelling* **45**: 63-67.

Mina, M., Bugmann, H., Klopcic, M., & Cailleret, M. (2017). Accurate modeling of harvesting is key for projecting future forest dynamics: a case study in the Slovenian mountains. *Regional Environmental Change*, **17**: 49-64.

Rasche, L., Fahse, L., Zingg, A. & Bugmann, H., 2011. Getting a virtual forester fit for the challenge of climatic change. *J. Appl. Ecol.* **48**: 1174-1186.

Rasche, L., Fahse, L., Zingg, A. & Bugmann, H., 2012. Enhancing gap model accuracy by modeling dynamic height growth and dynamic maximum tree height. *Ecol. Model.* **232**: 133-143.

Shao, G.F., Bugmann, H. & Yan, X.D., 2001. A comparative analysis of the structure and behavior of three gap models at sites in northeastern China. *Clim. Change* **51**: 389-413.

Solomon, A.M., 1986. Transient response of forests to CO_2-induced climate change: simulation modeling experiments in eastern North America. *Oecologia* **68**: 567-579.

Thornton, P.E., Running, S.W. & White, M.A., 1997. Generating surfaces of daily meteorological variables over large regions of complex terrain. *J. Hydrol.* **190**: 214-251.

Wehrli, A., Zingg, A., Bugmann, H. & Huth, A., 2005. Using a forest patch model to predict the dynamics of stand structure in Swiss mountain forests. *For. Ecol. Manage.* **205**: 149-167.

Wunder, J. & Abegg, M., in prep. Modelling individual tree mortality for Swiss forest species.

5. SIMULATION RESULTS FROM FORMIND

Friedrich J. Bohn and Edna Rödig

Helmholtz Center for Environmental Research - UFZ

1. METHODS

1.1 FORMIND

The individual based forest growth model FORMIND is based on four processes: tree growth, competition between trees, establishment of new trees and tree mortality. For this study, we applied the forest model on temperate forest sites and used the parametrization of Bohn et al. 2014.

The growth of a tree results from its carbon balance including photosynthetic production and respiratory losses (Thornley and Johnson, 1990). Photosynthesis is driven by available light that reaches the forest canopy. Thereby, large trees shade smaller ones of the same forest patch. In addition, photosynthesis is limited by temperature and available soil water content (Fischer et al. 2014, Gutierrez et al. 2014). Respiratory costs are tree-size dependent and rise with increasing temperature (Bohn et al. 2014). Growth of a tree is attributed to aboveground components: the stem and the crown.

In addition to asymmetric competition for light, trees compete equally for space. Crowded stands get thinned out and mortality is increased if tree crowns overlap (Köhler et al., 2001).

Tree seedlings emerge from a seed pool which originates from a surrounding forest. A germinated tree seedling establishes successfully if space and light are sufficient.

For more details about the FORMIND model see www.formind.org

1.2 MORTALITY FORMULATIONS (FORMIND)

We tested five different mortality scenarios which differ in the vulnerability of trees due to an imbalance of sequestered carbon (photosynthesis) and required carbon (respiration):

1. **Base mortality (BM):** Constant base mortality m_b of 2 % (inventory). Trees are removed stochastically. If NPP is negative, no influence on mortality. Negative NPP needs to be "refilled" the next years.
2. **NPP_0**: No base mortality. If NPP is negative, trees die immediately (Sitch et al. 2003).
3. **$NPP_{\%}$** : No base mortality. If NPP is negative, trees die with a certain probability $m_{\%}$. This probability increases with decreasing negative NPP. We assume a mortality increase as follows:
$$m_{\%} = a * \left(\frac{NPP}{LAI}\right)^b, \qquad if\ NPP < 0\ (a = -1, b = 1)$$
4. **BM+NPP_0**: Combines base mortality scenario with NPP_0.
5. **BM+$NPP_{\%}$:** Combines base mortality scenario and $NPP_{\%}$.

2. Simulation settings

2.1 Site data

Site name: Peitz

Location: East Germany; 14.36° East; 51.95° North; Altitude: 50 m.

Vegetation data: Planted in 1900, even-aged monoculture of Scots Pine (*pinus sylvestris*) and soil vegetation. Inventory data are available from 1941-2011. Site area covers 1000m².

Soil data: Arenosol of 2 meter thickness with 87% sand in the upper layers and up to 98% sand in the low layers. The wilting point is 7.5 Vol% for upper layers and 9 Vol% for lower layers.

Model adaptations: We use a general parameterization for pine trees according to Bohn et al 2014. Soil parameters are adapted to the Peitz site if available. Missing soil parameters are taken from Maidment 1996 (fully saturated conductivity = 0.06e-3 $\mathrm{m\ s^{-1}}$; pores size distribution index = 0.55).

2.2 Simulation Experiments

We simulate an area of 5 ha for all possible combinations of climate scenarios and mortality scenarios. Each combination is simulated with and without regeneration. This results in 150 5 ha-simulations. Climate scenarios are displayed in Table 1.

Table 1: Absolut changes in temperature (K), relative changes in precipitation and absolut changes in radiation (W m-2) averaged over the five climate model drivers at the simulated sites between the reference period (1981-2010) and the last 30 years in the dataset (2070-2099) for the two scenarios in each season (DJF: December, January, February; MAM: March, April, May; JJA: June, July, August; SON: September, October, November)

	**RCP 2.6**				**RCP 8.5**			
	DJF	MAM	JJA	SON	DJF	MAM	JJA	SON
**Temp**	1.80	1.49	1.92	1.75	4.65	3.88	5.33	4.71
**Precip**	1.08	1.03	1.07	1.02	1.17	1.06	0.95	1.05
**Radiation**	1.05	8.78	10.13	6.12	0.00	7.07	13.39	7.18

3. Simulation results

Five different mortality scenarios were tested with (Fig. 1, blue lines) and without (Fig. 1, yellow lines) regeneration. We simulated without regeneration in order to get a better understanding on how the model reacts to the different mortality scenarios. Without regeneration in mortality scenario $\mathrm{NPP}_{\%}$, the stand does not die out. If a tree reaches its maximum height, growth stops. The stress due to climate change is not high enough to let the trees die out. In the other mortality scenarios, the forest stand dies out without regeneration due to the background mortality.

In general, the simulated data matches the inventory well. We found a rather weak effect of climate scenarios on simulated forest properties but a strong effect of the mortality scenarios (Fig. 1).

Except for $\mathrm{NPP}_{\%}$, we see a decline in biomass and basal area for the next century followed by a stabilization of stand biomass between 75 and 125 t odm and basal area of around 20 m^2ha^{-1}). Lower values are found for RCP 8.5 for all mortality scenarios. Tree numbers decrease in the beginning as

observed but regenerate in 2000-2500 for BM and BM&$NPP_{\%}$, which indicates the existence of many small trees. For NPP_0 and BM & $NPP_{\%}$, tree numbers stabilize around 750.

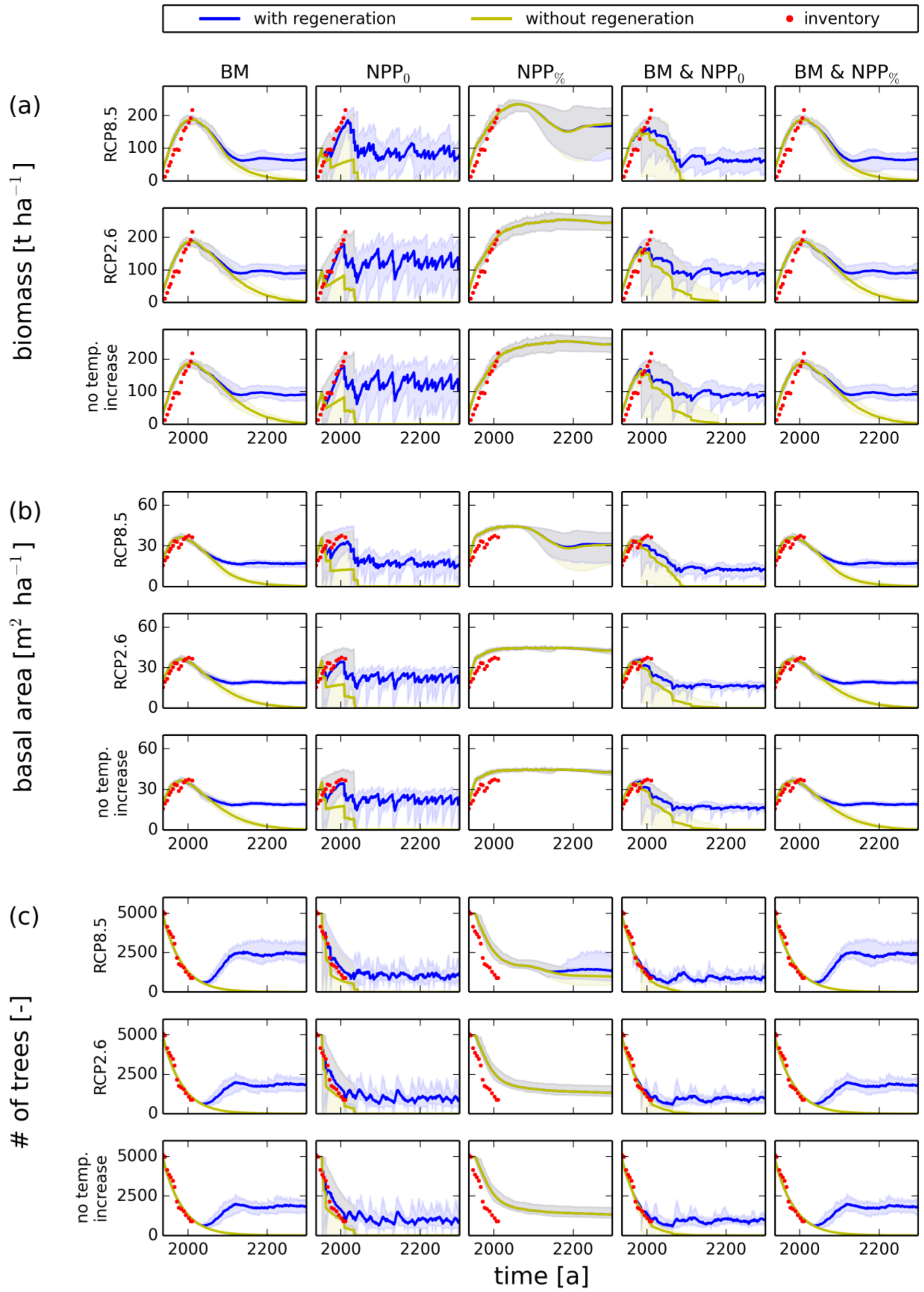

Fig. 1: Simulation output of (a) biomass, (b) basal area and (c) number of trees over time for the Peitz initialization (monoculture, pine forest) for different mortality scenarios (BM, NPP_0 , $NPP_{\%}$, BM &NPP_0 , BM &$NPP_{\%}$). The forest model is driven with different climate scenarios: no temperature change, the PCP2.6 sceneario and RCP8.5 scenario. The spread in simulation output results from the five different ensemble models and the stochasticity of mortality (range between 1 ha-plots)

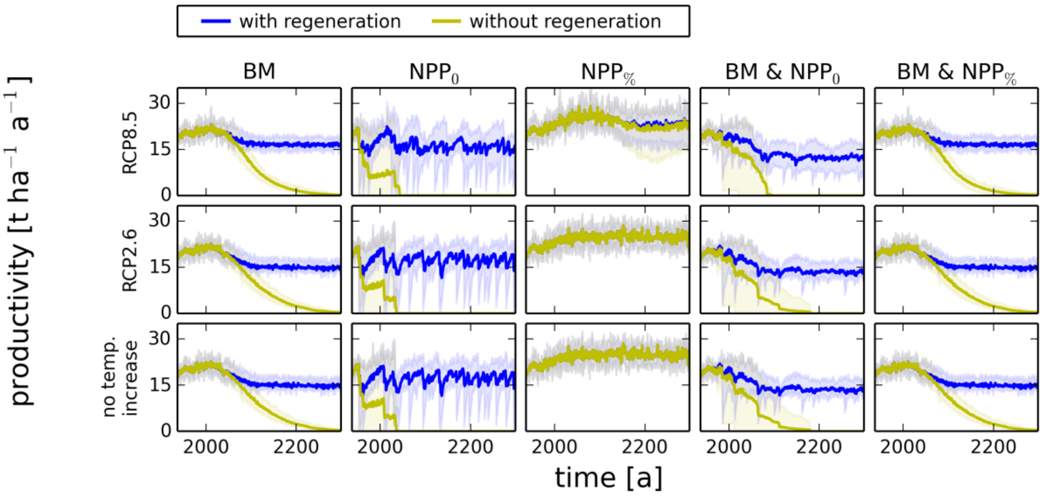

Fig. 2: Simulation output of productivity over time for the Peitz initialization (monoculture, pine forest) for different mortality scenarios (BM, NPP_0 , $NPP_{\%}$, BM &NPP_0 , BM &$NPP_{\%}$). The forest model is driven with different climate scenarios: no temperature change, the PCP2.6 sceneario and RCP8.5 scenario. The spread in simulation output results from the five different ensemble models and the stochasticity of mortality (range between 1 ha-plots)

4. References

Thornley, J.H.M. and Johnson, I.R., 1990. Plant and crop modelling: a mathematical approach to plant and crop physiology. Oxford University Press.

Bohn, F.J., Frank, K. and Huth, A. 2014. Of climate and its resulting tree growth: simulating the productivity of temperate forests. Ecological Modelling, 278: 9–17

Fischer,R., Armstrong, A., Shugart, H.H., and Huth, A. 2014. Simulating the impacts of reduced rainfall on carbon stocks and net ecosystem exchange in a tropical forest. Environmental Modelling & Software, 53: 200—206

Gutiérrez AG, Armesto JJ, Díaz MF, Huth A. 2012. Sensitivity of North Patagonian temperate rainforests to changes in rainfall regimes: a process-based, dynamic forest model. Biogeosciences Discussions, 9(6):6293-6333

Sitch, S., Smith, B., Prentice, I. C., Arneth, A., Bondeau, A. et al. 2003. Evaluation of ecosystem dynamics, plant geography and terrestrial carbon cycling in the LPJ dynamic global vegetation model. Global Change Biology, 9: 161—185

6. SIMULATION RESULTS FROM CLIMATE-FVS

Giorgio Vacchiano

Università degli Studi di Milano, DISAA, 20123 Milano, Italy

1. METHODS

1.1 FOREST MODEL

The Forest Vegetation Simulator (FVS) is an individual tree, distance-independent growth and yield model extensively used in the United States to predict forest stand dynamics. Spatial scales range from a single stand to thousands of stands. The temporal scale has traditionally been about 100 years, with simulation cycles of five to ten years. FVS operates at the individual tree level, simulating growth, mortality, and regeneration based on empirical relationships calibrated for 20 geographic areas ("variants") of the US (Dixon 2015). The original Prognosis model (Stage 1973) was developed for northern Idaho and western Montana, and modified as data from other geographic areas was analyzed to develop new model variants. A climate-sensitive model extension adjusts model predictions to take into account the effects of climate change on growth, mortality, carrying capacity, and regeneration (Crookston et al. 2010). Climate and species viability information are required as additional input to the model, and can be requested for the western US from an open-access US Forest Service website.

1.2 MORTALITY FORMULATIONS

Three types of mortality are estimated in FVS: (1) background mortality, (2) density related mortality and (3) breakup of overmature stands. Density related mortality accounts for mortality in stands that are dense enough for competition to be the causal agent (Dixon 1986). Mortality associated with the breakup of over mature stands is mainly age-related. All other mortality is attributable to background mortality (Dixon 2015). Mortality caused by insects, pathogens, fires, or other disturbance agents is handled by using supplementary FVS extensions or user-supplied keyword record files.

Regardless of its cause, the simulated amount of stand mortality (percent trees per hectare per simulation cycle) is dispersed to individual tree records (MR) in relation to a tree's percentile rank in the basal area distribution (PCT), and adjusted by a species-specific tolerance modifier (MWT). MR is bound between 0.01 and 1 (Dixon 2015).

[1] $MR = [0.84525 - (0.01074\ PCT) + (0.0000002\ PCT^3)] \cdot MWT \cdot 0.1$

Background mortality

Background mortality rate for each tree (RI) is computed when stand density is below the minimum level for density dependent mortality.

[2] $RI = [(1 + e^{p0 + p1 * DBH})^{-1}] \cdot 0.5$

where DBH is tree diameter at breast height, and p_0-p_1 are species-specific coefficients. The estimate is then adjusted to the length of the simulation cycle (Y) by a compound interest formula (Hamilton 1986):

[3] $RT = 1 - (1 - RI)^Y$

The overall amount of mortality calculated for the stand is the summation of the adjusted mortality rate (RT) across all live tree records.

Density-related mortality

Density related mortality is based on the relationship between current and maximum Stand Density Index (SDI) (Reineke 1933). Current stand SDI is calculated on a tree-by-tree basis (Stage 1968) using

[4] $SDI = \Sigma\ [a + b\ (DBH_i)^2]$

[5] $a = 10^{-1.605} \cdot [1-(1.605/2)\ ] \cdot [\Sigma\ (DBH_i)^2/N]^{1.605/2}$

[6] $b = 10^{-1.605} \cdot (1.605/2) \cdot [\Sigma\ (DBH_i)^2/N]^{(1.605/2)-1}$

where N is total trees per acre in the stand, and Σ is summation over all trees in the stand.

Maximum SDI is a species-specific value hard-coded in the model (but editable by the user). By default, density related mortality begins when the stand SDI is above 55% of stand SDI maximum, and peaks at 85% of stand SDI maximum. The stand SDI maximum is a weighted average of the individual species-specific SDI maxima. The weights are based on the basal area each individual species represents in the stand. The weighted maximum SDI is calculated each growth cycle. If the stand SDI at the time of inventory is > 90% of maximum stand SDI, then the maximum SDI is automatically reset, so the stand SDI at the time of inventory corresponds to the upper limit of density related mortality.

When stand density-related mortality is in effect, background mortality is turned off, and the total amount of stand mortality is determined based on position of the stand on its self-thinning trajectory relative to its maximum SDI. This trajectory can be thought of as a relationship between trees per hectare (TPHA) on the y-axis and quadratic mean diameter (QMD) on the x-axis, and determines how many trees will die in the projection cycle (Dixon 2015).

Climate-FVS mortality

The Climate extension instructs FVS to modify mortality predictions based on a species-specific viability score, e.g., an estimate of climate suitability from 0 (unsuitable) to 1 (suitable). The estimate is derived from bioclimatic models (Rehfeldt et al. 2009) fitted by a stepwise random forests algorithm for 74 tree species of the western United States on presence/absence from FIA permanent sample plots (Bechtold and Patterson 2005) supplemented with research plot data to provide about 117,000 observations (Crookston et al. 2010).

Data-free thresholds are defined for mortality modifications: at scores <0.2, 10-year survival probability is zero, then it scales linearly up to 1 at viability score = 0.5. Full survival is predicted for the viability score computed using contemporary climate. The logic is that if viability scores drop below those at which the species occurs currently, mortality rates increase, eventually resulting in extirpation. The overall mortality rate assumed by the model is the higher between competition- and climate-induced mortality.

An additional rule is that, if climate changes at a location more in magnitude than is equivalent to changing elevation 300 m, then trees start to die, even if their viability score stays high. The choice of 300 m was made to mimic the average elevation range of a seed zone (Rehfeldt 1994). The rule ramps up the 10-yr mortality rate to a maximum of 90%; trees growing beyond their adaptive range experience higher mortality rates.

The magnitude of climate change is computed by comparing the value of six climate metrics (mean temperature of the warmest and coldest months, degree days above 5°C, degree days below 0°C, mean annual precipitation times, and summer dryness index) for the year a given tree was born to those that correspond to a specific year in a simulation. This logic implies that trees that have established are adapted to the climate of the stand at the time of establishment.

Climate–FVS also modifies the maximum carrying capacity (SDI maximum) computed by FVS by the ratio of two weighted average maximum densities. For the first one, D1 (bound to >0), the weights equal the species viability scores at the beginning of the simulation. For the second, D2, the weights are the viability scores computed in simulated time. The proportional change in carrying capacity is r = D2/D1 (bound to <1.5). According to this logic, maximum stand density will increase when the site becomes more suitable for species that carry high densities, but will decrease when the climate favors species that carry lesser density (Crookston et al. 2010). The modified carrying capacity is then used in FVS mortality calculations.

Finally, Climate-FVS can optionally affect growth and regeneration submodels (details in Crookston et al., 2010).

Growth-based mortality

The Event Monitor extension (Crookston 2002) allows FVS to run specific instructions subject to conditional IF… THEN… ENDIF expressions in the model initialization file. A possible use of the Event Monitor is to execute instructions conditional on the level of any state variable at any time step within the simulation.

Extraction of individual tree parameters (e.g., to compute growth efficiency or maximum growth) is not possible in the Event Monitor. However, stand-scale state variables can be retrieved by using the COMPUTE SPMCDBH command in the FVS initialization file, to return the number of trees, basal area, total volume, merchantable volume, quadratic mean diameter, average height, percent cover, SDI, or 10-year diameter growth for a given species or species group and range of diameter and height. These metrics can be obtained for live trees, cut trees, dead trees, live trees after thinning, or mistletoe infected trees (Dixon 2015).

By using a combination of COMPUTE statements to generate 10-year radial increments (RW_i) for each species and dbh class, conditional statements, and FIXMORT statements in the initialization file to impose the desired mortality rates, we replaced competition-related mortality by an alternative mortality rate (RT_i) based on absolute growth increment thresholds, after the Jabowa (Botkin et al. 1972) logic:

[7] $RT_{10} = 0.01$ if $RW_{10} > 1.6$ mm

[8] $RT_{10} = 0.99$ if $RW_{10} < 1.6$ mm

2. SIMULATION SETTINGS

2.1 SITE DATA

We used three stands provided by the US Forest Management Service Center as test cases for FVS. The stands are located in Modoc National Forest, Big Valley Ranger District in Northeastern California (41.13° – 41.20° N; 120.78° – 120.86° W) on former lava flows at elevations of 1200, 1350, and 1500 m a.s.l. Forest types are California Mixed Conifer (2 stands, 25 plots) and Ponderosa pine (1 stand, 31 plots). The main species are Ponderosa pine (*Pinus ponderosa*) and incense-cedar (*Libocedrus decurrens*) in the upper canopy, and white fir (*Abies concolor*) in the understory. Stands are uneven-

aged with tree of up to 117 cm DBH and 38 m height. The location of the stands required using the South-Central Oregon and Northeast California variant of FVS (SO-FVS) (Keyser 2015). Data were collected during the 2007 FIA survey; FIA mapped (annual) design plots have four 0.016-ha subplots for a total surface area of 0.064 ha, resulting in an expansion factor of 15 trees per hectare for tally trees > 12.5 cm in diameter. Seedlings and saplings are measured on 13.3 m^2 microplots that are nested within each subplot, resulting in a per-hectare expansion factor of 187.425 (Bechtold and Patterson 2005).

Soil, climate and site properties are coded in FVS by Site Index and by model coefficients specific to the geographical variant and Location (e.g., National Forest), Habitat Type or Plant Association Code. In FVS-SO, Location is the only site-sensitive parameter, and it was held constant for all simulated stands in this exercise (Modoc National Forest = Location Code 509). Site index (base age: 50 years for white fir, 100 years for ponderosa pine) was entered using values provided by FIA. Species-specific SDI maxima and submodel coefficients are described by the SO-FVS variant manual (Keyser 2015).

Current climate simulations were run by turning off the Climate-FVS extension. Future climate scenarios were initialized via a Climate-FVS input file obtained by querying the website http://forest.moscowfsl.wsu.edu/climate/customData/fvs_data.php. Estimates are provided for each stand using spline climate surfaces (Hutchinson 1991) of monthly averages of mean, maximum, and minimum temperature and precipitation for stand coordinates. From monthly data, the spline algorithm generates 35 variables with direct relevance for plant ecophysiology, such as mean annual temperature and precipitation, degree days above 5 °C, degree days below 0 °C, the length of the frost period, and annual dryness index. Future climate scenarios (Table 1) are based on a downscaling of GCMs computed using a delta method (Daniels et al. 2012) relative to the observed climate in 1961-1990. An ensemble simulation was run using three specific GCMs (Geophysical Fluid Dynamics Laboratory, Hadley Center/Met Office UK, The Community Earth System Model) under IPCC Fifth Assessment Report's RCP 4.5 and RCP 8.5 scenarios (Intergovernmental Panel on Climate Change 2014).

Table 1 – Change in climate variables for year 2090 relative to 1960-1990

	Temperature difference				Precipitation ratio			
**scenario**	DJF	MAM	JJA	SON	DJF	MAM	JJA	SON
**RCP 4.5**	2.8	3.13	3.87	3.1	1.09	1.13	0.89	1.04
**RCP 8.5**	4.9	5.07	7.37	5.4	1.11	1.21	0.78	1.13

2.2 *Test data*

No long-term measurements were available for the simulated stands. Therefore, we carried out data-free verification of model behavior (Cawrse et al. 2010) by scrutinizing the self-thinning trajectory of each modeled stand, i.e., the increase in mean tree size with progressive density reduction due to competition-induced mortality. When plotted on log (QMD) – log (TPHA) axes, each stand should asymptotically approach the maximum self-thinning line as it develops through time. For this experiment, maximum SDI was computed at each cycle as the weighted average, by basal area proportion, of the individual species SDI maximums (ponderosa pine: 1427; white fir: 1897; incense-cedar: 1765) (Keyser 2015). Only current climate scenarios were used for model verification.

2.3 *Simulation experiments*

FVS runs were initialized with measured plot and tree data (no spin-up required). Climate-FVS runs were initialized with US Forest Service climate scenarios for the simulated stands from 2007 (inven-

tory year) to 2207. Three future time points are addressed in climate scenario input files: the 10 years surrounding 2030, 10 years surrounding 2060, and 10 years surrounding 2090. Points in time between those periods are linearly interpolated by Climate-FVS routines; values associated with the latest year are used when simulated time exceeds the latest year in the file – i.e., constant climate after 2090 (Crookston 2015).

Each climate scenario (current + 2 climate change) was run using the following alternate mortality formulations

1) a base FVS run with SDI-based and climate-induced mortality. Preliminary runs showed that age-related mortality did not produce significantly different outputs, therefore it was not simulated.
2) a growth-dependent mortality (JABOWA absolute thresholds)

All runs were operated on 10-years simulation cycles. To avoid local extinction due to climate-induced mortality, natural regeneration was added by using the NATURAL keyword (Ferguson and Crookston 1991) with a 250, 125, and 75 seedlings per hectare planted at every cycle (i.e., ten years) for white fir, ponderosa pine, and incense-cedar respectively. White fir seedlings were set to occur more frequently on plots with less overstorey basal area (shade code =2), while ponderosa pine and incense cedar seedlings were set to occur more frequently on plots with more overstorey basal area (shade code =1); seedling age and survival at each cycle were set at 2 years and 10%, respectively.

Model output was reported as time series of basal area (BA), live trees per hectare and mortality trees per hectare per cycle for the three stands, two mortality models, and three climate scenarios, for a total of 18 simulation runs.

3. SIMULATION RESULTS

The self-thinning trajectory of the three stands simulated by default FVS-SO (Figure 1, left) shows the effect of both regeneration, i.e., increasing trees per hectare in the earlier cycles, and of density-dependent mortality, i.e., stands tracing the maximum self-thinning line (with weighted SDImax higher for the stand dominated by white fir and lower for ponderosa pine cover types). The alternative growth-dependent mortality (Figure 1, right) does not seem to produce the correct emergent self-thinning trend, with the low- and mid- elevation stand growing past their theoretical carrying capacity, and the high elevation one collapsing after about one hundred years.

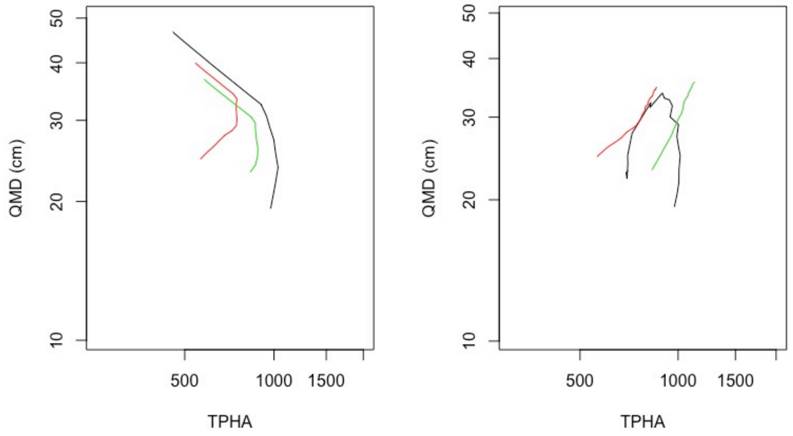

Figure 1 – QMD-tree density trajectories of the simulated mixed conifer stands (red: 1200 m, green: 1350 m, black: 1500 m a.s.l.) under current climate using two mortality algorithms (left: Climate-FVS, right: growth-dependent). Simulation time proceeds from bottom-right to top-left.

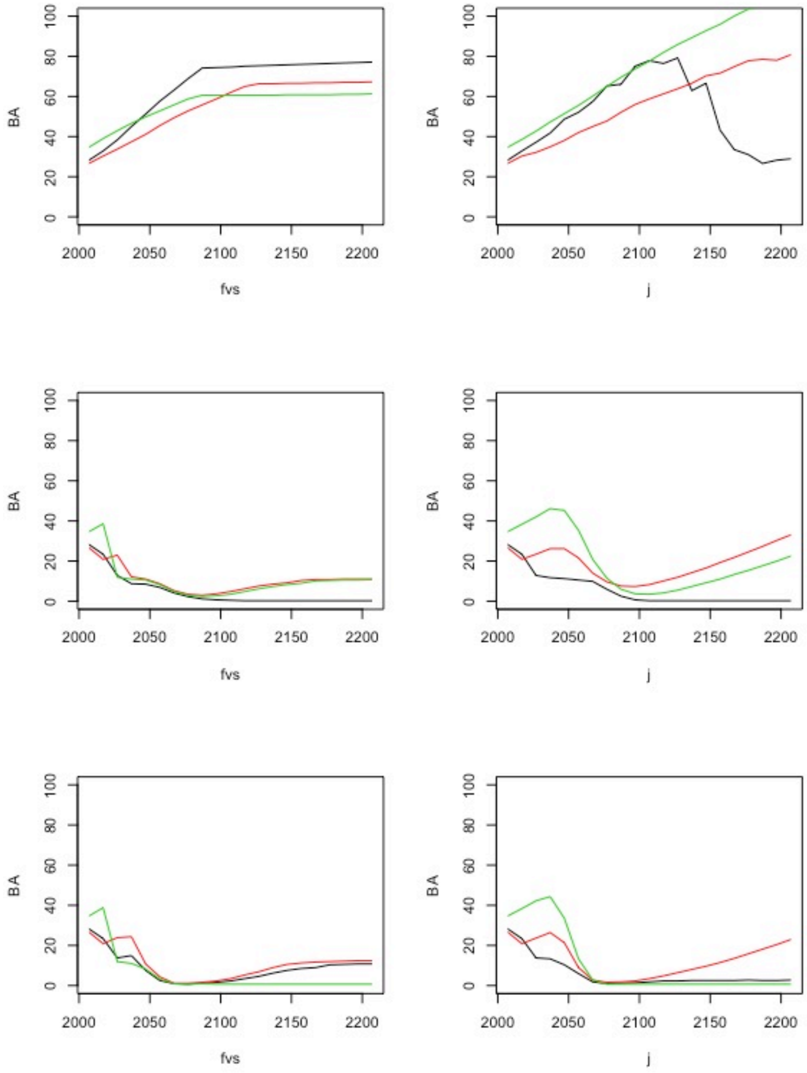

Figure 2 – Basal area [m^2 ha^{-1}] for the simulated mixed conifer stands (red: 1200 m, green: 1350 m, black: 1500 m a.s.l.) under current climate (top), RCP 4.5 (middle), and RCP 8.5 (bottom) and two mortality algorithms (left: Climate-FVS, right: growth-dependent).

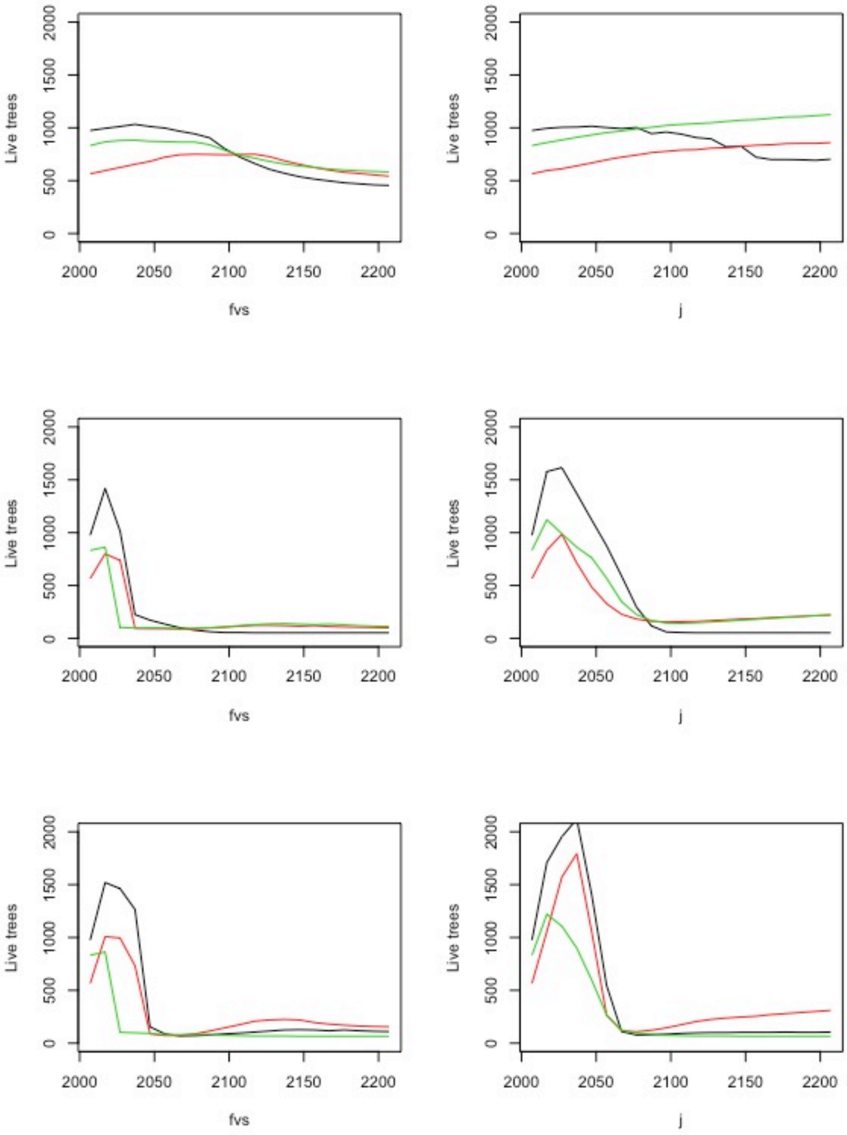

Figure 3 – Live tree density ha^{-1} for the simulated mixed conifer stands (red: 1200 m, green: 1350 m, black: 1500 m a.s.l.) under current climate (top), RCP 4.5 (middle), and RCP 8.5 (bottom) and two mortality algorithms (left: Climate-FVS, right: "j" growth-dependent).

Standard FVS-SO simulations produced a continuous increase of stand basal area, with similar trends in all three stands and up to a maximum of 60-80 m^2 ha^{-1} (Figure 2, upper left), i.e., close to the maximum basal area attainable by mixed stands of the three species. Mortality increases starting from year 70 of the simulation, after which stand density is reduced according to a self-thinning logic down to 500-600 trees per hectare (Figure 3, upper left), and is highest in the high elevation stand, probably due to worse growing conditions and more intense competition in the understory layer of white fir (Figure 4, upper left). Under growth-dependent mortality, stands grow more biomass than under SDI mortality, with a higher share of small diameters, except in the high elevation stand, where mortality steadily increases in the second hundred years of the simulation. This is probably due to the fact that in FVS-SO tree growth depends not only on competition but also on site parameters such as elevation and site index. Therefore, it is more likely that, with increasing stand basal area, trees exhibit slow growth and fall below the mortality threshold.

The effect of climate change on predicted basal area and stand density was higher than that of changes in the mortality algorithm. Final basal area was < 25 m^2ha^{-1} in all stands, likely due to increased drought stress. RCP 4.5 induced stand collapse in the high-elevation stand, while RCP 8.5 induced collapse in the mid-elevation one, likely due to the different response of the most common species in the stand to increased drought (RCP 4.5) or increased warming with unchanged precipitation (RCP 8.5, Table 1). Regeneration of the three main species showed some adaptation potential and contributed to maintain what little forest cover remained along the simulation (e.g., incense-cedar in the low-elevation stand, Figure 2 bottom). However, the way Climate-FVS computes mortality produced an unrealistic peak of mortality starting from the third simulation cycle, that affected all subsequent data (Figure 3). Even if trees are added by regeneration, in the following timesteps stands cannot recover from such severe climate-induced mortality, therefore the final tree densities are quite similar to each other regardless of the mortality algorithm used (i.e., low sensitivity to mortality algorithm under climate change).

These results suggest that the addition of climate impacts on this fundamentally empirical model is still far from efficient, and that validation against existing data is extremely important to fine-tune the thresholds and multipliers that are applied to the mortality algorithms.

4. References

Bechtold, W.A., & Patterson, P.L., eds. (2005). The enhanced Forest Inventory and Analysis program – national sampling design and estimation procedure. Gen. Tech. Rep. SRS-80. Asheville, NC: US Department of Agriculture, Forest Service, Southern Research Station.

Botkin, D. B., Janak, J.F., Wallis, J.R. (1972) Some ecological consequences of a computer model of forest growth. J Ecol 60:849-872.

Cawrse, D., Keyser. C., Keyser, T., Sanchez Meador. A., Smith-Mateja, E., Van Dyck, M. (2010) Forest Vegetation Simulator Model Validation Protocols. Fort Collins, CO: US Department of Agriculture, Forest Service, Forest Management Service Center.

Crookston, N. L. (2002). User's guide to the Event Monitor: part of Prognosis Model, version 6. Gen. Tech. Rep. INT-275. Ogden, UT:US Department of Agriculture, Forest Service, Intermountain Research Station.

Crookston, N. L. (2015). Climate-FVS Version 2: Content, users guide, applications, and behavior. Gen. Tech. Rep. RMRS-GTR-319. Fort Collins, CO: US Department of Agriculture, Forest Service, Rocky Mountain Research Station.

Crookston, N.L.; Rehfeldt, G.E.; Dixon, G.E.; Weiskittel, A.R. (2010). Addressing climate change in the forest vegetation simulator to assess impacts on landscape forest dynamics. Forest Ecology and Management. 260:1198-1211.

Daniels, A.E.; Morrison, J.F.; Joyce, L.A.; Crookston, N.L.; Chen, S.C.; McNulty, S.G. (2012). Climate projections FAQ. Gen. Tech. Rep. RMRS-GTR-277WWW. Fort Collins, CO: US Department of Agriculture, Forest Service, Rocky Mountain Research Station.

Dixon, G.E. (1986). Prognosis mortality modeling. Internal Rep. Fort Collins, CO: US Department of Agriculture, Forest Service, Forest Management Service Center.

Dixon, G.E., comp. (2015). Essential FVS: A user's guide to the Forest Vegetation Simulator. Internal Rep. Fort Collins, CO: US Department of Agriculture, Forest Service, Forest Management Service Center.

Ferguson, D.E., & Crookston, N.L. (1991). User's guide to version 2 of the regeneration establishment model: part of the prognosis model. Gen. Tech. Rep. INT-279. Ogden, UT: US Department of Agriculture, Forest Service, Intermountain Research Station.

Hamilton, D.A. (1986). A logistic model of mortality in thinned and unthinned mixed conifer stands of northern Idaho. Forest Science, 32(4), 989-1000.

Hutchinson, M.F. (1991). Continent wide data assimilation using thin plate smoothing splines. Pages 104-113, In: J.D. Jasper, ed. Data assimilation systems. BMRC Research Report 27, Bureau of Meteorology, Melbourne, Australia.

Intergovernmental Panel on Climate Change. (2014). Climate Change 2013: The physical science basis: Working group I contribution to the fifth assessment report of the Intergovernmental Panel on Climate Change. Cambridge University Press.

Keyser, C.E. (2015). South Central Oregon and Northeast California (SO) variant overview—Forest Vegetation Simulator. Intern. Rep. Fort Collins, Co. US Department of Agriculture, Forest Service, Forest Management Service Center.

Rehfeldt, G.E. (1994). Genetic structure of western red cedar populations in the Interior West. Canadian Journal of Forest Research, 24(4), 670-680.

Rehfeldt, G.E., Ferguson, D. E., & Crookston, N. L. (2009). Aspen, climate, and sudden decline in western USA. Forest Ecology and Management, 258(11), 2353-2364.

Reineke, L. (1933). Perfecting a stand-density index for even-aged forests. Journal of Agricultural Research, 46(7): 627-638.

Stage, A.R. (1968). A tree-by-tree measure of site utilization for grand fir related to stand density index. Research Note INT-77. Ogden, UT: US Department of Agriculture, Forest Service, Intermountain Research Station.

Stage, A.R. (1973). Prognosis model for stand development. Res. Pap. INT-RP-137. Ogden, Utah: Fort Collins, CO: US Department of Agriculture, Forest Service, Intermountain Forest and Range Experiment Station.

Van Dyck, M.G., & Smith-Mateja, E.E. comps. 2015 (revised frequently). Keyword reference guide for the Forest Vegetation Simulator. Internal Rep. Fort Collins, CO: US Department of Agriculture, Forest Service, Forest Management Service Center.

7. Simulation Results from GOTILWA+

Daniel Nadal-Sala[1], Santiago Sabaté[1,2], Carlos Gracia[1,2]

[1] Department de Biologia Evolutiva, Ecologia i Ciències Ambientals, Universitat de Barcelona. Av. Diagonal 643, 08028, Barcelona, Spain
[2]CREAF (Centre de RecercaEcològicaiAplicacionsForestals), CerdanyoladelVallès 08193, Spain

1. Methods

GOTILWA+ (Growth Of Trees Is Limited by WAter, http://www.creaf.uab.cat/gotilwa/) is a process-based (Keenan *el al.*, 2009b,c; Fontes*et al.*, 2010; Nadal-Sala *et al.*, 2014) forest growthsimulation model. GOTILWA+ model has been tested using data from Forest Inventories (e.g. National Forest Inventories), Eddy Flux towers outputs, as well as compared to other process based models (see Kramer *et al.*, 2002, Morales *et al.*, 2005, Keenan *et al.*, 2009a). GOTILWA+ has been successfully applied Europe-wide (see Schröter*et al.*, 2005; Keenan *et al.*, 2009b,c; Keenan *et al.*, 2010). GOTILWA+ performs forest growth under different climate, stand structure, management options and soil traits. GOTILWA+ describes carbon and water fluxes through forests and has been applied on a wide range of environmental conditions - from boreal northern Europe to Mediterranean basin, and also in the Ecuadorian Andes in *Polylepis reticulat*a tree species-. Its programme code is built using Microsoft Visual Basic (6.0) platform.

GOTILWA+ time step resolution is hourly based, and calculations are integrated into daily, monthly and yearly values. Leaf area vertical distribution distinguishes two canopy layers (under sunny and shaded conditions) but there is no explicit description of the leaf area horizontal distribution. Trees are grouped by DBH size classes, where individuals are treated mostly as identical. Light extinction coefficient is estimated using Campbell's equation (Campbell, 1986). Photosynthesis is calculatedusingFarquhar's equations (Farquhar and Von Caemmerer, 1982). Stomatal conductance calculation uses Leuning, Ball and Berry approach (Leuning, 1995). Leaf temperature is determined by the leaf energy balance equation described by Gates(1962; 1980).Potential evapotranspiration is estimated by Penman–Monteith equation (Monteith, 1965; Jarvis and McNaughton, 1986) following the hourly calculation procedure described in Allen *et al.* (1998). Specific tree species parameters related to photosynthetic capacity, leaf morphology and leaf hydraulic conductivity are used (taken from measurements or literature) and environmental input variables are incident radiation, wind speed, atmospheric water vapour pressure, temperature max and min, precipitation and atmospheric CO_2 concentration.

Carbon balance and tree decline by Carbon Starvation

Net Primary Production (NPP) is obtained from Gross Primary Production (GPP)minus maintenance respiration (M_R) following equation 1:

$$\mathrm{NPP} = \mathrm{GPP} - \left(\frac{\mathrm{M_R}}{\mathrm{E_E}}\right) \qquad \text{Equation 1]}$$

Where NPP andGPP are expressed in kg·hour^{-1}·ha^{-1}, M_R is maintenance respiration, expressed in kcal·hour^{-1}·ha^{-1} and E_E is the energetic equivalence of organic matter, assumed as a constant value of 9.4·10^3 kcal·kg^{-1}.

M_R is determined by the sum of the respiration of leaf biomass, fine root biomass and living wood biomass. Living wood biomass is a species-specific percentage of wood biomass. M_R rates depend on temperature according to a Q10 approach following equation 2.

$$Q10_t = Q10_{25}^{\frac{(T_t - 25)}{10}} \qquad \text{Equation 2]}$$

Where $Q10_t$ is the Q10 value at a given t, $Q10_{25}$ is the standardized value of Q10 at 25 ºC and T_t is the tissue temperature in ºC. GOTILWA+ differentiates between respiration rates of structural and mobile carbohydrates. Respiration rate is 33.3 kcal·kg^{-1}·day^{-1} at 25ºC for structural carbohydrates and 55.5 kcal·kg^{-1}·day^{-1} at 25ºC for mobile carbohydrates, following Ovington (1961). Thus, M_R for a given tissue follows the equation 3:

$$M_R = \mathrm{B} \cdot Q10_t \cdot RR_c \qquad \text{Equation 3]}$$

M_R is the tissue maintenance respiration for a given tissue, in kg·ha^{-1}·hour^{-1}, B is the respiring biomass of a given tissue, in kg·ha^{-1}, $Q10_t$ is the value of Q10 at a given tissue temperature, RR_C is the respiration rate for a given carbon fraction – i.e. structural or mobile carbon fraction – in kcal·kg^{-1}·hour^{-1}.

NPP is then allocated through the tree compartments following a set of hierarchical decision criteria. First NPP refills tree mobile carbohydrate reserves up to the maximum replenishment values. Then NPP is used to equilibrate according to the pipe model leaf area, fine root biomass and sapwood area (Shinozaki *et al.*, 1964). When new tissues are produced, carbohydrates are also spent on growth respiration (G_R). G_R is 32% of the invested carbohydrates for growth - i.e. a constant efficiency of 0.68 g of new tissue per g of carbohydrate (Ovington, 1961)-. Finally, if there is still NPP available, trees generate new sapwood area, new leave area and new fine root biomass according to the pipe model and accounting for G_R costs as above.

When there is no photosynthetic activity or this is not sufficient to compensate respiration rates, NPP values turn to negative. If so, the lack of photosynthesis to maintain respiring tissues is offset by the mobile carbon reserves. While mobile carbon pool is fully available, it can be depleted without consequences for tree population. When mobile carbohydrates reserves falls close to mortality threshold, trees lose respiring tissues as leaf and fine roots biomass. If carbon starvation continues and mobile carbon pool falls below a certain species-specific threshold, tree mortality event occurs. Mortality also occurs if the diametric class is completely defoliated, and mobile carbon is not available anymore, during the vegetative period in the case of deciduous tree species or at each moment of the year for the evergreen ones.

GOTILWA+ does not consider homogeneous distribution of mobile carbon reserves within a DBH class. Concerning mortality, GOTILWA+, instead of working with the DBH class average tree, it assumes differences within the class. The pool of MCH is not homogeneous distributed, thus there are trees in better condition than others. The number of dead trees is established as follows:

The number of trees that can be sustained by the current MCH values is calculated. Then, the difference between the current number of trees and the number of trees that can be sustained is the mortality within the DHB class. In addition to the MCH, the rest of the tree structure compartments are restructured accordingly.

Loss of functional sapwood area

GOTILWA+ establishes a threshold of yearly sap flow - in $l\cdot cm^{-2}_{sapwood}\cdot year^{-1}$ - to maintain sapwood functionality. If there is some stress in tree transpiration, - i.e. extremely dry year, insufficient evaporative demand - that produces a lesser sap flow rate than threshold, then the diametric class reduces sapwood area functionality following the equation:

$$As_{remaining} = \frac{\mathrm{Js}}{\mathrm{Js_{min}}} * \mathrm{As_{current}} \qquad \text{Equation 4]}$$

Where $As_{remaining}$ is the remaining functional sapwood area in $cm^2\cdot tree^{-1}$, Js is the simulated sap flow density in $l\cdot cm^{-2}_{sapwood}\cdot year^{-1}$ and Js_{min} is the threshold for sapwood functionality loss, also in $l\cdot cm^{-2}_{sapwood}\cdot year^{-1}$. $As_{current}$is is the current sapwood area in $cm^2\cdot tree^{-1}$ at the end of the year for a given DBH class.

Losing sapwood functionality does not imply tree mortality, but it produces a misbalance in the *pipe model*, which has to be compensated by leaf shedding and fine root biomass mortality. This affects both the tree available mobile carbon reserves and its photosynthetic capacity. It, then, may evolve into short-term carbon starvation and tree mortality.

Alternative mortality formulation: the self-thinning rule

It has been established tree mortality following the principles of self-thinning rule described by Yoda et al (1963). The formulation for the self-thinning rule has been implemented as described in equation 5:

$$\ln(AB_{tree}) = K - \ln(N) \qquad \text{Equation 5]}$$

Where $\ln(AB_{tree})$ is the logarithm of the mean tree basal area for a given stand, ln(N) is the logarithm of the number of trees for a given plot and K is a site-specific constant. A higher K value corresponds to higher maximum total basal area supported by the stand.

This optional model has been implemented in GOTILWA+ model to compare GOTILWA+ carbon starvation mortality with mortality driven by Self-thinning rule. In the last case, GOTILWA+ calculates annually if the number of individuals of the forest stand is higher than the maximum number possible for a given K and the current AB_{tree}. If so, random trees proportionally distributed across all DBH classes die until the current N matches the predicted N by Self-thinning rule.

2. SIMULATION SETTINGS

GOTILWA+ was run in Peitz (Brandemburg, Germany, 51.98º N, 14.13º E), in an even-aged *Pinus sylvestris* forest. Inventory data from this forest is available since 1952. Forest data during 1952-2010 period includes forest management. First, a model validation was performed using this period and applying the same management as described in the forest tables from the site. Then GOTILWA+ was run over 200 years, from 1971 to 2170, but not including forest management and with an initial tree population corresponding to the same present in 1971.

Measured daily climate data values covers from 1900 to 2010. After 2010, 160 years of baseline daily climate data were generated using GOTILWA+ weather generator. This baseline data keeps the same statistical characteristics and temporal autocorrelation proprieties than measured Peitz climate data of the period 1971-2010. Then, two climate change scenarios based on IPCC (2013), plus one baseline climate scenario called RCP0.0 were applied upon this baseline scenario. Those climate change scenarios modify the 2010-2100 period climate data according to the table 1. After the year 2100, no further climate change forcing was added, modifying the climate time-series according to the 2100

anomalies and keeping the CO_2 concentration constant at the 2100 values for all climate scenarios (Fig. 1).

Table 1: Climate change scenarios applied during the 2010-2100 period to the reference time-series following IPCC (2013) plus the Baseline zero emissions climate

Climate change scenario	CO_2 concentration (Δppm·year^{-1})	Temperature anomaly (ΔºCYear^{-1})	Precipitation anomaly (Δ%Year^{-1})
**RCP 0.0 (Baseline)**	0	0	0
**RCP 2.6**	0.21	0.018	-0.21
**RCP 8.5**	5.15	0.056	-0.35

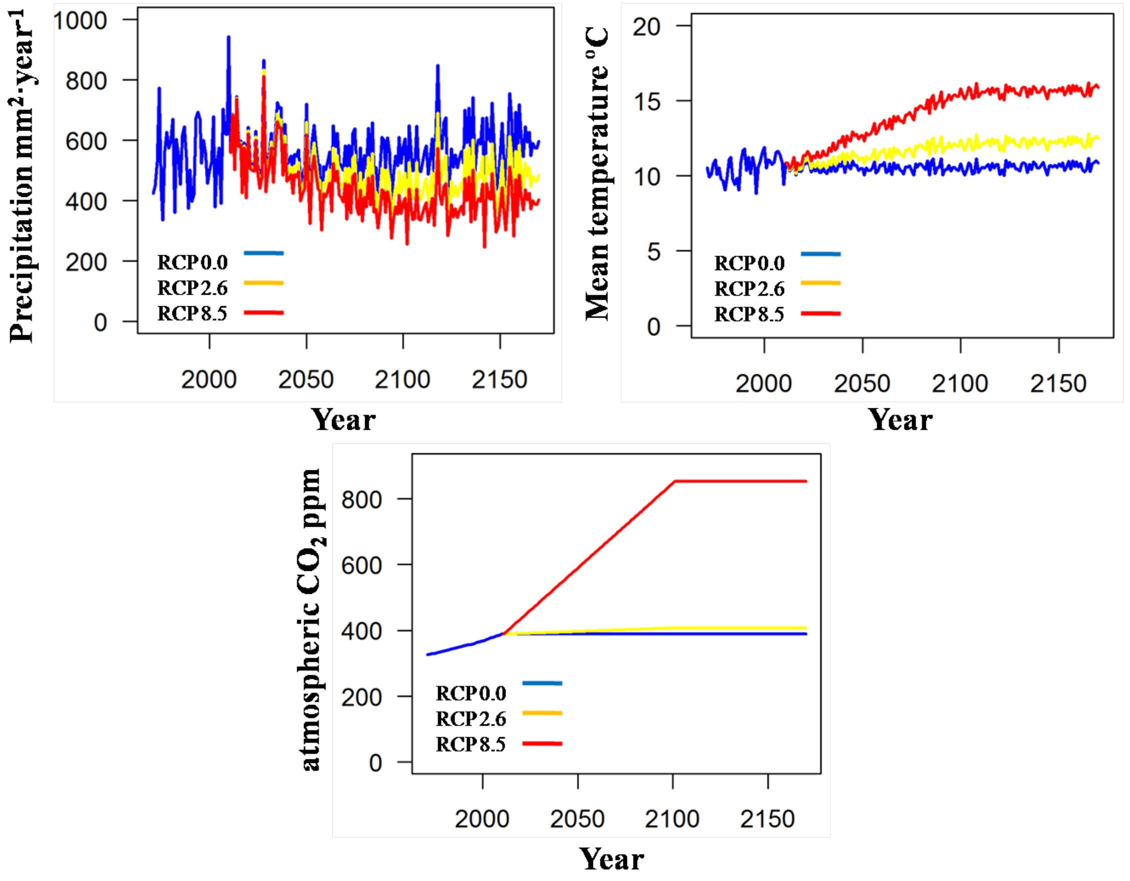

Figure 1: Meteorological inputs throughout the simulations for all three climate change scenarios. Figure shows annual precipitation in mm·year^{-1}, annual mean temperature in ºC and annual CO_2 concentration in ppm

Two datasets of forest structure were generated according to the forest inventories, one from the original 1952-year stand, and the other from the 1971 stand. The first one was used for model calibration and the second one for experimental runs. Stand tree density by DBH classes is summarized in table 2:

Table 2: Tree density in the Peitz plot at year 1952 and at year 1971

	Density (trees·ha-1)	
**DBH class (cm)**	Year 1952	Year 1971
**0-5**	260	0
**5-10**	2780	470
**10-15**	630	1280
**15-20**	0	400
**20-25**	0	0

An important input of GOTILWA+ is the soil characteristics, which is closely related to water availability. Following provided soil data, soil depth was set as 2 m, and soil carbon content was set as 1.75%. Field capacity was set as 26%. K value of equation 5 was 13.2. That was obtained from boundary analysis on basal area and tree density field measurements.

3. SIMULATION RESULTS

Model validation

A spin-off period of 19 years (1952-1970) has been applied to avoid model bias. During the 1971-2010 period, there is a significant (p<0,05, N = 8, R^2 = 0.501, figure 2) correlation between observed and modelled basal area (BA). Simulated basal area is on average 7.3% different than measured one. DBH also matches in both observed and modelled values. Observed and modelled DBH values differs 4.5% during the 1971-2010 period.

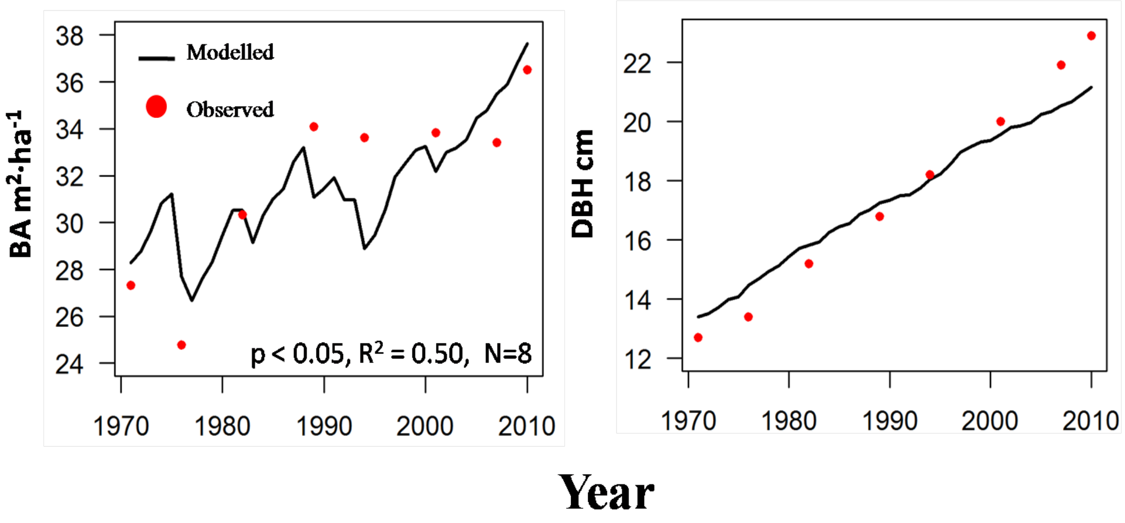

Figure 2: Comparison between the observed values of basal area (BA, left), and diameter at breast height (DBH, right), for the modelled (line) and the observed (dots) values during the 1971-2010 period

Simulation results for the future

In the Carbon Starvation algorithm results, tree mortality appears more intense during the first years of the simulation due to a higher competition between individuals (Fig. 3). We observed lower mortality rates in RCP8.5 climate scenario. This might be due to the fertilizing effect of the increase in atmospheric CO_2, which increases water use efficiency (Keenan *et al.*, 2013), and compensates the

worsening conditions of water availability and temperature. In RCP2.5, higher mortality rates than the baseline RCP0.0 climate scenario are shown throughout the simulation. In the self-thinning mortality algorithm, tree mortality starts at the period 45-55 of the simulation, far after mortality is observed in the carbon starvation algorithm. Abrupt mortality events occur in lower productive climate scenarios - i.e. RCP0.0 and RCP2.6-. Those abrupt mortality episodes are related to tree massive defoliation and complete mobile carbohydrates reserve depletion, which results in the death of all individuals within a DBH class.

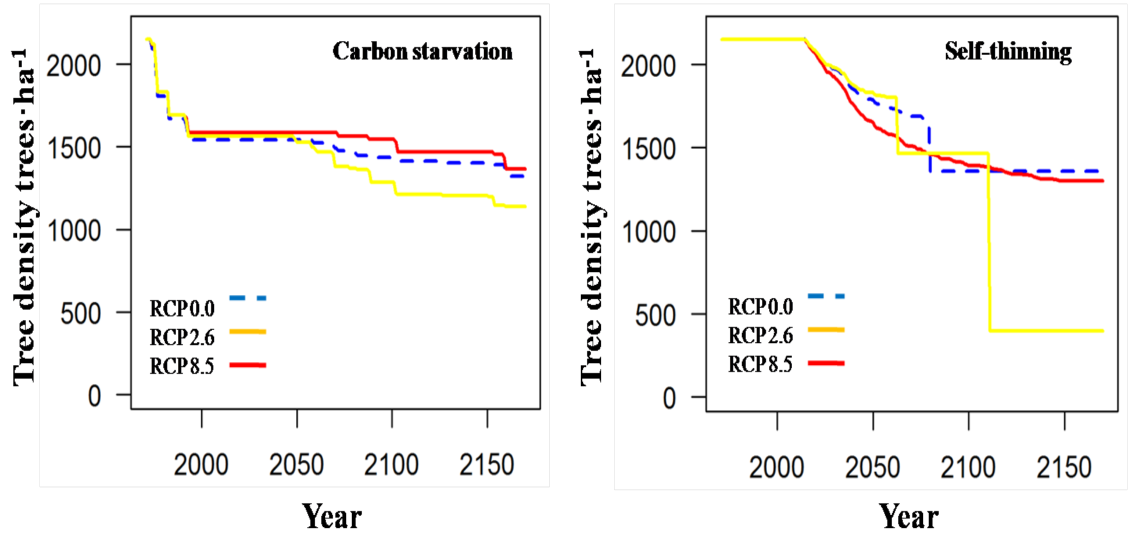

Figure 3: Tree density values across the simulation for the Carbon Starvation (left) and Self-thinning (right) mortality events scenarios. The three RCP scenarios are represented

On the other hand, basal area and aboveground biomass reaches higher values in the RCP8.5 scenario (Fig. 4), followed by the RCP0.0 and the RCP2.6 scenarios. Again, it is attributable to the fertilizing effect of rising atmospheric CO_2 concentration. However, the increase of atmospheric CO2 concentration wouldn't be enough in the RCP2.6 scenario to compensate the temperature increase as well as the reduction in precipitation amount.

Comparison of carbon starvation and self-thinning rule algorithms shows that they both converge in the final aboveground biomass for the RCP0.0 and RCP2.6 climate scenarios. Furthermore, differences between their values does not differ more than ±50 $Mg \cdot ha^{-1}$ during all simulation, and they almost overlap during all the period considered - ±1.7% difference across the simulation for the RCP0.0 and ±1.5% difference in the RCP8.5-.

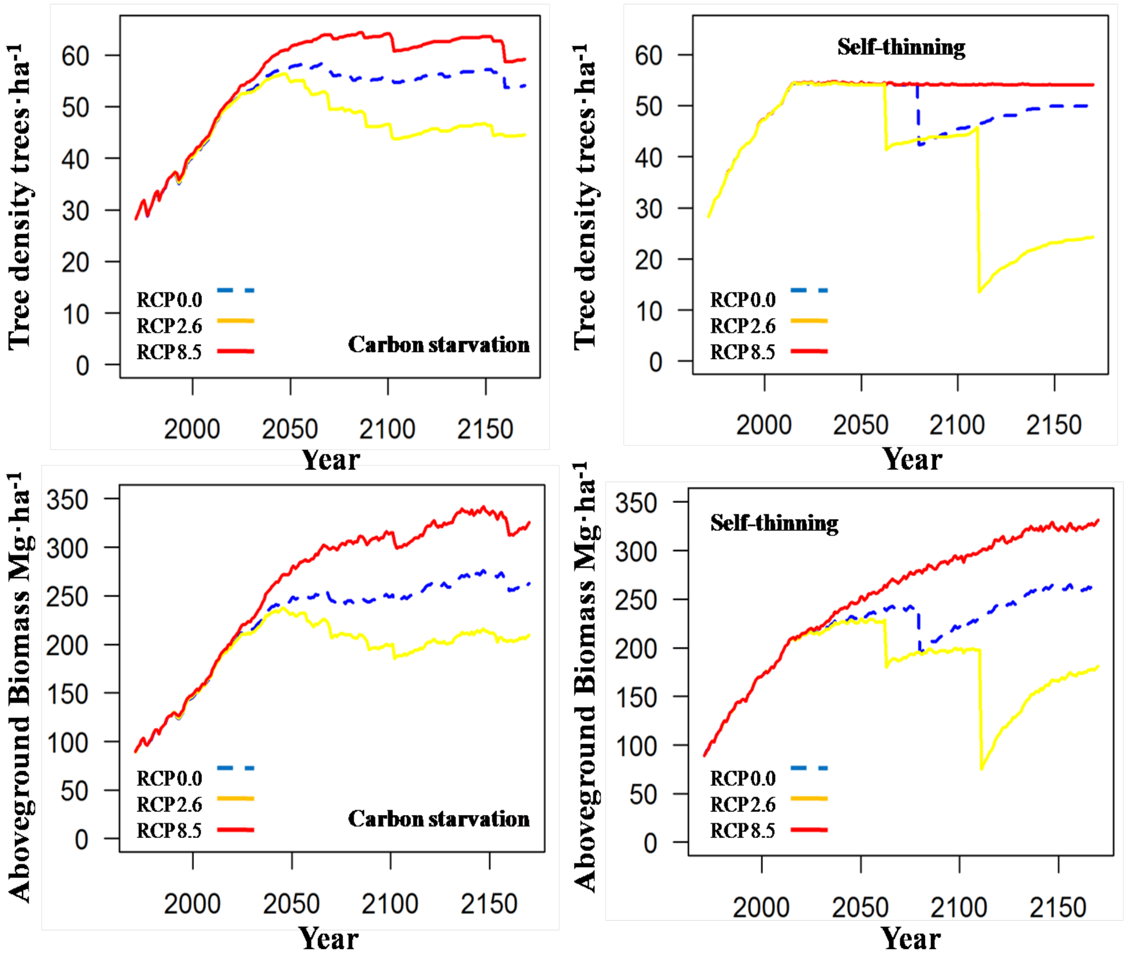

Figure 4: Basal area (up) and aboveground biomass (down) values across the simulation for the Carbon Starvation (left) and Self-thinning (right) mortality events scenarios. The three RCP scenarios are represented

Our results suggest that self-thinning rule by himself is not a good predictor of tree mortality in non-steady state conditions, because a given K value is assumed constant through the simulation. K value shall be modified across the time in a changing environment according to climate, which, in climate change scenarios, is not by definition in steady-state. Furthermore, we obtain the K value in an "*a posteriori*" analysis of the tree population, and, in changing environments, tree population changes its composition according to environmental conditions change. This fact is noted by the maximum basal area in the plot, which may increase above a threshold within the carbon starvation simulation and for the same climate scenario, but it is fixed at 54.8 $m^2 \cdot ha^{-1}$ in the self-thinning simulation across all climate change scenarios considered.

Forest transpiration is a key element in watershed-based water balances, particularly when precipitation is lower than potential evapotranspiration (Sabaté and Gracia, 2011). A crucial issue when anticipating the effect of climate change upon forest ecosystems is the amount of water returned to the atmosphere as transpiration. As indicatedin table 3, an increase of the green water fraction of precipitation is expected both by the RCP2.6 and RCP8.5 with respect to the baseline scenario. It is important to remark that an increase of forest fraction of evapotranspiration means a lesser amount of water availability for the other ecosystems downstream, included human society (Otero *et al.*, 2011). No differences in water use were observed between the carbon starvation and the self-thinning model.

Table 3: Fraction of precipitation transpired by forest canopy for all tree RCP climate scenarios and for three time slices representing the initial value, the mean simulation period and the last simulation period

Period (year)	1971-2010	2051-2090	2131-2170
**RCP 0.0**	0.67	0.72	0.7
**RCP 2.6**	0.67	0.78	0.82
**RCP 8.5**	0.67	0.82	0.88

4. References

Allen RG, Pereira LS, Raes D Smith M. 1998.Crop evapotranspiration. Guidelines for computing crop water requirements. FAO Irrigation and Drainage Paper No. 56, FAO, Rome, 290 pp.

Campbell GS. 1986. Extinction coefficients for radiation in plan canopies calculated using an ellipsoidal inclination angle distribution. Agric. For. Meteorol. 36, 317–321.

Farquhar GD,S Von Caemmerer. 1982. Modeling of photosynthetic response to environment. In: Lange, O.L., Nobel, P.S., Osmond, C.B., Ziegler, H. (Eds.), Encyclopedia of Plant Physiology: Physiological Plant Ecology II, Water Relations and Carbon Assimilation, Vol. 12B. Springer, Berlin, pp. 549– 587.

Fontes L, J-D Bontemps, H Bugmann, M Van Oijen, C. Gracia, K, Kramer et al. 2010. Models for supporting forest management in a changing environment. Forest Systems 19:8-29

Gates D.M. 1962. Leaf temperature and energy exchange. Theoretical and Applied Climatology 12-2, 321-336.

Gates D.M. 1980. Biophysical Ecology.Springer, New York.

IPCC, 2013. Fifth Assessment Report of the Intergovernmental Panel in Climate Change

Jarvis PG, KGMcNaughton. 1986. Stomatal control of transpiration: scaling up from leaf to region. Adv. Ecol. Res. 15, 1–49.

Keenan T, S. Sabaté, C. Gracia. 2010. Soil water stress and coupled photosynthesis–conductance models: bridging the gap between conflicting reports on the relative roles of stomatal, mesophyll conductance and biochemical limitations to photosynthesis. Agricultural and Forest Meteorology 150, 443-453.

Keenan T, R. Garcia, AD. Friend, S. Zaehle, C. Gracia, S. Sabaté. 2009a. Improved understanding of drought controls on seasonal variation in Mediterranean forest canopy CO2 and water fluxes through combined in situ measurements and ecosystem modelling. Biogeosciences, 6, 1423-1444.

Keenan T, Ü Niinemets, S Sabaté, C Gracia, J Peñuelas. 2009b. Process based inventory of isoprenoid emissions: current knowledge, future prospects and uncertainties. Atmospheric Chemistry and Physics, 9, 4053–4076.

Keenan T, ÜNiinemets, S Sabaté, C Gracia,JPenuelas. 2009c. Seasonality of monoterpene emission potentials in Quercus ilex and Pinuspinea: implications for regional VOC emissions modeling. Journal of Geophysical Research, 114, D22202,doi: 10.1029/2009JD011904.

Keenan T, D Hollinger, G Bohrer, D Dragoni, JW Munger, HP Schmid, A Richardson. 2013. Increase in forest water-use efficiency as atmospheric carbon dioxide concentrations rise. Nature, 449:324-327

Kramer K, I Leinonen, HH Bartelink, P Berbigier, M Borghetti, C Bernhofer, E Cienciala, AJ Dolman, O Froer, C Gracia, *et al.* 2002. Evaluation of six process-based forest growth models using eddy-covariance measurements of CO_2 and H_2O fluxes at six forest sites in Europe. Global Change Biology, 3-8, 213-230.

Leuning R. 1995. A critical appraisal of a combined stomatal–photosynthesis model for C3 plants. Plant Cell Environ. 18, 339-355.

Monteith JL 1965.Evaporation and environment. Symp. Soc. Exp. Biol. 19, 205–234.

Morales P, MT Sykes, IC Prentice, P Smith, B Smith, H Bugmann, B Zierl, P Friedlingstein, NViovy, S Sabaté*et al.* 2005. Comparing and evaluating process-based ecosystem model predictions of carbon and water fluxes in major European forest biomes. Global ChangeBiology, 11-12, 2211-2233.

Nadal-Sala D, S Sabaté y C Gracia. 2014. GOTILWA+: un modelo de procesos que evalúa efectos del cambio climático en los bosques y explora alternativas de gestión para su mitigación. Ecosistemas 22:29-36

Otero I, M Boada, A Badia, et al. 2011. Loss of water availability and stream diodiversity under land abandonment and climate change in a Mediterranean catchment (Olzinelles, NE Spain). Land Use Policy 28:207-218

Ovington, JD. 1961. Some aspects of energy flow in plantations of Pinussylvestris L. Annals of Botany, 25:12-20

Sabaté S and C Gracia 2011. Water processes in trees: transpiration and photosynthesis, in: Water for Forests and People in the Mediterranean – What Science Can Tell Us, edited by: Birot, Y., Gracia, C. A., and Palahí, M., European Forest Institut, Joensuu, available at: http://www.efi.int/portal/virtual_library/publications/what_science_can_tell_us/, pp 72–75.

Schröter D, W Cramer, R Leemans, IC Prentice, MB Araújo, NW Arnell, ABondeau, H Bugmann, TR Carter, CAGracia, *et al.* 2005. Ecosystem Service Supply and Human Vulnerability to Global Change in Europe. Science, 310 (5732): 1333-1337.

Shinozaki, K., K Yoda, K Hozumi, T Kira. 1964. A quantitative analysis of plant form – the pipe model theory. I. Basic analyses. Japanese Journal of Ecology, 14: 97–105.

Yoda, K. 1963. Self-thinning in overcrowded pure stands under cultivated and natural conditions. Journal of Biology Osaka City University, 14: 107-129.

8. SIMULATION RESULTS FROM iLAND

Rupert Seidl, Werner Rammer

Institute of Silviculture, Department of Forest- and Soil Sciences, University of Natural Resources and Life Sciences (BOKU) Vienna, Austria.

1. METHODS

1.1 FOREST MODEL

Here, we used iLand, the individual-based forest landscape and disturbance model, to evaluate simulated tree mortality and test the sensitivity of the model to an alternative mortality formulation in the context of scenario simulations. iLand is a forest landscape model operating at the grain of individual trees (Seidl et al., 2012a). Tree-level dynamics is simulated based on first principles of tree physiology. iLand is a spatially explicit model, where trees compete for resources based on ecological field theory. Production physiology is simulated based on a light use efficiency approach, accounting for the effect of suboptimal temperature, soil and atmospheric moisture availability, soil nutrient availability as well as atmospheric CO_2 content. Trees adapt their C allocation strategy dynamically to their environment in iLand. Mortality is based on the C balance of trees (see below for details), but also can result from chance and natural disturbance (wind, bark beetles, wildfire). In managed forest landscapes, the complex interaction between forest dynamics and adaptive management interventions is simulated via an agent-based forest management model (Rammer and Seidl, 2015). Regeneration and establishment of trees is simulated spatially explicit at a grain of 2m cells, and accounts for light availability, environmental filters, and seed dispersal on the landscape (Seidl et al., 2012b). In addition to tree demography iLand simulates above- and below ground forest C dynamics. The model is able to address forest dynamics over extents of up to several tens of thousands of hectares at the grain of individual trees, and has been successfully evaluated and applied for forest types in the western US as well as northern and central Europe (Seidl et al., 2014a, 2014b, 2012a, 2012b; Silva Pedro et al., 2015).

1.2 MORTALITY FORMULATIONS

Default mortality: iLand's default mortality algorithm contains two components, stress-related mortality and intrinsic mortality. Intrinsic mortality is age-related, assuming that a fixed share of trees live to reach their maximum age (species parameter) (see also Wunder et al. 2006). The second mortality component relates to stress, with stress being defined as a tree not being able to replace its annual C losses from maintenance respiration through NPP and the carbohydrate reserves pool. A scalar stress index SI [0,1] is calculated that is >0 once the C balance of a tree turns negative (i.e., respiratory losses exceed C gains and reserves), and reaches its maximum value if the C gains and reserves of a tree are zero (Seidl et al., 2012a). Stress-related mortality probability is subsequently derived as a function of SI and an empirically derived parameter. The mortality model was calibrated and tested against theoretical expectations of tree size – density relationships ("self-thinning rule") as well as against observed tree mortality in complex old-growth forest ecosystems (Seidl et al., 2012a). Mortality from disturbance was not considered here and is thus not described in detail.

Alternative mortality: As alternative variant for stress-related mortality, we adopted an approach widely applied by forest vegetation models, using an absolute diameter increment threshold as an indicator for stress (Keane et al., 2001). Once a tree falls below this threshold for a consecutive number of years its mortality probability increases. We here implemented this mortality variant as used in the forest gap model PICUS v1.5 (Seidl et al., 2005), and also applied PICUS species parameters: The dbh increment threshold for *Picea abies* was set to 0.03 cm yr^{-1}, and five consecutive years below this threshold were required to trigger a tree to be stressed. Once stressed, the mortality probability was set for only 10% of the trees surviving ten years of stress. The intrinsic mortality formulation of iLand was left unchanged also in the simulations with the alternate mortality variant.

2. SIMULATION SETTINGS

2.1 SITE DATA

We conducted simulations at three sites in Austria, comprising an elevation gradient from 540 m to 1250 m asl. Site "Eibiswald" (E) is located in southern Austria. It has a mean annual temperature of 5.3°C and a mean annual precipitation sum of 1446 mm (Table 1). The effective soil depth is 149 cm and the plant-available nitrogen is 50 kg N ha^{-1} yr^{-1}. Site "Karlstift" (K) is located in northern Austria. It has a mean annual temperature of 6.0°C and a mean annual precipitation sum of 816 mm. The effective soil depth is 150 cm and the plant-available nitrogen is 45 kg N ha^{-1} yr^{-1}. Site "Ottenstein" (O) is also located in northern Austria. It has a mean annual temperature of 7.3°C and a mean annual precipitation sum of 633 mm. The effective soil depth is 23 cm and the plant-available nitrogen is 62 kg N ha^{-1} yr^{-1}. A mean atmospheric CO_2 concentration of 380 ppm was assumed for all sites under baseline climate.

Climate data for minimum and maximum temperature, precipitation, vapor pressure deficit, and radiation (daily time step) were derived from a downscaled and bias corrected database of gridded climate data for Austria at 1 km resolution. From the same data base, three regionally downscaled climate change scenarios, representing different combinations of global and regional circulation models under A1B forcing, were available: CNRM-RM4.5 driven by the global climate models (GCM) ARPEGE and MPI-REMO, respectively, as well as ICTP-RegCM3 driven by the GCM ECHAM5 . Climate changed transiently in these scenarios until the end of the 21st century (temperature change of between 3.1°C and 3.3°C, and precipitation change of between –89 mm and + 141 mm in 2080-2099 relative to the baseline period). A stabilization of climate conditions at the level of 2080 – 2099 was assumed for the years beyond 2100 in the simulations. The same was assumed for atmospheric CO_2 levels. A detailed overview of the applied climate forcings is given in Tables 2-4.

Table 1. Characterization of the baseline climate (1981-2010) for the three study sites

Scenario		Temperature °C	Precipitation mm	Radiation MJ $m^{-2}d^{-1}$	VPD kPa
**Eibiswald**	MAM	4.74	324.3	16.04	0.21
	JJA	13.51	513.6	21.64	0.51
	SON	5.56	405.9	9.62	0.23
	DJF	-2.87	201.7	5.87	0.11
**Karlstift**	MAM	5.45	184.4	14.75	0.21
	JJA	14.08	309.5	19.82	0.45
	SON	6.37	176.0	8.69	0.24
	DJF	-1.99	145.6	4.75	0.08
**Ottenstein**	MAM	7.06	148.9	15.53	0.30
	JJA	15.89	255.8	20.35	0.64
	SON	7.26	141.2	9.18	0.28
	DJF	-1.20	87.0	5.32	0.09

Table 2: The climate forcing applied at site Eibiswald. Changes are for the period 2080-2099 relative to 1981-2010

		Scenario		
		A1B ARPEGE - CNRM-RM4.5	A1B REMO - CNRM-RM4.5	A1B ECHAM5 - ICTP-RegCM3
**Temperature**	MAM	+2.43	+2.61	+2.93
**(delta °C)**	JJA	+4.89	+3.92	+3.49
	SON	+2.93	+3.19	+2.61
	DJF	+1.76	+3.63	+3.17
**Precipitation**	MAM	-3.19	-3.63	-9.24
**(%)**	JJA	-18.05	-25.50	+1.64
	SON	-14.52	+32.59	+34.63
	DJF	+8.21	+16.93	+17.46
**Radiation**	MAM	+1.97	-2.75	+3.04
**(%)**	JJA	+5.69	+1.86	+7.18
	SON	+8.51	-6.65	-2.65
	DJF	-2.91	-9.17	-10.81
**VPD**	MAM	+28.25	+23.44	+36.46
**(%)**	JJA	+109.6	+53.79	+41.53
	SON	+72.82	+32.12	+13.48
	DJF	+8.82	+32.87	+13.82
**CO_2 (delta ppm)**		+290	+290	+290

Table 3: The climate forcing applied at site Karlstift. Changes are for the period 2080-2099 relative to 1981-2010

		Scenario		
		A1B ARPEGE - CNRM-RM4.5	A1B REMO - CNRM-RM4.5	A1B ECHAM5 - ICTP-RegCM3
**Temperature**	MAM	+2.17	+2.22	+2.84
**(delta °C)**	JJA	+4.59	+3.39	+3.26
	SON	+3.12	+2.86	+2.57
	DJF	+2.05	+3.45	+3.23
**Precipitation**	MAM	+1.80	+1.37	+11.46
**(%)**	JJA	-14.60	-6.97	-6.07
	SON	-5.51	+33.66	+21.81
	DJF	+10.28	+30.42	+24.68
**Radiation**	MAM	+1.50	-5.30	+1.10
**(%)**	JJA	+8.50	+0.99	+10.01
	SON	+5.32	-5.63	-5.66
	DJF	+-3.49	-9.57	-10.07
**VPD**	MAM	+17.41	+14.80	+37.38
**(%)**	JJA	+124.93	+41.85	+43.48
	SON	+85.86	+19.78	+10.00
	DJF	+19.19	+30.76	+24.11
**CO_2 (delta ppm)**		+290	+290	+290

Table 4: The climate forcing applied at site Ottenstein. Changes are for the period 2080-2099 relative to 1981-2010

		Scenario		
		A1B ARPEGE - CNRM-RM4.5	A1B REMO - CNRM-RM4.5	A1B ECHAM5 - ICTP-RegCM3
**Temperature**	MAM	+2.17	+2.17	+2.82
**(delta °C)**	JJA	+4.68	+3.28	+3.23
	SON	+3.03	+2.82	+2.58
	DJF	+1.76	+3.48	+3.20
**Precipitation**	MAM	+2.18	+1.92	+10.87
**(%)**	JJA	-17.85	-6.35	-8.60
	SON	-7.71	+41.82	+28.39
	DJF	+14.73	+38.44	+31.04
**Radiation**	MAM	+1.93	-4.20	+1.28
**(%)**	JJA	+5.53	+0.11	+7.29
	SON	+3.70	-7.31	-6.99
	DJF	-3.30	-9.61	-11.27
**VPD**	MAM	+23.41	+13.23	+35.48
**(%)**	JJA	+107.89	+35.05	+37.92
	SON	+70.95	+18.40	+11.51
	DJF	+17.01	+31.10	+22.72
**CO_2 (delta ppm)**		+290	+290	+290

2.2 *TEST DATA*

For all three sites, replicated observations of tree mortality were available from the control plots of thinning experiments (data courtesy of the Austrian Research Center for Forests, BFW). All three sites are stocked with pure Norway spruce forests. At site K, we had four unmanaged replicates spanning the period 1977-2009. At site O, we were able to use three unmanaged control plots for 1968-2012. At site E we also used three unmanaged control plots for the years 1968-2013. For each of these ten evaluation sites, between eight and eleven observations were made over time. These data are independent data, and have not been used in the parameterization or calibration of the model.

2.3 *SIMULATION EXPERIMENTS*

Evaluation: To test the model against the independent observations of the thinning trials we set up stands using the initial inventory at every site. We ran iLand over the respective observation period, using (i) the default mortality formulation and (ii) the alternate mortality formulation described above. We compared stem number development over time as well as diameter at breast height (dbh) and diameter distribution at the end of the observation period to assess model performance and differences between the two mortality formulations. The evaluation exercise was conducted at stand level (1 ha stands), in order to match the simulation to the observed extent of the reference data.

Scenario analysis: iLand was run at the stand level for this analysis. Seed input from 28 other species was considered. Natural disturbances and management interventions were omitted in the simula-

tions. In addition to a continuation of baseline climate, three climate scenarios were simulated over 200 years, using (i) the default mortality formulation and (ii) the alternate mortality formulation. Simulations were continued from the last observation made for every trial. Analyses were conducted at the level of sites, i.e. the stand replicates were averaged.

3. SIMULATION RESULTS

Evaluation

iLand was generally well able to capture the observed stand development at the three sites investigated. For a wide range of densities the model reproduced the observed stem number development well (Fig. 1). The alternate mortality formulation based on an absolute diameter threshold performed slightly better than the physiologically based iLand default variant, mimicking the observed decrease in stem numbers very closely (Fig. 2).

For very dense and very sparse stands stem numbers at the end of the simulation were moderately underestimated and overestimated, respectively by the default variant (Fig. 3). The simulated mean dbh at the end of the simulation period matched the observations well (Fig. 2). In the alternate variant, higher stem numbers were simulated accurately, yet sparsely stocked stands were still somewhat overestimated at the end of the evaluation period (Fig. 4). The estimated diameters at the end of the simulation period matched observations equally well as in the iLand default variant, yet with a slight underestimation of diameters in denser stands (Fig. 4). Overall, there were only marginal differences in the simulated diameter distributions between the two mortality model variants at the end of the observation period (Fig. 5).

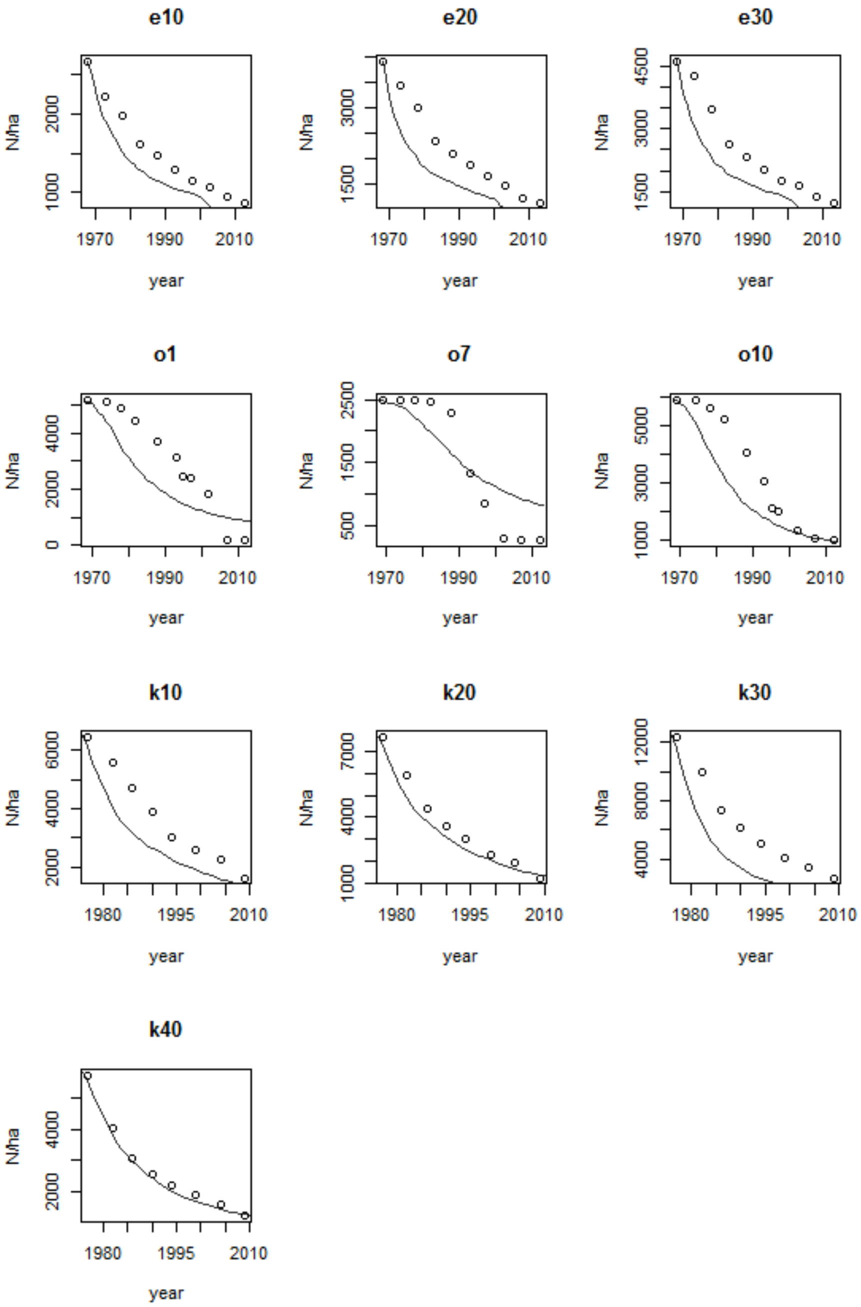

Figure 1: Simulated (line) and observed (dots) stem number at the three sites Eibiswald (E), Ottenstein (O) and Karlstift (K) using the iLand default mortality formulation. Every panel is one replicated observation of an unmanaged forest stand at the given site

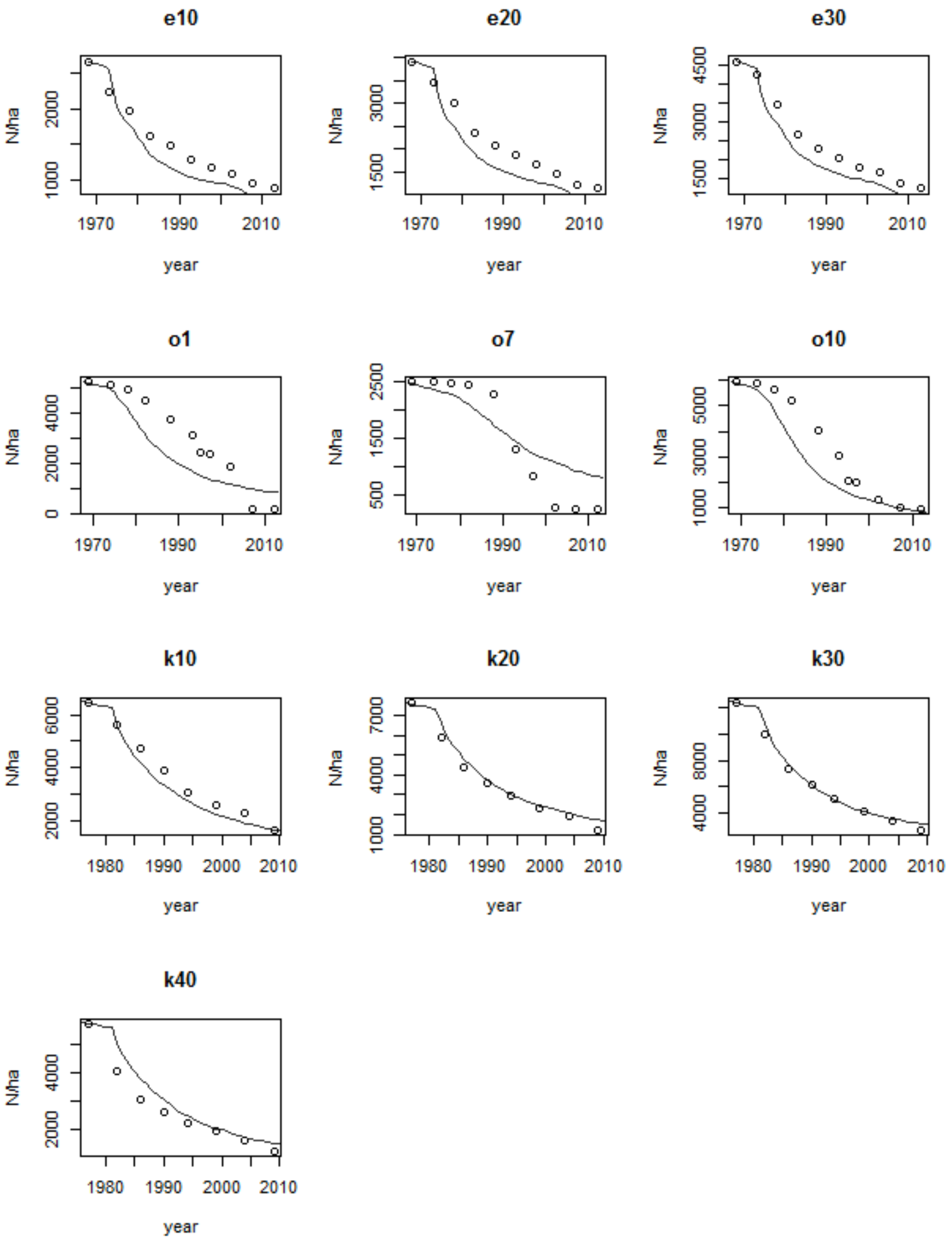

Figure 2: Simulated (line) and observed (dots) stem number at the three sites Eibiswald (E), Ottenstein (O) and Karlstift (K) using the alternate mortality formulation. Every panel is one replicated observation of an unmanaged forest stand at the given site

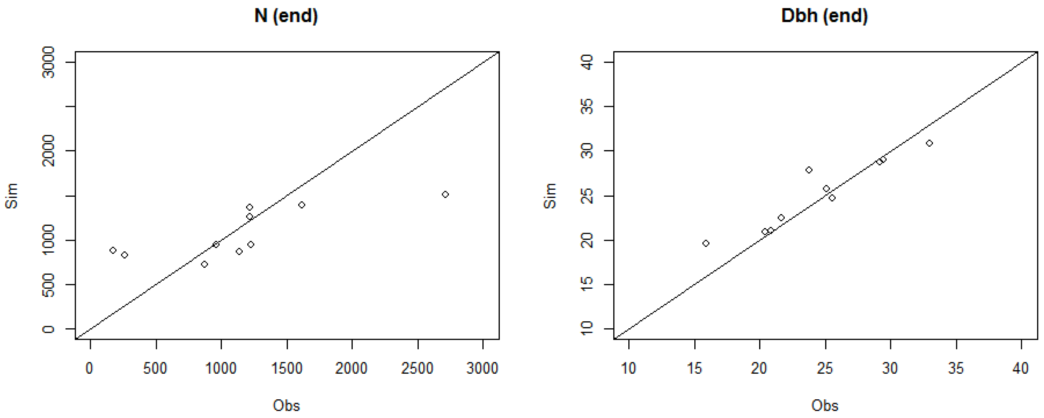

Figure 3: Simulated versus observed stem number and mean diameter across all sites at the end of the observation period, simulated using the iLand default mortality formulation.

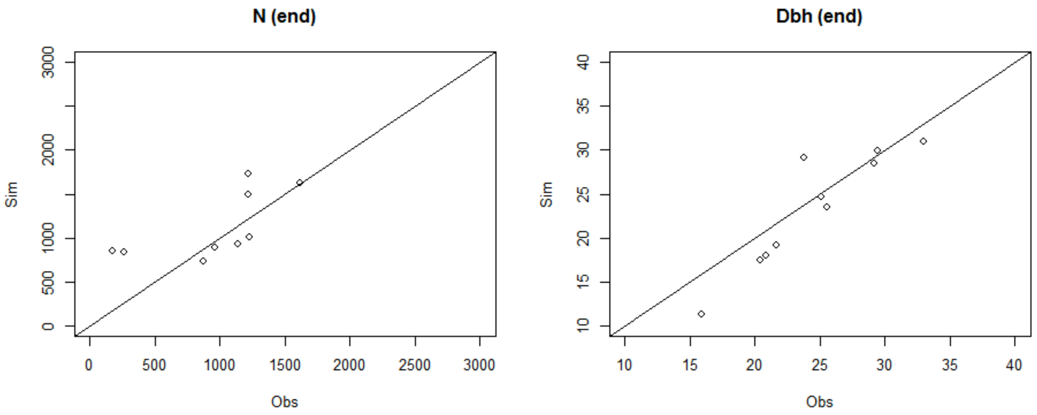

Figure 4: Simulated versus observed stem number and mean diameter across all sites at the end of the observation period, simulated using the alternate mortality formulation

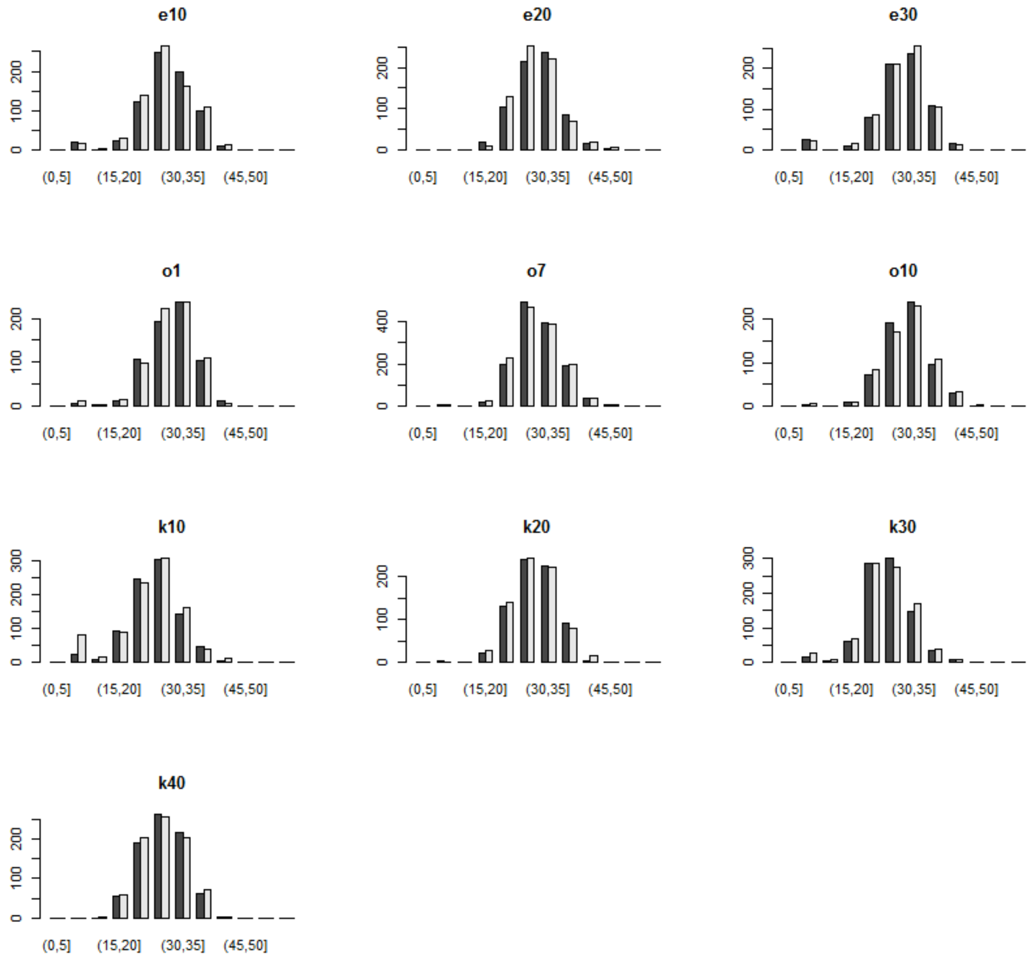

Figure 5: Simulated diameter distributions at the end of the evaluation period, using the iLand default and alternate mortality formulations. Every panel is one replicated observation of an unmanaged forest stand at the given site (E: Eibiswald, O: Ottenstein, K: Karlstift)

Scenario analysis

The effect of the different mortality variants increased with decreasing elevation, i.e. it was highest at the warm and dry low elevation site Ottenstein and lowest at the cool and wet high elevation site Eibiswald (Figure 6). At Ottenstein, where the dominant species Norway spruce is most stressed, mortality and regeneration was characterized by an episodic pattern, whereas the development over time at Eibiswald and Karlstift was predominately smooth (Figure 7). At Eibiswald and Karlstift stem numbers were found to decrease over the first half of the simulation period and increase in the later portion of the 200 years simulated, a pattern that was unaffected by the different mortality formulations. At Ottenstein, however, the carbon starvation-based mortality formulation gave generally lower stem numbers and basal areas compared to the alternate mortality formulation. Moreover, the pattern of mortality and regeneration showed stronger temporal variation in the default variant compared to that of the alternate variant. This is also reflected in the simulated tree species composition at Ottenstein. In all four climate scenarios, the share of Norway spruce is declining (Figure 8). Yet, under the baseline mortality formulation episodic spruce regeneration years are simulated, which is not the case for the alternate mortality formulation. In general, regeneration of Norway spruce becomes more episodic with climate change in all scenarios, and the share of the species in lower dbh classes decreases in all climate scenarios at all sites, compared to baseline climate (Figure 9).

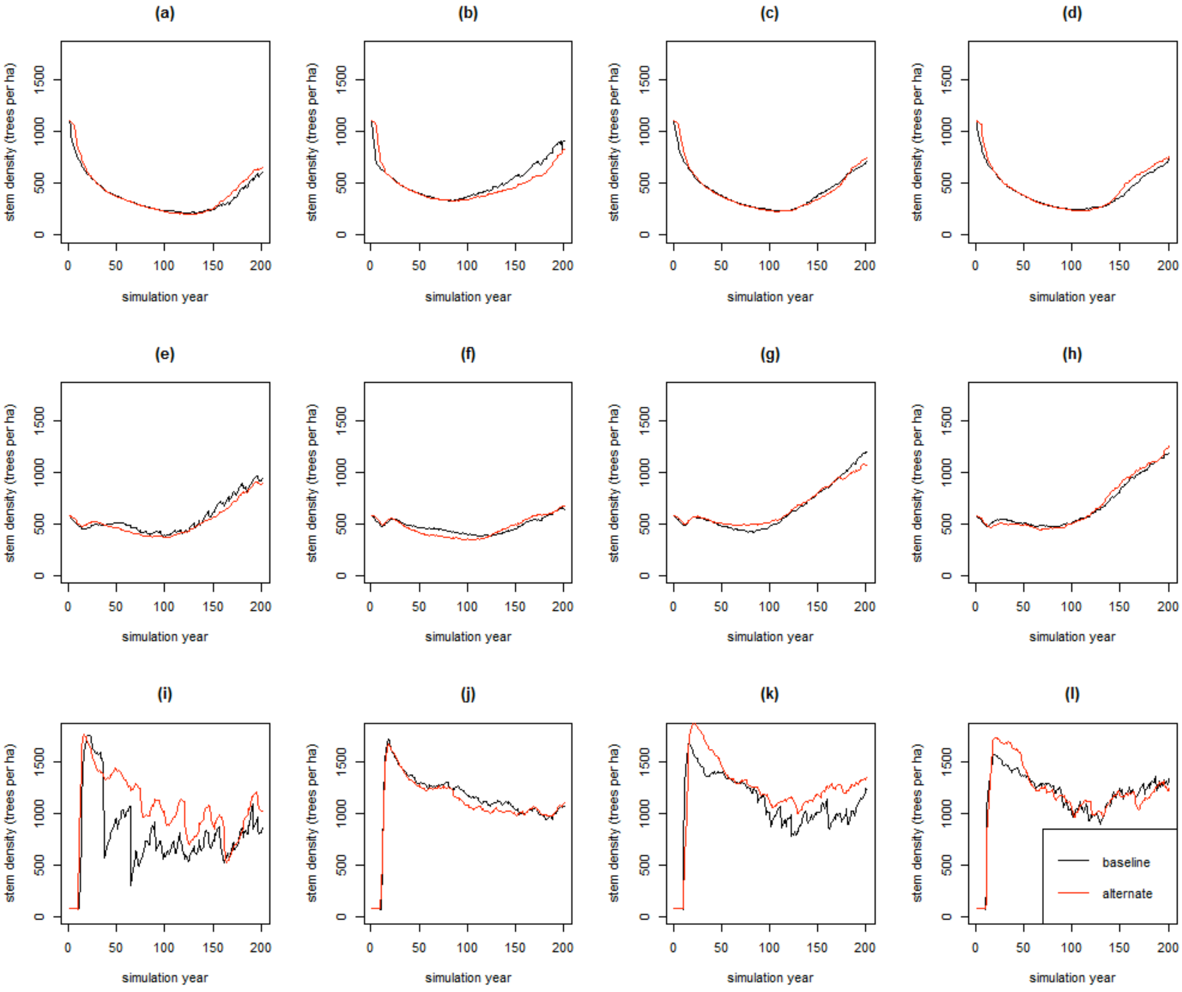

Figure 6: Stem number trajectories for the three landscapes (rows) in four different climate scenarios (columns) simulated using the iLand default (black) and alternate (red) mortality formulations. Top row: Eibiswald (panels a-d), center row: Karlstift (panels e-h), bottom row: Ottenstein (panels i-l). First column: baseline climate (panels a, e, i), second column: scenario A1B-ARPEGE-CNRM-RM4.5 (panels b, f, j), third column: scenario A1B-ECHAM5-ICTP-RegCM3 (panels c, g, k), fourth column: scenario A1B-MPI-REMO-CNRM-RM4.5 (columns d, h, l)

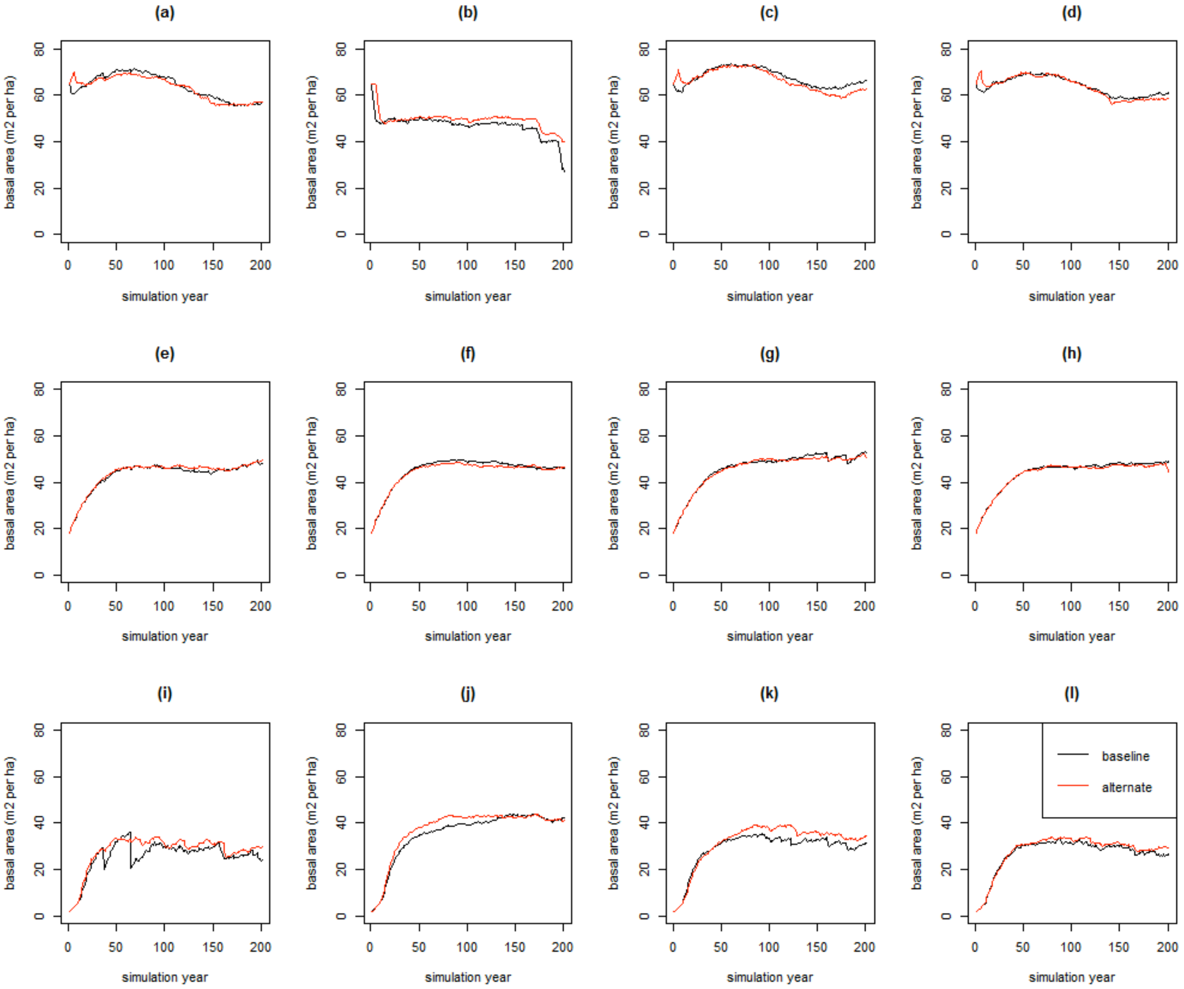

Figure 7: Basal area trajectories for the three landscapes (rows) in four different climate scenarios (columns) simulated using the iLand default (black) and alternate (red) mortality formulations. Top row: Eibiswald (panels a-d), center row: Karlstift (panels e-h), bottom row: Ottenstein (panels i-l). First column: baseline climate (panels a, e, i), second column: scenario A1B-ARPEGE-CNRM-RM4.5 (panels b, f, j), third column: scenario A1B-ECHAM5-ICTP-RegCM3 (panels c, g, k), fourth column: scenario A1B-MPI-REMO-CNRM-RM4.5 (columns d, h, l)

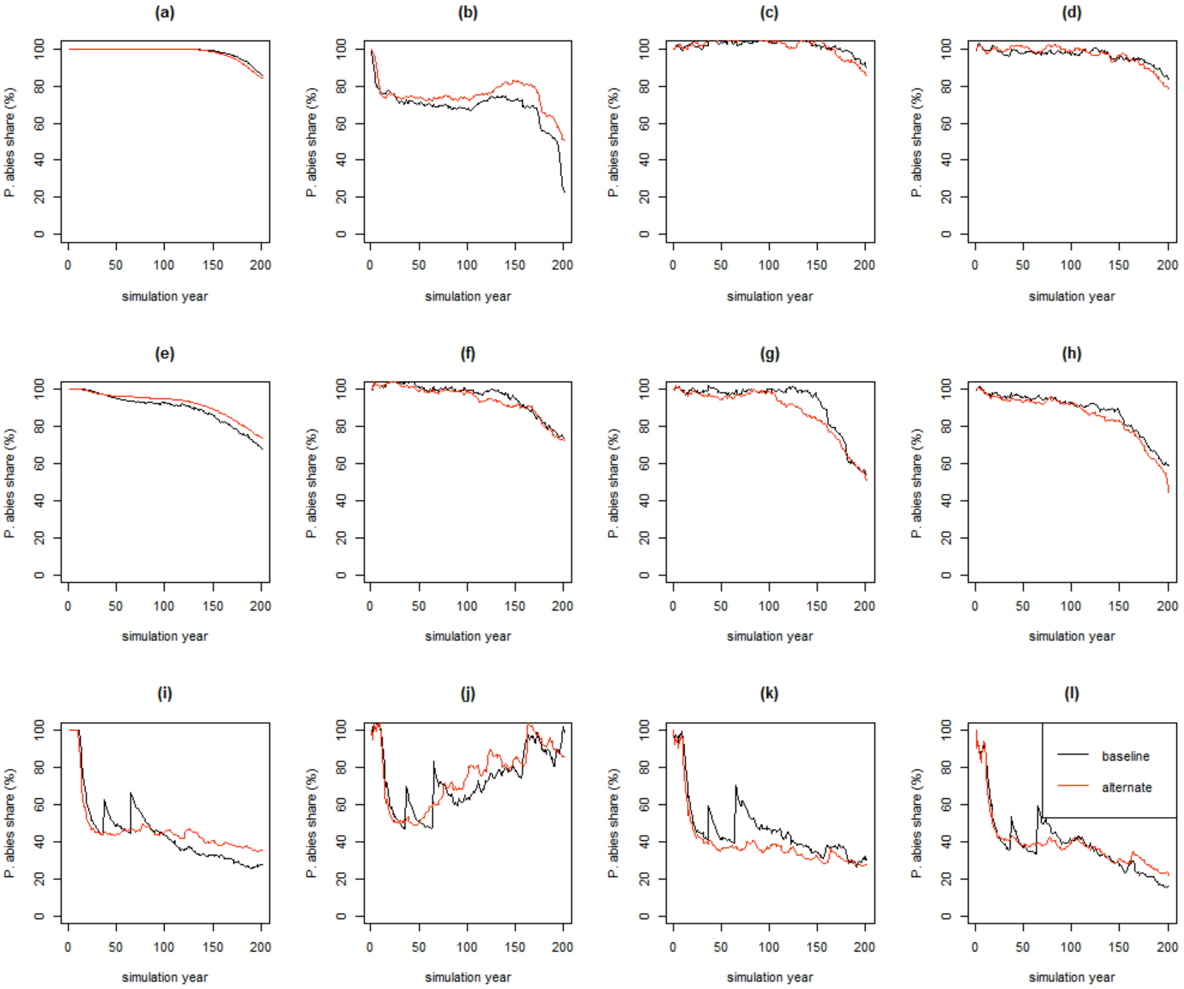

Figure 8: Share of Norway spruce (Picea abies (L.) Karst.) in the three landscapes (rows) in four different climate scenarios (columns) simulated using the iLand default (black) and alternate (red) mortality formulations. Top row: Eibiswald (panels a-d), center row: Karlstift (panels e-h), bottom row: Ottenstein (panels i-l). First column: baseline climate (panels a, e, i), second column: scenario A1B-ARPEGE-CNRM-RM4.5 (panels b, f, j), third column: scenario A1B-ECHAM5-ICTP-RegCM3 (panels c, g, k), fourth column: scenario A1B-MPI-REMO-CNRM-RM4.5 (columns d, h, l)

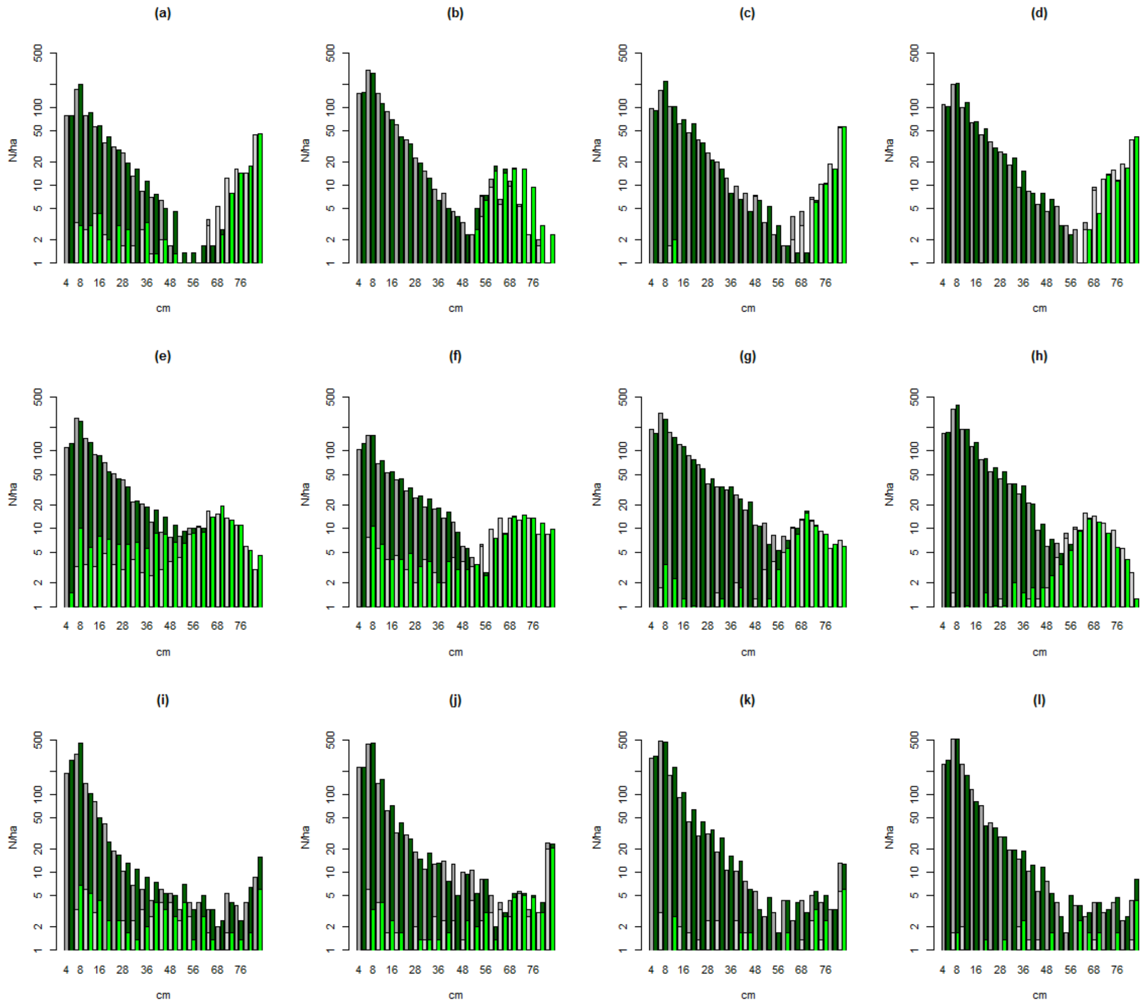

Figure 9: Dbh distribution in year 200 in the three landscapes (rows) in four different climate scenarios (columns) simulated using the iLand default (greyss) and alternate (greens) mortality formulations. Light hues (light gray, light green) are P. abies, dark hues (dark grey, dark green) are other species. Top row: Eibiswald (panels a-d), center row: Karlstift (panels e-h), bottom row: Ottenstein (panels i-l). First column: baseline climate (panels a, e, i), second column: scenario A1B-ARPEGE-CNRM-RM4.5 (panels b, f, j), third column: scenario A1B-ECHAM5-ICTP-RegCM3 (panels c, g, k), fourth column: scenario A1B-MPI-REMO-CNRM-RM4.5 (columns d, h, l)

Mortality rates generally increased in the second 100-year period of the simulation, mainly as a result of initializing the model with timber stage stands. An analysis of mortality rates shows clearly that higher mortality rates are simulated under the default C starvation mortality compared to the alternate diameter threshold model (Figure 10). This difference is particularly visible for sites and periods where mortality is generally high in both variants. This behavior is in line with the evaluation experiment conducted above (Figures 1-4), in which the default mortality formulation was found to simulate considerably higher mortality in dense stands as the alternative formulation. The differences here are again strongest for the low and mid elevation sites. Climate change showed a tendency to increase mortality at the high elevation site Eibiswald, where growth is increased through a warming climate and competitive pressure on trees is increased. At the mid and low elevation sites climate change showed a tendency to decrease mortality, in response to alleviated competition for light due to decreased productivity. This effect was considerably stronger in the C starvation based variant. Generally, differences between variants and scenarios were much stronger for mortality than for state variables at the end of the 200 year simulation (Figure 11).

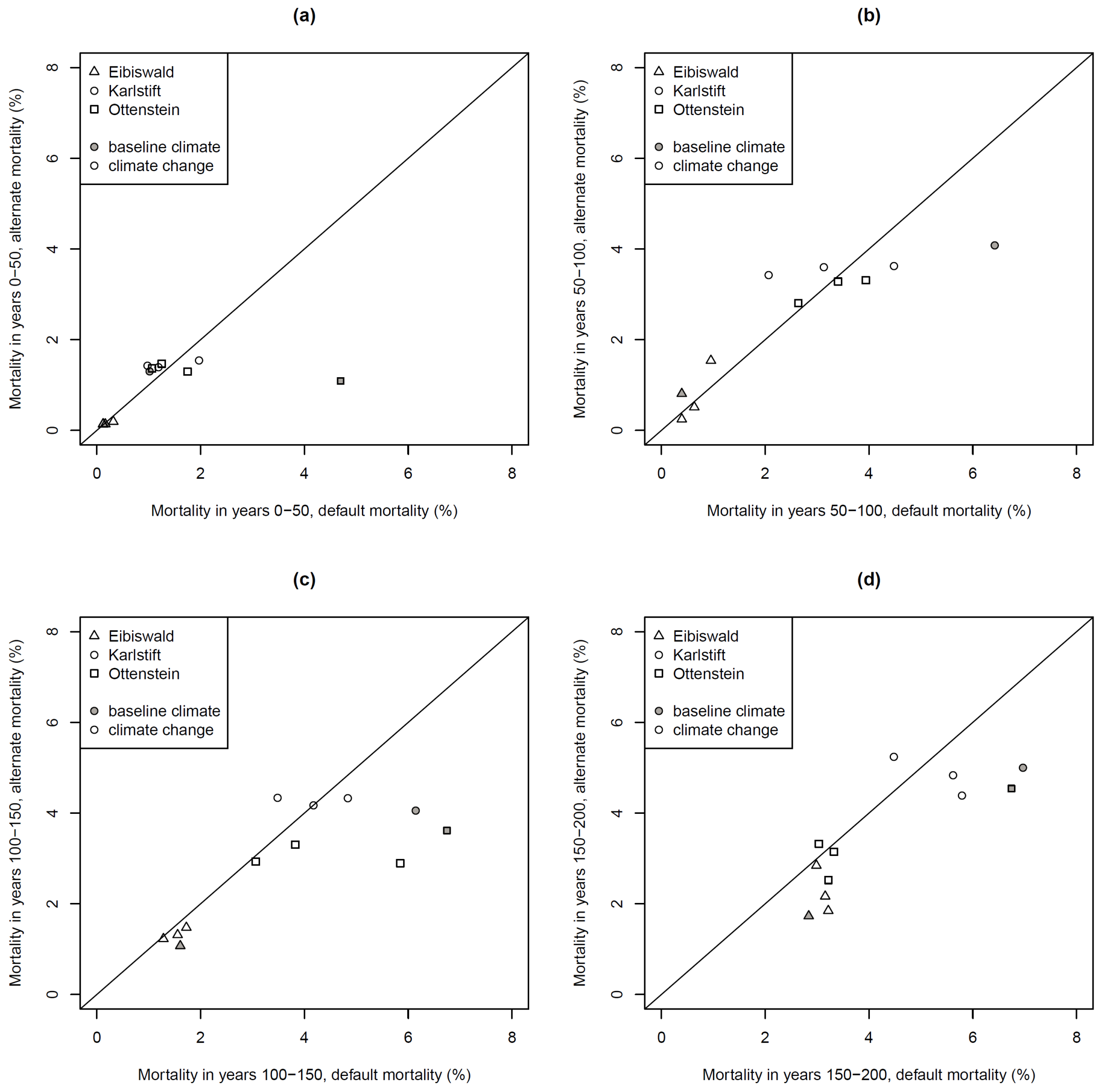

Figure 10: Mortality rates (%) for the two mortality formulations and the simulated climate and site combinations for the years 0-49 (a), 50-99 (b), 100-149 (c), 150-200 (d)

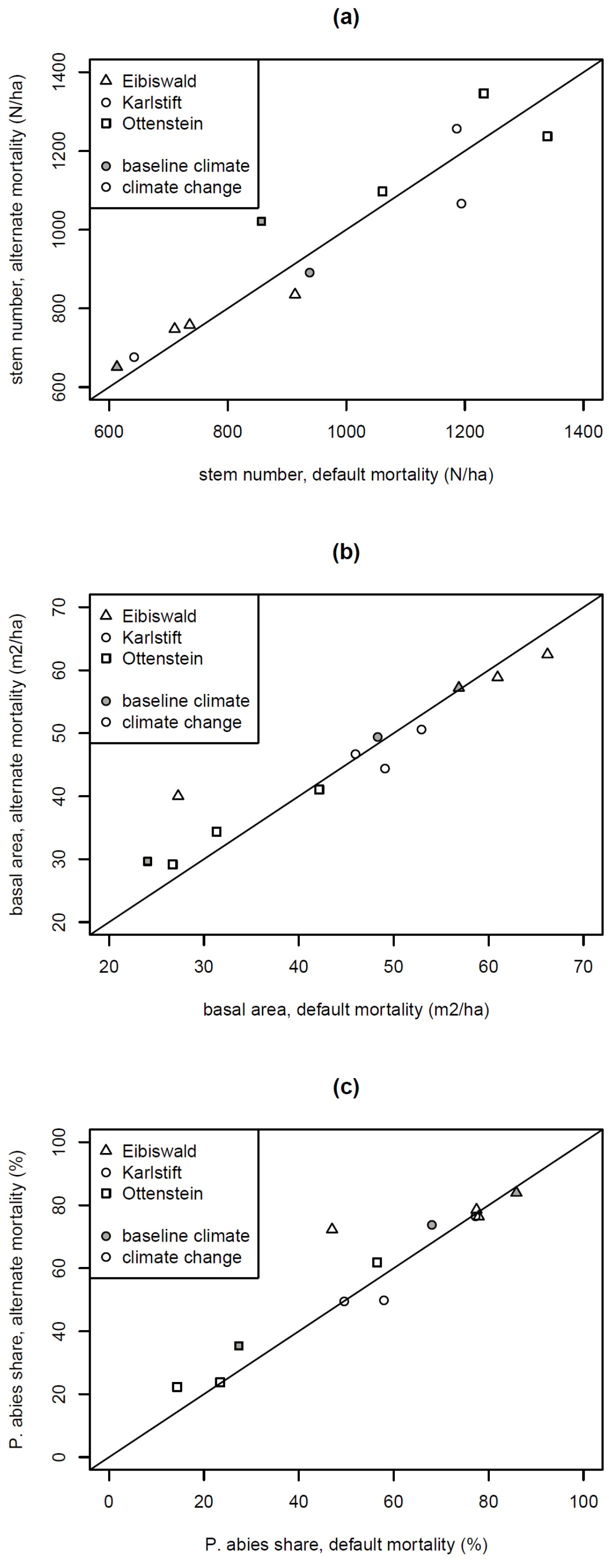

Figure 11: Comparison over sites and scenarios at the end of the 200 year simulation period: (a) stem number, (b) basal area, (c) share of Norway spruce on total basal area

4. Discussion

We conclude that both a theoretical mortality model widely used in gap models (Keane et al., 2001) and a physiologically-based mortality model of C starvation (Güneralp and Gertner, 2007; Seidl et al., 2012a) worked well within the framework of an individual-based forest landscape model. In a multi-decadal evaluation experiment across a wide environmental gradient both models were well able to reproduce observed mortality patterns, with a slight advantage for the alternate mortality variant. As diameter growth is usually of low priority in the C allocation scheme of trees, a hierarchy that is also considered in the individual tree C balance in iLand, the two indicators can be expected to be strongly correlated, leading to comparable results in the simulation.

Yet, some interesting differences between the two mortality variants emerged: We found that mortality effects in general and the sensitivity of the model to different mortality formulations in particular were strongly site dependent. At sites where Norway spruce is in the core of its realized niche (e.g., at Eibiswald) different formulations of stress-related mortality had only very little effect. Closer to the edge of a species' niche, however, different ways of modeling stress had a clearly visible effect. Here, the C balance variant showed higher sensitivity than the diameter increment threshold variant, leading to higher mortality rates and more episodic mortality patterns in the former variant at the site Ottenstein. Despite the fact that the diameter increment threshold is crossed before the C starvation threshold in the model for any given tree, the built-in delay for mortality to set in in the alternate variant seems to considerably buffer the system compared to the default variant. Of the investigated state variables species composition reacted most sensitive to different mortality formulations and climate scenarios.

We conclude that the effect of alternative mortality formulations differs on the what and where: Comparing mortality formulations that rely on functionally highly correlated indicators of stress (such as diameter increment and C starvation) not surprisingly also yields in only moderate sensitivity of simulation results to these different model variants. Nonetheless, at more extreme sites and towards the edges of a species' niche even similar formulations start to show significance divergence in simulated trajectories. We thus conclude that particularly at ecotones and/ or for the simulation of species distribution uncertainty in mortality model formulations should be a key concern for dynamic vegetation modeling.

5. References

Güneralp, B., Gertner, G., 2007. Feedback loop dominance analysis of two tree mortality models : relationship between structure and behavior. Tree Physiol. 269–280.

Keane, R.E., Austin, M., Field, C., Huth, A., Lexer, M.J., Peters, D., Solomon, A., Wyckoff, P., 2001. Tree mortality in gap models: Application to climate change. Clim. Change 51, 509–540.

Rammer, W., Seidl, R., 2015. Coupling human and natural systems: Simulating adaptive management agents in dynamically changing forest landscapes. Glob. Environ. Chang. in press.

Seidl, R., Lexer, M.J., Jäger, D., Hönninger, K., 2005. Evaluating the accuracy and generality of a hybrid patch model. Tree Physiol. 25, 939–51.

Seidl, R., Rammer, W., Blennow, K., 2014a. Simulating wind disturbance impacts on forest landscapes: Tree-level heterogeneity matters. Environ. Model. Softw. 51, 1–11.

Seidl, R., Rammer, W., Scheller, R.M., Spies, T.A., 2012a. An individual-based process model to simulate landscape-scale forest ecosystem dynamics. Ecol. Modell. 231, 87–100.

Seidl, R., Rammer, W., Spies, T.A., 2014b. Disturbance legacies increase the resilience of forest ecosystem structure, composition, and functioning. Ecol. Appl. 24, 2063–2077.

Seidl, R., Spies, T.A., Rammer, W., Steel, E.A., Pabst, R.J., Olsen, K., 2012b. Multi-scale drivers of spatial variation in old-growth forest carbon density disentangled with Lidar and an individual-based landscape model. Ecosystems 15, 1321–1335.

Silva Pedro, M., Rammer, W., Seidl, R., 2015. Tree species diversity mitigates disturbance impacts on the forest carbon cycle. Oecologia 177, 619–630.

Wunder, J., Bigler, C., Reineking, B., Fahse, L., Bugmann, H., 2006. Optimisation of tree mortality models based on growth patterns. Ecol. Modell. 197, 196–206.

9. SIMULATION RESULTS FROM LANDCLIM

Björn Reineking

UNIV. GRENOBLE ALPES, IRSTEA, UR LESSEM, BP 76, 38402 ST-MARTIN-D'HÈRES, FRANCE &
UNIVERSITY OF BAYREUTH, BAYCEER, 95447 BAYREUTH, GERMANY

1. METHODS

1.1 FOREST MODEL

LandClim is a spatially-explicit forest landscape model that was developed to assess the importance of climatic effects, wildfire and management on historical and future forest dynamics. It consists of a local vegetation model that simulates forest succession, and a landscape model that simulates processes such as fire, wind, forest pest outbreaks, forest management and seed dispersal.

LandClim operates on long time scales (hundreds to thousands of years) and large spatial extents (e.g. 30 km2) at a relatively fine scale (grid cells of 25 by 25 m), and has been tested and adapted to the European Alps, North American Rocky Mountains, and Mediterranean forests" [http://www.fe.ethz.ch/research/disturbance/landclim/index_EN, accessed 6 November 2015]. Introductory references are Schumacher et al. (2004), Schumacher & Bugmann (2006), Schumacher et al. (2006).

1.2 MORTALITY FORMULATIONS

Three alternative mortality formulations were employed:

1. Constant annual mortality rate
2. Growth-dependent annual mortality rate, i.e. logit(survival probability) = beta0 + beta1 * growth_reduction_factor. Where beta0 represents the logit of the annual survival probability for a growth reduction factor of 0, i.e. zero growth, and beta1 represents the change in logit survival probability per unit change in the growth reduction factor. The LandClim growth reduction factor ranges between 0 (no growth) and 1 (maximum growth attainable at the given tree biomass), and depends on light availability, water availability and degree days.
3. Combination of constant mortality rate and stress related mortality (the default formulation in LandClim version 1.6). For each year where the growth reduction factor is below a species specific threshold, a counter of "slow growth years" is increased by 1; if growth is above the threshold, this counter is reset to zero. If the number of slow growth years reaches a species specific threshold, stress mortality is calculated as (1.0 - pow(0.01, 0.1)) * (1.0-GrowthRF/thresholdGrowthReduction). The constant base mortality rate and the stress mortality rate are combined by a maximum function, i.e. only the value that is higher is taken into account.

Note that the constant annual mortality rate (1) is a special case of both alternatives (2) and (3).

For the simulations, we used the current standard parameterization of mortality formulation (3); parameter values are courtesy of Rebecca Snell. For the mortality formulations (1) and (2), parameter values were calibrated to data from the 2008 French National Forest Inventory (IFN). The IFN is a network of non-permanent forest plots. It reports for the selected individual sites information such as the species of individual trees; whether they are alive, have likely died in the past 5 years, or likely died earlier; for alive trees the radial increment for the past 5 years based on increment cores.

In a first step, we selected those 7 species for which the current LandClim version 1.6 has been parameterized that rank highest in basal area in the 2008 French National Forest Inventory. These species are Picea abies, Fagus silvatica, Fraxinus excelsior, Quercus petraea, Abies alba, Pinus sylvestris, and Quercus pubescens. In a second step, we selected those IFN sites where these species accounted for at least 99% of the basal area, resulting in 943 sites. The calibration used LandClim predicted growth reduction factors during the 5 years from 2004-2008; predictions used a constant soil depth, i.e. water holding capacity of 10 cm; climate information was taken from the "ENSEMBLES daily gridded observational dataset for precipitation, temperature and sea level pressure in Europe called E-OBS" [http://www.ecad.eu/download/ensembles/download.php]. The runs were initialized based on the IFN data; the dbh was backcalculated to year 2004 based on the 2008 dbh and the radial growth increment; recently dead trees were assumed to have been alive in 2004 and to have had in 2004 the same dbh that was reported for them in 2008. The model parameters were fitted with maximum likelihood (Figure 1). Elevation data were taken from worldclim at 30" resolution [http://www.worldclim.org]. For exposition and slope we used values from the IFN.

Based on the AIC criterion, for all 7 species model (2) would be preferred over the simple constant mortality rate (1). This is remarkable given that we used as predictor in (2) not the observed growth [which on the one hand would have been missing for the dead trees but, more importantly, would have presented the problem that differences between observed and simulated growth might lead to a mismatch between observed and simulated mortality rates], but rather simulated growth from LandClim – using the standard LandClim parameters and rather rough estimations of the environmental conditions at the sites.

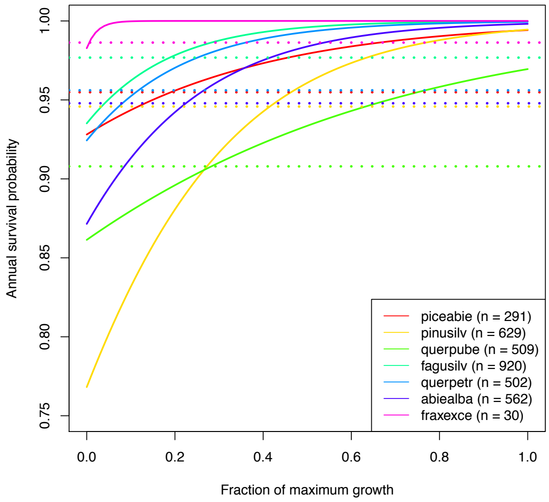

Fig 1: Fitted survival curves for the seven selected tree species based on 2008 French National Forest Inventory Data

2. SIMULATION SETTINGS

2.1 *SITE DATA*

Simulations were run for 119 sites of the 2008 IFN data situated in the region Rhône-Alpes. Current climate data were E-OBS for the period 1981-2010 (see above); climate change scenarios were created by adding anomaly values for monthly precipitation and mean temperature to the E-OBS data. These anomalies were calculated from CMIP5 scenarios at 2.5 minutes spatial resolution accessed from worldclim.org; we used two representative concentration pathways (RCPs), i.e. rcp 26 and rcp 85: as GCM we used MPI-ESM-LR. From the provided time periods: 2050 (average for 2041-2060) and

2070 (average for 2061-2080) we calculated annual changes for the time period 2000 to 2100 relative to the corresponding 2.5 minutes spatial resolution worldclim data by linear interpolation and added these anomalies to the E-OBS data. We assumed that worldclim data represent the year 1975. For the period 2100 to 2200 we assumed that no further climate change takes place. E-OBS data for 1981 to 2010 were used as the basis and recycled to yield 200 years of climate data input.

Other site parameters used were the same as those used in the calibration of the mortality models (1) and (2) (see above).

2.2 TEST DATA

No tests of model performance under current conditions were performed.

2.2 SIMULATION EXPERIMENTS

Simulations were initialized from measured data (2008 state). Simulations were run until 2200, for three climate scenarios: no change, rcp 26 and rcp 85 based on the GCM MPI-ESM-LR (see above).

At each of the 119 sites in the Rhône-Alpes region, simulations were run for a ca. 10 ha site (specifically, 144 replicates of 26.58 m pixels - IFN plots are circular with 15 m radius).

Table 1: Current climatic conditions and climate change scenarios (change calculated for the reference period 2071-2100 relativ to 1981-2010; temperature change in °C, precipitation change expressed as relative change [unitless])

	Temperature (°C)				Monthly precipitation sum (mm)			
	DJF	MAM	JJA	SON	DJF	MAM	JJA	SON
**Observed (1981-2010)**	2.0	8.9	18.1	10.3	219	240	189	303
**RCP2.6**	+4	+3.8	+5.2	+3.9	1.07	1.15	0.83	0.96
**RCP8.5**	+5.9	+5.7	+9.5	+7.2	1.15	0.94	0.52	0.87

3. SIMULATION RESULTS

The three model formulations result in markedly different forest dynamics. Already under prolonged current conditions, the constant mortality model (1) leads to consistently different trajectories relative to the two other mortality formulations.

Under strong climate change scenarios, the mortality formulations (1) and (2) are more similar to each other than to formulation (3): (1) and (2) project, averaged across the 3 sites, a constant or decreasing biomass, whereas (3) still projects strong biomass growth.

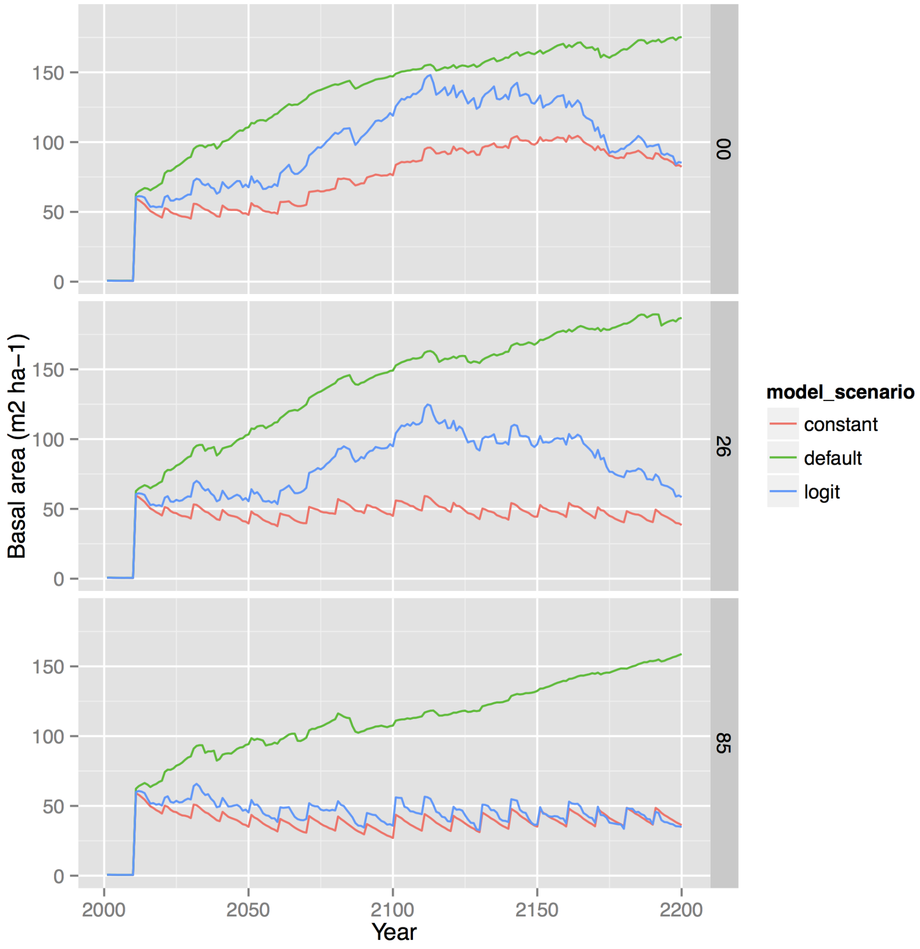

Figure 2: Basal area dynamics. Fluctuations at decadal intervals are due to the regeneration process in LandClim; new cohorts are only added every ten years. This effect is stronger in years with overall low basal area; in years with high basal area, regeneration is limited by shading effects

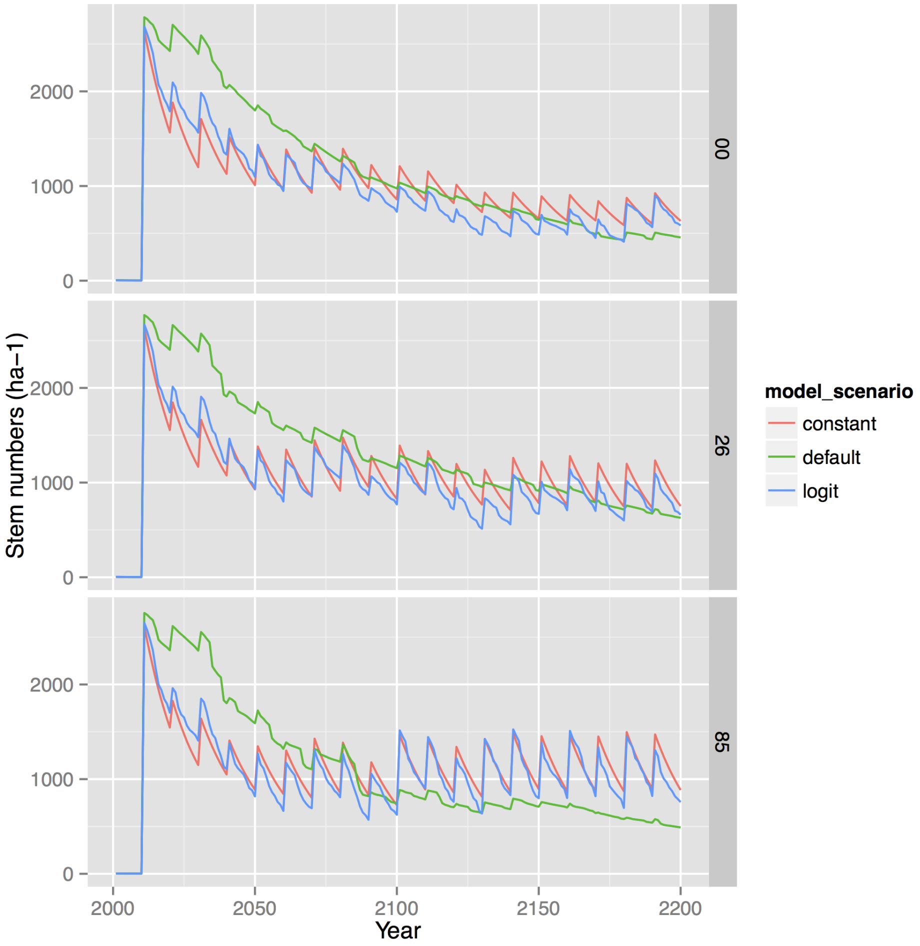

Figure 3: Stem number dynamics. See figure 2 for an explanation of the "sawtooth" pattern in stem numbers due to decadal recruitment

4. REFERENCES

Schumacher, S., H. Bugmann, and D. J. Mladenoff. 2004. Improving the formulation of tree growth and succession in a spatially explicit landscape model. Ecological Modelling 180:175-194.

Schumacher, S. and H. Bugmann. 2006. The relative importance of climatic effects, wildfires and management for future forest landscape dynamics in the Swiss Alps. Global Change Biology 12:1435-1450.

Schumacher, S., B. Reineking, J. Sibold, and H. Bugmann. 2006. Modeling the impact of climate and vegetation on fire regimes in mountain landscapes. Landscape Ecology 21:539-554.

10. Simulation results from LANDIS-II

Josef Brůna[1], Jan Wild[1], Brian R. Miranda[2]

[1]Institute of Botany, The Czech Academy of Sciences, Czech Republic

[2]USDA Forest Service, Northern Research Station, Rhinelander, WI USA

1. Methods

1.1 *Forest model*

We are using LANDIS-II (Scheller et al., 2007) with a modified version of the Biomass Succession extension that is still in development phase. Our modifications add tracking of tree characteristics based on allometric equations fitted to our field data. This additional information will be further used by other extensions developed for our region. In this simulation exercise we compared mortality definition used in Biomass Succession extension v3.0 with a modified one that substitutes age related mortality with height related mortality.

For the purpose of mortality modeling, we have used a single-cell testing tool LANDIS-II-Site(Miranda 2012). This tool (previously called SiteVegCalculator) is designed to simulate succession on a single site using the same shade, establishment and growth algorithms that are used in LANDIS-II succession extensions. It enables exploration of the site level processes without the added complexity of spatial processes on the landscape level in the full LANDIS-II model. For the same purpose, it does not include disturbances including stress related mortality.

1.2 *Mortality formulations*

In LANDIS-II, mortality processes are included in the succession extensions. We have used the Biomass Succession extension v3.0 with two types of mortality, which are described in detail in Scheller & Mladenoff (2004):

1) Growth related mortality - This includes mortality of individual trees or limbs within an age cohort. It is designed not to exceed Annual net potential productivity (ANPP). It increases logistically with increasing biomass until reaching equilibrium with ANPP (Fig 1 a).

2) Age related mortality – is modeled as an exponential increase in mortality with cohort age. It reaches 100% at species longevity (Fig 1 b)

$$\mathrm{M}_{Age} = B\,\frac{e^{age/\max\_age \times d}}{e^{d}}$$

Where d is a mortality shape parameter that ranges 5 -25 and we have used 15. M_{Age} ranges from 0 to 1.

Disturbance related mortality is included in other extensions including drought/stress mortality but none of them was used here. This has large impact on assessment of the role of climate, compared to models, where drought is included in the general mortality equations.

Our modified Biomass succession extension (unpublished) includes age related growth of tree height and allometric equations (height ~ DBH), which allowed us to apply other formulations of age related mortality. We have implemented height related mortality, following principles described in Garcia (2009), who argues that size would dominate over any ageing effects, especially in trees, where meristems are constantly renewed.

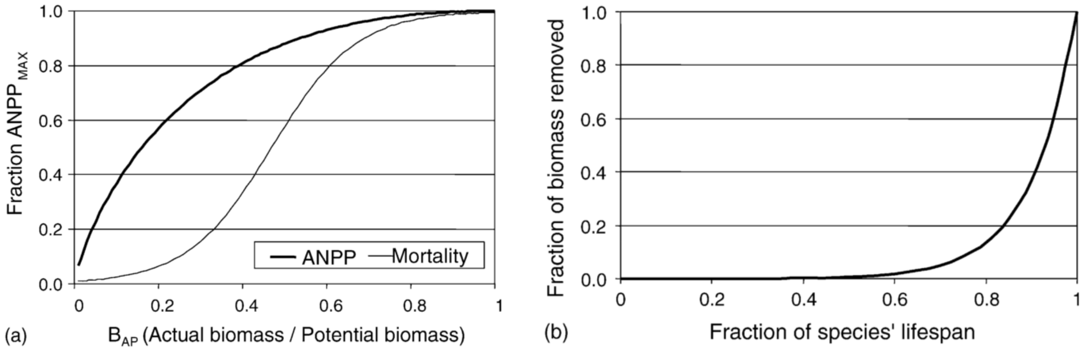

Fig. 1 Two types of mortality included in the Biomass Succession extension v3.0 of LANDIS-II. Figure from Scheller & Mladenoff (2004)

Our second reason for this choice comes from the fact, that climate has no impact on the Age related mortality in the original Biomass succession extension. According to Pretzsch et al. (2014), climate only changes ANPP, which will have effect on the Growth related mortality.

We have used the same equation, but since height is not linear, we have added a height mortality modifier that follows similar exponential curve as age related mortality and is rescaled (numerator) to fit the same range 0-1, meaning cohort will reach 100% height related mortality at species longevity. H_{Height} is a mortality for a given tree height calculated as a biomass to be removed, where B is the current biomass of the cohort. Max_*height* is a maximum height of the species and *height_at_longevity* is height calculated for a tree at age of longevity, which is slightly lower than max_*height*.

$$\mathrm{M}_{Height} = B\,\frac{e^{height/\max\_height \times d}}{e^{d}} \times \frac{\max\_height - height\_at\_longevity}{\max\_height - height}$$

Here, the parameter *d* is again mortality shape parameter that ranges 5 -25. We have used 10, to fit the original biomass curve of age related mortality in unaltered conditions. We have not modified the Growth related mortality in any of the simulations.

2. SIMULATION SETTINGS

2.1 SITE DATA

Modelled site is 1 ha of naturally monospecific spruce forest at 1250-1300 m a.s.l. in non-intervention zone of the National park Šumava – Trojmezná region. Species (Norway Spruce *(Picea abies)*) and site parameters were derived from our field surveys in NP Šumava and NP Bavarian forest and from Svoboda (2005) (Table 1.). Maximum site biomass and ANPP was taken from the most productive site at the same elevation. Allometric equations have been calibrated based on our extensive field data (Macek et al. 2017).

We used climate data from CORDEX project (2 GCM and 2 related RCM) and two RCP (4.5 and 8.5) to illustrate expected climate changes in the region, but climate data were not used explicitly in the model.

Table 1. Site and species parameters used for current and future conditions

	Current conditions	Future conditions
**Maximum tree height**	30 m	
**Maximum biomass for Spruce**	280 000 kg/ha	
**Maximum site biomass**	294 000 kg/ha	
**Max ANPP (annual net primary productivity)**	7 000 kg/ha	9 400 kg/ha (increase in growth rates by 20%)
**Species longevity**	300 years	
**Establishment probability**	0.5	
**Tree count and basal area for verifica-tion.**		
**Allometric growth speed modifier**		1.2 (increase in growth rates by 20%)
**Random number seed for climate**	1	1000

2.2 *Test data*

Resulting tree numbers and basal area were compared to Svoboda (2005) to make sure that allometric equations produce plausible cohort/site characteristics. Biomass succession model was parameterized using ANPP and biomass, but basal area and tree counts were not used in the model. The comparison indicated that all models produce plausible results.

2.2 *Simulation experiments*

In a full LANDIS-II simulation, the model runs a spin-up period to calculate starting biomass for the given age structure, but in the single cell site tool all cohorts grow from age zero. Simulations are therefore similar to site conditions in 1870 when the site experienced large scale disturbance, clearing most of the cohorts (Brůna et al. 2013).

Simulations – current conditions

Current maximum biomass and ANPP was used, as well as current parameters for allometric equations to simulate growth and mortality under current climate conditions. We have done two simulations for current conditions:

1) Age-related mortality – using original Biomass Succession Extension settings

2) Height-related mortality – which replaces age-related mortality

Simulations – future conditions

In general, the LANDIS-II Biomass Succession extension can be calibrated by the PnET-II model to get direct climate sensitivity (Xu et al. 2009). There is even a new PnET-Succession extension for LANDIS-II that fully integrates PnET-II (de Bruijn et al. 2014), which can be used instead of the Biomass Succession extenstion, which may be more useful for climate predictions. However, we lacked the data to parameterize the PnET-II model and instead used a proxy for climate change within the Biomass Succession extension. That is, we increased productivity (ANPP) and growth rates according to results by Pretzsch et al. (2014), with the assumption that we can expect at least changes in growth condition as documented for the period 1900-2000 AD.

3) Age related mortality – using original Biomass Succession extension settings with increased ANPP and faster growth in allometric equations

4) Height related mortality – which replaces Age related mortality with increased ANPP and faster growth in allometric equations

3. SIMULATION RESULTS

Simulations without regeneration

Differences in mortality models are best visible in models without regeneration. For the current climate, the two mortality formulations were tuned so that they feature the same development of biomass over time (Fig. 2).

Future climate results show that faster growth due to elevated ANPP and increased growth along allometric curves will result in shorter lifespan of cohorts when using height-related mortality.

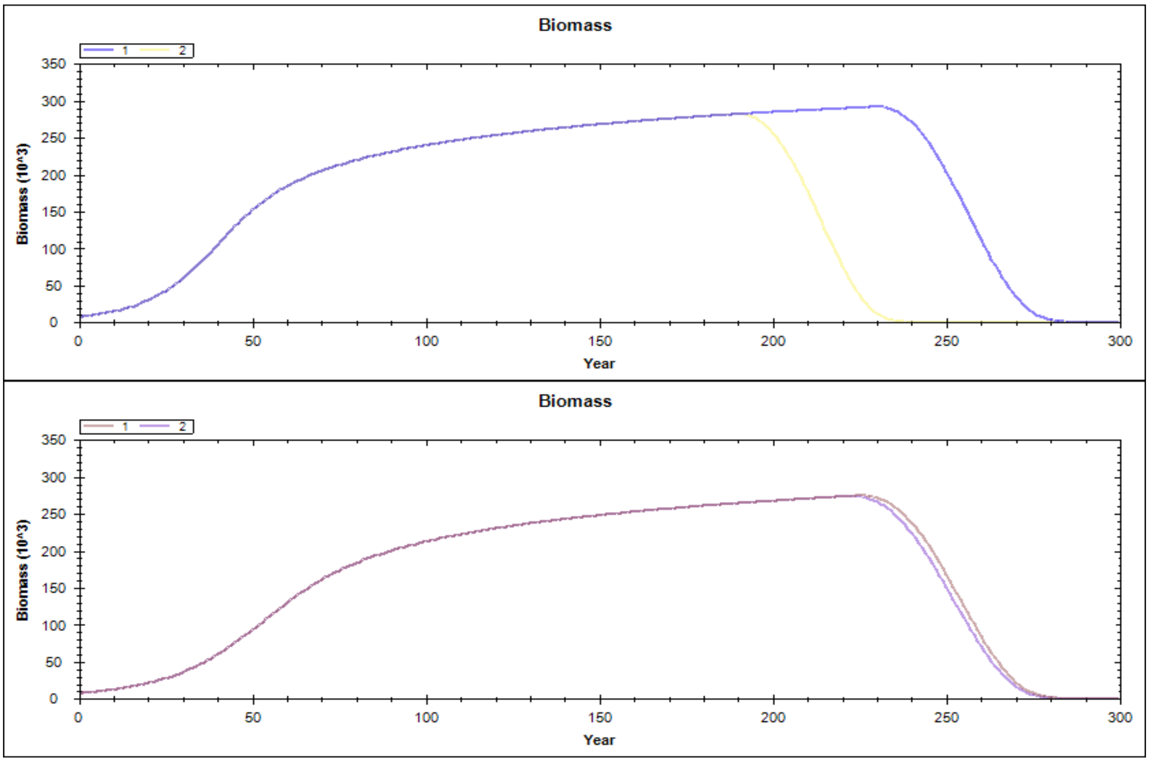

Fig. 2: Biomass curve of a stand using two version of mortality under current climate (top graph) and future climate (bottom graph). Line (1) Age-related mortality, (2) Height-related mortality.

Simulations with regeneration

Regeneration is done stochastically based on establishment probability, which means each run is different. We would normally use multiple runs to derive the distribution of results, but in order to keep outputs simple, a constant random number generator was used to mitigate this behavior.

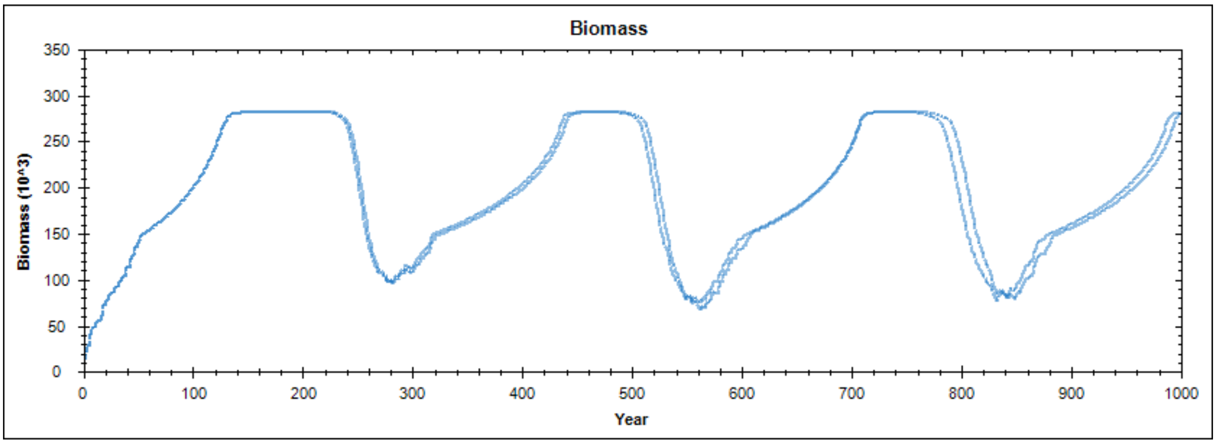

Fig. 3 Biomass curve of a stand using two version of mortality under current climate. Age related mortality and Height related mortality simulations show similar biomass pattern across several stand cycles.

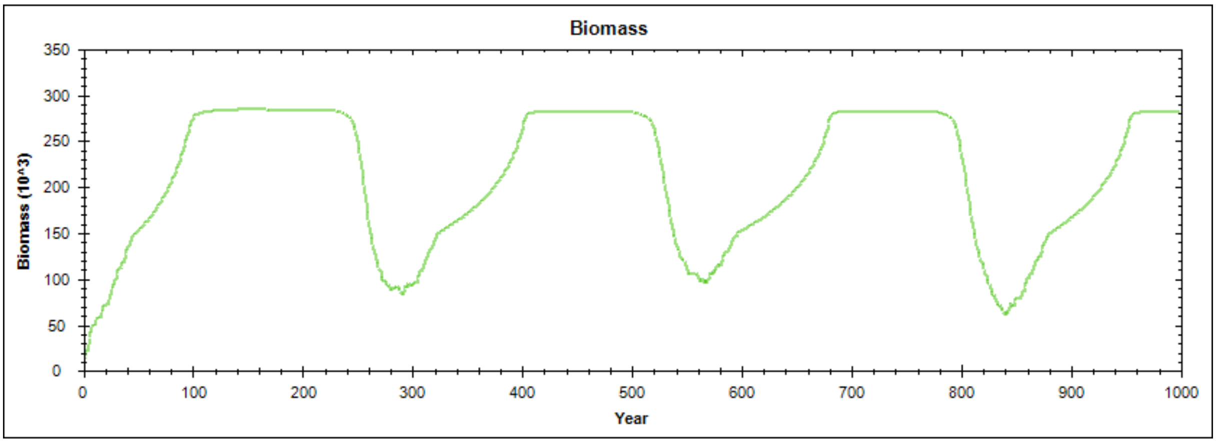

Fig. 4 Biomass curve of a stand using Age related mortality under future climate

The age-related mortality in the original model is not affected by higher ANPP and faster growth, since the process of aging is not changed (Fig. 3-4.). But the biomass will reach its maximum sooner and this state will last longer.

Extended growing season, expected with climate change (Pretsch et al, 2014) will significantly increase annual growth rate and trees will therefore reach its limits faster. We have chosen height, because it is perceived as a growth limit, especially in the mountains, where tree height is inversely dependent on elevation (Svoboda, 2005).

Height related mortality shows increased mortality under conditions of faster growth (Figures 5-6). This will result in shorter lifespan of individual cohorts and lower mean stand age. Period of maximum stand biomass will also be shorter, because the stand will reach critical heights sooner. Stands will also be more prone to wind disturbances, since tree exposure to wind increases with its height. But this is out of scope of these simulations.

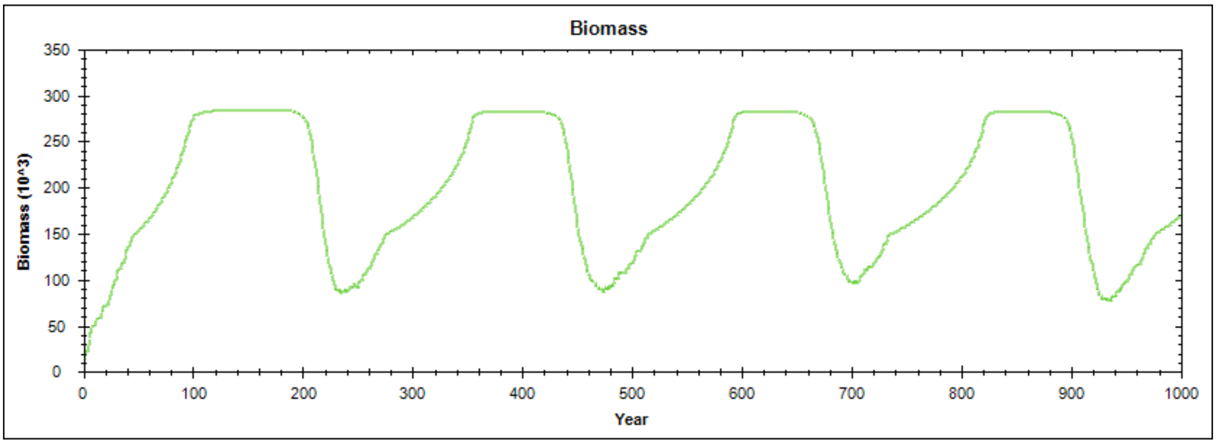

Fig. 5 Biomass curve of a stand using Height related mortality under future climate

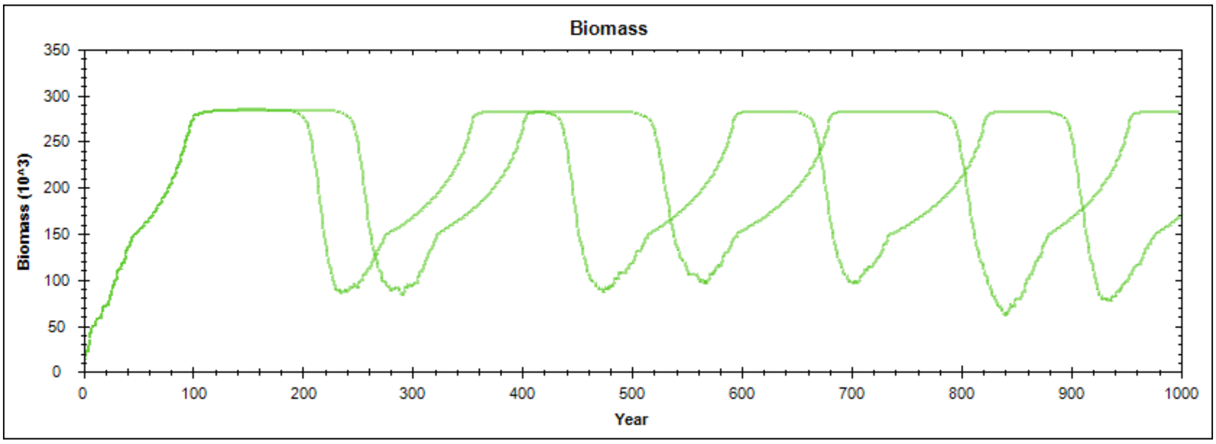

Fig. 6 Comparison of Age and Height related mortality using biomass of a stand under future climate

4. REFERENCES

Arjan de Bruijn, Eric J. Gustafson, Brian R. Sturtevant, Jane R. Foster, Brian R. Miranda, Nathanael I. Lichti, Douglass F. Jacobs. 2014. Toward more robust projections of forest landscape dynamics under novel environmental conditions: Embedding PnET within LANDIS-II. Ecological Modelling Volume 287, 10 September 2014, Pages 44–57

Brůna, J., Wild, J., Svoboda, M., Heurich, M., Müllerová, J., 2013. Impacts and underlying factors of landscape-scale, historical disturbance of mountain forest identified using archival documents. For. Ecol. Manage. 305, 294–306. doi:10.1016/j.foreco.2013.06.017

García, O. (2009). A simple and effective forest stand mortality model. *Mathematical and Computational Forestry & Natural-Resource Sciences (MCFNS)*, *1*(1), pp–1. http://doi.org/10.1063/1.3517105

Macek, Martin, Jan Wild, Martin Kopecký, Jaroslav Červenka, Miroslav Svoboda, Jitka Zenáhlíková, Josef Brůna, Reinhard Mosandl, and Anton Fischer. 2016. "Life and Death of Picea Abies after Bark-Beetle Outbreak: Ecological Processes Driving Seedling Recruitment." Ecological Applications 27 (1): 156–67. doi:10.1002/eap.1429.

Miranda, B. R. 2012. LANDIS-II-Site v2.3 User Guide. Available online at : http://www.landis-ii.org/tools/veg-site-tool

Muukkonen, P., 2007. Generalized allometric volume and biomass equations for some tree species in Europe. Eur. J. For. Res. 126, 157–166. doi:10.1007/s10342-007-0168-4

Pretzsch, H., Biber, P., Schütze, G., Uhl, E., Rötzer, T., 2014. Forest stand growth dynamics in Central Europe have accelerated since 1870. Nat. Commun. 5, 4967. doi:10.1038/ncomms5967

Scheller, R.M., Domingo, J.B., Sturtevant, B.R., Williams, J.S., Rudy, A., Gustafson, E.J., Mladenoff, D.J., 2007. Design, development, and application of LANDIS-II, a spatial landscape simulation model with flexible temporal and spatial resolution. Ecol. Modell. 201, 409–419.

Scheller, R.M., Mladenoff, D.J., 2004. A forest growth and biomass module for a landscape simulation model, LANDIS: design, validation, and application. Ecol. Modell. 180, 211–229. doi:10.1016/j.ecolmodel.2004.01.022

Svoboda, M., 2005. Struktura horského smrkového lesa v oblasti Trojmezné ve vztahu k historickému vývoji a stanovištním podmínkám. Silva Gabreta 11, 43–62.

Xu, C., G. Z. Gertner, and R. M. Scheller. 2009. Uncertainty in the response of a forest landscape to global climatic change. Global Change Biology15, 116–13.

11. SIMULATION RESULTS FROM LPJ-GUESS

Jörg Steinkamp and Thomas Hickler

Senckenberg Biodiversity and Climate Research Centre, Frankfurt, Germany

Abstract: We show results of a comparison between two different background mortality algorithms, longevity and diameter based, with the dynamic global vegetation model LPJ-GUESS. Five documented sites in Europe were chosen, two boreal and three temperate, to compare the simulation results to. The model was run with five climate drivers (ISI-MIP Fasttrack) and two scenarios (RCP 2.6/8.5). For each scenario LPJ-GUESS results from the different climate drivers were averaged. For temperate regions our results confirm (Bugmann & Bigler, 2011), that with a diameter based mortality trees die younger under elevated CO_2 and less carbon is accumulated in the vegetation biomass. In the boreal zone this is only true for a high emission scenario. Whereas, under the low emission scenario in boreal zone trees seem to live longer when the background mortality is based on diameter instead of longevity.

1. METHODS

1.1 FOREST MODEL

LPJ-GUESS is a dynamic global vegetation model with gap-dynamics, which can be applied from the global to the local scale. Depending on the knowledge of species in the simulated regions either global PFTs or regional to local species can be parameterized (Smith *et al.*, 2001, 2014; Sitch *et al.*, 2003; Hickler *et al.*, 2012). At the European sites species parameterization from (Hickler *et al.*, 2012) were adopted to the new model version including nitrogen cycling (Smith *et al.*, 2014).

1.2 MORTALITY FORMULATIONS

In LPJ-GUESS mortality consists of three components: 1.) Fire, with a parameterized probability to die per PFT if fire occurs; 2.) Background mortality based on longevity parameterized per PFT and 3.) Mortality through bad growing conditions measured as a 5-year running mean of growth efficiency, where growth efficiency is NPP divided by absolute leaf area (Smith *et al.*, 2001; Manusch *et al.*, 2012).

As alternative mortality scheme, the background mortality was modified, to use the tree diameter instead of the longevity as measure. The formula (Eq. 1) remains the same, but the constants are adjusted to yield a comparable average cumulative mortality probability for the three main species at the sites under current climate conditions (Fig. 1). For the three major shade-tolerant species (*Picea abies*, *Fagus sylvatica, Pinus sylvestris*) the maximum diameter was determined as 105 and 115 cm, respectively, and for *Pinus sylvestris* (intermediate shade tolerant) 72cm. For the co-occurring shade-intolerant species *Betula pubescens* and *Populus tremula* the maximum diameters were 65 and 57cm, respectively.

$$P_{mortality} = C * \frac{Q+1}{x_{max}} * \left(\frac{x}{x_{max}}\right)^Q \qquad \text{Eq. 1}$$

with C = -ln(0.001), Q = 2, x_{max} = longevity, x = age for the longevity based background mortality and C = 1.75, Q=3, x_{max} = max. diameter and x = actual diameter for the diameter based background mortality.

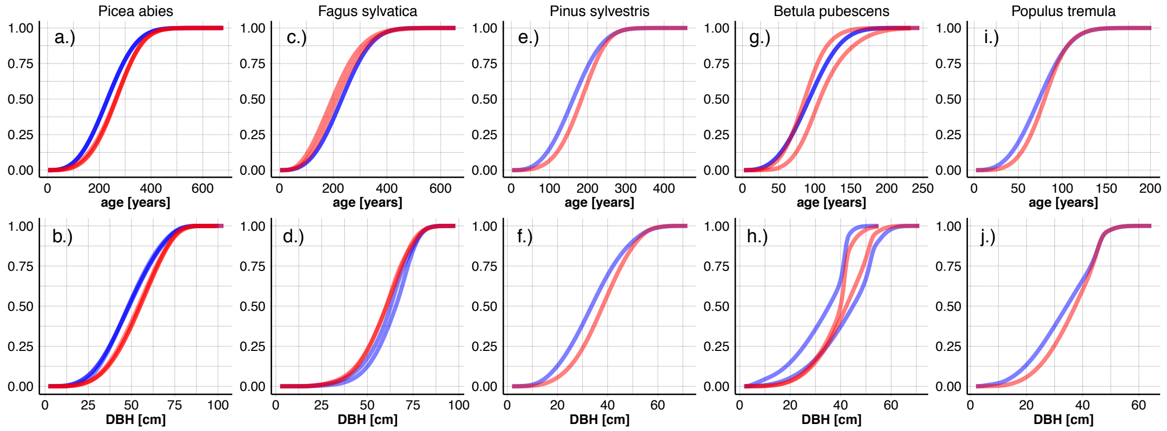

Fig. 1: Cumulative mortality probability based on longevity (blue) and diameter (red) for spruce (a, b), beech (c, d), pine (e, f), birch (g, h) and poplar (i, j) in relation to age (a, c, e, g, i) and diameter (b, d, f, h, j)

This should result in a faster carbon turnover rate with increased CO_2 fertilization under climate change scenarios as postulated by Bugmann & Bigler (2011).

2. Simulation Settings

2.1 Site data

We choose 5 sites, two boreal, three temperate. The simulated soil has a depth of 1.5 m with two layers (top 0.5 m, bottom 1 m) for each stand (Fig. 2). Texture classes are supplied with the model. Additionally atmospheric nitrogen deposition is required, which is based on (Lamarque *et al.*, 2011, 2013). Climate data (short wave radiation, temperature and precipitation) was taken from the 5 available ISI-MIP Fast-track models for the nearest grid cell with 0.5° resolution (Warszawski *et al.*, 2014). As future scenario RCP 2.6 and RCP 8.5 were applied and after 2100 the last 30 years were randomly repeated for additional 300 years. The CO_2 increased from 389.3 ppm in 2010 to 421.4 and 926.7 in 2099 for RCP 2.6 and RCP 8.5, respectively and was kept constant after 2099. Differences of the climate drivers are summarized in Tables 1-3. We implemented a very simple management to achieve a close to natural regeneration for the future simulations. After plantation, no establishment and disturbance was allowed within the first ten years. Establishment then increased linearly for the next 20 years to reach its normal values.

Table 1: Absolut changes in temperature (K) averaged over the five climate model drivers at the simulated sites between the reference period (1981-2010) and the last 30 years in the dataset (2070-2099) for the two scenarios in each season (DJF: December, January, February; MAM: March, April, May; JJA: June, July, August; SON: September, October, November)

	RCP 2.6				RCP 8.5			
	DJF	MAM	JJA	SON	DJF	MAM	JJA	SON
**Flakaliden**	2.72	2.04	2.29	1.96	7.53	5.73	5.72	5.33
**Hyytiala**	2.59	2.28	2.29	2.00	7.35	6.08	5.80	5.39
**Peitz**	1.80	1.49	1.92	1.75	4.65	3.88	5.33	4.71
**Solling**	1.59	1.29	1.63	1.56	4.27	3.53	4.94	4.45
**Soroe**	1.62	1.51	1.87	1.56	4.39	4.00	4.79	4.37

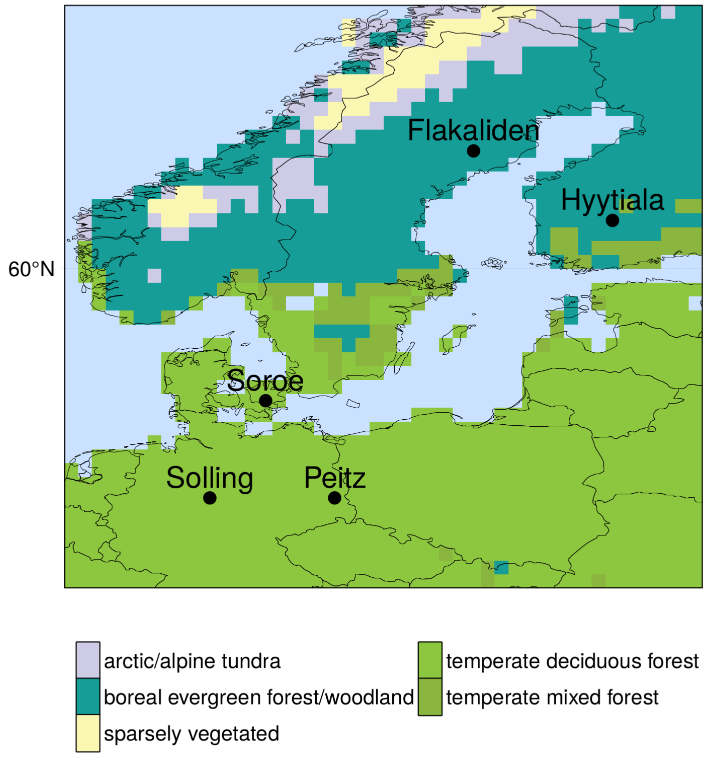

Fig. 2: Simulated sites with potential natural vegetation under current climate conditions

Table 2: Relative changes in precipitation averaged over the five climate model drivers at the simulated sites between the reference period (1981-2010) and the last 30 years in the dataset (2070-2099) for the two scenarios

	**RCP 2.6**				**RCP 8.5**			
	DJF	MAM	JJA	SON	DJF	MAM	JJA	SON
**Flakaliden**	1.11	1.05	1.04	1.07	1.37	1.17	1.09	1.19
**Hyytiala**	1.07	1.01	1.04	1.08	1.29	1.11	1.06	1.19
**Peitz**	1.08	1.03	1.07	1.02	1.17	1.06	0.95	1.05
**Solling**	1.09	1.03	1.10	1.07	1.19	1.06	0.97	1.07
**Soroe**	1.08	0.99	1.05	1.09	1.25	1.08	1.01	1.16

Table 3: Absolute changes in radiation (W m^{-2}) averaged over the five climate model drivers at the simulated sites between the reference period (1981-2010) and the last 30 years in the dataset (2070-2099) for the two scenarios

	**RCP 2.6**				**RCP 8.5**			
	DJF	MAM	JJA	SON	DJF	MAM	JJA	SON
**Flakaliden**	-0.45	0.37	9.15	1.78	-1.52	-4.06	11.38	1.99
**Hyytiala**	-0.29	3.29	9.38	2.96	-1.34	1.27	10.09	3.45
**Peitz**	1.05	8.78	10.13	6.12	0.00	7.07	13.39	7.18
**Solling**	0.81	8.38	9.73	5.42	0.33	6.53	14.32	7.03
**Soroe**	0.98	9.20	10.78	3.85	0.62	6.40	11.00	4.69

Test data

Test data were partly extracted from the literature (Table 4); detailed stand data for comparison were available for two ISI-MIP stands (Peitz and Solling). Further stand data from EUROFLUX sites were used for Hyytiälä and Sorø.

Table 4: Literature obtained values for the simulated stands. [a]co-occuring species in brackets; PiAb: *Picea abies*; PiSy: *Pinus sylvestris*; BePu: *Betula pendula*; PoTr: *Populus tremula*; FaSy: *Fagus sylvatica*

Stand	Year	Species composition[a]	Biomass kg^{-2}	LAI $m^2\ m^{-2}$	BA $m^2\ ha^{-1}$	Density ha^{-1}	Reference
**Flakaliden**	1986-96	PiAb			2.1 – 11.0	2400 – 2740	(Bergh *et al.*, 1999)
	2001	(PiSy, BePu)	4.63		20		(Kleja *et al.*, 2007)
	2002			3.4			(Lindroth *et al.*, 2008)
**Hyytiälä**	1995	PiAb	4.7	4.5		2500	(Kulmala *et al.*, 2001)
	1996	(PoTr, BePu)	7.2			2100 – 2500	(Markkanen *et al.*, 2001)
	1999			3.3			(Lindroth *et al.*, 2008)
	2000			3.5			(Rannik *et al.*, 2002)
**Peitz**		PiSy					
**Solling**		FaSy					
**Sorø**	1995	FaSy (PiAb)		5	32.1	283	(Pilegaard *et al.*, 2011)

2.2 Simulation experiments

The model was run on 50 replicated patches per site, using the plantation years shown in Tab. 5, and it was spun up for 750 years.

Table 5: Plantation year

Stand	Plantation year	Reference
**Flakaliden**	1963 (4-year old saplings)	(Hedwall *et al.*, 2015)
**Hyytiälä**	1962	(Markkanen *et al.*, 2001)
**Peitz**	1900	
**Solling**	1847	
**Sorø**	1921	(Pilegaard *et al.*, 2011)

3. Simulation results

Due to the missing management (thinning) in the simulation, we obtained many more trees in the lower DBH classes (Fig. 3, green bars) than observed. However, without regeneration future projections would not be possible. This also leads to a stronger competition of the old

growth trees with younger generations, especially visible at the Solling site, where the simulated trees do not reach up to the same DBH values as observed.

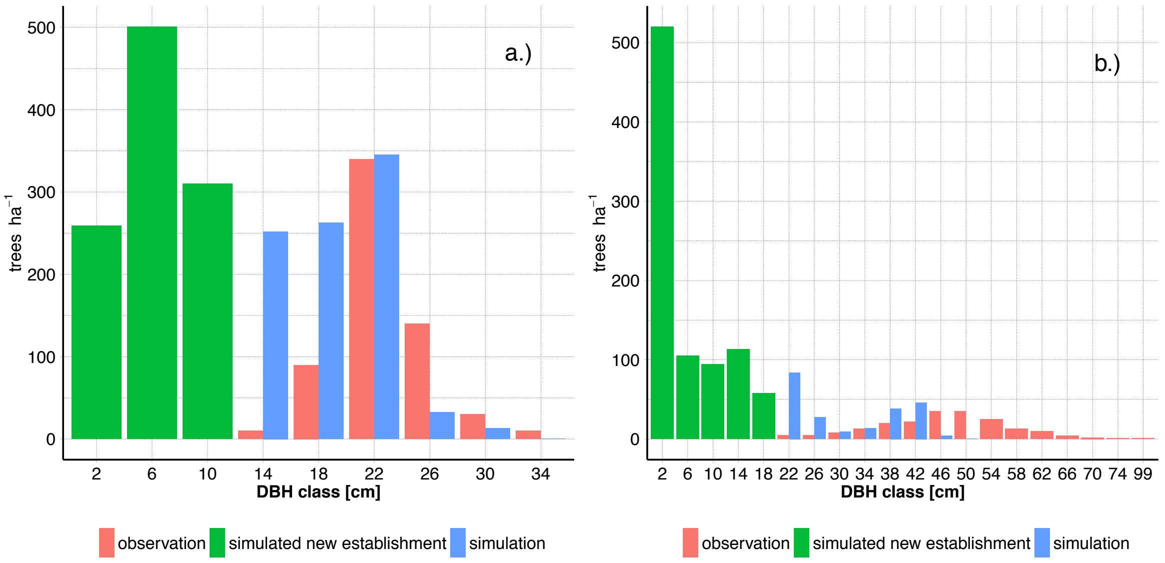

Fig. 3: Observed (blue) and simulated (green/blue) DBH distribution for a) Peitz in 2008 and b) Solling in 2004

Due to the slow growth at higher latitudes (Flakaliden and Hyytiälä), simulated forests accumulated more carbon with a diameter-based algorithm under the low emission scenario RCP 2.6 (Fig. 4, 6). However, this gain turned into a carbon loss by faster growth and turnover under the high emission scenario RCP 8.5 (Fig. 5, 7). For the temperate sites, there is – not surprisingly – hardly any difference under the low emission scenario, since the two algorithms were calibrated under current climate conditions to yield similar results and the RCP 2.5 does not deviate strongly from current conditions (Fig. 8, 10, 12). Under the high emission scenario, the simulated vegetation carbon decreases faster with the diameter-based algorithm compared to the longevity-based algorithm (Fig. 9, 11, 13).

Table 6: Count of killed trees per ha between 2000 and 2199 for the respective site and mortality algorithm

		RCP 2.5		RCP 8.5	
		longevity	diameter	longevity	diameter
**Flakaliden**	BePu	2388	2427	2696	3071
	PiAb	2998	3069	3186	3452
	PiSy	2204	2095	2481	2644
	Σ	7589	7591	8363	9168
**Hyytiälä**	BePu	1810	1878	1774	1773
	PiAb	3126	3035	3019	3001
	PoTr	1811	1867	1770	1849
	Σ	6746	6780	6564	6623
**Peitz**	PiSy	6227	5932	6305	6705
**Solling**	FaSy	7973	7157	7820	7857
**Sorø**	FaSy	4990	5109	5223	5247
	PiAb	5900	5739	5793	5903
	Σ	10890	10848	11016	11149

By counting all killed trees from one year old saplings to big mature trees between 2000 and 2199 for the two scenarios and mortality algorithms (Table 6), the sites Flakaliden, Peitz and Sorø show an increase in the number of dead trees with the high emission scenario compared to the low emission

scenario. In contrast, in Hyytiälä the number of killed trees is slightly lower under the high emission scenario, and in Solling the number of dead trees decreases with the longevity algorithm and increases with the diameter algorithm from RCP 2.6 to RCP 8.5.

4. REFERENCES

Bergh J, Linder S, Lundmark T, Elfving B (1999) The effect of water and nutrient availability on the productivity of Norway spruce in northern and southern Sweden. *Forest Ecology and Management*, **119**, 51–62.

Bugmann H, Bigler C (2011) Will the CO2 fertilization effect in forests be offset by reduced tree longevity? *Oecologia*, **165**, 533–544.

Grünwald T, Bernhofer C (2007) A decade of carbon, water and energy flux measurements of an old spruce forest at the Anchor Station Tharandt. *Tellus B*, **59**, 387–396.

Hedwall P-O, Skoglund J, Linder S (2015) Interactions with successional stage and nutrient status determines the life-form-specific effects of increased soil temperature on boreal forest floor vegetation. *Ecology and Evolution*, **5**, 948–960.

Hickler T, Vohland K, Feehan J et al. (2012) Projecting the future distribution of European potential natural vegetation zones with a generalized, tree species-based dynamic vegetation model. *Global Ecology and Biogeography*, **21**, 50–63.

Kleja DB, Svensson M, Majdi H et al. (2007) Pools and fluxes of carbon in three Norway spruce ecosystems along a climatic gradient in Sweden. *Biogeochemistry*, **89**, 7–25.

Kulmala M, Hämeri K, Aalto PP et al. (2001) Overview of the international project on biogenic aerosol formation in the boreal forest (BIOFOR). *Tellus B*, **53**, 324–343.

Lamarque J-F, Kyle GP, Meinshausen M et al. (2011) Global and regional evolution of short-lived radiatively-active gases and aerosols in the Representative Concentration Pathways. *Climatic Change*, **109**, 191–212.

Lamarque J-F, Dentener F, McConnell J et al. (2013) Multi-model mean nitrogen and sulfur deposition from the Atmospheric Chemistry and Climate Model Intercomparison Project (ACCMIP): evaluation of historical and projected future changes. *Atmos. Chem. Phys.*, **13**, 7997–8018.

Lindroth A, Lagergren F, Aurela M et al. (2008) Leaf area index is the principal scaling parameter for both gross photosynthesis and ecosystem respiration of Northern deciduous and coniferous forests. *Tellus B*, **60**, 129–142.

Manusch C, Bugmann H, Heiri C, Wolf A (2012) Tree mortality in dynamic vegetation models – A key feature for accurately simulating forest properties. *Ecological Modelling*, **243**, 101–111.

Markkanen T, Rannik U, Keronen P, Suni T, Vesala T (2001) Eddy covariance fluxes over a boreal Scots pine forest. *Boreal environment research*, **6**, 65–78.

Pilegaard K, Ibrom A, Courtney MS, Hummelshøj P, Jensen NO (2011) Increasing net CO2 uptake by a Danish beech forest during the period from 1996 to 2009. *Agricultural and Forest Meteorology*, **151**, 934–946.

Rannik Ü, Altimir N, Raittila J et al. (2002) Fluxes of carbon dioxide and water vapour over Scots pine forest and clearing. *Agricultural and Forest Meteorology*, **111**, 187–202.

Rey A, Pegoraro E, Tedeschi V, De Parri I, Jarvis PG, Valentini R (2002) Annual variation in soil respiration and its components in a coppice oak forest in Central Italy. *Global Change Biology*, **8**, 851–866.

Sitch S, Smith B, Prentice IC et al. (2003) Evaluation of ecosystem dynamics, plant geography and terrestrial carbon cycling in the LPJ dynamic global vegetation model. *Global Change Biology*, **9**, 161–185.

Smith B, Prentice IC, Sykes MT (2001) Representation of vegetation dynamics in the modelling of terrestrial ecosystems: comparing two contrasting approaches within European climate space. *Global Ecology and Biogeography*, **10**, 621–637.

Smith B, Wårlind D, Arneth A, Hickler T, Leadley P, Siltberg J, Zaehle S (2014) Implications of incorporating N cycling and N limitations on primary production in an individual-based dynamic vegetation model. *Biogeosciences*, **11**, 2027–2054.

Warszawski L, Frieler K, Huber V, Piontek F, Serdeczny O, Schewe J (2014) The Inter-Sectoral Impact Model Intercomparison Project (ISI–MIP): Project framework. *Proceedings of the National Academy of Sciences*, **111**, 3228–3232.

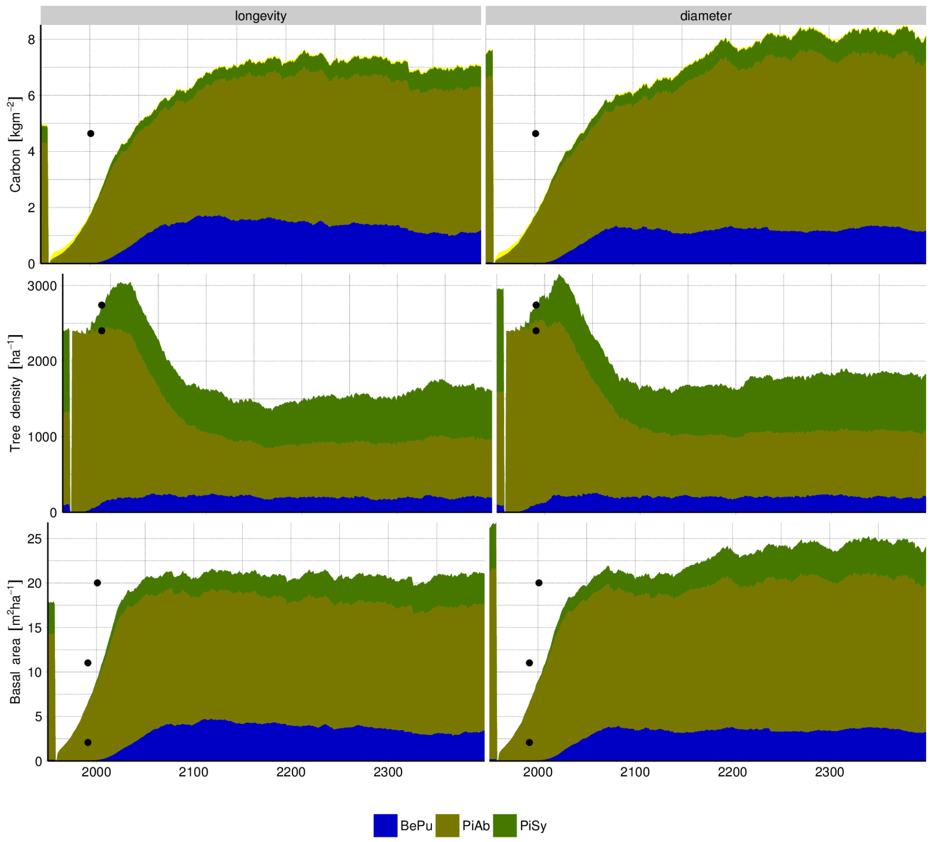

Fig. 4: Past and projected vegetation Carbon content, tree density and basal area for the site Flakaliden under scenario RCP 2.6 for the two described mortality algorithms (longevity and diameter).

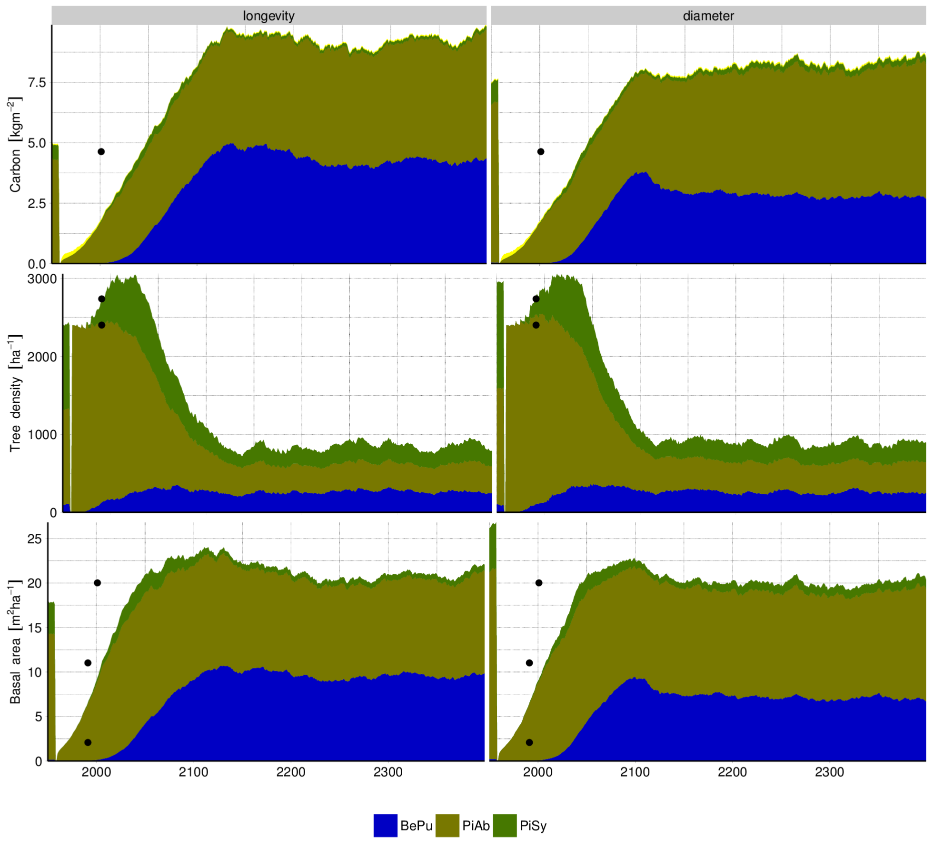

Fig. 5: Past and projected vegetation Carbon content, tree density and basal area for the site Flakaliden under scenario RCP 8.5 for the two described mortality algorithms (longevity and diameter).

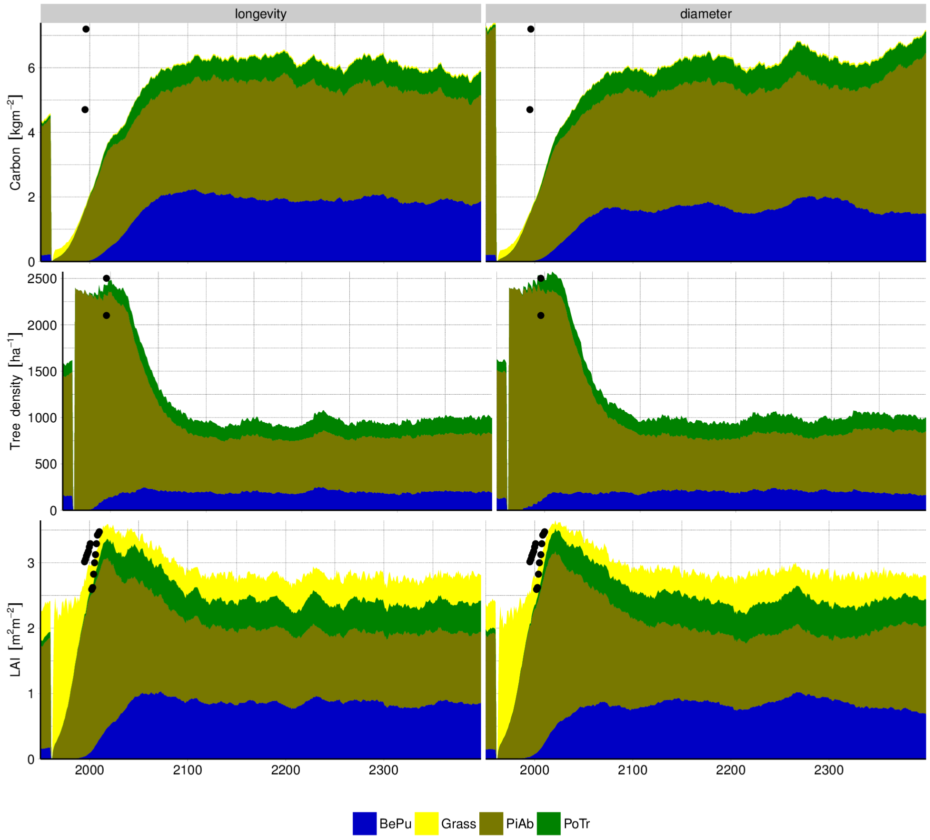

Fig. 6: Past and projected vegetation Carbon content, tree density and leaf area index (LAI) for the site Hyytiälä under scenario RCP 2.6 for the two described mortality algorithms (longevity and diameter). Dots are observed values from literature and the EUROFLUX data for LAI).

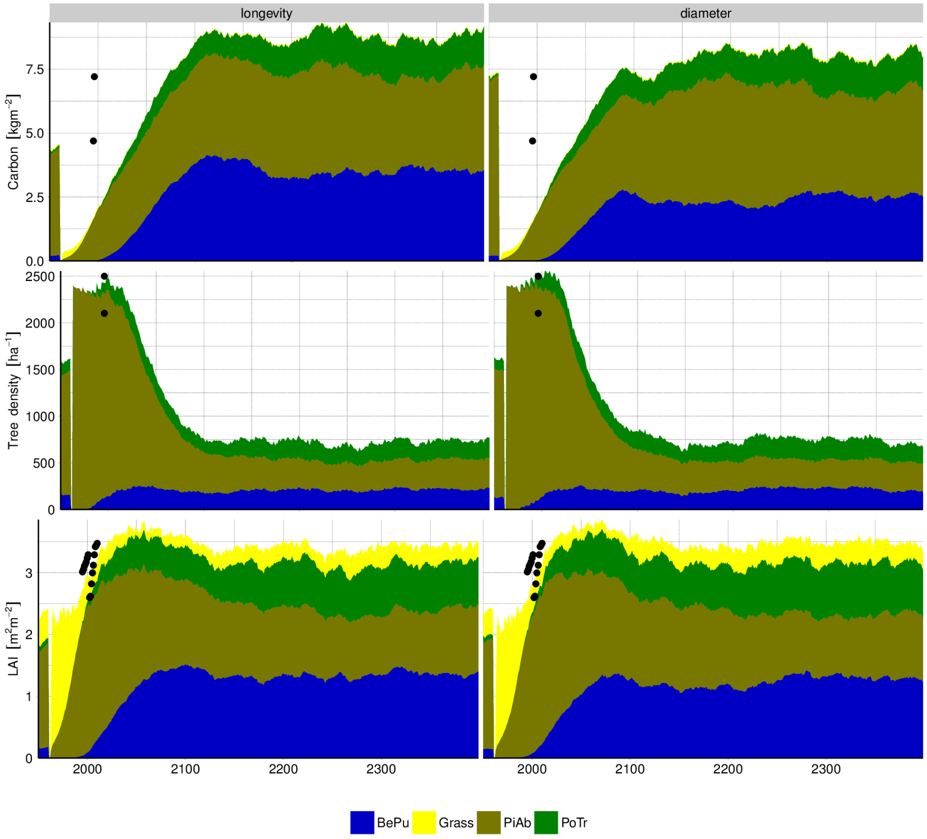

Fig. 7: Past and projected vegetation Carbon content, tree density and leaf area index for the site Hyytiälä under scenario RCP 8.5 for the two described mortality algorithms (longevity and diameter).

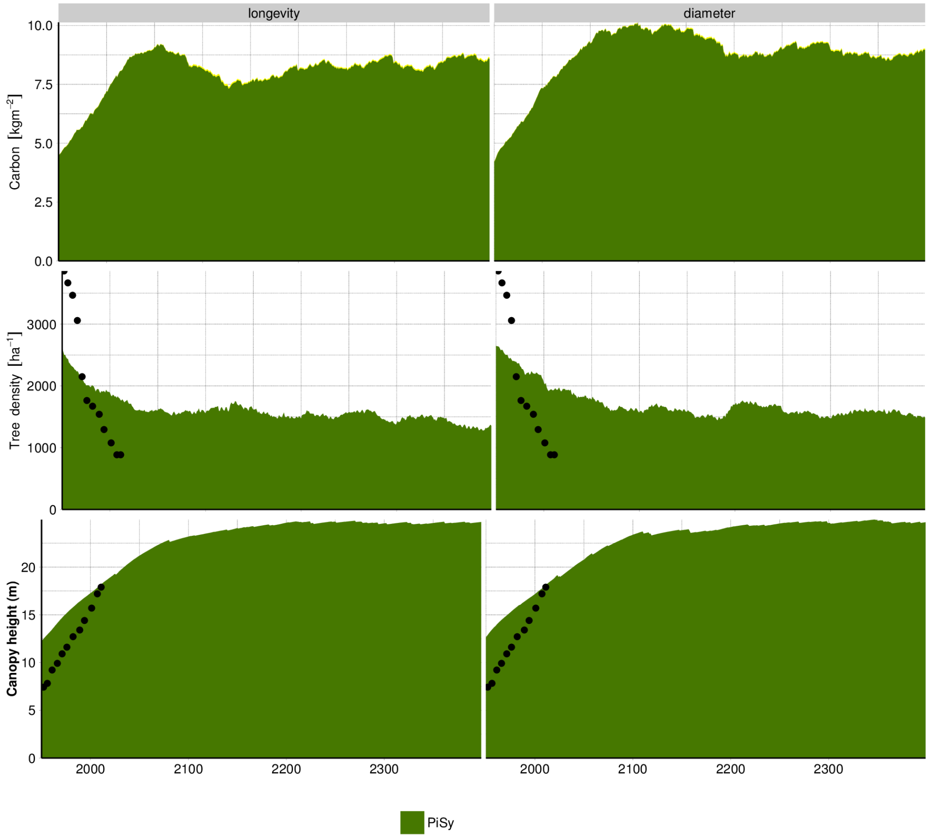

Fig. 8: Past and projected vegetation Carbon content, tree density and canopy height for the site Peitz under scenario RCP 2.6 for the two described mortality algorithms (longevity and diameter). Dots are observed values (from the ISI-MIP project)

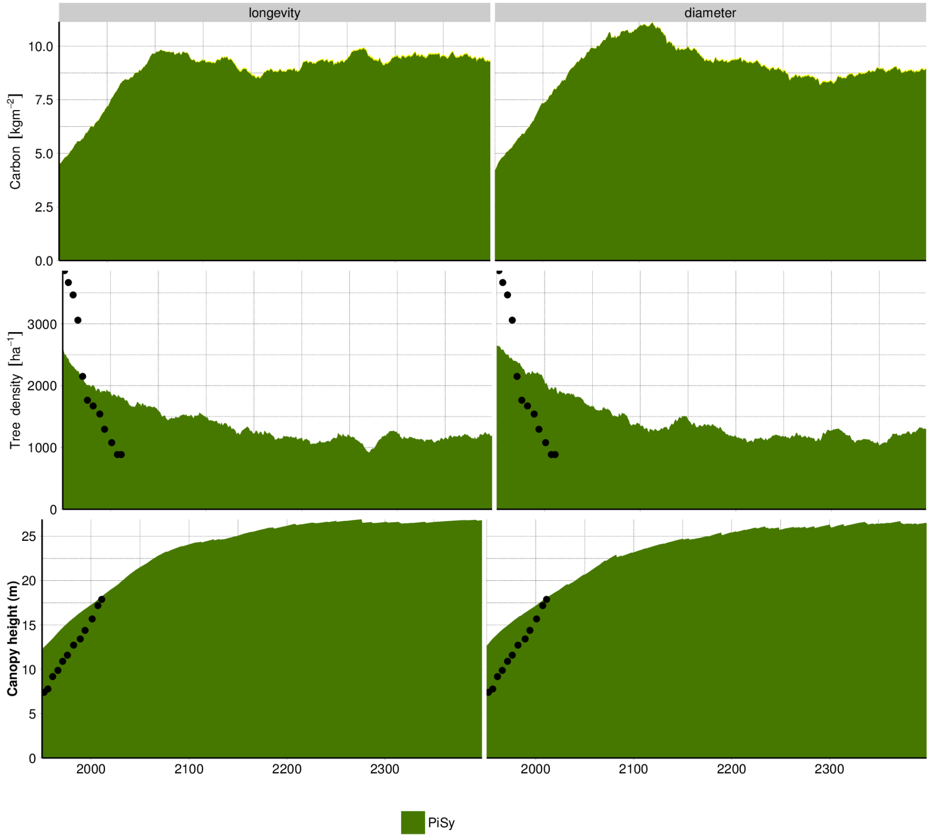

Fig. 9: Past and projected vegetation Carbon content, tree density and canopy height for the site Peitz under scenario RCP 8.5 for the two described mortality algorithms (longevity and diameter). Dots are observed values (from the ISI-MIP project)

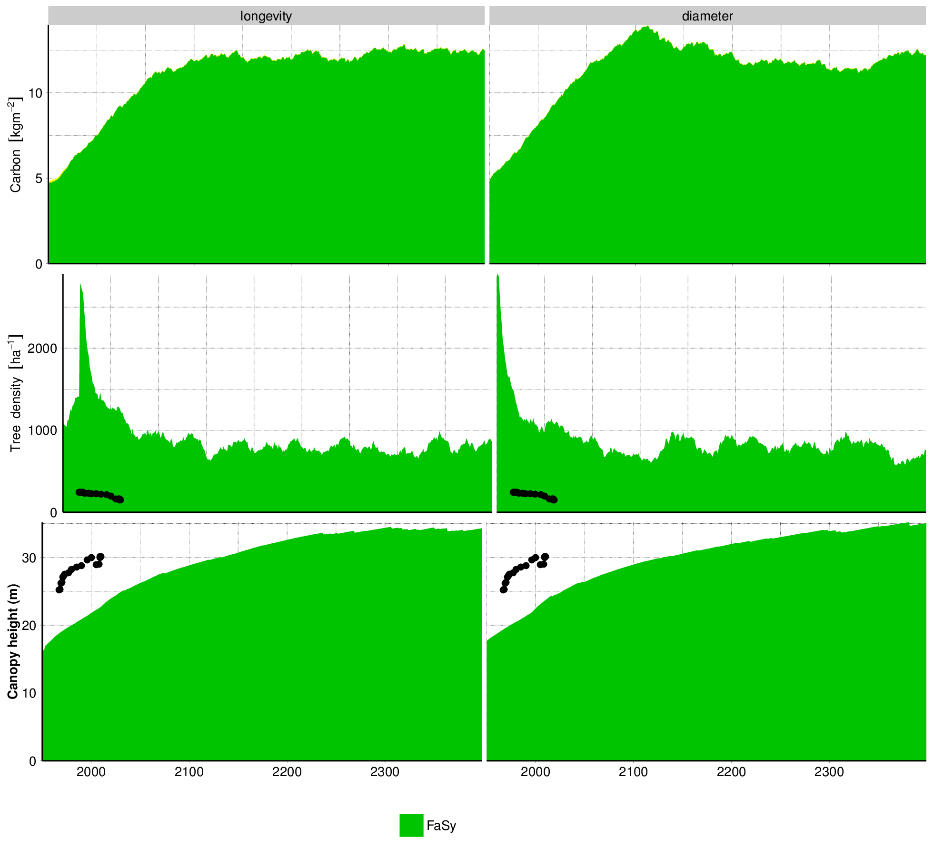

Fig. 10: Past and projected vegetation Carbon content, tree density and canopy height for the site Solling under scenario RCP 2.6 for the two described mortality algorithms (longevity and diameter). Dots are observed values (Ref. XXXXX)

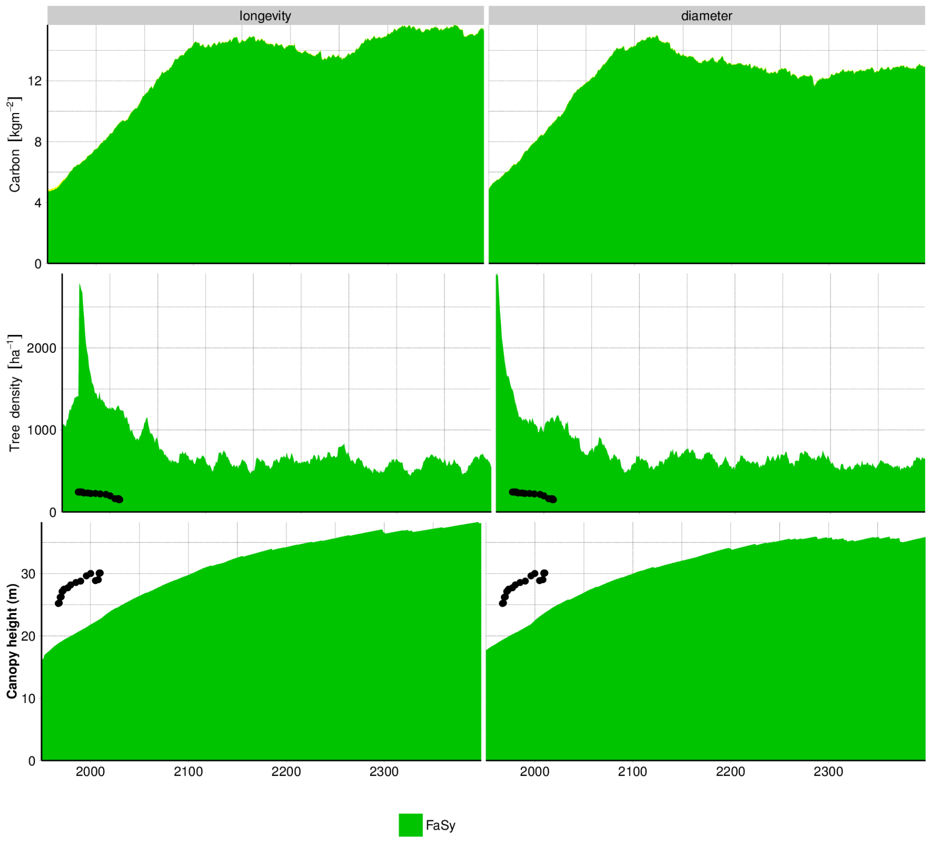

Fig. 11: Past and projected vegetation Carbon content, tree density and canopy height for the site Solling under scenario RCP 8.5 for the two described mortality algorithms (longevity and diameter).

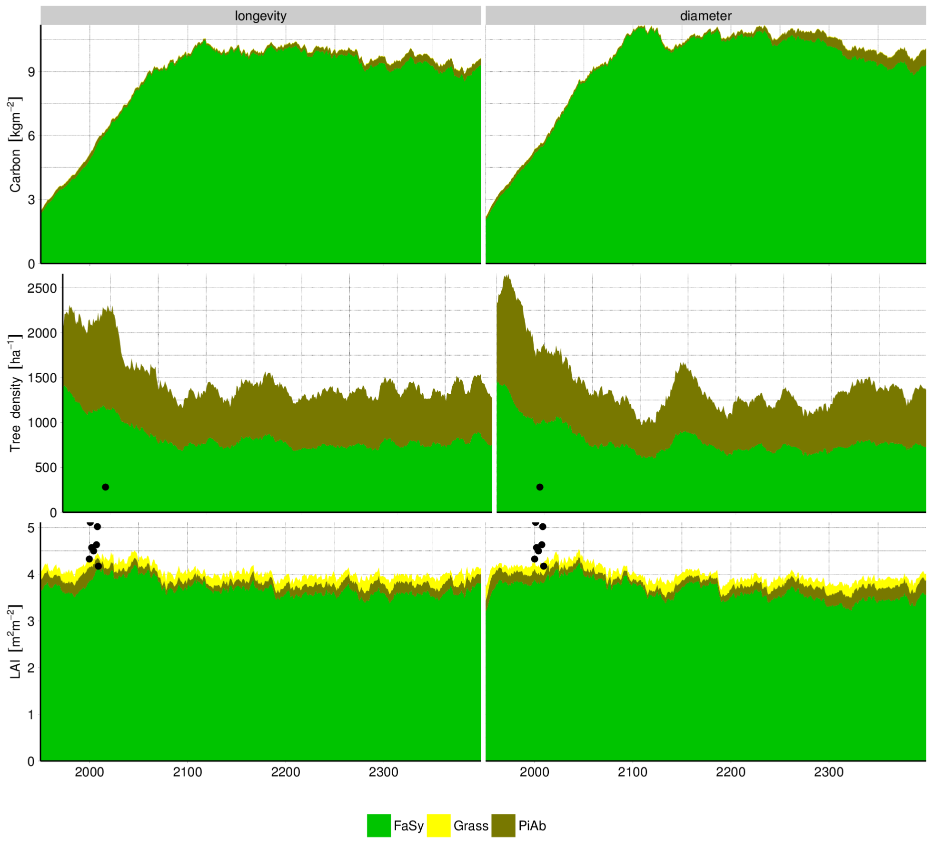

Fig. 12: Past and projected vegetation Carbon content, tree density and leaf area index (LAI) for the site Sorø under scenario RCP 2.6 for the two described mortality algorithms (longevity and diameter). Dots are observed values from the EUROFLUX site data.

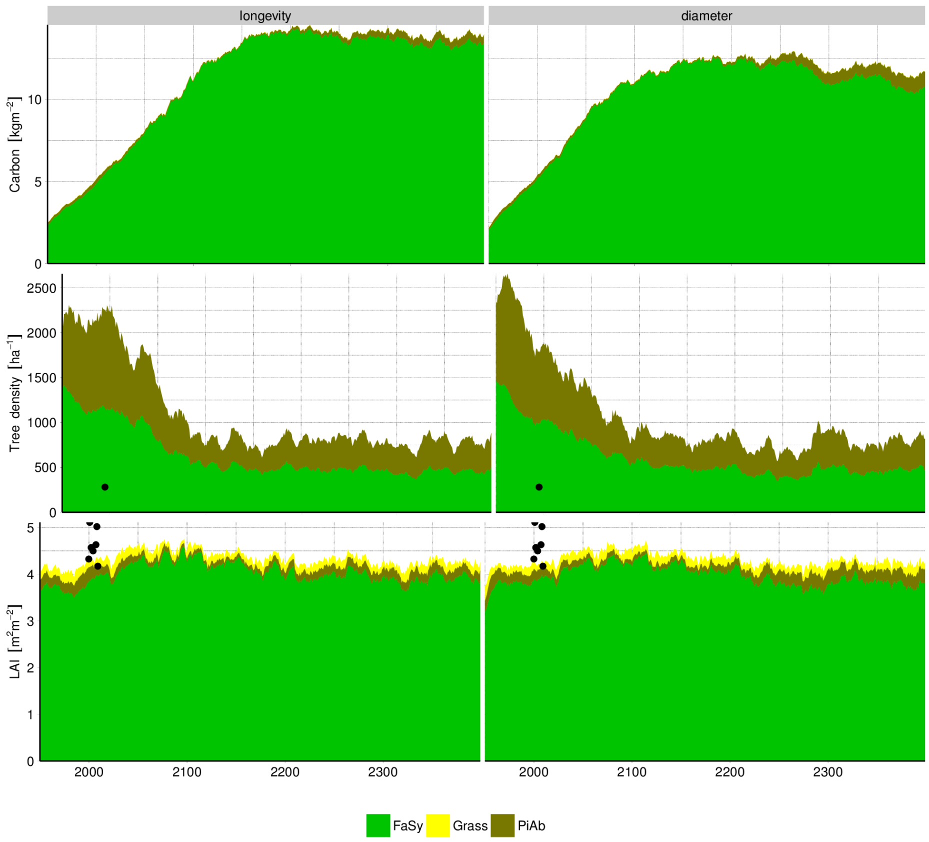

Fig. 13: Past and projected vegetation Carbon content, tree density and leaf area index (LAI) for the site Sorø under scenario RCP 8.5 for the two described mortality algorithms (longevity and diameter)

12. Simulation results from LPJmL

Anja Rammig[1,2], Jens Heinke[2]

[1]Technische Universität München TUM, School of Life Sciences Weihenstephan, Freising, Germany

[2]Potsdam Institute for Climate Impact Research (PIK), Potsdam, Germany

1. Methods

1.1 Forest model

We applied the Lund-Potsdam-Jena Dynamic Global Vegetation Model for managed Land (LPJmL; Bondeau et al. 2007). This model represents process-based large-scale terrestrial ecosystem dynamics and related carbon and water cycles (Sitch et al. 2003). The model relates plant physiological processes such as photosynthesis, maintenance and growth respiration, and canopy conductance to ecosystem processes such as tissue turnover and soil organic matter and litter dynamics. Vegetation in the Amazon is represented by three plant functional types (PFT), namely tropical evergreen forest, tropical raingreen forest and C4 grasses.

1.2 Mortality formulations

We applied two mortality formulations, (1) the standard mortality that is based on Sitch et al. (2003) and (2) a modified mortality formulation based on Pretzsch et al. (2002).

In the standard mortality formulation, the overall mortality rate for a population is the sum of the rates for individual mortality

$$mort = \max\,(mort_{shade} + mort_{greff} + mort_{NPP} + mort_{heat} + mort_{lim}, 1) \quad \text{(Eq. 1)}$$

Corresponding to mortality from light, low growth efficiency, a negative annual carbon balance, heat stress or mortality from exceeding bioclimatic limits, respectively (cf. Eq. 29 in Sitch et al. 2003). Our focus here is particularly on the mortality from low growth efficiency, $mort_{greff}$, which is calculated as

$$mort_{greff} = \frac{k_{mort\_max}}{1 + k_{mort2} \cdot greff} \quad \text{(Eq. 2)}$$

Where k_{mort_max} represents the maximum mortality rate and k_{mort2} is a parameter determining the slope of the relationship between mortality and growth efficiency (Sitch et al. 2003, Eq. 32). For our runs, which were tuned to match global biomass and vegetation distribution, k_{mort_max} is set to 0.03 and k_{mort2} is set to 0.1. Growth efficiency is calculated as

$$greff = \frac{\Delta C}{C_{leaf} SLA} \quad \text{(Eq. 3)}$$

and can thus be described as the ration of this year's net biomass increment (ΔC) to leaf area (Sitch et al. 2003, Eq. 31).

In the runs with the modified mortality version, we replace $mort_{greff}$ by a mortality that is described by the relation between basal area increment (BAI) and stem diameter (DBH). Following Pretzsch et al. 2002, we calculate the modified mortality as

$$mort_{mod} = \frac{1}{1+\exp\left(a_0 + a_1 . {BAI}/_{DBH}\right)} \quad \text{(Eq. 4)}$$

Values for parameters a_0 and a_1 are given in Pretzsch et al. (2002) for European tree species. For our purpose we had to tune the parameters so that they approximately reached the predicted rates of $mort_{greff}$ and to match the slopes of our reference data from Brienen et al. (2015). We chose a_0 =3.7 and a_1=100 (units in Pretzsch et al. were cm^2/cm^2 for this parameter, for our purpose it was multiplied by 100 to convert to m^2/m^2). These are preliminary parameter settings, since the output variables seem to be quite sensitive to changes in these parameters, in particular a_0.

2. SIMULATION SETTINGS

2.1 *SITE DATA*

For comparison of simulation results, we use site data from Brienen et al. (2015), who sampled aboveground biomass at several plots across and slightly beyond the Amazon basin. We focus here on the results that Brienen et al. (2015) shown in Figure 1, which are derived from 117 plots. For comparison with the simulation results, we selected the grid cells of 0.5° *x* 0.5° lon/lat resolution that contain the sample plots. Since some grid cells contained more than one plot, we obtained in total the values from 69 grid cells.

For an additional visual comparison of aboveground biomass distribution across the Amazon basin (Fig. 2), we use the biomass maps from Mitchard et al. (2014).

The model runs for current conditions (1901-2010) are driven by temperature data from CRU-TS-3.2 and precipitation from GPCC_v6.

The model runs for future scenarios are driven by the climatology from MPI-ESM-LR from the CMIP5 simulation runs for the period 1900-2299 for RCP4.5 and RCP 8.5. In these extended runs, it is assumed that all emissions are constant from 2100 (Meinshausen et al. 2011).

LPJmL uses atmospheric CO_2 concentration as input. For the runs for the current period, we use observed atmospheric CO_2 levels from Mauna Loa Station, for the future runs, we use the CO_2 emission pathways following RCP4.5 and RCP 8.5.

LPJmL uses soil type as input which comes from the HWSD Harmonized World Soil Database (FAO/IIASA/ISRIC/ISSCAS/JRC 2012).

2.2 *TEST DATA*

We compare model behavior to observed data from the Amazon basin from Brienen et al. (2015).

2.2 *SIMULATION EXPERIMENTS*

- Current: 5000 years spin-up 1901-1930 cycle, after that transient simulations from 1901-2010.
- Future: 5000 years spin-up 1901-1930 cycle, after that transient simulations from 1901-2299.

3. PRELIMINARY SIMULATION RESULTS

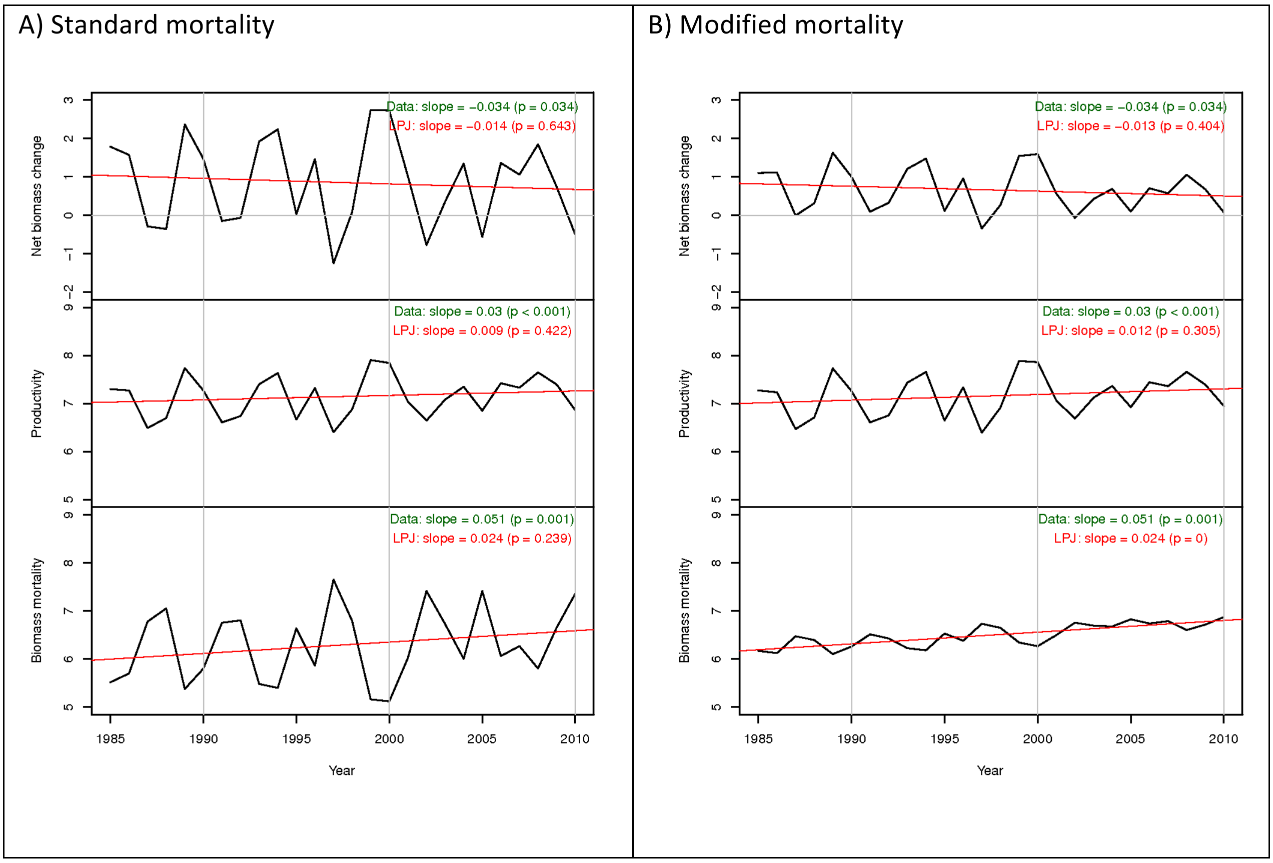

Fig. 1: Reproduced Figure 1 from Brienen et al. 2015 for the measurement sites as simulated with LPJmL. The two columns show the simulation results with A) the standard mortality formulation and B) with the modified mortality formulation. The three panels show from top to bottom: net biomass change, productivity and biomass mortality from 1985 to 2010. The slope of the timeseries is calculated by a linear fit to compare the simulated trend (LPJmL, red letters) with slopes from observational data (from Brienen et al. 2015, green letters)

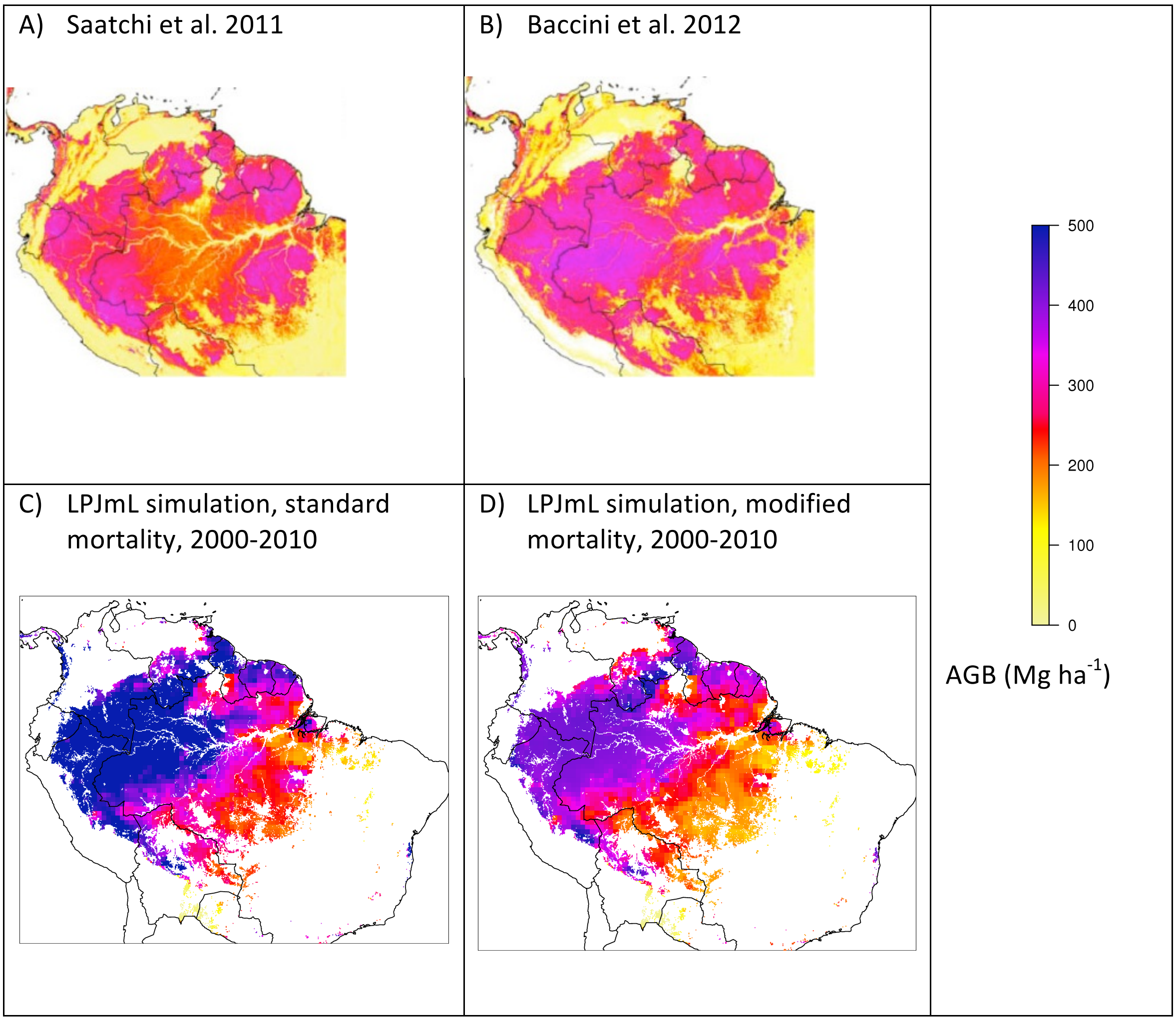

Fig. 2: Maps of aboveground biomass (AGB) for the Amazon forest under current conditions (2000-2010). A) observed, B) observed, C) simulated (standard mortality), D) simulated (modified mortality). The maps from Saatchi et al. 2011 and Baccini et al. 2012 are taken from Mitchard et al. 2014

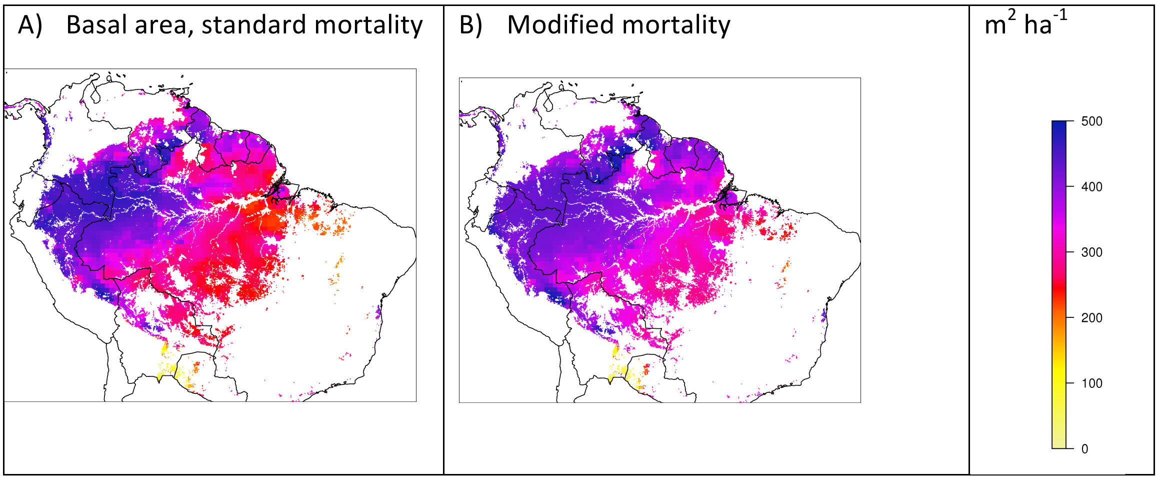

Fig. 3: Maps of basal area for the Amazon forest under current conditions (2000-2010) for (A) standard mortality and (B) modified mortality simulations

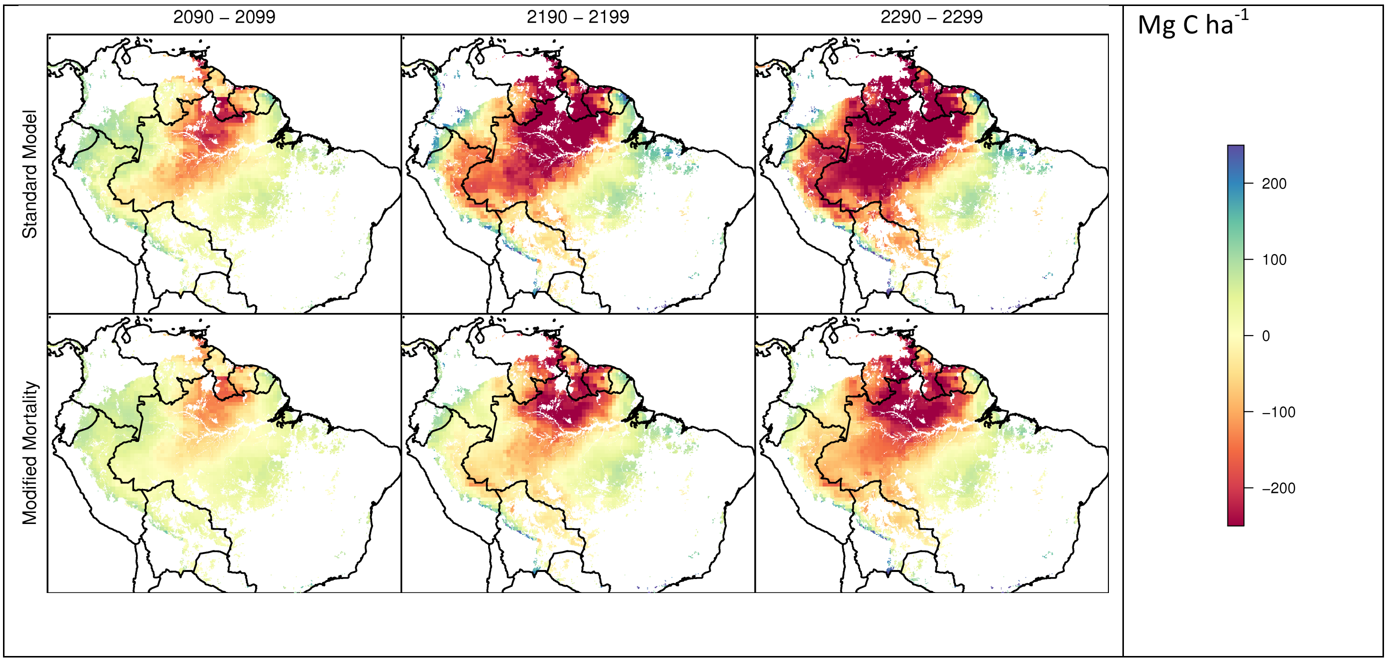

Fig. 4: Difference maps of AGB for standard mortality (upper row) and modified mortality (lower row). Baseline is 2000-2010, comparison to 2090-2099, 2190-2199 and 2290-2299 for RCP8.5

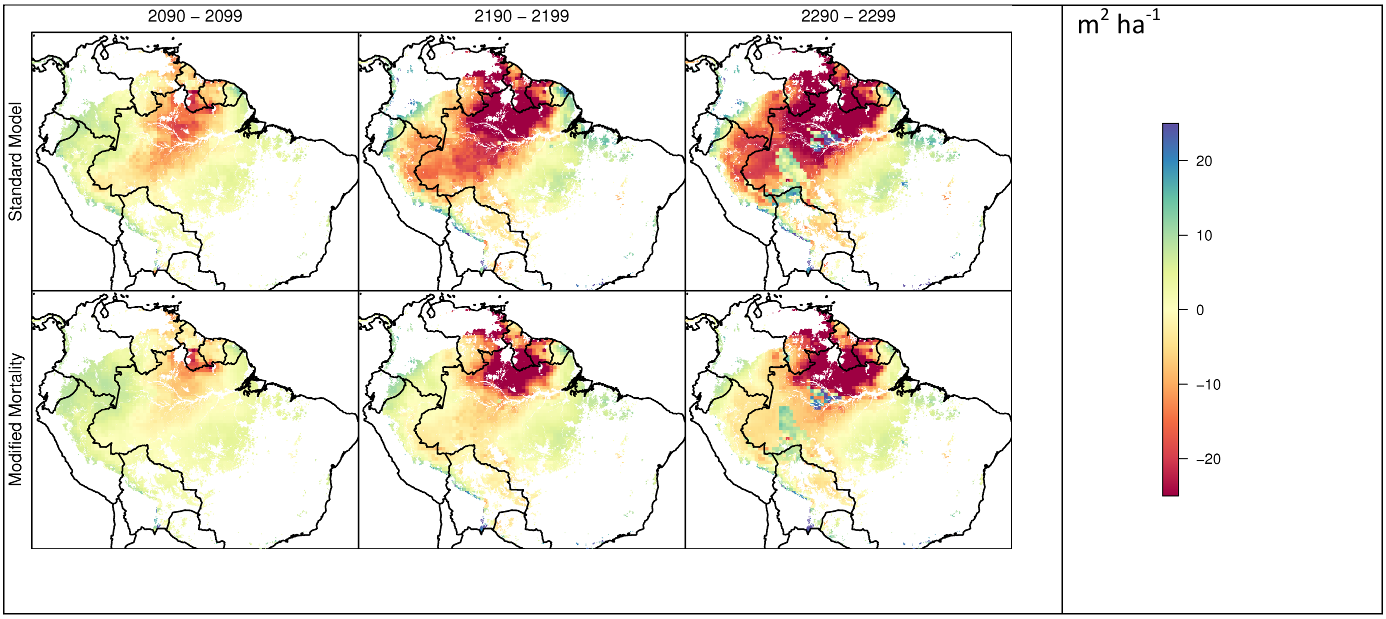

Fig. 5: Difference maps of basal area for standard mortality (upper row) and modified mortality (lower row). Baseline is 2000-2010, comparison to 2090-2099, 2190-2199 and 2290-2299 for RCP8.5

4. References

Brienen, RJW, Phillips, OL, Feldpausch, TR et al. (2015). Long-term decline of the Amazon carbon sink. Nature **519**(7543): 344-348.

FAO/IIASA/ISRIC/ISSCAS/JRC (2012). Harmonized World Soil Database (version 1.2). FAO, Rome, Italy and IIASA, Laxenburg, Austria.

Meinshausen, M, Smith, SJ, Calvin, K, Daniel, JS, Kainuma, MLT, Lamarque, JF, Matsumoto, K, Montzka, SA, Raper, SCB, Riahi, K, Thomson, A, Velders, GJM and van Vuuren, DPP (2011). The RCP green-

house gas concentrations and their extensions from 1765 to 2300. Climatic Change **109**(1-2): 213-241.

Mitchard, ETA, Feldpausch, TR, Brienen, RJW et al. (2014). Markedly divergent estimates of Amazon forest carbon density from ground plots and satellites. Global Ecology and Biogeography **23**(8): 935-946.

Pretzsch, H, Biber, P and Dursky, J (2002). The single tree-based stand simulator SILVA: construction, application and evaluation. Forest Ecology and Management **162**: 3-21.

Sitch, S, Smith, B, Prentice, IC, Arneth, A, Bondeau, A, Cramer, W, Kaplans, JO, Levis, S, Lucht, W, Sykes, MT, Thonicke, K and Venevsky, S (2003). Evaluation of ecosystem dynamics, plant geography and terrestrial carbon cycling in the LPJ dynamic global vegetation model. Global Change Biology **9**: 161-185.

13. SIMULATION RESULTS FROM PICUS

M.J. Lexer and W. Rammer

University of Natural Resources and Life Sciences (BOKU) Vienna,
Peter Jordan Straße 82, 1190 Wien, Austria

1. METHODS

1.1 FOREST MODEL

The model used for this study is the hybrid forest ecosystem model PICUS v1.5. The model is a hybrid of classical gap model components (PICUS v1.2, Lexer and Hönninger 2001) and process-based stand-level NPP algorithms. A detailed description of the model is provided in Seidl et al. (2005). PICUS simulates growth, regeneration and mortality of individual trees on a grid of 10 x 10 m² patches. Tree biomass is arranged in cells with a vertical depth of 5 m. A three-dimensional light model, allowing for the explicit consideration of direct and diffuse radiation within the canopy, is used to estimate absorbed radiation for each tree. Stand level productivity is estimated with a simplified model of light use efficiency, which depends on temperature, radiation, vapor pressure deficit, soil water, and nutrient supply. Redistribution of assimilates to individual trees, assuming fixed respiration rates, is accomplished according to the relative competitive success (i.e. biomass increment in the preceding year) of the individuals (see Lexer and Hönninger, 2001). The development of seedlings and saplings is modeled in a size class approach within five height classes. The PICUS model includes also a bark beetle disturbance module which (i) computes the stochastic infestation risk for simulated forest stands, (ii) estimates the damage intensity if an infestation occurs, and (iii) distributes the resulting tree mortality within the simulated stand. In the current study the disturbance module was switched off. The model requires information about the soil water storage capacity, the pH value of the mineral soil as well as plant-available nitrogen as a proxy for nutrient supply as well as a number of parameters for the soil submodel. The PICUS model in the current study was driven by monthly values of temperature, precipitation, solar radiation and vapor pressure deficit of the atmosphere. With the current model version stands of up to 25 ha can be simulated. A PICUS simulation can start from bare ground or with any defined stand structure. The initial state of a simulated stand (trees with DBH >1 cm) can be provided as a tree list and a related map with tree positions containing species, DBH and height for each individual or as a species specific DBH distribution and a height-diameter model. If no tree coordinates are available individuals can be distributed randomly or based on qualitative information about the mixture form (i.e. small groups, patches, etc.). Please note, that the population dynamics model, as well as the NPP module, do not distinguish the position of individual trees below the 100m^2 patch resolution. Regeneration as species-specific density (n/ha) in 5 height classes can be initiated as patchy pattern (100 m^2 resolution) or as a homogeneous regeneration layer throughout the simulated stand.

1.2 MORTALITY FORMULATIONS

The standard mortality algorithms (incl. parameter estimates) from PICUS v1.5 (Seidl et al. 2011) has been compared against the tree mortality function in PROGNAUS Monserud & Sterba (1999). The equations were developed using the extensive data set of the Austrian National Forest Inventory (ANFI).

The PICUS mortality algorithms are based on the classical gap model approach including (a) an intrinsic background mortality, and (b) a growth-related mortality which is applied for trees which fail to achieve a specific minimum diameter growth (absolute, relative) (see Table 1). A special feature of PICUS v1.5 is that the intrinsic mortality depends on the site quality (i.e. nutrient supply, water supply, temperature regime). The lower the average site quality, the lower the intrinsic background mortality and the older a stand will grow at relatively high stem densities.

Table 1. Parameter values for the PICUS mortality model. Agemax =maximum age of a species, stress years = number of years which a species can tolerate below the growth thresholds without increased mortality risk

species	agemax	Stress years	Threshold (abs.) [cm]	Threshold (rel.)
**Picea abies**	600	5	0.03	0.065
**Fagus sylvatica**	400	5	0.03	0.065
**Larix decidua**	750	5	0.02	0.040

The alternative PROGNAUS equation is given by

$$P = \left(1 + \mathrm{e}^{(b_0 + b_1/D + b_2 CR + b_3 BAL + b_4 D + b_5 D^2)}\right)^{-1} \qquad (1)$$

where

P = 5-year probability of death for an individual tree

D = DBH [cm]

CR = crown ratio [-]

BAL = basal area of all trees larger than the target tree (m^2/ha)

Parameter values for *Picea abies*, *Fagus sylvatica* and *Larix decidua* are provided in Table 2.

When extrapolating the equation to very small diameters, results show very high probabilities of tree death. We therefore applied the equation (1) in the PICUS implementation only for trees > 5 cm (which is also the lower limit of the inventory data used for fitting the model). Trees smaller than 5cm DBH are treated with the standard PICUS mortality algorithm.

Table 2. Parameter values for the PROGNAUS mortality model. See also Eq. 1

Species	b0	b1	b2	b3	b4	b5
**Picea abies**	2.1283	-10.0745	3.8251	-0.0186	0.0425	-0.00081
**Fagus sylvatica**	3.5734	-13.9542	3.1339	-0.0161	0	0
**Larix decidua**	4.407	-12.9395	2.2039	-0.0326	0	0

2. SIMULATION SETTINGS

2.1 SITE DATANER

We use 3 sites along an altitudinal gradient in the Eastern Alps in Austria. At each elevation, we defined sites with a water holding capacity of 200 mm and 100 mm, respectively. Available Nitrogen at both site variants is 62.5 kg/ha·yr. Historic climate based on the instrumental period 1961-1990 and a transient climate change scenario (A1B) for each of the sites is shown in Tables 3a-b. Both climate records were extended randomly to cover 200 years. The climate change scenario extended beyond 2100 by sampling randomly from the years 2080-2100.

Table 3a. Climatic averages for the baseline climate scenario (1961-1990) for the three sites. DJF = December-February; MAM = March-May; JJA = June-August; Son = September-November

site	Season	Temperature [°C]	Precipitation [mm]
**Low**	DJF	-0.84	243
	MAM	8.06	277
	JJA	16.96	365
	SON	8.56	236
**Mid**	DJF	-1.98	244
	MAM	5.67	265
	JJA	14.34	415
	SON	6.99	247
**High**	DJF	-4.02	234
	MAM	3.70	262
	JJA	12.45	452
	SON	5.36	263

Table 3b. Climate change signal for the used A1B climate scenario. Shown are the differences of the period 2080-2100 compared to the baseline (1961-1990). DJF = December-February; MAM = March-May; JJA = June-August; Son = September-November

Season	Temperature Delta [°C]	Precipitation Multiplier [-]
**DJF**	5.00	1.05
**MAM**	2.37	1.24
**JJA**	3.97	0.99
**SON**	3.97	1.04

Mixture types

Table 4 shows the initial mixtures at the three sites. The simulation runs are initialized with 4000 trees per ha in height class 30-50 cm (i.e. in the regeneration layer). At the site “low” (spruce - beech) and the site “high” (spruce – larch), the mixture is random.

Table 4. Site and mixture information for the simulation units. WHC = water holding capacity, Nav = plant available Nitrogen. PA = *Picea abies*, FS = *Fagus sylvatica*, LD = *Larix decidua*

site	altitude	WHC [mm]	Nav [kg ha^{-1} yr^{-1}]	Mixture [%]
**Low**	400m	100, 200	62.5	PA 50, FS 50
**Mid**	900m	100, 200	62.5	PA 100
**High**	1400m	100, 200	62.5	PA 50, LD 50

2.2 SIMULATION EXPERIMENTS

At each site the two WHC variants were simulated with the PICUS mortality model and the PROGNAUS mortality function starting with the respective mixture type extending over 200 years under baseline climate and under climate change scenario A1B. No active management intervention was included. Regeneration was enabled but was confined to seed produced in the simulated stand. No external seed input was allowed. In total 24 runs were implemented (3 x 2 x 2 x 2).

3. SIMULATION RESULTS

In general, both mortality algorithms produce quite similar results. Figure 1 shows the DBH distribution of the simulated stands after 200 years at the three sites. The main canopy trees (between 30-60cm DBH) show a very similar structure, density varies a bit with no clear trend for any of the two mortality models.

That the general mortality pattern is quite similar between he two models is also apparent in Figure 2. The original PICUS mortality algorithm produces a higher mortality rate in period 3 (100-150 yrs) compared to PROGNAUS mortality function, and the opposite pattern appears in period 4, with higher mortality rates for PROGNAUS.

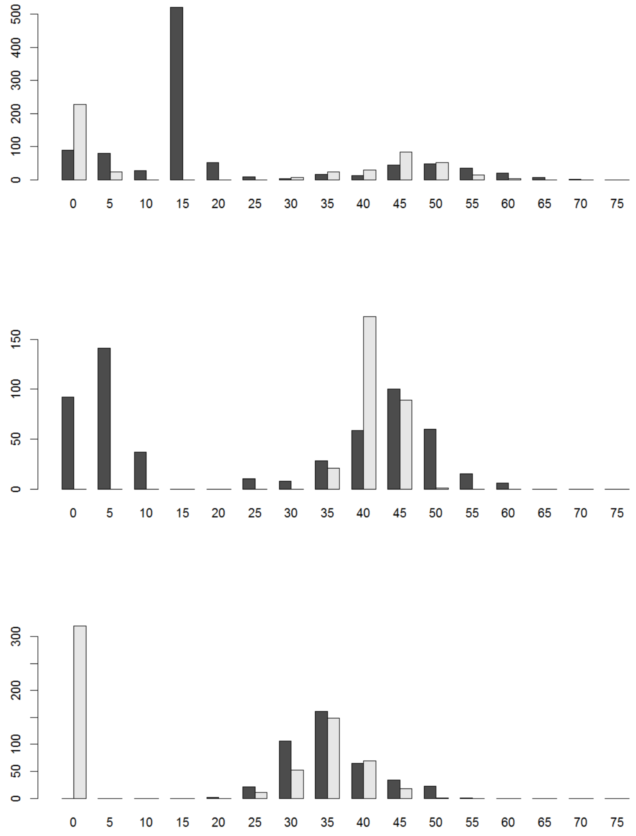

Figure 1. Diameter distribution of simulated stands in year 2200. Top panel: low elevation, mid panel: mid elevation, lower panel: high elevation. Dark grey = Prognaus mortality, light grey = standard PICUS mortality. WHC = 100 mm, baseline climate

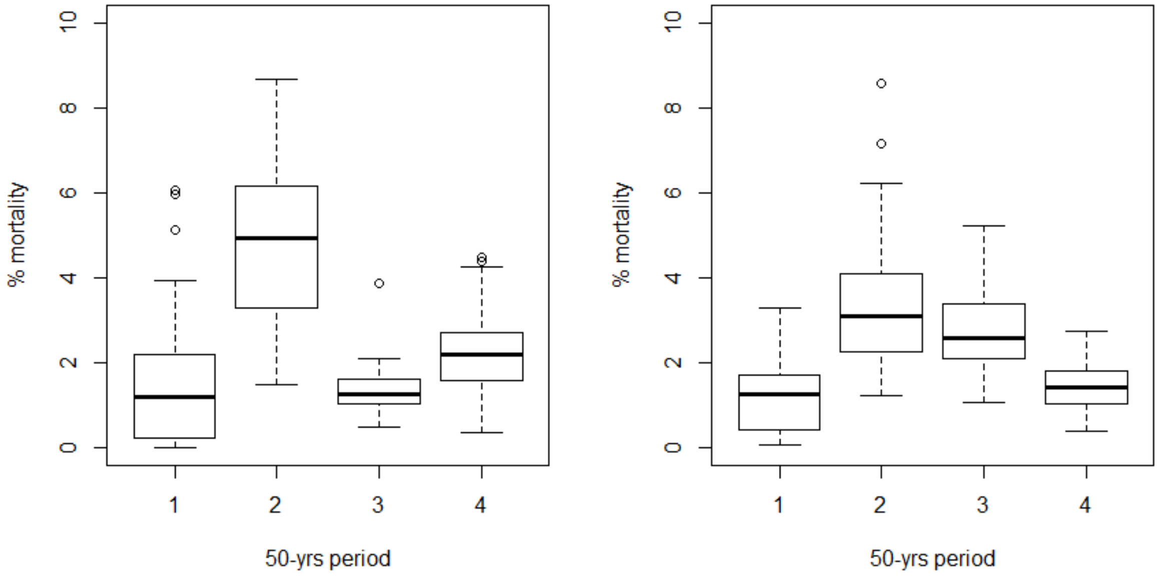

Figure 2. Percent of trees that died annually in the four 50-year periods for PICUS mortality algorithm (left) and PROGNAUS mortality function (right). Data are for the high-elevation site (baseline climate, 100 mm WHC). The mortality percentage is calculated as the fraction of trees that died relative to the total number of trees (per year)

Figure 3 indicates that the differences between the 100 m and 200 mm sites (WHC) are fairly small. Precipitation levels are in general so high that the reduction of summer precipitation under scenario A1B does not result in severe drought conditions. Consequently, the PICUS mortality model, which is sensitive to such „fast" drivers of mortality, is again very similar to the PROGNAUS algorithm.

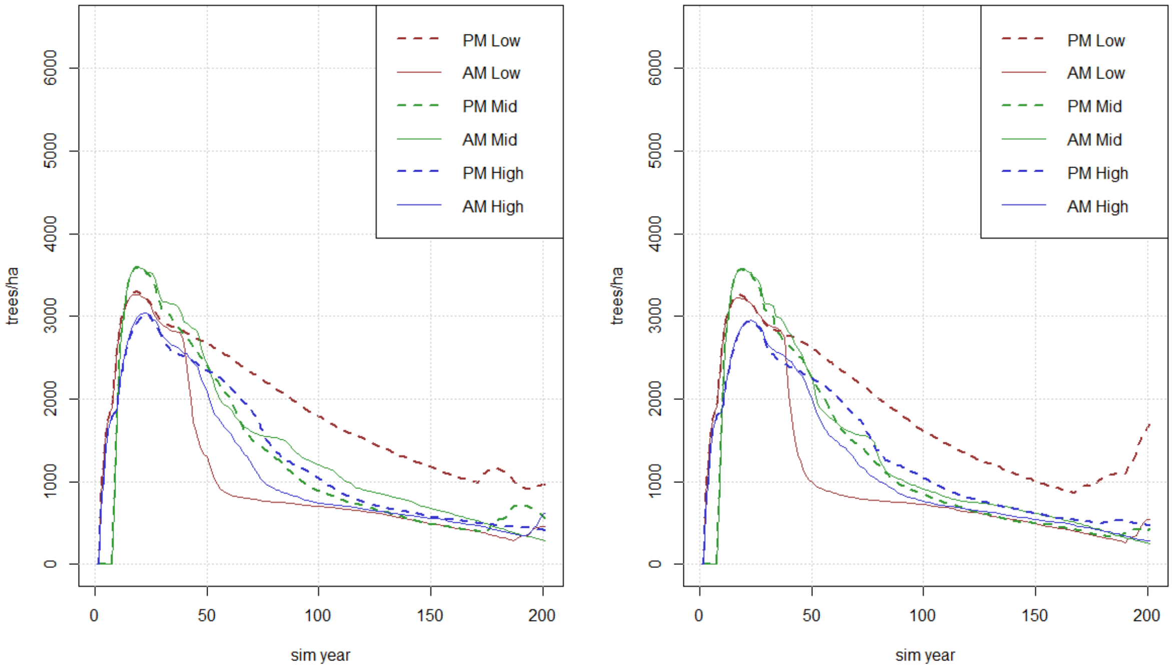

Figure 3. Development of stem numbers in simulated stands. Dashed: PROGNAUS Mortality, solid lines: PICUS. Red = low elevation, green = medium elevation, blue = high elevation. Left panel: WHC 100 mm, right panel: WHC=200mm

4. References

Lexer, M.J., Hönninger, K., 2001. A modified 3D-patch model for spatially explicit simulation of vegetation composition in heterogeneous landscapes. For. Ecol. Manage. 144: 43-65.

Monserud, R.A., Sterba, H. 1999. Modelling individual tree mortality for Austrian forest species. For. Ecol. Manage. 113: 109-123.

Seidl, R., Lexer, M.J., Jäger, D., Hönninger, K., 2005. Evaluating the accuracy and generality of a hybrid patch model. Tree Physiol. 25: 939–951.

Vospernik, S., Monserud, R.A., Sterba, H., 2015. Comparing individual tree growth models using principles of stand growth for Norway spruce, Scots pine, and European beech. Can. J. For. Res. 45: 1006-1018.

14. SIMULATION RESULTS FROM SIBYLA

Ján Merganič[1, 3], Katarína Merganičová[1,3], Marek Fabrika[1], Slavomír Strmeň[2], Adriana Leštianska[1], C.P.O. Reyer[4], Katarína Střelcová[1]

[1]Technical University Zvolen, T.G.Masaryka 24, 960 01 Zvolen, Slovakia

[2]National Forest Research Centre, T.G.Masaryka 22, 960 92 Zvolen, Slovakia

[3]Forest Research Inventory and Monitoring, Huta 14, 962 34 Železná Breznica, Slovakia

[4]Potsdam Institute for Climate Impact Research, Telegrafenberg, P.O. Box 601203, 14412 Potsdam, Germany

1. METHODS

1.1 FOREST MODEL

SIBYLA is the simulator of forest biodynamics. It belongs to the category of semi-empirical tree growth simulators. It consists of the set of mathematical models and algorithms that are transformed into an integrated software package SIBYLA Suite. The model has been developed at the Department of Forest Management and Geodesy, Technical University in Zvolen, Slovakia. The basis of the model was the modeling principle and algorithms implemented in SILVA 2.2 (Pretzsch 1992, Kahn 1994). The model is sensitive to climatic factors (days of vegetation season, mean temperature during vegetation season, annual temperature amplitude, and total precipitation during vegetation season). The climatic factors modify height and diameter growth potential, and consequently tree increment. The climatic factors also influence tree mortality model. More detailed information about Sibyla can be found in Fabrika (2005, 2007), http://etools.tuzvo.sk/sibyla/english/model.htm.

1.2 MORTALITY FORMULATIONS

For the simulations, we used two empirically derived statistical mortality models.

Standard mortality model in Sibyla

The model of natural tree mortality implemented in Sibyla was adopted from SILVA 2.2 (Ďurský 1997, Ďurský et al. 1996). The model is always activated at the beginning of a 5-year simulation interval, and consists of two parts: a model simulating the probability of tree survival, and a model simulating the threshold stand density.

The model of tree survival probability is based on the logistic regression, which is a function of survival probability (function F-logit). The variables included in the model are the tree diameter at breast height (D), the annual increment of tree basal area (ig), tree height (h), and the absolute height (site) class AVB50. Absolute height (site) class is expressed as a potential top height in the simulation plot reached at the age of t = 50 years. The function of tree survival probability is then transformed to the function of tree mortality. The obtained value Mrt% is then compared with the random number drawn from the uniform distribution from the interval <0;100). The tree i dies if the calculated value exceeds the random number. The age at which the tree dies is randomly generated from the 5-year interval.

The model of threshold stand density is based on the reduction of the stand basal area, if this exceeds the maximum possible level. First, maximum stand basal area is calculated as a weighted arithmetic mean of maximum basal areas of individual tree species (j) in the stand, while tree crown projections (cdi2) are used as the weights. Maximum stand basal area of a particular tree species is calculated from its top height (h95%). Next, stand basal area of dying trees is obtained from the actual stand basal area (G) and the stand basal area of dead trees (Gdead) as follows:

Gmort = G - Gmax - Gdead ; if Gmort > 0

Afterwards, the trees to be removed are selected according to the following rules:

The total basal area of selected trees has to be equal to or slightly greater than Gmort .

1. First, the trees with the greater probability of mortality than the predefined threshold (by default 50%) are selected, starting from the highest value Mrt%.

2. If it is necessary, the trees with the lower probability of mortality than the threshold value are also selected using random sampling.

The age of tree death is randomly generated from 5-year interval.

More detailed information about the mortality model implemented in Sibyla can be found in Fabrika (2005), and http://etools.tuzvo.sk/sibyla/english/model.htm.

Mortality model according to Monserud and Sterba (1999)

The probability of mortality was modeled using a logistic equation. Independent variables that enter the model are diameter at breast height (D in cm), crown ratio (CR, dimensionless ratio of the crown length to total tree height), and basal area in larger trees (BAL in $m^2.ha^{-1}$). Diameter enters the model in three different forms: in its original form (i.e. D), as hyperbolic transformation (1/D), and as quadratic transformation (D^2). The probability of mortality is calculated for a period of 5 years.

The model was parameterized using the data from the Austrian National Forest Inventory. The parameters of the logistic equation were estimated using maximum likelihood methods. More information about the model can be found in Monserud and Sterba (1999).

2. SIMULATION SETTINGS

2.1 SITE DATA

For the simulations, we used Predmier I research plot (49°24’ N, 18°35’E, 500 m a.s.l.) situated in the Kysuce – Western Beskids protected landscape area (Fig. 1). The plot is 0.2592 ha large, located at an elevation of 500 m a.s.l. on a mild 10% slope with eastern aspect. Geological substrate is flysch with varying portions of sandstones, claystones, and marlites. The soil type is a cambisol podzol with a depth of about 90 cm. From the climatic point of view, this area is situated in the temperate climatic zone with temperate Central-European climate. The area belongs to a mildly warm region with mean air temperature of 6.7 °C and mean precipitation totals of 875 mm per year (Fig. 2).

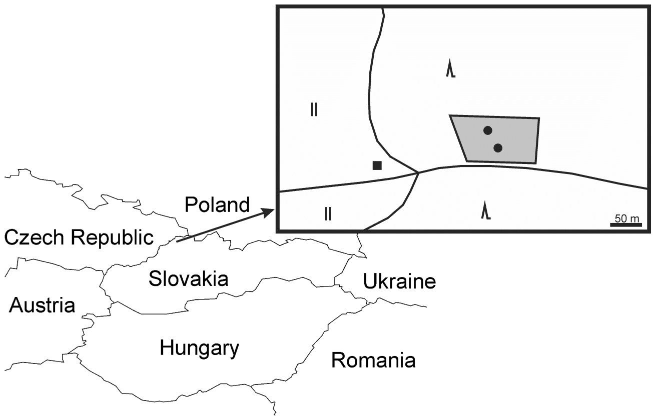

Fig. 1. Location of Predmier I research plot. The black square indicates the position of the weather station

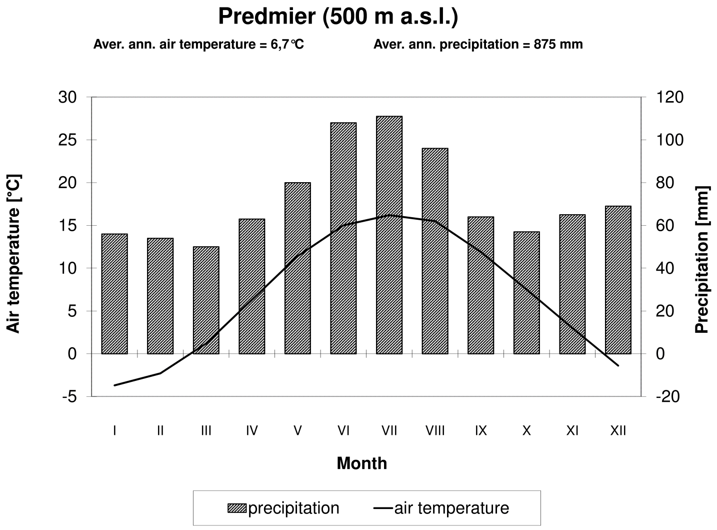

Fig. 2. Climate specification of the region; long-term average monthly data (1961–1990) – air temperature from Čadca weather station (423 m a.s.l.) and precipitation from Turzovka weather station (465 m a.s.l.)

The forest stand in the research plot is a pure even-aged stand composed of Norway spruce. It was established from vegetative reproduction in 1989, when three-year-old cutting transplants were planted systematically in a net of 1.5x1.5 m. The transplants were taken from the parent stand situated in the Western Beskids at 800 m a.s.l. at a southern slope. From each selected tree of the parent stand, 8 transplants were taken and planted at the research plot (Chlepko 1993 ex Strmeň 2004).

The plots were re-measured 5 times (1989, 1994, 2000, 2007, 2014). For the purpose of this study, we used the measurements from the last three inventories because of the model limitations to simulate trees smaller than 1.3m.

Site specification

The growth simulator SIBYLA uses the model of ecological classification applied in the growth simulator SILVA 2.2, which was derived by Kahn (1994). Site is specified using ecological site characteristics describing climate, air, and soil, also called site variables:

- s1 (N2O) ... NOx concentration in air (ppb)
- s2 (CO2) ... CO2 concentration in air (ppm)
- s3 (NUTR) ... soil nutrient supply (relative value in the range from 0 to 1)

- s4 (DAYS) ... number of days in the vegetation period (days with daily mean temperature above 10°C)
- s5 (TAMPL) ... annual temperature amplitude (the difference between minimum and maximum monthly temperature in °C)
- s6 (TEMP) ... daily mean temperature in the vegetation period in °C (from April to September)
- s7 (MOIST) ...soil moisture (relative value in the range from 0 to 1)
- s8 (PRECIP) ... precipitation amount in the vegetation period in mm (from April to September)
- s9 (ARID)... aridity index according to de Martone in mm.°C-1

These factors directly influence the production capacity of a stand (i.e., tree height and diameter increment).

Current climate data

Long-term averages (1961–1990) were available from two weather stations – air temperature from Čadca weather station (423 m a.s.l.) and precipitation from Turzovka weather station (465 m a.s.l.). Short-term climate data (2008-2012) were taken from the portable weather station (Environmental Measuring Systems, Brno, www.emsbrno.cz) situated at an open space close to Predmier I research plot (approximately 200 m from the plot) at 2 m height (Fig. 1).

Future climate data

We used three different climate scenarios, one representing constant climate (reference scenario, hereafter called as SC1), and two different climate change scenarios, which were derived on the base of two emission scenarios: low-end IMAGE-RCP3-PD(2.6), and high-end MESSAGE-RCP8.5. The climate change scenarios were obtained from HADGEM2-ES Model. They are defined below and in Table 1 as:

SC1: Constant reference climate
SC2: RCP2.6 baseline
SC3: RCP8.5

Tab. 1: Environmental driving forces of the simulations. The values refer to the year 2100

	Temperature (delta values, °C)				Precipitation (multipliers, unitless)				CO2 (ppm)
**Scenario**	DJF	MAM	JJA	SON	DJF	MAM	JJA	SON	
**RCP2.6**	2.65	2.18	3.70	1.79	1.04	0.99	0.72	0.98	371
**RCP8.5**	6.97	5.51	9.97	7.14	1.25	1.08	0.51	0.78	929

2.2 Test data

Model behavior was tested using the data from the research plot representing the two observation periods 2000-2007 and 2007-2014. We compared simulated mortality with the observed mortality both from the point of number of trees and basal area. The results shown in Fig. 3a suggest that the mortality simulated with the default mortality model in SIBYLA was not significantly different from the observed values.

The validation of the second mortality model of Monserud and Sterba (1999) showed that in the first period the model overestimated the actual mortality rate, whereas in the second period it underestimated the mortality rate calculated either from the number of trees or basal area (Fig. 3b). Due to this, we did not reparametrize the model, because in the first period we would have to decrease the

mortality rate, while in the second period the increase would be necessary. This means that no general modification of the model could be performed. Therefore, we used the original model as published by Monserud and Sterba (1999) without any modifications.

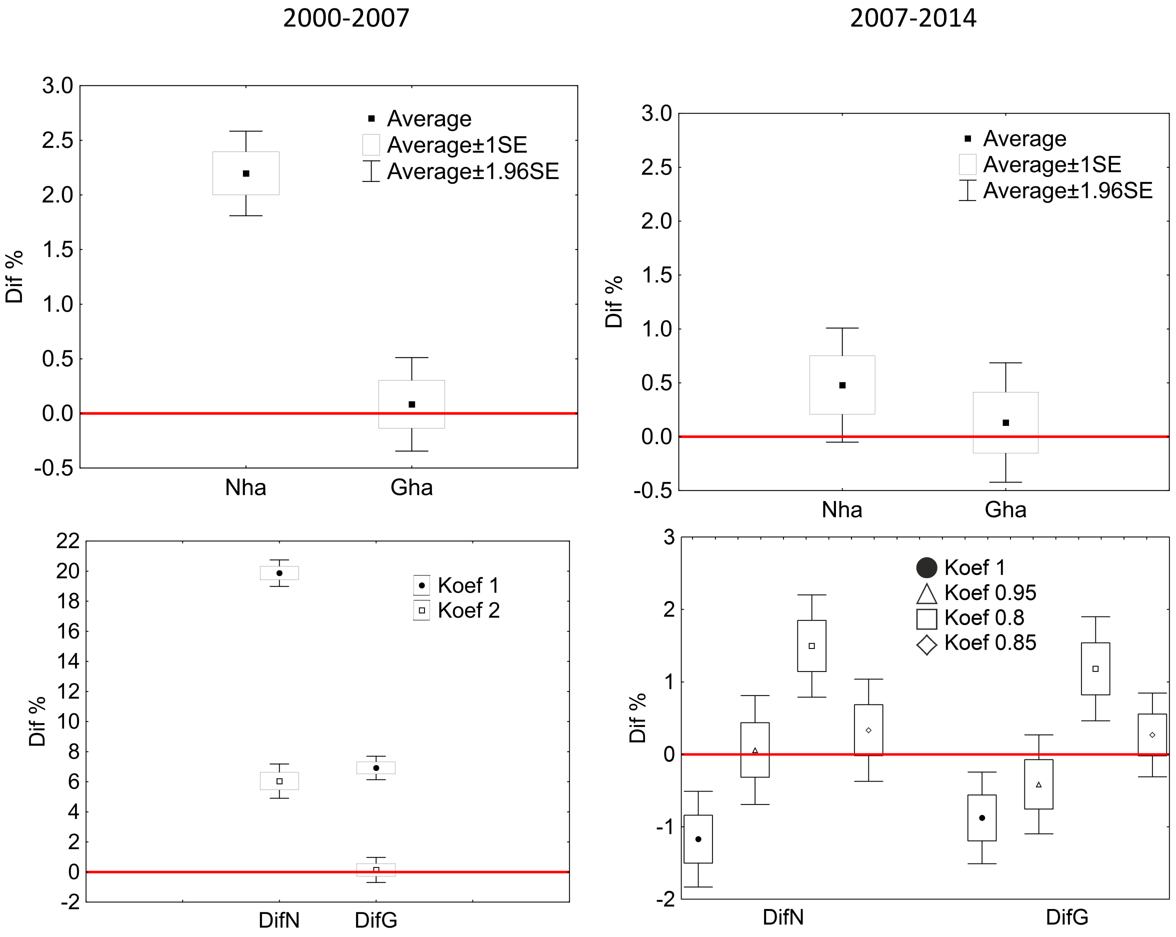

Fig. 3: Evaluation of the original mortality model in Sibyla (top row) and the mortality model of Monserud and Sterba (1999) (bottom row). Left: Period 2000-2007; right: period 2007-2014. Percent differences are shown with respect to stem numbers (Nha and DifN, respectively) and basal area (Gha and DifG, respectively). Since the Monserud and Sterba (1999) model performed much worse than the original Sibyla model, a correction using a multiplier (Koef) was tried for the two periods, where "Koef *x*" indicates a multiplier of *x*. Since the multipliers for the two periods would be very different, no correction was attempted for the remaining simulations.

2.3 *Simulation experiments*

The simulation was initialized with the data measured in the year 2000, because in the measurements before 2000 (i.e. 1989 and 1994), most trees were smaller than 1.3 m in height, which the model Sibyla cannot simulate. The plot was simulated as an unmanaged plot, where only natural mortality occurs.

The simulations were performed for a period of 200 years, i.e. until the year 2200. The climatic conditions were changed according to the predefined scenarios until 2100. After this year, the climatic conditions were randomly chosen from the last twenty years before the year 2100.

3. SIMULATION RESULTS

The comparison of the development of the stand basal area over the simulated period under two different mortality models is shown in Fig. 4.

SC1: Reference climate

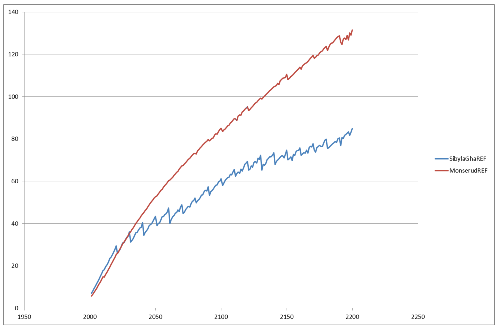

SC2: RCP2.6 baseline

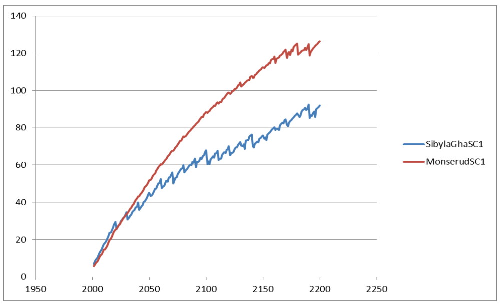

SC3: RCP8.5

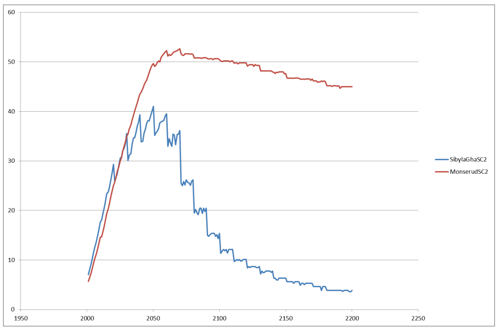

Fig. 4 Stand basal area (G) under three pre-defined climate change scenarios and two different mortality models

As can be seen from Fig. 4, basal area produced by the default mortality model of Sibyla is consistently lower than the stand basal area obtained with Monserud and Sterba mortality model.

In the case of mortality represented by the number of dead trees, the model outputs differ in the first half of the simulation period (Fig. 5), while in the second half of the simulation period, the mortality estimates do not differ significantly between the two models. Sibyla mortality model tends to simulate higher mortality rates calculated both from the number of trees and basal area than the mortality model by Monserud and Sterba (Fig. 5).

Number of trees

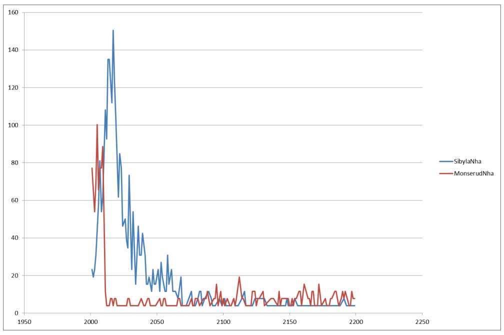

Basal area

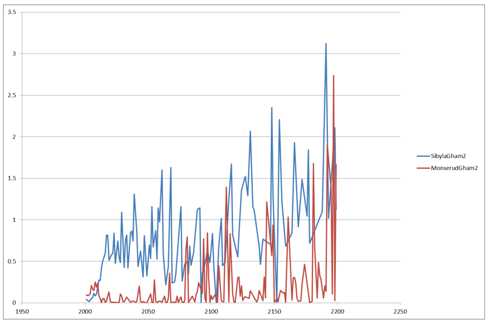

Fig. 5 Mortality represented by the number of trees and basal area (G) over the whole simulation period for the reference climate and two mortality models

The reason for higher mortality rates obtained by the default mortality model in Sibyla is that the default model tends to remove trees with greater diameters from the stand. Diameter frequency distribution of trees that died is shifted to the right in comparison to diameter frequency distribution obtained when the Monserud and Sterba (1999) mortality model was applied (not shown here).

4. REFERENCES

Fabrika, M., (2005): Simulátor biodynamiky lesa SIBYLA, koncepcia, konštrukcia a programové riešenie. Habilitačná práca. Technická univerzita vo Zvolene, 238 p.

Ďurský, J., (1997): Modellierung der Absterbeprozesse in Rein- und Mischbeständen aus Fichte und Buche. Allg. Forst- und Jagdzeitung, 168. Jg., H. 6/7: 131-134.

Ďurský, J., Pretzsch, H., Kahn, M., (1996): Modellhalfe Nachbildung der Mortalität von Fichte und Buche in Einzelbaumsimulatoren. Jahrestagung 1996 der Sektion Ertragskunde des DVFFA in Neresheim, Tagungsber., pp. 267-277.

Pretzsch, H., (1992): Konzeption und Konstruktion von Wuchsmodellen für Rein- und Mischbestände. Forstliche Forschungsberichte München, Nr.115, 358 p.

Kahn, M., (1994): Modellierung der Höhenentwicklung ausgewählter Baumarten in Abhängigkeit vom Standort. Forstliche Forschungsber. München, Vol. 141, 221 p.

Monserud R.A., Sterba H. (1999): Modeling individual tree mortality for Austrian tree species. Forest Ecology and Management 113: 109-123.

Strmeň S. (2004): Stav autovegetatívneho smrekového porastu 11 rokov po výsadbe v imisiami zasiahnutej oblasti Kysúc. Forestry journal, 50 (1): 41–52.

15. Simulation results from xComp

Tobias Mette

Bavarian State Institute of Forestry (LWF), Soil and Climate Department, 85354 Freising, Germany

1. Methods

1.1 Forest model

xComp is an individual tree based (ITB) forest growth model. It was developed for monospecific *Nothofagus* forests in Patagonia, South America. Like most ITB models the strength of xComp lies in (1) position dependent competition sensitivity which allows simulating the effect of different thinning regimes or disturbances, (2) output of single tree dimensions which makes it applicable in forestry practice, and (3) a simple parameterization, especially a stable mortality self-calibration (Mette 2014).

In xComp, growth and mortality are functions of site index (SI), a measure based on an asymptotic age-height relation. To make xComp climate-sensitive, a simple relation of SI to temperature and precipitation is established. The national forest inventory for Germany and the WorldClim climate serve as input data. In addition to the competition based mortality which affects the suppressed trees, a new "height-antagonistic" mortality is introduced. The idea behind this mortality is that when changing climate induces a decrease in SI, trees that exceed the SI-dependent maximum height have a higher mortality. In contrast to the competitive mortality, the height-antagonistic mortality affects dominant trees and thereby can simulate drastic dieback scenarios.

Model parameterization and algorithms

Norway spruce growth is parameterized according to the yield tables of Assmann and Franz (1963). Norway spruce mortality is calibrated according to the diameter-tree number relations Pretzsch and Biber (2005). Mortality is formulated at tree-level as a diameter (dbh) and competition (comp) dependent logistic function:

$$mortality = \frac{1}{exp(f(comp, dbh)) + 1}$$

A numerical optimization searches a solution that fulfills a size-frequency relation at stand level, according to Reineke (1933):

$$N = sdi \cdot (dq/25)^{-rk}$$

With the variables N = tree number/ ha, dq = root mean square diameter of the stand, and the user-defined parameters sdi = stand density index, rk = exponent.

Growth and mortality parameters are stored as diameter- and competition-dependent functions of site index (SI). SI is defined by stand height at an age of 100 yrs (unit: *meter*) according to the Chapman-Richards equation:

$$height = A \cdot (1 - \exp(-k \cdot age))^p$$

where height and age are stand-level variables, and A, k, p are regression parameters: A is the height asymptote (maximum height), k the growth rate and p a sigmoidality term.

1.2 Mortality Formulation

Height-antagonistic mortality

In the frame of PROFOUND, an alternative *height-antagonistic* mortality has been designed which replaces the competitive mortality when active. Height antagonistic mortality is assumed to occur if the height by which a tree exceeds the SI-determined maximum height. It is 0 for trees lower than the maximum height, and approaches 1 as the tree:

$$height\ ant.mortality = 0.011 \cdot (height - max.height)^{1.5}$$

Site index relation to temperature and precipitation

Norway spruce site index is related to temperature and precipitation. The national forest inventory for Germany (BMEL 2016) and the WorldClim climate (Hijmans et al. 2005) serve as input data. SI is determined from the NFI data as described in Kölling et al. (2016); temperature is represented by mean summer temperature T_{JJA}, precipitation by summer precipitation sum P_{JJA}. For the temperature-precipitation space that is realized by the NFI a generalized additive model (gam) is used to explain SI from T_{JJA} and P_{JJA}. Also, a simple second-order linear model (lm) is employed to predict the SI outside the temperature and precipitation space of the NFI. The SI decrease for temperatures above 19 °C was accelerated to approximately 4 m/ 1 °C. Where T_{JJA} exceeds 17 °C, spruce is increasingly replaced by Scots pine, above 18.5 °C also sessile and pedunculated oak. Spruce reaches its distribution limit at T_{JJA} ~19 °C. Fig. 1 summarizes the SI model visually.

2. Simulation Settings

2.1 Site data

Simulations for the PROFOUND mortality evaluation are carried out exemplary for a 34 yr old even-aged Norway spruce stand in South-West Germany (725 m a.s.l.). The second NFI documents the stand with following parameters (reference year 2002): **N/ ha = 1925, basal area = 56 m2/ ha, standing volume over bark = 436 m3/ ha**. A **dominant height of 21.8 m**, a **dominant diameter of 23.8 cm** and a **site index of 35.1 m** at an age of 100 yrs are derived from the sample tree data. Regionalized temperature data estimate a summer temperature T_{JJA} of 15.5 °C (T_{YR} = 7.5 °C) for the climate reference period 1985-2010. Summer precipitation P_{JJA} is estimated at 325 mm (P_{YR} = 1040 mm) from WorldClim data for the period “current”.

2.2 Simulation experiments

Climate change scenarios are taken from WorldClim. Summer temperature T_{JJA} and precipitation P_{JJA} are available as periodic averages for the periods “current” and “2061-2080”. Comparison with regionalized climate data shows that the WorldClim period “current” corresponds well with the 1971-2000 average. To interpolate between “current” and “2061-2080” and extrapolate until 2200, T_{JJA} and P_{JJA} are assumed to approach an asymptotic value. The asymptote is fixed by multiplying the difference for T_{JJA} and P_{JJA} between “current” and “2061-2080” by a factor of 1.35. Using this asymptotic function temperature and precipitation trajectories are calculated in 5yr intervals from 2000-2200. To initialize the simulations with the temperature and precipitation for the reference year 2000, the temperature trajectory is adjusted by the regionalized climate data average for 1981-2010. The simulations are run for one baseline scenario (no climate change) and the RCP 4.5 and RCP 8.5

scenarios of three climate models of different intensities: “mg” weak, “no” moderate, ”gf” strong. Tab. 1 and Fig. 1 summarize and visualize the temperature-precipitation trajectories.

Stand management and regeneration: No stand management is applied, i.e. the spruce stand develops naturally for 200 years. The final age of ~230 years in 2200 is still far from the potential age for Norway spruce. Regeneration is neglected since stands where no “height antagonistic dieback” occurs remain closed, and stands where “height antagonistic dieback” occurs are unlikely to regenerate with spruce.

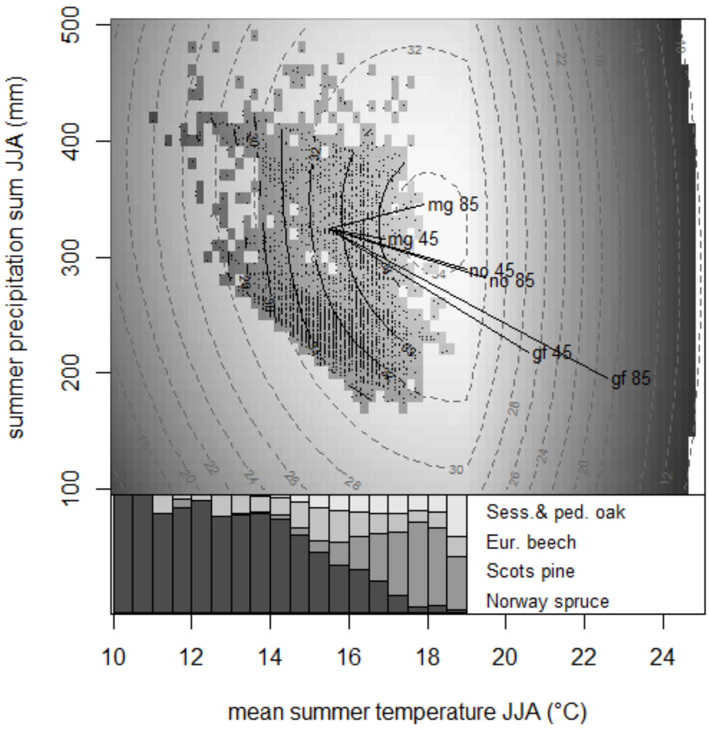

Fig. 1: Norway spruce site index (SI) in temperature-precipitation space. Realized data space in the German NFI with prominent colors, dots for data points and solid SI-isolines based on data interpretation with a GAM. Extrapolation space outside the data with weak colors and SI-isolines based on data interpretation with a linear model (modified above 19 °C T_{JJA}). Temperature and precipitation trajectories from 2000-2200 for Norway spruce site 3845.3 that is used for the PROFOUND mortality simulation

Table 1: Temperature, precipitation trajectories from 2000-2200 for the Norway spruce NFI site 3845.3 (WorldClim data, extrapolated beyond 2070, temperatures corrected from regionalized temperature data)

Model+RCP	ΔT_{YR} (2000-2100)	T_{YR} (°C)			P_{YR} (mm)			TJJA (°C)			PJJA (mm)		
		2000	2100	2200	2000	2100	2200	2000	2100	2200	2000	2100	2200
**no change (baseline)**	0 °C	7.5	7.5	7.5	1040	1040	1040	15.5	15.5	15.5	325	325	325
**mg45 (weak)**	+1.2 °C	7.5	8.7	8.9	1040	1103	1116	15.5	16.7	16.9	325	316	316
**mg85 (weak)**	+2.1 °C	7.5	9.6	10.1	1040	1138	1159	15.5	17.5	17.9	325	341	346
**no45 (mod.)**	+2.1 °C	7.5	9.6	10.1	1040	1023	1020	15.5	18.5	19.1	325	295	290
**no85 (mod.)**	+2.8 °C	7.5	10.3	10.9	1040	1076	1083	15.5	18.8	19.5	325	287	281
**gf45 (strong)**	+3.1 °C	7.5	10.6	11.2	1040	1019	1014	15.5	19.8	20.6	325	234	217
**gf85 (strong)**	+4.6 °C	7.5	12.1	13.1	1040	983	971	15.5	21.4	22.7	325	215	194

3. Results

Starting the simulation from a moderate mean summer temperature of 15.5 °C in 2000, only the most severe climate change scenarios (gf45 and gf85) lead to a dieback. In these scenarios, mean summer temperatures in 2200 rise to 20.6 °C and 22.7 °C, respectively. In the other scenarios, the temperature rise barely exceeds the current temperature range of spruce in the German NFI. Since the site index rises with temperature, and abiotic and biotic risks are excluded, the simulations of these more moderate scenarios lead to giant dense forests with volume accumulation above 1500 m3/ ha.

The contribution points out the intriguing phenomenon that higher temperatures enhance the SI, i.e. growth, while at the same time the spruce presence practically decreases to 0. It is reasonable to assume that biotic risks, mainly the bark beetle, outweigh the expected higher yield. These risks also make it difficult to verify the existence of a height-antagonistic mortality, not to speak of its parameterization. Empirically, it may be promising to look at climates with stronger periodic climate oscillations. Theoretically, carbon balance models with individual tree allocation algorithms could advance the proposed idea of a height antagonistic mortality.

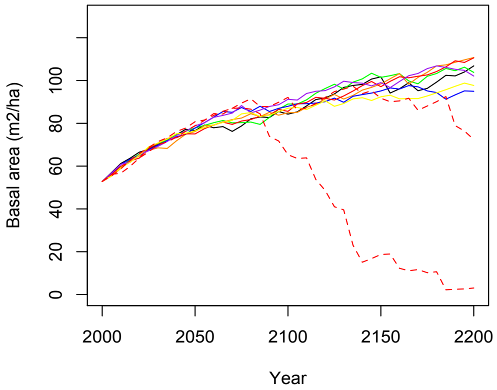

Fig. 2: Basal area development of the simulated Norway spruce stand under six climate change scenarios (+ one baseline scenario without climate change). All climate scenarios lead to very similar results; only the most severe climate change scenarios (gf45 and gf85) lead to a strong BA decline through the dieback of upper canopy trees if the "height-antagonistic" mortality algorithm is used (dashed lines)

4. References

Assmann E, Franz F (1963). Vorläufige Fichtenertragstafel für Bayern. Inst. f. Ertragskunde der forstl. Versuchsanstalt München.

BMEL Bundesministerium für Ernährung und Landwirtschaft (2016). Bundeswaldinventur (BWI). https://www.bundeswaldinventur.de/ (last called 24.01.2018)

Kölling C, Mette T, Knoke T (2016). Waldertrag und Anbaurisiko in einer unsicheren Zukunft. SZF 167, 29-38. doi: 10.3188/szf.2016.0029

Mette T (2014). Modelling Patagonian Lenga-forest dynamics (Nothofagus pumilio) in Chile - Final Report. DFG - Proj.Nr. 192579022 (available upon request from DFG or author)

Pretzsch H, Biber P (2005). A re-evaluation of Reineke's rule and stand density index. Forest science, 51(4), 304-320.

Reineke LH (1933). Perfecting a stand density index for even-aged forests. Journal of Agricultural Research, 46:627-638.

Hijmans RJ, Cameron SE, Parra JL, Jones PG, Jarvis A (2005). Very high resolution interpolated climate surfaces for global land areas. International journal of climatology, 25(15), 1965-1978.
